# A proof of concept for a targeted enrichment approach to the simultaneous detection and characterization of rickettsial pathogens from clinical specimens

**DOI:** 10.3389/fmicb.2024.1387208

**Published:** 2024-04-10

**Authors:** Adrian C. Paskey, Kevin L. Schully, Logan J. Voegtly, Catherine E. Arnold, Regina Z. Cer, Kenneth G. Frey, Paul W. Blair, Danielle V. Clark, Hong Ge, Allen L. Richards, Christina M. Farris, Kimberly A. Bishop-Lilly

**Affiliations:** ^1^Genomics and Bioinformatics Department, Biological Defense Research Directorate, Naval Medical Research Command, Frederick, MD, United States; ^2^Leidos, Reston, VA, United States; ^3^Austere Environments Consortium for Enhanced Sepsis Outcomes (ACESO), Biological Defense Research Directorate, Naval Medical Research Command, Frederick, MD, United States; ^4^Defense Threat Reduction Agency, Fort Belvoir, VA, United States; ^5^The Henry M. Jackson Foundation for the Advancement of Military Medicine, Bethesda, MD, United States; ^6^Viral and Rickettsial Diseases Department, Infectious Diseases Directorate, Naval Medical Research Command, Silver Spring, MD, United States

**Keywords:** rickettsial pathogens, targeted enrichment, detection, characterization, qPCR, high throughput sequencing

## Abstract

Infection with either *Rickettsia prowazekii* or *Orientia tsutsugamushi* is common, yet diagnostic capabilities are limited due to the short window for positive identification. Until now, although targeted enrichment had been applied to increase sensitivity of sequencing-based detection for various microorganisms, it had not been applied to sequencing of R. prowazekii in clinical samples. Additionally, hybridization-based targeted enrichment strategies had only scarcely been applied to qPCR of any pathogens in clinical samples. Therefore, we tested a targeted enrichment technique as a proof of concept and found that it dramatically reduced the limits of detection of these organisms by both qPCR and high throughput sequencing. The enrichment methodology was first tested in contrived clinical samples with known spiked-in concentrations of *R. prowazekii* and *O. tsutsugamushi* DNA. This method was also evaluated using clinical samples, resulting in the simultaneous identification and characterization of *O. tsutsugamushi* directly from clinical specimens taken from sepsis patients. We demonstrated that the targeted enrichment technique is helpful by lowering the limit of detection, not only when applied to sequencing, but also when applied to qPCR, suggesting the technique could be applied more broadly to include other assays and/or microbes for which there are limited diagnostic or detection modalities.

## 1 Introduction

The *Rickettsiaceae* comprise a family of gram-negative obligate intracellular bacteria that has areas of endemicity on every continent except Antarctica. *Rickettsia prowazekii*, a member of the globally distributed typhus group of rickettsiae, is the etiologic agent of epidemic typhus, vectored by the human body louse (*Pediculus humanus*). *Orientia tsutsugamushi*, the etiologic agent of scrub typhus, is transmitted by trombiculid mites (particularly by the larva stage known as chiggers) and is historically distributed across approximately the Tsutsugamushi Triangle, an area spanning 13,000,000 km^2^ from South Asia in the west to Eastern Russia and Japan in the east and south to Australia. More recently, scrub typhus has been confirmed outside of the Tsutsugamushi Triangle including South America, Sub-Saharan Africa, and the Middle East with etiologic agents such as *Candidatus Orientia chiloensis* and *Candidatus Orientia chuto*, significantly expanding the distribution and risk of scrub typhus (Kelly et al., 2002; Abdad et al., 2018; Jiang et al., 2022).

Due to the broad geographic distribution of these pathogens and their vectors, billions of people are at risk for infection. Historically, outbreaks of epidemic typhus have occurred in overcrowded or unhygienic environments, such as jails and other resource-limited settings including war, societal collapse, or natural disasters (Perine et al., 1992; Badiaga and Brouqui, 2012; Nyatanyi et al., 2016). Following an incubation period of 7–14 days, *R. prowazekii* infection causes a high fever and severe headache. Patients may also present with myalgias, dry cough, and delirium. A key feature is a dull red rash that begins on the trunk and spreads peripherally, sparing the soles and palms. If left untreated, mortality can be as high as 60% (Akram et al., 2021). *O. tsutsugamushi* is responsible for nearly one-quarter of febrile illnesses in endemic areas and mortality can be as high as 30% without proper treatment (Chattopadhyay and Richards, 2007; Sriwongpan et al., 2013; Griffith et al., 2014). Recent evidence of the acute manifestation of scrub typhus is characterized by sudden onset of fever approximately 6–21 days after exposure with chills, headache, backache and myalgia, profuse sweating, vomiting and enlarged lymph nodes. In some patients, an eschar may develop at the site of arthropod feeding, often located at the interface of two skin surfaces, such as axilla, groin, and inguinal areas (Devine, 2003).

Serial serology (acute and convalescent titers) remains the diagnostic gold standard for rickettsial infections but is not clinically useful for guiding acute treatment. Agents that cause rickettsial diseases are obligate intracellular pathogens and isolation requires cell culture techniques that are not widely performed and can require weeks of incubation for growth, identification, and characterization (Gouriet et al., 2005). Therefore, the intracellular lifestyle of *Rickettsiaceae* results in a narrow diagnostic window that makes detection difficult (Luce-Fedrow et al., 2015), and treatment generally begins before confirming a positive test result. Culture is further complicated by *R. prowazekii’s* designation as a Select Agent and the biocontainment requirements for most rickettsiae and orientiae. While qPCR can be used to confirm rickettsial infections more quickly than serology or culture, sensitivity is low with standard techniques (Angelakis et al., 2012). High-throughput sequencing (HTS) has the potential to aid not only in a more rapid diagnosis of rickettsial diseases but also in predicting antibiotic susceptibility and virulence. However, one of the limitations of using molecular approaches (i.e., quantitative real-time polymerase chain reaction (qPCR), whole genome sequencing) is that this pathogen is generally present in the blood at very low levels and for a relatively short period (Chattopadhyay et al., 2005; Luce-Fedrow et al., 2015; Paris et al., 2015). Applying a targeted enrichment strategy to clinical specimens could lower the limit of detection (LoD) for qPCR or HTS and achieve more rapid and reliable diagnosis of rickettsial diseases.

It has previously been demonstrated that a variety of in-solution hybridization-based enrichment (Gnirke et al., 2009) designs can increase the sensitivity of HTS for the detection of viruses or bacteria in complex sample matrices (Christiansen et al., 2014; Matranga et al., 2014; Bonsall et al., 2015; Briese et al., 2015; Mate et al., 2015; Wylie et al., 2015; Brown et al., 2016; O'Flaherty et al., 2018; Lim et al., 2019; Paskey et al., 2019; Furtwängler et al., 2020; Li et al., 2020; Elliott et al., 2021; Wylezich et al., 2021; Kuchinski et al., 2022) although there can also be challenges in terms of false positive identification of pathogens and the bioinformatic methods involved (Kapel et al., 2023). Hybridization enrichment sequencing has also been demonstrated to enrich the sensitivity of detecting antimicrobial resistance genes in wastewater samples (Baba et al., 2023). Here, we extend the application of this strategy to investigate the possibility that a whole genome, targeted enrichment approach could widen the diagnostic window for *R. prowazekii* and *O. tsutsugamushi* and, unlike previous studies, including an *O. tsutsugamushi* study (Elliott et al., 2021) we apply this approach not just to whole genome sequence-based detection and characterization, but also to qPCR-based detection. Biotinylated 120-mer oligonucleotides were synthesized based on the publicly available *R. prowazekii* and *O. tsutsugamushi* complete genome sequences and used to enrich pathogen genomic DNA present in clinical-type specimens containing known copy number of pathogen genomes. The enriched samples were then subjected to qPCR to detect highly specific pathogen-derived genetic elements, as well as subjected to HTS to detect and characterize pathogen sequences. While targeted enrichment dramatically improved both qPCR and HTS detection of *R. prowazekii* and *O. tsutsugamushi* genomes in spiked, clinical-type specimens, we also demonstrated that the HTS approach enabled both detection and characterization of *O. tsutsugamushi* genomes from real world sepsis patient samples in which only sporadic detection of one or two *Orientia* genes per sample via metagenomic sequencing was previously reported. This underscores the full potential of this technique, which achieved coverage of the entire *Orientia* genome – a more straightforward positive result that would also allow for characterization (e.g., antimicrobial susceptibility).

## 2 Methods

### 2.1 Spiked, contrived clinical samples

A series of contrived samples were created with known concentrations of pathogen DNA. For samples intended for sequencing, human genomic DNA was extracted from 200 μL of whole blood (Reprocell; Beltsville, MD) using the Qiagen DNeasy kit (Qiagen; Valencia, CA) and normalized to 100 ng/sample. Genomic DNA from *O. tsutsugamushi* str. Karp and *R. prowazekii* str. Breinl from the Naval Medical Research Command’s collection (Bishop-Lilly et al., 2013) was isolated using Zymo Quick-DNA Miniprep kit (Zymo Research; Irvine, CA) and spiked into the human gDNA at 0, 12, 25, and 250 genome copies per sample. For samples intended for qPCR testing, plasmid oligonucleotide targets (Eurofins; Louisville, KY) were spiked into the human gDNA at a range from 0–20,000 copies. For the *R. prowazekii* assay, a pET24a vector containing the sequence fragment A of the *ompB* gene (Jiang et al., 2003) was used and for the *O. tsutsugamushi* plasmid assay, a VR1012 vector targeting a 118 base pair (bp) sequence of the 47 kDa antigen gene (*hrtA*) was used (Watt et al., 2005; Jiang et al., 2013).

### 2.2 Clinical samples

Whole human blood was obtained from the Austere environments Consortium for Enhanced Sepsis Outcomes (ACESO) observational sepsis cohort in Cambodia under study NMRC.2013.0019 that has been previously described (Rozo et al., 2020) and approved by Naval Medical Research Command Institutional Review Board. Briefly, adult patients with a suspected infection were enrolled following written informed consent. Enrollment and exclusion criteria have been described previously, but in brief Adult patients (≥18 years) admitted within the last 48 h and who had a suspected infection (as judged by the attending physician) and met at least two of three clinical criteria (thermodysregulation defined as temperature > 38°C or < 36°C, tachypnea defined as Respiratory rate > 20/min and tachycardia defined as heart rate > 90 bpm) were considered for inclusion (Rozo et al., 2020). Multiple biospecimens were collected throughout the patients’ hospitalization and an exhaustive effort was undertaken to identify the infecting pathogens (Rozo et al., 2020). In addition to collecting other study-specific samples, one milliliter of whole blood drawn into a Na-Citrate blood tube was mixed with one milliliter of DNA/RNA Shield (Zymo Research; Irvine, CA). DNA was extracted from whole blood using the Zymo Quick-DNA Miniprep kit (Zymo Research; Irvine, CA). We leveraged our previously published work that was performed to identify infecting pathogens and chose a subset of these samples for inclusion in this current study. The previous data leveraged included serological testing and metagenomic analyses of total RNA isolated from the peripheral blood of sepsis patients, that ultimately identified *O. tsutsugamushi* infection in 16 patients. The samples selected were specifically chosen to assess the clinical utility of our enrichment approach and therefore consisted of three groups. The first group included blood samples in which few *O. tsutsugamushi* sequences were detected by unbiased shotgun sequencing and the samples were IgM+ by serological analysis (n = 8). The second group included samples from two patients negative for *O. tsutsugamushi* by unbiased shotgun sequencing and who were IgM+ serologically. The third group included samples from ten patients who were negative for *O. tsutsugamushi* by unbiased shotgun sequencing and serology, but positive for another pathogen as identified by culture (n = 7) or qPCR (n = 3) (Rozo et al., 2020).

### 2.3 Probe design and synthesis

**Figure 1:**
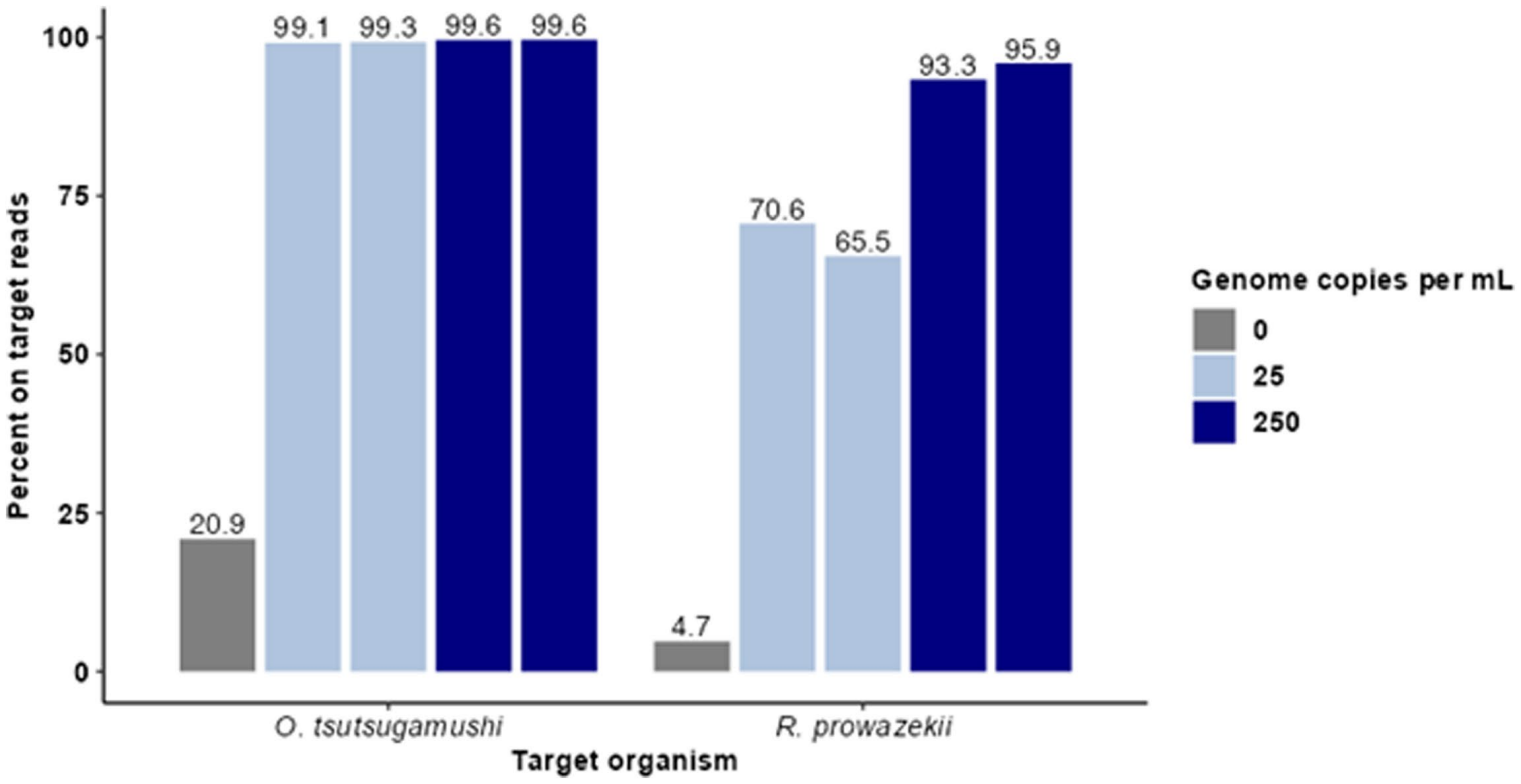
Hybridization-based enrichment of rickettsial sequences from complex samples. Known concentrations of gDNA from *O. tsutsugamushi* and *R. prowazekii* were spiked into complex matrices at 25 and 250 genome copies per mL. Sequence data from enriched samples were mapped to NCBI reference genomes *O. tsutsugamushi* Karp and *R. prowazekii* Breinl. The percent of trimmed reads mapped for each sample is graphed for each replicate.

**Figure 2:**
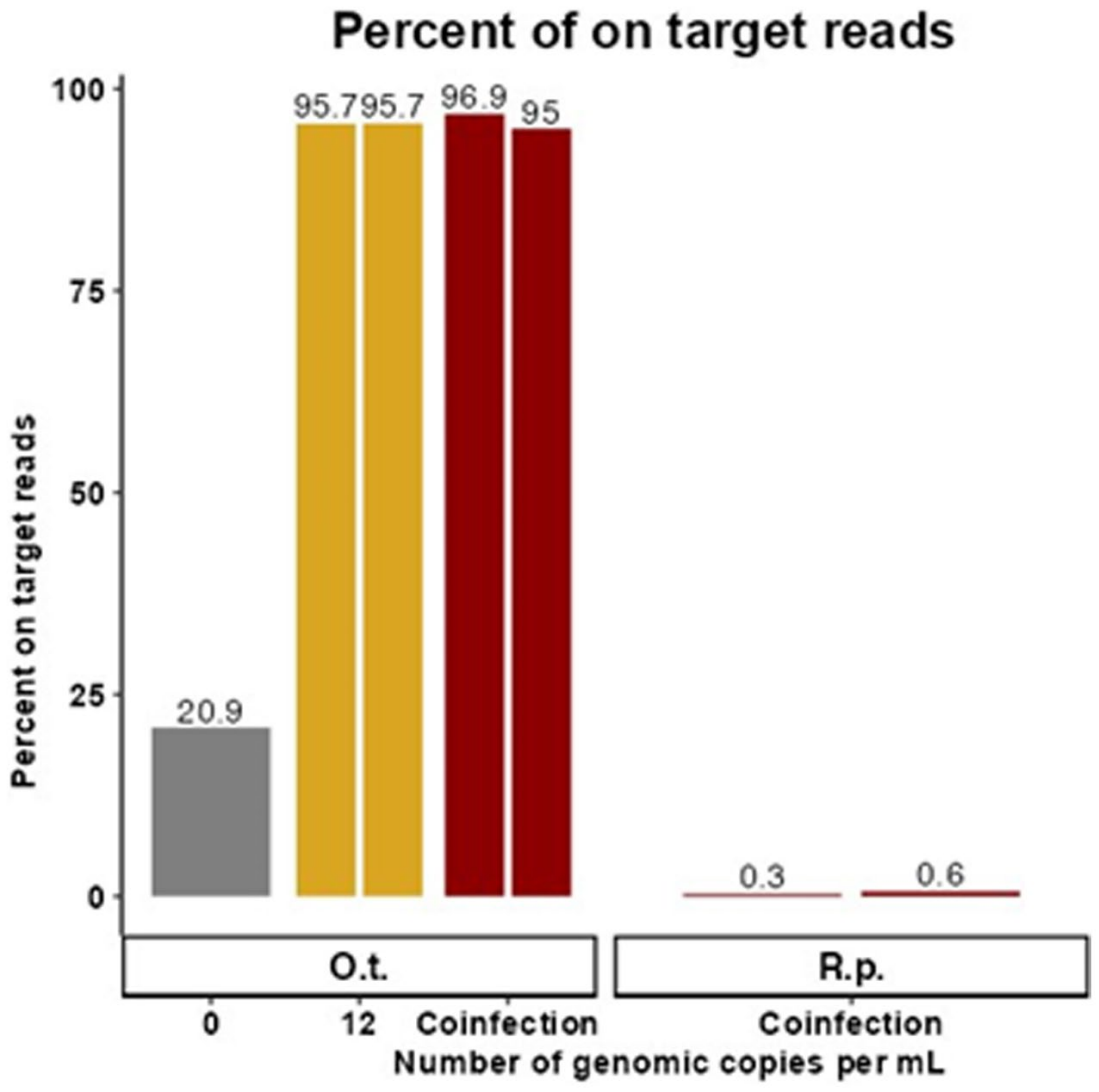
Targeted enrichment sequencing is effective in synthetic coinfection samples. These data represent samples containing target genetic material at the LoD of the existing qPCR assay for *O. tsutsugamushi* in the presence of *R. prowazekii* and human gDNA. The proportion of trimmed reads that were on target for *O. tsutsugamushi* is graphed for each replicate with 0 or 12 genome copies per mL of *O. tsutsugamushi* spiked in. Duplicate contrived coinfection samples also containing *R. prowazekii* genomic DNA are labeled as “Coinfection.” The proportion of *R. prowazekii* reads in the coinfection sample are represented in red bars on the right of the figure. There was no replicate for 0 gc/mL.

The Agilent SureSelect design process was used to generate 120-mer RNA oligonucleotide probes to complement the entire length of each target genome. Custom probes for the Agilent SureSelect XT HS library kit (Agilent Technologies; Santa Clara, CA) were designed using the *R. prowazekii* and *O. tsutsugamushi* genomes listed in Table 1. The genomes were analyzed using a multiple-sequence alignment (MSA) and visualized using Mauve v2.3.1 to determine areas of genome collinearity and SNPs (Figures 1, 2; Supplementary materials) (Darling et al., 2004). To exclude probes with areas of low-complexity (i.e., homopolymer regions or di-nucleotide repeats), the probe set was filtered using DUST v1 (Morgulis et al., 2006). To further reduce redundancy, probes with greater than 95% similarity were excluded. As a final step, the probe set was aligned to the genomes of *Mus musculus* and *Homo sapiens* and complementary sequences were excluded from the probe set in order to prevent off-target binding due to cross-reactivity with host sequences. The resultant enrichment set comprises of 57,000 probes of varying depth along the target genomes (Supplementary Figures S3,S4 demonstrate alignment of probes to target genomes and the Supplementary Table 1 contains probe sequences and coordinates on target genomes used in design).

**Table 1:** Summary of NCBI genome data used in probe design.

Reference genome name	Genome Length (bp)	GenBank accession identifier
*R. prowazekii* Breinl	1,109,301	NC_020993
*R. prowazekii* Madrid E	1,111,523	NC_000963
*R. prowazekii* NMRC Madrid E	1,111,520	NC_020992
*R. prowazekii* BuV67	1,111,445	NC_017056
*R. prowazekii* Chernikova	1,109,804	NC_017049
*R. prowazekii* GvV257	1,111,969	NC_017048
*R. prowazekii* RpGvF24	1,112,101	NC_017057
*R. prowazekii* Dachau	1,109,051	NC_017051
*R. prowazekii* Rp22	1,111,612	NC_017560
*R. prowazekii* Naples	1,111,769	CP014865
*R. prowazekii* Katsinyian	1,111,454	NC_017050
*O. tsutsugamushi* Ikeda	2,008,987	NC_010793
*O. tsutsugamushi* Boryong	2,127,051	NC_009488

### 2.4 Sequencing library preparation

100 ng of gDNA from each sample was sheared to 450 bp fragments using a Covaris Ultra-Focused Sonicator M220 following manufacturer’s protocol (Covaris; Woburn, MA). Sequencing libraries were prepared using the Agilent SureSelect XT HS library kit per manufacturer’s protocol (Agilent Technologies; Santa Clara, CA). Hybridization was performed using the custom Agilent SureSelect probe set described above. Libraries were pooled in sets of 10–16 samples at a concentration of 10–12 pM and sequenced using MiSeq v3 chemistry (Illumina; San Diego, CA) for 600 cycles.

### 2.5 qPCR assays

Quantitative polymerase chain reaction (qPCR) assays targeting *htrA*, the 47 kDa antigen gene of *Orientia tsutsugamushi*, and Rprow, the outer membrane protein B (*ompB*) of *Rickettsia prowazekii*, were performed as previously described using a StepOnePlus Real-Time PCR System (Applied Biosytems; Foster City, CA) to determine copy number (Jiang et al., 2003; Dasch et al., 2004). For *htrA*, each 25 μL reaction contained 1 μL of template DNA or negative control, 0.1 μM of each primer, 0.2 μM of probe, 0.5 μL of ROX Reference Dye (Invitrogen; Waltham, MA), 12.5 μL 2x Platinum Quantitative SuperMix-UDG (Invitrogen; Waltham, MA), and a final concentration of 5 mM MgCl_2_. For Rprow, each 25 μL reaction contained 1 mL of template DNA, 0.2 μM of each primer, 0.2 μM of probe, 0.5 μL of ROX Reference Dye, 12.5 μL 2x Platinum Quantitative SuperMix-UDG, and final concentration of 6 mM of MgCl_2_. A minimum of two replicates was performed per condition, as indicated in results tables.

### 2.6 Bioinformatic analyses

#### 2.6.1 Reference mapping comparison of enriched rickettsial sequences

The resultant sequence reads were trimmed, filtered to remove host and laboratory contaminant reads, then mapped to the available NCBI reference genome closest to the spike-in strain (Karp (Accession LS398548) Breinl (Accession NC_020993) using default parameters requiring a minimum of half the length of the read to map with 80% identity in CLC Genomics Workbench v23 (QIAGEN; CA, United States).; Karp (Accession LS398548) Breinl (Accession NC_020993). Results were visualized using R package ggplot2 (Wickham, 2016; R Core Team, 2022).

#### 2.6.2 Sequence typing

Sequences were quality controlled using bbduk v38.84 (Bushnell, 2014) with Q10 filtering and Q20 trimming, then assembled using SPAdes v3.15.2 (Bankevich et al., 2012). The resultant contigs were submitted to pubMLST (Jolley et al., 2018) specifically for the organism *O. tsutsugamushi*. The resulting allele numbers for each respective gene were used to determine the potential Sequence Type.

#### 2.6.3 Phylogenetic analysis

Type Surface Antigen 56 (*tsa56*) and *sucB* genes were selected for phylogenetic analysis based on the rationale that the *tsa56* gene has historically been used for genotyping *O. tsutsugamushi,* and the *sucB* gene has been used by pubMLST for sequence typing. The full length of *sucB* (1,278 nt) and a 338 nt region of *tsa56* were used for phylogenetic analysis. The nucleotide sequences of these genes were extracted from the SPAdes contigs by first using Bandage v0.8.1 (Wick et al., 2015) and BLAST (Altschul et al., 1990) to identify the contig that contains the genes and then using CLC Genomics Workbench 22 (CLC; QIAGEN; CA, USA) to extract the gene sequence. A short fragment of the 1,590 bp TSA56 was used because the full-length gene was not represented in the sequence data from every sample. For both genes, a multi-sequence alignment was generated using CLC. A maximum likelihood (ML) tree was generated using CLC with GTR + G + T model and 100 bootstrap and the resulting trees were visualized by FigTree v1.4.4 (Rambaut, 2009). The reference Boryong strain was omitted from *sucB* analysis due to a highly divergent sequence that created a long branch with little support.

#### 2.6.4 Antimicrobial resistance analysis

Antimicrobial resistance (AMR) genes were identified using the Bacterial and Viral Bioinformatics Resource Center (BV-BRC) (Olson et al., 2023) database for the eight existing references of *O. tsutsugamushi*: Boryong, Gilliam, Ikeda, Karp, Kato, TA686, UT176, and UT76. Contigs from each sample were screened for AMR genes using tBLASTn in CLC with a minimum threshold of 60% length of the gene.

#### 2.6.5 Clinical feature analysis

Clinical features of 62 patients enrolled in an observational study of sepsis in Cambodia were analyzed. Of these patients, 54 were previously determined to be infected with pathogens other than *O. tsutsugamushi* (Rozo et al., 2020). Fisher’s exact tests were used on contingency tables to determine *p*-values. For clinical parameters, a Mann–Whitney U test was used to determine p-values. All statistical calculations were performed using GraphPad Prism 8.3.1 (GraphPad Software Inc.; San Diego, CA, United States).

## 3 Results

### 3.1 Targeted enrichment recovers rickettsial genomes from contrived clinical samples with high specificity

**Figure 3:**
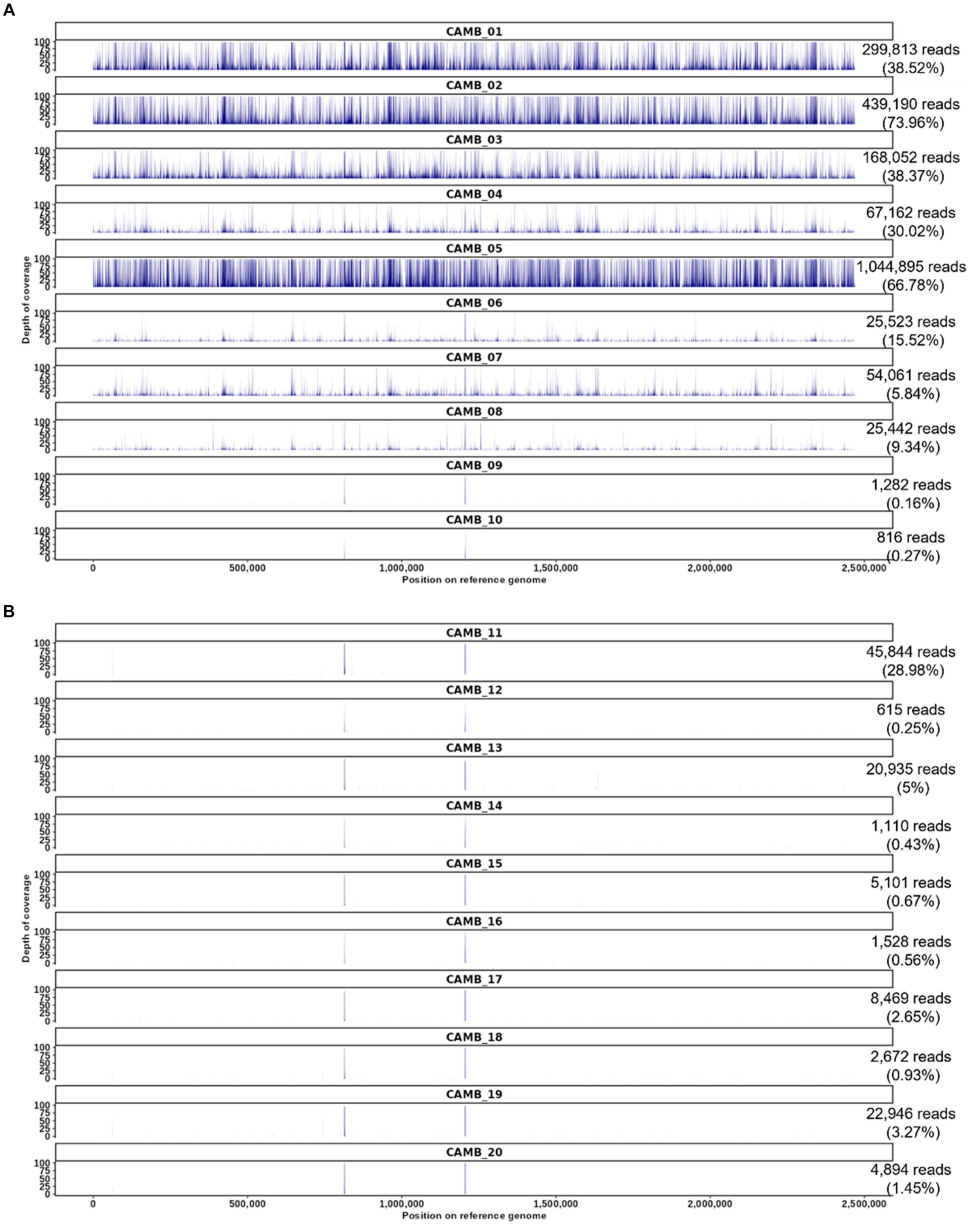
*O. tsutsugamushi* targeted enrichment applied to whole blood patient samples derived from an observational study of sepsis in Cambodia. **(A)** Patients who were positive by serology and weakly positive for *O. tsutsugamushi* sequences by unbiased shotgun sequencing and patient samples who were serology positive but whose samples did not contain *O. tsutsugamushi* sequences according to unbiased shotgun sequencing results (CAMB_09, CAMB_10). **(B)** Patient samples that were serology negative and unbiased shotgun sequencing negative for *O. tsutsugamushi* sequences, but positive for other pathogens by culture or qPCR in the prior study (Rozo et al., 2020). The number of reads mapped for each sample is shown on the right with the proportion of trimmed reads mapped in parentheses. The reference used in this read mapping is *O. tsutsugamushi* isolate Karp genome, accession LS398548, length 2,469,803 nucleotides.

For this proof of concept in detecting rickettsial pathogens using enrichment, we first sought to understand the limit of detection (LoD), specificity, cross-reactivity, depth, and breadth of targeted enrichment with rickettsial genomes using *O. tsutsugamushi* and *R. prowazekii* as representative species. To do so, contrived clinical samples were prepared with the pathogen genomic DNA spiked in at clinically relevant titers (measured in genomic copies (gc)/mL). Human gDNA was first extracted from 200 μL of whole blood and normalized to 100 ng/sample. Genomic DNA from *O. tsutsugamushi* and *R. prowazekii* was spiked in at 25 and 250 genome copies per mL. Agilent SureSelect probes targeting the *O. tsutsugamushi* and *R. prowazekii* genomes were used to enrich the contrived samples in duplicate, and enriched samples were sequenced using an Illumina MiSeq. Targeted enrichment performed well for each organism tested, recovering 85–88% of the *O. tsutsugamushi* genome and 88–91% of the *R. prowazekii* genome. Enriched samples were found to have >65% on-target reads (Figure 1). The average read coverage depth was 249x for *O. tsutsugamushi* and 165x for *R. prowazekii*; a depth of coverage more than adequate to perform strain level identification and SNP analysis in most cases. Minimal cross-reactivity producing two short regions of non-specific binding by human sequences was observed (visible in clinical results presented later, see Figure 3).

Due to overlapping areas of endemicity and shared vectors, rickettsial co-infection has been observed although it is rare, and it poses a potential challenge for molecular diagnostics (Nogueras et al., 2015; Abreu et al., 2019; Kim et al., 2019). Therefore, replicate samples containing target genetic material at 12 gc/mL, the LoD of the existing qPCR assay for *O. tsutsugamushi* in the presence of *R. prowazekii,* were created in a background of human gDNA to simulate co-infected samples (and a control sample containing 0 gc/mL of *O. tsutsugamushi* gDNA was also included). Cleaned reads were mapped to the available NCBI reference genome closest to the spike-in strain (Karp or Breinl), and the proportion of on target reads calculated (Figure 2). For each replicate, 10,922 (0.3%) and 5,745 (0.6%) reads specific to *R. prowazekii* were enriched in the coinfected sample, compared to 3,766,741 (96.9%) and 918,840 (95%) reads specific to *O. tsutsugamushi.* Although we observed preferential enrichment for *O. tsutsugamushi* in the background of a low titer rickettsial coinfection, there was still a sufficient breadth of reads covering both genomes to provide evidence to determine the species and possibly proceed to further analyses (Supplementary Figure S1). The higher efficiency of enrichment by the *O. tsutsugamushi* probes as compared to the *R. prowazekii* probes in this 12-genome copy per pathogen per mL coinfection scenario was consistent with what we observed in singularly spiked, monoinfection samples at 25 gc/mL, whereas at higher titers, the probes perform similarly (Figure 1; Supplementary Figure S1). The presence of *R. prowazekii* genomic DNA did not impact the recovery of *O. tsutsugamushi* DNA, as similar proportions of “on target” (e.g., *O. tsutsugamushi*-specific) reads were recovered from samples with and without *R. prowazekii* spiked in.

### 3.2 Targeted enrichment for *O. tsutsugamushi* generates robust genome sequences with high specificity from clinical specimens from sepsis patients

We previously utilized unbiased shotgun sequencing to identify *O. tsutsugamushi* sequences directly from clinical specimens obtained from an observational study of sepsis in Cambodia as described in the methods section and elsewhere (Rozo et al., 2020), but in that prior work the genome sequences produced by shotgun sequencing were partial sequences. Therefore we resequenced those same samples here and found that our targeted enrichment sequencing assay produced whole genome sequence data from all eight samples that were previously known to be positive for *O. tsutsugamushi* (labeled CAMB_01 through _08 in Figure 3) (Rozo et al., 2020). While the previous results were weakly positive for *O. tsutsugamushi* from shotgun sequencing data, with reads mapping only to one or two genes (typically rRNA genes), targeted enrichment sequencing resulted in reads covering the entire *O. tsutsugamushi* genome (Figure 3A). Two samples that were previously IgM positive for *O. tsutsugamushi* but negative for *O. tsutsugamushi* by unbiased shotgun sequencing, CAMB_09 and _10, remained negative for *O. tsutsugamushi* using the targeted enrichment sequencing assay (Figure 3A), recapitulating the previously published findings. The culture and sequence-negative samples were still negative using the hybridization enrichment assay (Figure 3B), suggesting they may have had a titer below the LoD for the assay or the infection may already have been cleared from peripheral blood in these patients by the time these blood samples were drawn. Minimal cross-reactivity producing two short regions of non-specific binding by human sequences was observed (Figure 3). Finally, all samples that were positive for another pathogen as determined by culture or qPCR were also negative for *O. tsutsugamushi* sequences using targeted enrichment (Figure 3B). These results demonstrate that targeted enrichment provides increased specificity and sensitivity for detection and genetic characterization from real world clinical samples.

The depth and breadth of coverage obtained from *O. tsutsugamushi* positive clinical samples was considerably higher than was reported using shotgun metagenomic analysis of transcriptomic data from the same patients (Rozo et al., 2020). Therefore, we leveraged our newly enhanced dataset to analyze the characteristics of these pathogens circulating in Cambodia. Taxonomic classification and analysis of antibiotic resistance markers was performed as described in the methods.

**Figure 4:**
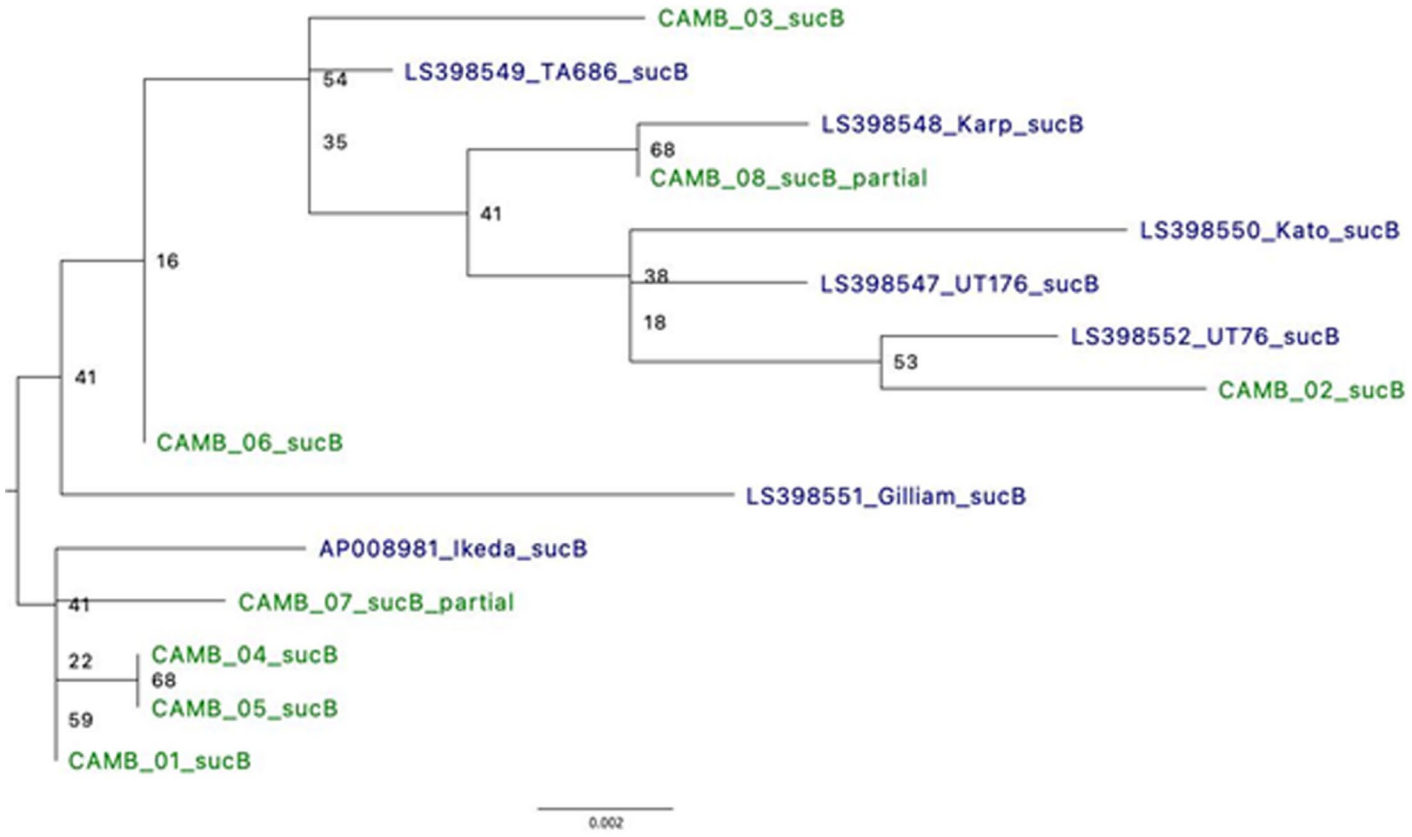
Phylogeny using full length *sucB* sequences demonstrates relatedness to known *O. tsutsugamushi* strains. Where possible, alignments of full *sucB* sequences were used to generate a Maximum Likelihood tree. There were two samples for which only a partial *sucB* gene sequence was available for analysis. *UT76 and UT176 are clinical isolates similar to the Karp strain (Paris et al., 2009).

Type Surface Antigen 56 (TSA56), derived from the 56-kDa gene (*tsa56*), has been the standard for typing *O. tsutsugamushi*. Similarly, dihydrolipoamide acetyltransferase component or Dihydrolipoyllysine-residue succinyltransferase component of 2-oxoglutarate dehydrogenase complex (*sucB*) is a gene used in multi-locus sequence typing (MLST) for *O. tsutsugamushi* (Jolley et al., 2018). Based on analysis of a 338 bp fragment of the gene *tsa56*, four samples (CAMB_01, CAMB_02, CAMB_04, and CAMB_05) were more closely related to the Kato strain whereas two samples (CAMB_03 and CAMB_06) were more closely related to Karp, UT76, and UT176 strains (Supplementary Figure S2). The *tsa56* gene was not present in the assemblies for samples CAMB_07 and CAMB_08 and therefore those two samples could not be included in the analysis. Phylogenetic analysis of *sucB* revealed that the samples grouped with various representative sequences from the public database: CAMB_02 was most closely related to UT76 strain; sample CAMB_08 grouped with the Karp strain; samples CAMB_01, CAMB_4, CAMB_05, and CAMB_07 grouped with the Ikeda strain; and samples CAMB_03 and CAMB_06 were more closely related to TA686 and Gilliam strains (Figure 4). Note that samples CAMB_08 and CAMB_07 are only partial sequences and their placement is not well supported.

In addition to conducting phylogenetic analyses, we also screened the dataset for 20 common antimicrobial resistance genes as described in the methods (Table 2). Of note, two genes that confer resistance to rifampicin (*rpoB* and *rpoC*), a treatment option for *O. tsutsugamushi*, were universally absent from each of our clinical isolates (Table 2) (Eremeeva and Dasch, 2002; el Sayed et al., 2018). These results demonstrate the clinical utility of HTS following targeted enrichment for the identification and characterization of *O. tsutsugamushi* directly from clinical specimens.

**Table 2:** Genes that confer antimicrobial resistance (AMR) present in *O. tsutsugamushi* positive clinical specimens.

	*ddl*	*fabF*	*fabI*	*fusA*	*gidB*	*gyrA*	*gyrB*	*ileS*	*murA*	*pgsA*	*rho*	*rplF*	rpoB*	*rpoC*	*rpsJ*	*rpsL*	*strB*	tuf*	10ufA|tufB*	EC -2.1.1.170
O.t. – Boryong	X	X	X	X	X	X	X	X	X	X	X	X	X	X	X	X		X		
O.t. – Gilliam	X	X	X	X		X	X	X	X	X	X	X	X		X	X			X	X
O.t. – Ikeda	X	X	X	X	X	X	X	X	X	X	X	X	X	X	X	X		X		
O.t. – Karp	X	X	X	X		X	X	X	X	X	X	X	X		X	X			X	X
O.t. – Kato	X	X	X	X		X	X	X	X	X	X	X	X		X	X	X		X	X
O.t. – TA686	X	X	X	X		X	X	X	X	X	X	X	X		X	X	X		X	X
O.t. – UT176	X	X	X	X		X	X	X	X	X	X	X	X		X	X			X	X
O.t. – UT76	X	X	X	X		X	X	X	X	X	X	X	X		X	X			X	X
90%																				
CAMB-06	X		X			X			X						X	X		X	X	
CAMB-01	X	X	X	X		X		X	X	X	X				X	X		X	X	
CAMB-07									X						X	X		X	X	
CAMB-02	X	X	X	X		X	X	X	X	X	X	X			X	X		X	X	
CAMB-03	X		X						X		X	X			X	X		X	X	
CAMB-04															X	X		X	X	
CAMB-08									X							X		X	X	
CAMB-05	X	X	X		X	X	X		X		X	X			X	X		X	X	X
60%																				
CAMB-06	X	X	X		X	X	X	X	X	X	X				X	X		X	X	X
CAMB-01	X	X	X	X	X	X	X	X	X	X	X	X			X	X		X	X	X
CAMB-07		X							X	X					X	X		X	X	
CAMB-02	X	X	X	X	X	X	X	X	X	X	X	X			X	X		X	X	X
CAMB-03	X	X	X			X	X	X	X		X	X			X	X		X	X	
CAMB-04		X							X	X	X				X	X		X	X	
CAMB-08		X							X						X	X		X	X	
CAMB-05	X	X	X		X	X	X	X	X	X	X	X			X	X		X	X	X

### 3.3 Targeted enrichment improves qPCR performance

HTS may not be available or appropriate in every case and qPCR remains a viable alternative for molecular diagnostics. One limitation to qPCR, as with HTS, is that the number of genomic copies in blood samples is extremely low in rickettsial infections. In fact, one study found the median titer was the equivalent of 13 copies per mL of blood, but as low as zero in many cases (Sonthayanon et al., 2009). To assess if targeted enrichment could increase the performance of qPCR for the detection of rickettsial genomes, we first sought to determine the limits of detection (LoD) of qPCR for *R. prowazekii* Breinl in contrived clinical samples both with and without targeted enrichment. Dilutions of either plasmid DNA containing fragment A of the *ompB* gene, or *R. prowazekii* Breinl genomic DNA were prepared ranging from 20,000 copies to ~1.5 copies, as described in the methods. A qPCR assay targeting the *ompB* gene of *R. prowazekii* was performed in duplicate as published by Jiang et al. (2003) and Dasch et al. (2004). While one replicate of qPCR detected ~6 copies of *R. prowazekii* Breinl genomic DNA, the lower LoD without targeted enrichment was ~12 copies (Table 3). We therefore selected 12.5, 25 and 50 genome copies for further evaluation. To confirm the baseline LoD, 20 replicates of each of the three target copy numbers were assayed using qPCR without prior enrichment. Without enrichment, the 12.5 genome copies per mL set was only detected in 17 of 20 replicates (85%), however, the target was reliably detected at both 25 and 50 copies per mL for all 20 replicates. Therefore, we determined that the LoD was 25 genome copies per mL for this qPCR assay (Table 4).

**Table 3:** qPCR of standard curve for determination of LoD (without enrichment).

Copy#	Ct Value (Plasmid DNA)		Ct Value (Genomic DNA)	
20,000	25.63	25.58	26.31	26.00
4,000	27.18	27.19	28.47	28.68
800	28.82	28.89	30.96	30.91
400	29.41	29.51	32.00	31.63
200	30.56	30.54	32.91	33.19
100	31.15	31.19	33.27	33.15
50	32.72	32.59	34.17	35.64
25	33.91	32.79	36.46	36.54
12.5	34.85	34.81	36.92	36.90
6.25	35.21	35.74	36.21	–
3.13	36.61	36.96	–	–
1.56	39.66	36.78	–	–
0	–	–	–	–

**Table 4:** Confirmation of LoD based on 20 replicates for each copy number (without enrichment).

Copy#	Positive	Negative
0	–	–
50	20	0
25	20	0
12.5	17	3

Once the LoD for unenriched qPCR was established, we assessed if targeted enrichment coupled with the same qPCR assay could reduce the LoD by detecting even fewer genomic equivalents. To accomplish this, enriched sequencing libraries with starting material ranging from approximately 1.5 to 50 genome copies were produced in triplicate for each copy number and qPCR was performed in duplicate on an aliquot from each library, resulting in six replicates for each copy number being tested. Post-enrichment qPCR data allowed for positive detection down to one copy across six replicates, indicating the success of applying enrichment to increase sensitivity of the qPCR assay (Table 5). The Ct values for the 15 sequencing libraries after enrichment strategies (Supplementary Table S2) were consistent with the Ct values equivalent to greater than 4,000 genome copies (Table 3), an observation reinforced by copy number determination of the enriched sequence libraries (Supplementary Table S2). Taken together, these data demonstrate that genome-enriched samples performed better in qPCR assays than non-enriched samples.

**Table 5:** Comparison of qPCR data generated before and after enrichment.

Copy#	Before enrichment (2 replicates)		Copy#	After enrichment (6 replicates)*					
50	+	+		ND	ND	ND	ND	ND	ND
25	+	+	30	+	+	+	+	+	+
12.5	+	+	10	+	+	+	+	+	+
6.25	+	−		ND	ND	ND	ND	ND	ND
3.13	−	−	3	+	+	+	+	+	+
1.56	−	−	1	+	+	+	+	+	+
0	−	−	0	−	−	−	−	−	−

^*^Enriched sequencing libraries were produced in triplicate for each copy number and qPCR on each library was performed in duplicate, resulting in 6 replicates for each copy number.

## 4 Discussion

For both bacterial and viral pathogens, pathogen genomic sequencing is used to trace outbreaks routinely. For instance, a recent fatal outbreak of *Burkholderia pseudomallei* within the United States among patients with no travel history was traced to aroma therapy products using genomics. These four cases generated clinical isolates that were sequenced (Gee et al., 2022). However, in some cases, there are constraints that prevent clinical isolates from being grown from the primary patient samples (in particular when there are many patients or the organism is hard to culture). The genomic enrichment method would be valuable in cases where no clinical isolate is available and pathogen genetic variations are being used to perform molecular epidemiology. For instance, other examples of pathogen genomic sequencing that have been conducted in our own laboratory for similar use cases include that of SARS-CoV-2 (Cer et al., 2022; Lizewski et al., 2022) as well as methicillin-resistant *Staphyloccus aureus* [MRSA: (Millar et al., 2017, 2019)]. In addition to our own use of whole genome sequencing to trace transmission chains of SARS-CoV-2 in military settings, others have used whole genome sequencing to trace transmission chains in the hospital setting (Keehner et al., 2024) and the same has been done for Dengue virus in household transmission settings (Berry et al., 2021). These are just a few examples out of many that clearly demonstrate outbreaks for a variety of pathogens, both viral and bacterial, can be traced to identify source and transmission chains by use of whole genome sequencing data. Therefore, it is quite possible that in the future similar methods could be applied if there were an outbreak of a rickettsial organism.

A limitation of using unbiased HTS of metagenomic samples is that it suffers from a lack of sensitivity for pathogens in complex sample types that contain both host and commensal sequences in addition to the pathogen of interest (i.e., human clinical samples or environmental samples). Other complicating factors that affect the ability to detect an organism of interest include the organism’s genome size compared to other organisms present in the matrix (particularly for viral genomes), sample complexity (the total range of organisms present within the sample matrix), and titer. These factors influence the contribution of organism-specific versus host- and/or commensal-specific reads, and hence the LoD. We evaluated probe-based targeted enrichment as a strategy to augment both sequencing and molecular diagnostics (e.g., qPCR) for the detection of the intracellular pathogens *R. prowazekii* and *O. tsutsugamushi*, which generally have narrow diagnostic windows. This method has been applied previously for sequencing of some microbes in clinical samples (for example *C. trachomatis* in (Christiansen et al., 2014) and *O. tsutsugamushi* in Elliott et al., 2021), but to our knowledge not for sequencing Rickettsia spp. specifically, and only in very scarcely few examples in the literature for PCR assays of any pathogens until now (Yang et al., 2016; Bai et al., 2019), and not for PCR for rickettsial pathogens. Using this approach, we report improved sensitivity over traditional diagnostic methods – LoD as low as one genomic copy (Table 5) was detected using qPCR post enrichment, as compared to 25 genome copies without enrichment. We have also expanded the sensitivity of whole genome sequencing data that can be generated from clinical specimens, covering nearly the entire genome of the targeted organism as compared to the detection of only one or two pathogen genes without enrichment. Taken together, we demonstrated that this strategy allows for both identification and characterization of rickettsial diseases directly from clinical specimens, dramatically increasing the potential applications of this method. As compared to traditional diagnostic methods (i.e., culture-based assays, serology, qPCR), a HTS-based detection approach in general provides more information than other diagnostic assays through its ability to garner information beyond identification of the agent to include strain level typing and presence/absence of antimicrobial resistance genes and/or virulence factors.

Our data demonstrate that genome enrichment greatly enhances the sensitivity of both qPCR and sequencing for direct detection of pathogen nucleic acids. The previous sequence data we obtained for these clinical samples via unbiased shotgun (e.g., metagenomic) sequencing and reported in Rozo et al. (2020) consisted of sporadic detection of one or two *Orientia* genes per sample (typically rRNA genes), whereas now with the genome enrichment sequencing strategy reported in the current study, we have achieved coverage of the entire *Orientia* genome from these same samples (Figure 3). Obtaining from a patient sample a few partial gene sequences with homology to the pathogen of interest’s genome at low depth of coverage and within the milieu of reads deriving from commensal organisms and background might be considered an equivocal result as compared to obtaining an entire pathogen genome from a patient sample, which would be much more straightforward and would also allow for characterization (e.g., antimicrobial susceptibility). In other words, though both unbiased metagenomic sequencing and enrichment sequencing “detected” the pathogen, enrichment sequencing data are much more conclusive and leave much less room for doubt. In addition to demonstrating the increased sensitivity when genome enrichment is applied to sequencing-based detection, we also demonstrated in this study that genome enrichment greatly increased the sensitivity of detection by qPCR – reducing the limit of detection down from 25 genome copies per mL without enrichment to 1 genome copy per mL with enrichment. Therefore, we have demonstrated a much more sensitive direct detection assay.

We proved the clinical utility of this method by enriching patient blood samples obtained from an observational study of sepsis in Cambodia (Rozo et al., 2020). While rickettsial infections respond to early treatment with antibiotics, rickettsial infections are challenging to identify clinically. Eschars, while associated with a number of rickettsial infections, are often absent or overlooked and clinical laboratory features are nonspecific (Abdad et al., 2018). Multiple features consistent with a diagnosis of scrub typhus were observed among the eight patients from whom *O. tsutsugamushi* genomes were detected (Supplementary Tables S3,S4). Specifically, symptoms of fever and shortness of breath were uniformly present. Notable laboratory abnormalities that were present in most but not all *O. tsutsugamushi* positive patients included liver function test elevation and thrombocytopenia. These features are supportive of the diagnosis of scrub typhus and consistent with what has been reported in prior literature (Sriwongpan et al., 2013). Epidemiologic clues including the occupation of farming were also present (Rozo et al., 2020) but could be of limited clinical utility in predominantly agrarian societies where most patients may have risk factors for mite exposure. For an infection that is treated based on clinical suspicion, the lack of pathognomonic features escalates the need for improved clinical diagnostics – a gap that could be filled via the enrichment-augmented assays described in this study.

Clinical experience and animal models demonstrate that the diagnostic window for detection of *O. tsutsugamushi* from blood by qPCR is short lived, and the initial day for detection is dose related (Jiang et al., 2003; Chattopadhyay et al., 2005; Watt et al., 2005; Paris et al., 2015). While targeted enrichment increases the sensitivity in both contrived and clinical specimens, we were unable to draw definite conclusions regarding diagnostic window. Two of the septic patient samples we attempted to characterize using targeted enrichment were IgM positive at the time of sample collection. However, targeted enrichment failed to produce meaningful coverage of the *O. tsutsugamushi* genome. IgM antibodies can be nonspecific and could represent false positives due to cross-reactivity or could be derived from a previous infection, since their longevity can last up to a year from infection. A sufficiently powered study utilizing well-characterized *O. tsutsugamushi* patient samples will be necessary to draw specific conclusions with regard to diagnostic window. Taking into consideration the reduction of sensitivity down to a single genome copy per sample, we hypothesize that an expanded diagnostic window would result.

In addition to the identification of *O. tsutsugamushi* in patient samples, hybridization enrichment allowed us to detect antibiotic resistance markers and to perform MLST, neither of which was possible in the previous analysis of these same samples that used metagenomic sequencing without enrichment (Rozo et al., 2020). While sequence types were identified for four of the eight clinical specimens, there were not enough data to determine the sequence types for the other four because not all loci were found in contigs. We expected this result due to the variety of confounding features that could limit the completeness of the genomes including unknown duration of infection and previous antimicrobial therapy. We generated Maximum Likelihood trees using the standard *O. tsutsugamushi* classification regions t*sa56* and *sucB* (Figure 4) and *tsa56* (Supplementary Figure S2). In a congregate setting, such as many military settings like ships and recruit training centers, pathogen strain relatedness can be used to assess if there was one or multiple introductions of a pathogen into the congregate setting, and that can inform force health protection decision-making about what infection control measures should or should not be put in place to prevent further spread. This can apply to other congregate settings as well, such as hospitals and school dormitories. Also, if antimicrobial resistance markers are detected that would render a particular treatment ineffective that particular therapy could be avoided reducing treatment delay and improving clinical outcomes.

In the literature, resistance to tetracycline has been hypothesized as a mechanism underlying delays in clinical improvement for almost 30 years. It is not currently known what the true prevalence of antibiotic resistance is in *O. tsutsugamushi*, but it remains as a formal possibility [reviewed in (Lu et al., 2021)]. Thus, we also verified the presence of traditional *O. tsutsugamushi* antimicrobial resistance markers. Incorporating this approach with traditional diagnostic and treatment algorithms could both reduce diagnostic delay and lessen the need for empiric antimicrobial therapy. Given that the diagnostic window of *R. prowazekii* and *O. tsutsugamushi* by currently available assays is rather narrow and so treatment is often based on suspicion rather than positive tests, development of a diagnostic tool that could simultaneously provide a positive species- or strain-level identification simultaneously with information as to antimicrobial sensitivity could be expected to result in more timely and accurate diagnoses. Treatment generally begins before confirming a positive test result via culture, which could take weeks. The time estimation for sequencing via this method, not including DNA extraction, is 11 h: 8 h of library preparation, 1.5 h of quality control, and 1.5 h to load the sequencer. The sequencer would then run for 1–3 days (end-user can decide how long) and then a quick read mapping-based analysis could be completed within an hour. The result could be that more patients get treated adequately with the correct antimicrobials, with less delay. As such, we posit that this assay could allow for a novel sequence finding that would predict functional resistance faster than clinical treatment failure, and that there is an intrinsic benefit to developing assays that help avoid clinical treatment failure.

We have provided evidence that this technique is effective for the detection, identification, and characterization of *R. prowazekii* and *O. tsutsugamushi* in clinical-type samples and we are currently exploring some of the many potential applications for expansion of this enrichment technique. It is likely that these probes could be used to enhance sensitivity for detection of related organisms from almost any complex sample type. For instance, we performed sequence analyses that indicate these probes would work similarly for detection of the other typhus group rickettsia, *Rickettsia typhi,* which has a significant similarity at the nucleotide level (Supplementary Table S5) and overall genome synteny (Supplementary Figure S3). Minor modifications to the 120-mer probes could produce a pan-rickettsial enrichment panel. Given that the probes work in co-infection scenarios, it would be most economical to expand to a pan-rickettsial test that can detect multiple possible etiological agents. Additionally, deeper multiplexing can be used to decrease cost. Beyond rickettsial applications, probes for targeted enrichment can be designed against any relevant pathogen, resulting in combinations of relevant probes that could produce regionally specific panels, biothreat panels, and environmental panels to increase the LoD for pathogens of interest. Commercial vendors provide both freely-available tools to design custom probes, as well as make probe design available as part of ordering options. Enriched products can be characterized by qPCR simply for pathogen identification or via HTS for in-depth genetic characterization. The benefit of generating enough sequence data for genetic characterization, as demonstrated by our data, is that genome enrichment greatly enhances the ability to further characterize a clinical sample that is outside of the standard diagnostic window. We conclude this work demonstrates the following proof of concept: This method increased the sensitivity of both qPCR and sequencing for direct detection of pathogen nucleic acids that not only allowed for the species level identification but also virulence genes and AMR markers. Specifically, we demonstrated that this method enables the detection and characterization of *O. tsutsugamushi* genomes directly from sepsis patient samples.

## Data availability statement

The datasets presented in this study can be found in online repositories. The names of the repository/repositories and accession number(s) can be found at: https://www.ncbi.nlm.nih.gov/, PRJNA902390.

## Ethics statement

The studies involving humans were approved by Naval Medical Research Command Institutional Review Board. The studies were conducted in accordance with the local legislation and institutional requirements. The participants provided their written informed consent to participate in this study.

## Author contributions

AP: Formal analysis, Investigation, Writing – original draft, Writing – review & editing. KS: Investigation, Writing – original draft, Writing – review & editing. LV: Investigation, Writing – review & editing. CA: Formal analysis, Writing – review & editing. RC: Investigation, Supervision, Writing – original draft, Writing – review & editing. KF: Investigation, Writing – review & editing. PB: Investigation, Writing – review & editing. DC: Investigation, Writing – review & editing. HG: Investigation, Writing – review & editing. AR: Investigation, Writing – review & editing. CF: Investigation, Writing – review & editing. KB-L: Conceptualization, Formal analysis, Funding acquisition, Investigation, Supervision, Writing – original draft, Writing – review & editing.

## Author disclaimer

The views and conclusions contained in this document are those of the authors and should not be interpreted as necessarily representing the official policies, either expressed or implied, of the U.S. Department of Health and Human Services, the U.S. Department of Defense, the U.S. Navy, the U.S. government or of the institutions and companies affiliated with the authors. Several of the authors are U.S. government employees. This work was prepared as part of their official duties. Title 17 U.S.C. § 105 states, “Copyright protection under this title is not available for any work of the United States Government.” Title 17 U.S.C. §101 defines a U.S. Government work as a work prepared by a military service member or employee of the U.S. Government as part of that person’s official duties.
