# Supplementary material for: A proof of concept for a targeted enrichment approach to the simultaneous detection and characterization of rickettsial pathogens from clinical specimens
Source: Front Microbiol. 2024 Apr 10;15:1387208. doi: 10.3389/fmicb.2024.1387208 (PMC11039911; doi:10.3389/fmicb.2024.1387208)
Supplement: Supplementary file 1 [file Table_1.docx]

Supplementary Material

**Supplementary Table S1. Probe sequences with coordinates on genomes used in design**

TargetID ProbeID Sequence Start Stop

NC_009488_1_Orientia_tsutsugamushi_Boryong_complete_genome probe_cstm_Rprow_021218_1x_1 TAACTGATCTTAATAAATGTAATGAATACACTTCTGCAACAGCCAAATACTACTAAAGATGAGTTATACTTTCATGAGTTATGGAGTAGCGTCAAGCGAGATTTAAGGCATCATTATGGT 0 120

NC_009488_1_Orientia_tsutsugamushi_Boryong_complete_genome probe_cstm_Rprow_021218_1x_10 TATGAAATTACTATTGAAAATACTCAAGAAATTTTAAGAGATTTGCTACGATCTAACCAGAAAACAATTACCATTGATAGCATTCaaaaaaaaGTAGCTGAGCGTTATAATATTAAGATA 1080 1200

NC_009488_1_Orientia_tsutsugamushi_Boryong_complete_genome probe_cstm_Rprow_021218_1x_100 TCTAATTGGCAACAAGAATGGCTAAAAAGTAATTATGATAAATTGCAAAAAATAAACCAGTGTATTTTGACGCAAGaaaaaaaaaTAGTTGAATTTAATGCTGCAGATCGTATTATAGAA 11880 12000

NC_009488_1_Orientia_tsutsugamushi_Boryong_complete_genome probe_cstm_Rprow_021218_1x_1000 TTAAGACCATCAACTTTCCTCAAAATATCCACCATCTTTGAAAATGTTACCTTCCTTACTCCTGTTAATCGACGAAATTTTTCATCCTTTAACTCTTTAATCTTATCGAATTTCGTTATT 119880 120000

NC_009488_1_Orientia_tsutsugamushi_Boryong_complete_genome probe_cstm_Rprow_021218_1x_10000 CGAATTAGAATGTGTTCTAATAATGCAGTTTATACAGTTGAGAATGTTGGCtttttttCTCCTAAAAAACAAATTTCAGGTGTACTATATACTGGTGAAATAGGGTTTGTTACTGCAGCA 1199880 1200000

NC_000963_1_Rickettsia_prowazekii_str__Madrid_E_chromosome_complete_genome probe_cstm_Rprow_021218_1x_100009 TTTTTTGTTTAGCATTTTCTAATCTTGCTTGAGCATCTAGTATTTCCTTAGAAATTAGTGCACTAGATTCTGAAAATTCGTAtttattttcttttttttgatttactttctctaaagttc 90600 90720

NC_009488_1_Orientia_tsutsugamushi_Boryong_complete_genome probe_cstm_Rprow_021218_1x_10001 ATTAAACAGGTTGCTGATTGCAGAGTTGGTGATACAATAACTGATGATAAAAAGCCATGCGCTCAAATATTACCAGGGTTTAAACCTAATTTGCCAGTTGTTTTTTGTGGATTATACCCT 1200000 1200120

NC_009488_1_Orientia_tsutsugamushi_Boryong_complete_genome probe_cstm_Rprow_021218_1x_10002 TCTGATGCTTCACAATTTAACCATTTAAAAGATTCCTTGCAAAAATTAAGATTAAATGACGCTAGCTTTGAATTTGAACAGGAAAGCTCTGGCGCTCTTGGATTTGGATTTCGTTGCGGC 1200120 1200240

NC_009488_1_Orientia_tsutsugamushi_Boryong_complete_genome probe_cstm_Rprow_021218_1x_10003 TTTTTAGGATTGCTACATCTAGAAATTATTCAAGAACGTTTAGAGCGTGAATTTGATCTTGATATGATTACTACTGCACCAAGTGTTATGTATAAAGTATACTTAAATACTGGAAAAGTG 1200240 1200360

NC_009488_1_Orientia_tsutsugamushi_Boryong_complete_genome probe_cstm_Rprow_021218_1x_10004 ATTGATGTGCATAATCCAGCAGATTTACCTGAAATGCAAAAGATTAAAAGCATGAGTGAGCCATGGGTTGAAGCAACTATTTTTATACCTGATCAATATTTGGGAGCTATTCTTGGTTTA 1200360 1200480

NC_009488_1_Orientia_tsutsugamushi_Boryong_complete_genome probe_cstm_Rprow_021218_1x_10005 TGCACTGAAAAACGTGGAGTGCAAGTAGATCTAACTTATGTAAATGGCAGAGCTAAACTAGTATATCTGTTGCCTCTAAATGAAATAGTATTTGATTTTTATGACAAACTAAAATCTTAC 1200480 1200600

NC_009488_1_Orientia_tsutsugamushi_Boryong_complete_genome probe_cstm_Rprow_021218_1x_10006 TCTAAGGGATATGCAAGTTTTGATTGGCAGATAGATAGCTATAAACAAAGCGACTTAATCAAGCTTAGTATATTAGTTAATGGAAATCCTGTTGATGCTTTATCAACAATTGTACACCGT 1200600 1200720

NC_009488_1_Orientia_tsutsugamushi_Boryong_complete_genome probe_cstm_Rprow_021218_1x_10007 TCAAAAGCAGAATTTAGAGGACGAGAATTATGCAAAAGGCTTAAAGATTTAATTCCAGCTCATTTATTTCAAATTGCAATTCAAGCAGCTATTGGTAGCAGAATAATTGCGCGAGAAACT 1200720 1200840

NC_009488_1_Orientia_tsutsugamushi_Boryong_complete_genome probe_cstm_Rprow_021218_1x_10009 GAAATACCTCAATCAGCGTTTATTGCTGCATTGAAGATTACTAATAAAGATTAAGAATAGTAAAGATGAAATAAACGAAGAGATAGTATGCCATTAGATCAGTTTAATTTGAGCTAAGAA 1200960 1201080

NC_009488_1_Orientia_tsutsugamushi_Boryong_complete_genome probe_cstm_Rprow_021218_1x_1001 ACCTCAAATCAGATATTTATAACACTATTCTACATCATTCTCTAGTTTCGAAAGAAGTCTATTTTAAATTAATGCACGATATATCTCTAACTATGAAGGCATATAGAACTTATTATGATC 120000 120120

NC_009488_1_Orientia_tsutsugamushi_Boryong_complete_genome probe_cstm_Rprow_021218_1x_10010 GAACTACTAACCTATGAACAAGAGTTAAAGCGTATATGAGATAATAAAGCAGTTGAAGATTATAAACTCGAACGTGCTAAAGCTGAATTCATAAAGTTTGGTGAGGTTAAAGGTAAAGCT 1201080 1201200

NC_009488_1_Orientia_tsutsugamushi_Boryong_complete_genome probe_cstm_Rprow_021218_1x_10011 AAAGCaaaaaaaGATTTTACAATAAAATTATTGAAATCTAAATTCTAAGTTGAGATGATTGCTGAATATACGGATTTATCAATACAAGAAGTATTAAATCTGAAAAATAGTGCAAAATAA 1201200 1201320

NC_009488_1_Orientia_tsutsugamushi_Boryong_complete_genome probe_cstm_Rprow_021218_1x_10012 TAATGAATATACAACATATAAAATTTTCCACTTTGATGTGTTGTGTGCTATTGCTATTAAATCATATAGATGCTTTATATTACACTATAAGTTCTAAATTATACTCAACATTGAGAAGGC 1201320 1201440

NC_009488_1_Orientia_tsutsugamushi_Boryong_complete_genome probe_cstm_Rprow_021218_1x_10013 CATGCTTTAATACTCCAATGTTCTTAACATATCTTCAATGTTTAGACTACTGTGTTATTATAAGCATCTCTTAGTAGCTAAAGGTTTATTGCTAGCTCCATTAGGATATCCAAGGCTTTT 1201440 1201560

NC_009488_1_Orientia_tsutsugamushi_Boryong_complete_genome probe_cstm_Rprow_021218_1x_10014 AGGCAAAAAAGTTGGCAAATAATGAATCATCTCTTTAAGCAAAATGCTATACAAGAGCTGGTTAAATATAATAAATGCTTACTTTCTGTAACTATATTAGACCTCTTTCGAAACTGGTTA 1201560 1201680

NC_009488_1_Orientia_tsutsugamushi_Boryong_complete_genome probe_cstm_Rprow_021218_1x_10016 TATTACTGTCTGCAGCTAATATAATTGCGATAATGGTTGCGATTACAAAAGAAGAAAAGTGGTTATTAATTCCAGCGATAGAGCCTGATCGTAAAATGAGTGTTTCATCGAAAAATTACC 1201800 1201920

NC_009488_1_Orientia_tsutsugamushi_Boryong_complete_genome probe_cstm_Rprow_021218_1x_10017 ATGAGACTTATTTAAAAGAATTGGTAATTTAGAGTTTTTATCAACACGTCTTCTATAACATATAATACAAGTCttttttcgagttttttGCTTCAACTTTCTCAAGATTATTAACGCCAG 1201920 1202040

NC_009488_1_Orientia_tsutsugamushi_Boryong_complete_genome probe_cstm_Rprow_021218_1x_10019 ATTTCTACAAGTATTAGTTCATAATACTATACTTAATAACCTTAACATTGACCCATTGTAACACTTGTATATTATCTCTTCCTAACTGTTTCTCATTCTAAATTTGCATTTGCAGATATG 1202160 1202280

NC_009488_1_Orientia_tsutsugamushi_Boryong_complete_genome probe_cstm_Rprow_021218_1x_1002 CCCATAGCAGAGACATTCTAGTTAACAAAAAATAGTTATGAAAATATTAACACACTTGAGTTACTTGCTAAGCAAACAAGCAATAGTAAGAGCGCTGTTTACACTAGCATACAGCCCATA 120120 120240

NC_009488_1_Orientia_tsutsugamushi_Boryong_complete_genome probe_cstm_Rprow_021218_1x_10020 AAAGCAATAAATAAATGGCTATTAATTAAAACTATATTAGACCTTATAAATGCCAGAGATCAAATTAAAACTATGTGAGTAGACCTAACCAATTGCTCAATTAGGCCCCTCATCAGAACC 1202280 1202400

NC_009488_1_Orientia_tsutsugamushi_Boryong_complete_genome probe_cstm_Rprow_021218_1x_10024 TCTCTCCTTGAGGTATATTTTAGTCTCCATGTTATGCCAAATCTTGATTTTCTCCAATAGCAAGTAAATCCAAGAAAATTATAACTTGAGATCTTCTTGTCTTGTTTGGCTAAATTTGCA 1202760 1202880

NC_009488_1_Orientia_tsutsugamushi_Boryong_complete_genome probe_cstm_Rprow_021218_1x_1003 TACTCTATATATTAGCGAACACCAACTTTTTTGTACGTCACTTAATGCTGATCCTGATAAATTAGCAACTATGCAATTTGATACTGGATCTGATATATTTCTCACTACTCCAAAAGGAAC 120240 120360

NC_009488_1_Orientia_tsutsugamushi_Boryong_complete_genome probe_cstm_Rprow_021218_1x_1004 TTTCATATAATCAGCTACGACCGCTACAAATGCATCATCCATTTCTTGAGCAGCATATTTACTATGATTACCACTAGTATTTGCAACTTCAAAAGTATTCGTAGACAATATTGGTTTCTT 120360 120480

NC_009488_1_Orientia_tsutsugamushi_Boryong_complete_genome probe_cstm_Rprow_021218_1x_10044 TTAGATTTAATTTGATCTCTGGCATTTATAATTTTGATCTACCTTAACCAGTTTCGAAAGAGGTCTAAtttttgttttttttATGTCTTTCTATAGGAGTCTCAGTAGCATCAATCAAGA 1205160 1205280

NC_009488_1_Orientia_tsutsugamushi_Boryong_complete_genome probe_cstm_Rprow_021218_1x_10045 CTACTTCATAATTCATATCACTCGTCATTAGAGCTTTACGACCTGGAAAGAGCAAAATTTGGGTGTTTAACTAGGGGGTCTTCTACCCATTTTACAGCTTTATAACGCCAGTTTAGGATA 1205280 1205400

NC_009488_1_Orientia_tsutsugamushi_Boryong_complete_genome probe_cstm_Rprow_021218_1x_10046 AGAAAAAGCAGGGAAAGCATAATGTAAAAGGTCTACGAGAGATAATGCACAATTAAATTATGTAATATATTAAACTATAATTTCAATTAATAACAGTTTATTTATTGCTCATATTTCTAC 1205400 1205520

NC_009488_1_Orientia_tsutsugamushi_Boryong_complete_genome probe_cstm_Rprow_021218_1x_10047 AAGTATTAGTTCATAATACTATACTTAATAACCTGAACATTGACCATTGTAATACTTGTATATATATCTCTTTCTAACTGTTTCTTATTCTAAATTTGCATGTGCAAACATGCGAGCAAT 1205520 1205640

NC_009488_1_Orientia_tsutsugamushi_Boryong_complete_genome probe_cstm_Rprow_021218_1x_10048 AAATAAATGGCTATTAATTAAAACTATATTTAATATATCACATAATTATAATTGCACATTATCTCTTGTAGACCTTTTGTATTATGACTTCCCTGCTTTCTCTTATCCTAAACTGACGTT 1205640 1205760

NC_009488_1_Orientia_tsutsugamushi_Boryong_complete_genome probe_cstm_Rprow_021218_1x_10049 TATATGCTCAACTTTCACTAATCCCATAGTTCTGACCTATATGGAAATAAGTACGGTATTCTCTAAGGTATTCTAAGGCCATCAGCAACTGTTCCTCCAAATTAAGCTTATTTTTACGCC 1205760 1205880

NC_009488_1_Orientia_tsutsugamushi_Boryong_complete_genome probe_cstm_Rprow_021218_1x_1005 ATCACACAGATTGTTTAGCCTTGGATGAAGTGCTAATTCAGGATCAATAGAGTCATTTAATAAATCGCTTAACTTTATTTGCGAAATATCATAGTGTCTAGTCACTTCATTGCGAATTAG 120480 120600

NC_009488_1_Orientia_tsutsugamushi_Boryong_complete_genome probe_cstm_Rprow_021218_1x_10050 CACCTTTTGATTTCTTAAGACCATCATCTTTCCTCAAAATATCCACCATCTTTGAAAATGTTCCCTTCCTTACTCCTGTTAATCGACGAAATTTTTCATCACCTAACTCTTTAATCTGAT 1205880 1206000

NC_009488_1_Orientia_tsutsugamushi_Boryong_complete_genome probe_cstm_Rprow_021218_1x_10051 CTAATTTCATTATTACTTCAAATAACATATTTATAACACCATTCTACATCATTGTCTACTTTCGAAAGAAGTCTATTATAGTTAAAAAACAGAAACAGGGCTCCAACAAAAATCTCCATT 1206000 1206120

NC_009488_1_Orientia_tsutsugamushi_Boryong_complete_genome probe_cstm_Rprow_021218_1x_10052 GATAAACTAAAAGAAGGTATGAGTCAATTGATGCATATCAGTATGGAAGAGCTGAACGACTAGGAGTAAGTAAGTCTGGTATACAAAAGAATTAAAGAGATTAAATATTACATAATaaaa 1206120 1206240

NC_009488_1_Orientia_tsutsugamushi_Boryong_complete_genome probe_cstm_Rprow_021218_1x_10056 ATAATACACCTAGAACTCACGGATATGCAAAGGCATAAGATGTTACTGCGTTTATGGTTAGCATCCATAAAAGAAAATTAATATTATAGGAGCATTAGTAGATAAATTTCTGCTCTAGCT 1206600 1206720

NC_009488_1_Orientia_tsutsugamushi_Boryong_complete_genome probe_cstm_Rprow_021218_1x_10057 GTTAGATAGAACAGTATTTAATTCCAACATTAACTAATAATTCTGTGGTTGTGATAGATAATGCAAGTTTTTATAAAAGTCAGCATTTTAAAAACTATAATAGAAAAAGATGGACATATT 1206720 1206840

NC_009488_1_Orientia_tsutsugamushi_Boryong_complete_genome probe_cstm_Rprow_021218_1x_1006 TTTTTCGTATATTTTTTCAAGTTCATTATTATTGAGTGGAACTTTAGTAAAGAATTTTGATAATTGTGATGTTATTTGTGGTTTCCATTTTGAGGTATAGCAGCTATTATTAATTTTAAT 120600 120720

NC_009488_1_Orientia_tsutsugamushi_Boryong_complete_genome probe_cstm_Rprow_021218_1x_10065 TAATTTCATTATTACTTCAAATCAGATTTTTATAACACCATTTTACATCATTGTATAGTTTCGAAAGAAGTCTAGTATTTACCGCCTTATTTTCCTGATTTAAATCTTATTGAGGAAAAA 1207680 1207800

NC_009488_1_Orientia_tsutsugamushi_Boryong_complete_genome probe_cstm_Rprow_021218_1x_10066 TAGTTTCAAGCTAAATCAAGAATAATGAAGTATCACTGTAACTTAGACCAGCTTTTCAAAAAGTATATAATATAACTGTTTTATAATGGTTTAGCTATATATGCTTCATGACGTATGCAA 1207800 1207920

NC_009488_1_Orientia_tsutsugamushi_Boryong_complete_genome probe_cstm_Rprow_021218_1x_10067 AATTTTTGCCAAATTGATTTCCACCCCTTATCATGACTAATGGATTCAAACTTAATTACTTTTGCAAAGTTTACGTGTTGTACAAACTTATCAGCAATAAATGTGGGTACTATCTTGTCT 1207920 1208040

NC_009488_1_Orientia_tsutsugamushi_Boryong_complete_genome probe_cstm_Rprow_021218_1x_10068 TTCATGCCAGATAAATGAAGCTGTGGTATGTACTTAACTTGTTCAGCATAGTCAATAGGATTAAGAGAATCTGTCATGTGAGGATTATTATGAAAATTATTAAATGCAATATGATCAAGA 1208040 1208160

NC_009488_1_Orientia_tsutsugamushi_Boryong_complete_genome probe_cstm_Rprow_021218_1x_10069 TTACCAGCTATAGTTGTAATTGTCCTGACATTAGGGTTAATTGCTGCAACTAATACTGCTAGTCCTCCACCACCAGAGTAACCAATAATATCACATGGTTGATTATTACTAATTTTTACA 1208160 1208280

NC_009488_1_Orientia_tsutsugamushi_Boryong_complete_genome probe_cstm_Rprow_021218_1x_1007 ACCTTTATATACAGCATTTTCAAAATTTATTCCATGACATTTATTAAGCTGGCATAGCGCTTGATTTGTAATATTGACAGTGCCGATCACATCTTCAATTACAGTTCCACCAGCTGTACC 120720 120840

NC_009488_1_Orientia_tsutsugamushi_Boryong_complete_genome probe_cstm_Rprow_021218_1x_10070 ATAGCCTCATTGATAGCATCGACAATTTCTTTGGCAAATCTTTTGTTAGTCCAGTATTCTTTTACGCAATTTTGCTGCAGTGATAGAGGAGTATATTGGCAAGGCCTGGCTATATAAACC 1208280 1208400

NC_009488_1_Orientia_tsutsugamushi_Boryong_complete_genome probe_cstm_Rprow_021218_1x_10071 ACATTTGGTCGCGGATCAATAGTAGCAAGCTCTATTACCAACGGAAGTAATGGTGTCGGATTATTAGATATACTGTCATTATAATATATCAAGCCATCACCTTCAATATAAAACACAAAG 1208400 1208520

NC_009488_1_Orientia_tsutsugamushi_Boryong_complete_genome probe_cstm_Rprow_021218_1x_10072 TTTGTATTTGGGTTTGTGATTTTATGAAAAACTGTAAGTAAGAACTTATTAGTTTTTATTATATCATGTTTCATTTCTGTATTTTGAACAACTTTGTTTGCCCTATTTACACGAGCATTA 1208520 1208640

NC_009488_1_Orientia_tsutsugamushi_Boryong_complete_genome probe_cstm_Rprow_021218_1x_10073 TAGCTTGTATTGATATAACTCGATAATAATAAACTTATAAGTAAGTTTATTAACACTAACTCTAGAtttttttttCGTGCCACAAATACAGAATAATTCATAATTATTTTTATTAATGCT 1208640 1208760

NC_009488_1_Orientia_tsutsugamushi_Boryong_complete_genome probe_cstm_Rprow_021218_1x_10074 TATTACGTTTATTACTAATAACTAGTATACTCAAAAGTTCATTTGAAATATTCTATAAGCTAAAATGGTAGATCATCATCATCTAGTGATTGACTTAAATTTGACTGTGTTTTGTTTTGA 1208760 1208880

NC_009488_1_Orientia_tsutsugamushi_Boryong_complete_genome probe_cstm_Rprow_021218_1x_10075 ACAGACAGCGTATCACTTGGTGTTTTAGTATGAAGAGATGATGCATCACTAGAAGATTTTGAATCAAGCAAAATTAAACTCGCATTATAATTTTGTAATATTATTTCAGTGGTATATTTT 1208880 1209000

NC_009488_1_Orientia_tsutsugamushi_Boryong_complete_genome probe_cstm_Rprow_021218_1x_10076 TCTTGTCCAGATGAATCTTGCCATTTTCTAGTTTGTAAACTACCTTCAATAAGCACTTTACTACCTTTTTTAACATAATTTTTGATAATATTGACTATGCCATCATTAAATACTACTATA 1209000 1209120

NC_009488_1_Orientia_tsutsugamushi_Boryong_complete_genome probe_cstm_Rprow_021218_1x_10077 CGATGCCACTCTGTTTTCTCCTTCTTTTCATTGCTATGCTTATCACGCCATGTTTCTGTTGTTGCTAAGCTAAATGCTGCTATCTCTTTACCGTCATTTGTTTTTCTAATTTCTGGATCA 1209120 1209240

NC_009488_1_Orientia_tsutsugamushi_Boryong_complete_genome probe_cstm_Rprow_021218_1x_10079 ttgttatgtttattttgaaacatgttttaGTAAGTATGATATACACTGATTTATATGAATCATGACTTACGAAAAACAAGCTTATAACGCCAGATTAGGATAAGTGGAAATATGAAATTT 1209360 1209480

NC_009488_1_Orientia_tsutsugamushi_Boryong_complete_genome probe_cstm_Rprow_021218_1x_1008 GCTTGTTATTATAAGACTAGGAGAAGTTTCATGAATAATTTTTTCAATTAATACAGTAGTTGGCACTTGATTTATTTCACAATTAAACTTTTTTGCTACAATATCGTCATTAATATTTGT 120840 120960

NC_009488_1_Orientia_tsutsugamushi_Boryong_complete_genome probe_cstm_Rprow_021218_1x_10080 AGAGAATAAAAGGTATACAAAGAGAGAATGTTGAGTTATaaaaaaaaTTTAAATAAACAAGGAATAACCCAACATGAAAAAATGTATTATAACAGTATACTATTTAATAGACAATTTTTG 1209480 1209600

NC_009488_1_Orientia_tsutsugamushi_Boryong_complete_genome probe_cstm_Rprow_021218_1x_10081 CAAGATATATCACGAGTTGGAGAGAAAGAGGTTAATACCAAGTAGAGTAATCAAAGGAACAGAGATGGGAAGTTGTCCTTAGCTGAGTTATTAACAATAGACCTCTTTCGAAACTGGTTA 1209600 1209720

NC_009488_1_Orientia_tsutsugamushi_Boryong_complete_genome probe_cstm_Rprow_021218_1x_10086 CCTAGTAATGTGGGTGGAGCAAAGGGTTAGCAGAAAAGTAAGAATGCAAGAGGGAAACAATGGCTGTACACAGCATAGACAGAAATACATGGTTAACGAAACTTGAGCGTATAAAGTTGC 1210200 1210320

NC_009488_1_Orientia_tsutsugamushi_Boryong_complete_genome probe_cstm_Rprow_021218_1x_10087 TATCATCGAAAAATCAAGACATAAAGTTTAATAATCTAAATCAAGACATAAAGTTTAATAATCTTGGACATATCATTGATTTAAAGATGTTAGAAGAACAATATAAGGAACTCGATAGCA 1210320 1210440

NC_009488_1_Orientia_tsutsugamushi_Boryong_complete_genome probe_cstm_Rprow_021218_1x_10088 AGAAAGCGATAGGAATAGATGGTATAACCAAAGCTGATTATGGTAAGAAGTTGAAAGCAAATCTGCTCTCGCTTCTTACTAGAATTCGCAAATGGCAATATCAAGCTAAACCTGCACGAA 1210440 1210560

NC_009488_1_Orientia_tsutsugamushi_Boryong_complete_genome probe_cstm_Rprow_021218_1x_1009 AATTAGCCCATCTGTTGATGGATGCATATCTGACTTGTATAATAAAAGTGTTTTTCCATTTGATAATTGAAATATTGCATATCTTCCCCAAGCTTCTAGTTTTTTAGAAGGAAGAACAGC 120960 121080

NC_009488_1_Orientia_tsutsugamushi_Boryong_complete_genome probe_cstm_Rprow_021218_1x_10098 TCTTATTTTTGAGAGCCGGATGCGGTAATTCTGCAACTCCGGTTCTGAGGAGGGGCCTAATTAAGCAATTGGTTAGGTCTACTCACACAACAACAAGATGTAAAGGAGTTAGGCCATGCA 1211640 1211760

NC_009488_1_Orientia_tsutsugamushi_Boryong_complete_genome probe_cstm_Rprow_021218_1x_10099 CATTCTGCATATTGGGATCAGCATCGTGATCAAGTAGAAGCTGTACAATATCTAGGTTGTTAATAAAAGAATTTGCTGTACAATGTAATGGAGTTTGATCAACATTGTCTTTTACATTTG 1211760 1211880

NC_009488_1_Orientia_tsutsugamushi_Boryong_complete_genome probe_cstm_Rprow_021218_1x_101 AGCGAAAATCATCAAAAAAGTTTAAATAATAAAATTGATTTTTTATCAAGTAGTAAGGAAGTATTACTAAAGTCTAAAGTCAAGCATCAGTTTGATATTAACCaaaaaaaaGAACCTGCA 12000 12120

NC_009488_1_Orientia_tsutsugamushi_Boryong_complete_genome probe_cstm_Rprow_021218_1x_1010 CCCCTTTAACTCAACTGCATGCATAAAATCTTGATATCCAGATTTATATAAGCGCCAGTTAGTATTGTTAAAAGCATTACTATCAGCTTCTCTATATTTTATACTGCCGTTATTGaaaaa 121080 121200

NC_009488_1_Orientia_tsutsugamushi_Boryong_complete_genome probe_cstm_Rprow_021218_1x_10100 TATTAGCGCCATTCTTTACTAAAAACTTGATAATTGCATTTATATCGTCATTTACACTGTGGTTATTAGTCTCATGCTGTGTTAAAATCCCGACAGAATCTGCAATATTTTTACCATTAT 1211880 1212000

NC_009488_1_Orientia_tsutsugamushi_Boryong_complete_genome probe_cstm_Rprow_021218_1x_10101 AATTACCACCATCCGGAATAATAGACATATGCAAAGCTGTATGTAAAGCAGTGTGTCCACTATTATTTTTTGCATTGATGTCAGCTCCATTGTTCAGCAAAATCTTAGCAATAGGTATAT 1212000 1212120

NC_009488_1_Orientia_tsutsugamushi_Boryong_complete_genome probe_cstm_Rprow_021218_1x_10102 TCTTATATTTTAAAGCAACAGATAAAGCGGTAATACCGTCTTTATATTTTGAATTGATAAGTGTACTATCATTATTAATAAGATGCTTCACTTTTTCAGTATCACCATCTTTAATAGCAC 1212120 1212240

NC_009488_1_Orientia_tsutsugamushi_Boryong_complete_genome probe_cstm_Rprow_021218_1x_10103 GATGTAAAGCATATCTATATTTAGCCTTAGATTCAGACATCATTTTACTCCTGAAATTATACTATCACTATTGTAATATAATCTTCAGTACAGCTTTGCTTAGCAGCAAAGTATAGAACG 1212240 1212360

NC_009488_1_Orientia_tsutsugamushi_Boryong_complete_genome probe_cstm_Rprow_021218_1x_1011 aaCATGATTCAAGGCTGCAAATTCAGCGTTTGTCCATACGATAGCAACATAATCTGCTTTGGGTAGCTTAGCAACTGATGAACTCTCTTCAGAAGCAACAAACTTGGGATTCCCAATGCC 121200 121320

NC_000963_1_Rickettsia_prowazekii_str__Madrid_E_chromosome_complete_genome probe_cstm_Rprow_021218_1x_101164 aaatgttgagccagaattattaaataatattattaaattTCGACTTGATGAAATTGAACATAAAAATATTGCTATAATATATAGTACTGAGTCAATATTTACAGACATCATTAGTAAAAT 229200 229320

NC_009488_1_Orientia_tsutsugamushi_Boryong_complete_genome probe_cstm_Rprow_021218_1x_1012 AATATTACTAAAATCAATAGCTGGTAGGTTTGGAAGATTTGTATGGCTGTCAAAATTATCAACTGAATCTGATTGAGAGAAAGAAAGTATATGTCTAGTTTTTATGTACATAAAATAATC 121320 121440

NC_009488_1_Orientia_tsutsugamushi_Boryong_complete_genome probe_cstm_Rprow_021218_1x_10120 ACCACGTTTTTATCCCGCGTAAATATAGCAATTTAGTAGCTATACTCAAAATTTAACACTCTGAAAGGCTTATAATAACTTAAGTAGTTTTAAATATTGAAAGGTATATATTACATCGAT 1214280 1214400

NC_009488_1_Orientia_tsutsugamushi_Boryong_complete_genome probe_cstm_Rprow_021218_1x_10122 ATAGACCTCTTTCGAAACTGGTTGACGTAGTTCAAAATTATAAATGCCAGAGATCAAATTAAATCTAAGACCTAATCTTTTACGTCTATTTTGATATTTATCAGCAATAATTTTGAACTA 1214520 1214640

NC_009488_1_Orientia_tsutsugamushi_Boryong_complete_genome probe_cstm_Rprow_021218_1x_10123 CGTCAACCAGTTTCGAAAGAGATCTAATCAATAATAAAGGCGAAATAATGTCAGTTAAAATTATTAAAGGCAATAAAAGCGACCTATCTGTATCTTCAGTTTTTTCTAAAGGCTTATCTG 1214640 1214760

NC_009488_1_Orientia_tsutsugamushi_Boryong_complete_genome probe_cstm_Rprow_021218_1x_10124 GTAAATTGTTTAGTGATAAAGCTTACATATCTAAAAAGTTATTTCATCAACTGCTGACCAATTGTCTACGTTTATTTACTAATCTTCGTAAAGATATGAAAACATATTTATTGGACATAC 1214760 1214880

NC_009488_1_Orientia_tsutsugamushi_Boryong_complete_genome probe_cstm_Rprow_021218_1x_10125 AAGATAAGCGGTTATTAAATAAACGTTCTTTAATTGAGTCTGTCTTTAATGTACTaaaaaaaCATATGCATTTAGAGCATACTAGACACCGTTCTCCTCTTAATTTCTTTGTTCATATAA 1214880 1215000

NC_009488_1_Orientia_tsutsugamushi_Boryong_complete_genome probe_cstm_Rprow_021218_1x_10126 TTGCTTCTCTTGCTAGTTATTCTATCTCTAAACTTAATCCCCATCCTATCTCTTCTTCTTCTCCTGACTCCTTATCCTAAATTGACGTTTGAAAGCAATAACTTCAGATAGGCCATAGGC 1215000 1215120

NC_009488_1_Orientia_tsutsugamushi_Boryong_complete_genome probe_cstm_Rprow_021218_1x_10127 AATAATATGAAATTATCAGGAAAATGTAATACGTGAAACCCATACGTTACGTTTGATATAGCATGTGATGGAAACTGTGATAACTAAACTACTGTGCCTTAACTTGCCCATCCTTAGAGG 1215120 1215240

NC_009488_1_Orientia_tsutsugamushi_Boryong_complete_genome probe_cstm_Rprow_021218_1x_10128 CGAAGATAATGGCAATATTTCTTTGTTAAATGACAAGATATACTTGCTAGCAATATTAGAGCAGCAAATATTAAATATACAGTACTGAATTGAGATTGTACTTAATGTAATGTATTAAAA 1215240 1215360

NC_009488_1_Orientia_tsutsugamushi_Boryong_complete_genome probe_cstm_Rprow_021218_1x_10129 ATTATAACAAAAAATTGTGCTTTATATACCACTATGATTATACTTTTCATTTCCATTACTCAGCTCTAATCTGCTAATTATATCAAACTCAGGTTATAATCAAGGTGTTTTAGACAAACT 1215360 1215480

NC_009488_1_Orientia_tsutsugamushi_Boryong_complete_genome probe_cstm_Rprow_021218_1x_1013 TCGTATTTTTTCTTTAAGAAGCTTAAATACAATTTTAAATTGCGTTAGTCAACAGTAAATTTAGATACTATAGCTAAACCAGTATAAAACAGTTTATTATATACTTTTCAAAAAGAGTAA 121440 121560

NC_009488_1_Orientia_tsutsugamushi_Boryong_complete_genome probe_cstm_Rprow_021218_1x_10130 TTTACTAGATTTGATAAAAATACTTTTTTCATCTAGTATATTTTTTACAGTTGTTGTTTTATAGTTACGATAAAGTAAGTGCACAGAGCCAGTTAAAGTAATTTTTGCGCGATTTAAAAA 1215480 1215600

NC_009488_1_Orientia_tsutsugamushi_Boryong_complete_genome probe_cstm_Rprow_021218_1x_10131 TTATATTTACCATATAGTATTTTAATGATAAAATTGCTTTTGATAATTTTATTAGTGATATATACTTCTAAAGTAACTTAAGTAATTACTTAAAATAAAAAGATATGGCAAGATTTCCGA 1215600 1215720

NC_009488_1_Orientia_tsutsugamushi_Boryong_complete_genome probe_cstm_Rprow_021218_1x_10132 TTACTATAAAAGGGTTTCATAAGCTTGAGCAAGAACTTAAGCATTTAAAGTATGTTGAGCGGCTTAAGATTACAACAGACATATCTACTGCTAGAGAGTTTGGAGATTTATCTGAAAATG 1215720 1215840

NC_009488_1_Orientia_tsutsugamushi_Boryong_complete_genome probe_cstm_Rprow_021218_1x_10133 CTGAGTACAAGGCAGCAAAGGAAAGGCAGTTATTAAATGATaaaaaaaTTTACGACTTAGAAAATAAATTAGCAAATGCAGAAGTTATTGAAATAACTAAGATTAATAGTAACTCAGTTA 1215840 1215960

NC_009488_1_Orientia_tsutsugamushi_Boryong_complete_genome probe_cstm_Rprow_021218_1x_10134 AGTTTGGGGCGCGAGTAGTATTATTGGACCTAGATACTGAAAAGGAAGTAGTATATCAAATTGTTGGAGAGTATGAAGCAGATATTACACAAAACCTTATTTCTATAGCATCTCCAATAG 1215960 1216080

NC_009488_1_Orientia_tsutsugamushi_Boryong_complete_genome probe_cstm_Rprow_021218_1x_10135 CACAAGCATTGATTGGAAAAAAGGCAGGAGATATTATTGAAGTTATTACTCCTAAAGGTGGAAGATTTTATGAGCTACTAAAAGTTCAATATGTTGATTTTTAAAGTAATGCATTTAATT 1216080 1216200

NC_009488_1_Orientia_tsutsugamushi_Boryong_complete_genome probe_cstm_Rprow_021218_1x_10136 CATAGTTACATATAAAATAAAGAAATTGTTATGGATAATGAAATAGATAATTTACCAATATCAGCTTCAGATACAGATAATGGATTTTATATTTACTTACAAAAGATTAATAGAATTCCT 1216200 1216320

NC_009488_1_Orientia_tsutsugamushi_Boryong_complete_genome probe_cstm_Rprow_021218_1x_10137 TCTTTGACTGCCGAGGAGGAATATATGCTTGCAAAGTCTTATTTTGAGCAAAACAACTTAAAGGCAGCGCATTCATTAGTCAAAAGCCATCTTAAGCTGGTTGCTAAAATAGCTTTGTCT 1216320 1216440

NC_009488_1_Orientia_tsutsugamushi_Boryong_complete_genome probe_cstm_Rprow_021218_1x_10138 TATCGTGGCTATGGTCTTCCAGTAGTTGATTTAGTTTCAGAGGGGAATATAGGGTTACTACAAGCAGTaaaaaaaTATAATCCTGAACTTGGCTTTAGGTTGTCTACATATGCAATTTGG 1216440 1216560

NC_009488_1_Orientia_tsutsugamushi_Boryong_complete_genome probe_cstm_Rprow_021218_1x_1014 CTACGTCACAGCGATATTTCATTCTTCTTGATTTAGCTTGAAAGCTTTTTTCAATGTGATTTAAATCAGGAGAATAAGGAAATAAATACTCTAATATATGCCAGATTAGGATAAGAAAAA 121560 121680

NC_009488_1_Orientia_tsutsugamushi_Boryong_complete_genome probe_cstm_Rprow_021218_1x_10140 AAGATGCATGCAAGAGCTATTAATTCATCTGATTATAAGCAAATTGCTGATGAGTCAGGAGTATCAATAGCAGAAGTATCAGAAGTTAATCAACGATTATCAAACTCCGATCTATCGCTT 1216680 1216800

NC_009488_1_Orientia_tsutsugamushi_Boryong_complete_genome probe_cstm_Rprow_021218_1x_10141 AATACTCCTATTAGTTATCACGAAGATGATGCTACTGAATTAGTTGAATTAGTACCAGAGCATAGACCAAATCAAGAAATAATTTTAGCTAATCAGCAGGATTTAAACTTaaaaaaaGAA 1216800 1216920

NC_009488_1_Orientia_tsutsugamushi_Boryong_complete_genome probe_cstm_Rprow_021218_1x_10142 GTTTTGGAAAACGCTTTACAAACTCTTACACAAAGAGAATTATATATTATTCGTGCACGAAAGCTACAAGATGAACCTGATACGCTAGAAAAATTAAGTTTGCACTTTAATGTCTCCAAG 1216920 1217040

NC_009488_1_Orientia_tsutsugamushi_Boryong_complete_genome probe_cstm_Rprow_021218_1x_10143 GAGAGGATTCGCCAGATAGAAAGCAAAGCTTTTGAGAAGCTACAGCAGTATGCAATGAACACTAAGCATAAGTTATTACATTTTGTTTAGTAATGTAGAGTAAGGCTTGAtttttttGCT 1217040 1217160

NC_009488_1_Orientia_tsutsugamushi_Boryong_complete_genome probe_cstm_Rprow_021218_1x_10144 GTAGTATAATATTTCAATTTCATTATTGCTATATAAGCTTTGATGCAAGATTTATACTTTATTACTTGAAGAACTAGATAATCTGTAAATTAAATATCAATAGTATAAATTAAACATTGT 1217160 1217280

NC_009488_1_Orientia_tsutsugamushi_Boryong_complete_genome probe_cstm_Rprow_021218_1x_10146 TAACGTAGTTCAAAATTATTGCTGATAAATATTGAAATAGACGTAAAAGATTCGGTCTGAGATTTAATTTGATCTCTGGCATTTATAATTTTGAACTACGTTAACCAGTTTCGAAAGAGG 1217400 1217520

NC_009488_1_Orientia_tsutsugamushi_Boryong_complete_genome probe_cstm_Rprow_021218_1x_10147 TCTAATAAAAGCGAAATAATGTCAGTTAAAATTACTAAAGGCAATAAAAGCTACCTATTTATAGCTTCAGTTATTTCTAAAGGCTTGTCTGATAAATTGTTTTGTGATAAAGCTTACATA 1217520 1217640

NC_009488_1_Orientia_tsutsugamushi_Boryong_complete_genome probe_cstm_Rprow_021218_1x_10148 CCAAAAGCATTATTTCATCAACTGCTAACCAACGATTTACGTATGTTTACAAATCTTCGTAAACATACGCAAACATACTTATTGAACATAGAAGATAAGCTTTTATTAAAAAGATGTTCC 1217640 1217760

NC_009488_1_Orientia_tsutsugamushi_Boryong_complete_genome probe_cstm_Rprow_021218_1x_10149 TTAATTGAGTTGTCTTTAATGTACTaaaaaaaCATATGTATTTAGAGATACTAGACATCGTTCTCCTATTAATTTCTTGGTTCATATAATTGCTTCTCTTATACAACTTATTCTATCTCC 1217760 1217880

NC_009488_1_Orientia_tsutsugamushi_Boryong_complete_genome probe_cstm_Rprow_021218_1x_1015 GCATGAAAAGCATAATATAAAAGGTCTACAAGAGATAATGTACAAGTGTTACAATGGTCAATGTTCAGGTTATTAAGTATAGTATTATGAACTAATACTCGTAGAAATATGAGCAATAAA 121680 121800

NC_009488_1_Orientia_tsutsugamushi_Boryong_complete_genome probe_cstm_Rprow_021218_1x_10150 AAACTTAACCCCTATTTATCTCCTCTTCTTTTCTTTCATCTATTCTATCCTAATCTGATGTTTAAATAGTATACTGTTATAATACATTTTGTCATGTTGAGTTATTCCttttttttattt 1217880 1218000

NC_009488_1_Orientia_tsutsugamushi_Boryong_complete_genome probe_cstm_Rprow_021218_1x_10152 TTTAGTACTTTGATCTTTCATACAAAAAGTCAGATCGAATGAATCACCAACTGTTAATTGTTTTGTAACTCCAGTTAGCATAATATGGAAACCTCCAGGTTTTAACTTAAATGTAGTTTG 1218120 1218240

NC_009488_1_Orientia_tsutsugamushi_Boryong_complete_genome probe_cstm_Rprow_021218_1x_10153 CAGAGGAATTAATAAATGCTCAATATGGCTCATACTAATAACTCCATTATTATCTCTTATGGATTGATGGAACATAGCTGCATTAGCTATATTAGGAGCAGAAACACCTATAATTTCAAG 1218240 1218360

NC_009488_1_Orientia_tsutsugamushi_Boryong_complete_genome probe_cstm_Rprow_021218_1x_10154 ATTACTAAGAGACTGATTGTTAATTGTTAAGTAAACAGCAGTATTTTTACCTTTCTCTGCTGGTCTTGCCCATGCATTTAGGAATTGAACTGCAGTATTGCTAAATTCGTTATTATTAGA 1218360 1218480

NC_009488_1_Orientia_tsutsugamushi_Boryong_complete_genome probe_cstm_Rprow_021218_1x_10155 TAGTGATGTATTGTCTTGGTCAAAATGTTGGCTATTGTGATGTACTAAGCAACAGCTAGAACTAACCTCTTTTGGCTCTAATTGAAGCTCTGTAGCATAAATTTGCCAAGTAAAAATATT 1218480 1218600

NC_009488_1_Orientia_tsutsugamushi_Boryong_complete_genome probe_cstm_Rprow_021218_1x_10156 TGATAAAGTTAATAAAATTACTGTACTTTTTCTCATATATCTTTCCAATTTGTAAAAAATAGTATATATAATAACTGTATTTCTTTATGTATAAAAGATAAAATGAATACAAAGTATAAT 1218600 1218720

NC_009488_1_Orientia_tsutsugamushi_Boryong_complete_genome probe_cstm_Rprow_021218_1x_10157 TATTTATATTTATTATATCCAAAAGCAACATAGCTAATAAGTCTGATAATAGAAGAGATGTTACCATATCTCGCATTCTATAAGGTATGATCGAGTAATTATGAGGTAGTTTTGTGATTT 1218720 1218840

NC_009488_1_Orientia_tsutsugamushi_Boryong_complete_genome probe_cstm_Rprow_021218_1x_10158 CAGTTTTCATCAGCTATCTAGTCAAAAATAGCGTAAAAATTTCCCGCATTATGCTTTTCTGATAGCCTAGTGTTGCCTATGGATTATCTGCTGTTAATGCTTTCAAAAATCTATAATATA 1218840 1218960

NC_009488_1_Orientia_tsutsugamushi_Boryong_complete_genome probe_cstm_Rprow_021218_1x_10159 GAATTTAGTAATCATTATATAATTCCATCTTCTCAAACGGCTAGAGTTACCAATAGTATAAACTTTAAATTTAATGATGTAGAAAGATTTAAAGAACAGTATCTAACTAAGATTAAATCT 1218960 1219080

NC_009488_1_Orientia_tsutsugamushi_Boryong_complete_genome probe_cstm_Rprow_021218_1x_1016 TAAACAGTTATTAATTGAAACTATATTTAATATCTCACATAATCATAATTGCACATTATCTCTTGCATACCTTTTACATTATGCTCTTCATACTTTCTCTTATCCTAATCTGGCGTTTAT 121800 121920

NC_009488_1_Orientia_tsutsugamushi_Boryong_complete_genome probe_cstm_Rprow_021218_1x_10160 ATTATAGATCATAGTATAAAGGCTTAGTTGAAGCGTAAAATAGCTTGAGCTTTTGAGAGGCGGATACTGCTTATCAAATTTTGATCTCATGTGCTTTTGAGTAAGTAGTTGAAAACAAAT 1219080 1219200

NC_009488_1_Orientia_tsutsugamushi_Boryong_complete_genome probe_cstm_Rprow_021218_1x_10161 ATTACATCAAGTTACTTAAGAATACTTCACTATCAGATCATATTAAATTTAAGATATAGCAAAACCAGTAAAAACAGTTACGTTGTATACTGTCCAAAAATGGTGTCTACGCCACAGCAA 1219200 1219320

NC_009488_1_Orientia_tsutsugamushi_Boryong_complete_genome probe_cstm_Rprow_021218_1x_10162 TACTTCATTCTTCTTGATTTAGCTTGAAACTATTTTTTCAATAAGATTTAGGAAAATAAGAAGGTAAATACTATAAATATATGTTCATTTTTTCTATCATAGTTTTTAAATGAGAACTTT 1219320 1219440

NC_009488_1_Orientia_tsutsugamushi_Boryong_complete_genome probe_cstm_Rprow_021218_1x_10163 TATAAAAACTTGCATTACATATCACAACTACAAAATTATTAAGGTAATTTCTGAATTAAATCCTGTTCTATCGAACAGTCAAAAATTGATACTGTTAGCAGCAATTTATCTACTAATCAT 1219440 1219560

NC_009488_1_Orientia_tsutsugamushi_Boryong_complete_genome probe_cstm_Rprow_021218_1x_10164 CCTGTATAGCCAAGGAACTATAAAATAGTAGTTATATTATATACTTTTCGAAAAGGATATCTAGGGCATAGTGATAGCCAAACTATTATAACTGTTTTATAGTAGTTTAGCTATATATTA 1219560 1219680

NC_009488_1_Orientia_tsutsugamushi_Boryong_complete_genome probe_cstm_Rprow_021218_1x_10165 AAAGAAGAGTATAAATTGTTAAAATTAAGATTTGGTCTGGTGGTAGTATCATGTTGTCATAGACTAGTCAAATGGCATGGTTAACATAGTGCGATGTGAAGTTATGTATATGATTGTTTA 1219680 1219800

NC_009488_1_Orientia_tsutsugamushi_Boryong_complete_genome probe_cstm_Rprow_021218_1x_10166 ACGGCGAGAGCAGATTAAACCTGTTGTTCTACCAACAGAAGCCTACTGGTACATGCAATGGAGTGATCCTATGTATGAAGCTATCAGGACAATACATTCAGTTGTTAAGAAGATTTAACT 1219800 1219920

NC_009488_1_Orientia_tsutsugamushi_Boryong_complete_genome probe_cstm_Rprow_021218_1x_10167 AACTTGAAGAAATAAGTTAGAGTAAGAGTTATTATGAGCTTTATACACTTTATAAAAGCTGCTATTTAGTTTATACTCGTTTGAGTTGATATATAAACTTAACTGTATTACAATATGATG 1219920 1220040

NC_009488_1_Orientia_tsutsugamushi_Boryong_complete_genome probe_cstm_Rprow_021218_1x_10168 ATAACACCTTAATGATAATTGCGCAAATATAACGCAAATTTATATATGACACTTAGCTAATTGAGTGCTGATTTTAAATCCAACAACTTAAATTTATAGTTGATAAGCTTAGAGCAATCT 1220040 1220160

NC_009488_1_Orientia_tsutsugamushi_Boryong_complete_genome probe_cstm_Rprow_021218_1x_1017 CATCTATGTCCAATAAATATGTTTTCATATCTTTACGAAGATTAGTAAATAAACGTAGATTATTGGAGAAGAGTTGATGAAATAACTCTTTAGATATGTAAGCTTTATCAGCAAACAATT 121920 122040

NC_009488_1_Orientia_tsutsugamushi_Boryong_complete_genome probe_cstm_Rprow_021218_1x_10170 tatttaattacttcaattttaaatttatGAATGTGGTGATAAATATGTCAGTTGGATAATTCCATCTTATCATATAACTTTCTTCTACTCCGCTTTTTCCAGCTGAAGCCTTTCAATTGA 1220280 1220400

NC_009488_1_Orientia_tsutsugamushi_Boryong_complete_genome probe_cstm_Rprow_021218_1x_10172 AGCTAAACTACTATAAAACAATTATAATAAATTGTCATaaaaaaaaTTCATAGCCTAAGGTTAAAGTGTAAACCAGCTTTTTGATTTATGCTATATAATATAACTCTTCTAACTCTTAAG 1220520 1220640

NC_009488_1_Orientia_tsutsugamushi_Boryong_complete_genome probe_cstm_Rprow_021218_1x_10173 TTATCACAATAAATTTTATTTTGTATTTTTAAGCTGTTAATTTTGCGCTTAATCCATTAAATTATATGTATCTGTGACCTGAGAAAGGCTGAGGATCTTTTTTCTTAGCTTCGCTTGTCT 1220640 1220760

NC_009488_1_Orientia_tsutsugamushi_Boryong_complete_genome probe_cstm_Rprow_021218_1x_10174 TATTTTGTTGAATTTTTGATAAATCTTCATTACTCAGATGCTTTAGTATATTGAGTTTGACTTCAGGTGGTAGAACATTCCAACTTTCCTTGCTTTCCGTGGAATGTTCTTTAGGATGTT 1220760 1220880

NC_009488_1_Orientia_tsutsugamushi_Boryong_complete_genome probe_cstm_Rprow_021218_1x_10175 CTTTACATATATCATCCATTGATTCAAGTGCTCCATGTAGCAGCTGGTTTCTAGTACTTCCTGCATCTATATGTTTTTTGAGGTCATCGGAATATATAGGAAAATTTTCTTCACAACATT 1220880 1221000

NC_009488_1_Orientia_tsutsugamushi_Boryong_complete_genome probe_cstm_Rprow_021218_1x_10176 CATAAATTTTTTGAACTTTAGGATCATTTATATATCTTGATAATTCATTATCATCTGTTGTATTAGCAAGAAAAGCACTCAACATACtttttttGCCACCACCAACTTTGATGTTATTTA 1221000 1221120

NC_009488_1_Orientia_tsutsugamushi_Boryong_complete_genome probe_cstm_Rprow_021218_1x_10177 GtttttttCTAGTTCTTTTTCACACGCAAGCTTATACTCACTAAGAGTTAATGATTGTTGTATAAGTTGCTGATTTGTTAGGCTGCCTGCTGAATTAGTCTTTTTATCACTAGAATATTC 1221120 1221240

NC_009488_1_Orientia_tsutsugamushi_Boryong_complete_genome probe_cstm_Rprow_021218_1x_10178 TAACTTGGTAATATGAGCAGTTAAAAATTCAGGAATTCCTGTTTGCATATTGATGATCGAACTACATAAAATAGCATTACTCAGCGGTGTACAACCTCTGCTATCATATGCATTAATATC 1221240 1221360

NC_009488_1_Orientia_tsutsugamushi_Boryong_complete_genome probe_cstm_Rprow_021218_1x_10179 AGCACCATACTTTAATAAAGCTTGAATAGCTGGTAAGGCACTATATATTTTTTCAGCAGCATGATGTAACGGAGCGCTACCGTCATTGTTTTGTGCATTAACATCAGCTCCATGATGTAA 1221360 1221480

NC_009488_1_Orientia_tsutsugamushi_Boryong_complete_genome probe_cstm_Rprow_021218_1x_1018 TACTAGATAAGCCTTTAGAAATAATTGAAGCTACAGATAGGTAGCTTTTATTGCCTTTGGTAATTTTAACTGACATTATTTCGCCTTTATTATTGAAGGTAACAAAGCATCAATTTTTGT 122040 122160

NC_009488_1_Orientia_tsutsugamushi_Boryong_complete_genome probe_cstm_Rprow_021218_1x_10180 TACAGCTTCTACAATTCTTTCATTTGGAGCACGGCCTTGCGCAGCACAATGTAAAACACTGTTACCATGTTGACCTTTTAAATGCACGTTTGCACCACTTTCCAATAAACAAACAACAGT 1221480 1221600

NC_009488_1_Orientia_tsutsugamushi_Boryong_complete_genome probe_cstm_Rprow_021218_1x_10181 AGAGAGGAGTCCATTTCTTGCAGCACGATGCAAAGAAGTATCACCTACATTATTTTGTGCATTAACCATTGGCACCATGATTTAACAATATCTCAGTCACATCTAGGTGGTGGAAAATGG 1221600 1221720

NC_009488_1_Orientia_tsutsugamushi_Boryong_complete_genome probe_cstm_Rprow_021218_1x_10182 TTTGCTGCGTAATGTAAAGCTGTATTACCATTCTTATCTTGTAAATTAACGTTTGCACCATGGGTTAATAATGCTTGAGCAATTTTTAGTGATTCTGTACTTAGCAAACTAGAAACAGAT 1221720 1221840

NC_009488_1_Orientia_tsutsugamushi_Boryong_complete_genome probe_cstm_Rprow_021218_1x_10183 TGGGAACTAACTAGTCTTGTTAATGCTAGGTGTAAAGGAGTACGACCCATGTTGTTTTGTACATCAACAATAGTTTTATTATAACTTAACAGTATTTCAGTTATTTTTAAATTGTAACAC 1221840 1221960

NC_009488_1_Orientia_tsutsugamushi_Boryong_complete_genome probe_cstm_Rprow_021218_1x_10184 TGAGCAGCTAAATGCAATGGTGTGTTTTGATTATTATCTTGCAGTGTAGCATCAGCATTATGCCCTAATAGCACATTAATAATTTTTTCTTGTTTATATATACTGAACTGATAAATGCAT 1221960 1222080

NC_009488_1_Orientia_tsutsugamushi_Boryong_complete_genome probe_cstm_Rprow_021218_1x_10185 AGGGGTTCTGCCATACCTATCTTGCGAATTAACAATAGTAGGATCTACATCTAAAATACGCTTTACAGCATTTACATCACCATGCTTAATAGCTAAACTCAAAGCAACAGTATTCATATT 1222080 1222200

NC_009488_1_Orientia_tsutsugamushi_Boryong_complete_genome probe_cstm_Rprow_021218_1x_10186 GATTACCTCTTAATCTTAGTTAATTAGCACTTTTCAATTTTAAATTGAGTTCAATATTATTCTAATTAATTAAACTATACAATTATTTTTTATTACCAAAGTTCGATATAAGGAGAATAT 1222200 1222320

NC_009488_1_Orientia_tsutsugamushi_Boryong_complete_genome probe_cstm_Rprow_021218_1x_10188 gaaaaagtatcaaaaCGTTTTGAGCTTGATCATGCAAAAACTCTGTTCTTTTATTAGCTAACTCAATAAATATCTCTCCTTTACGATATGACTGCCAGTTGCTTAAAAAATTCTCTATAT 1222440 1222560

NC_009488_1_Orientia_tsutsugamushi_Boryong_complete_genome probe_cstm_Rprow_021218_1x_10189 TATTACTACCAACCATCAATACTTGCAGGCTACTTTCAGCTGGTAAATTTTCATCGCTTTTCAGAAATTCAGCAATTTCATTTTGAGCCGAAACGCTAGCTCCAACCAATGGCCATGCAA 1222560 1222680

NC_009488_1_Orientia_tsutsugamushi_Boryong_complete_genome probe_cstm_Rprow_021218_1x_1019 TACAGGTGGCCTAGTATCAGTTTTCAATGGTGATAGCGGTACTGGTATAGACTTAGGAGTTGGTACAACAATAAACTTATTAAGAGATATATATTTAGATATTGAATGTAGCACAATGAA 122160 122280

NC_009488_1_Orientia_tsutsugamushi_Boryong_complete_genome probe_cstm_Rprow_021218_1x_10190 GCAATACAAAACCTATTGAACCACAATTAAAGAATAGCTGAGTCTCCTCATCATATGATTCATAGACAAAATGTTTAGAAAATCTTTCTCGATCAAAATCCTTATGTATGCTTGCGTTCA 1222680 1222800

NC_009488_1_Orientia_tsutsugamushi_Boryong_complete_genome probe_cstm_Rprow_021218_1x_10191 CCTTGACTCTGTTTTGATTTAAATAAATTAATAAGATAtttttttggttttttAGGTGAAGTGTTAACAACAGCTACACCTTGAACTGCTTTAGATCGCTTACAGTATAGAACACAACGC 1222800 1222920

NC_009488_1_Orientia_tsutsugamushi_Boryong_complete_genome probe_cstm_Rprow_021218_1x_102 GTATTTAAGCTCAAACCTAAAACTTCACAAGGAAGGAGCATGGTTTGACTATATAGTCTAACTCATCAAATGTATCAATTTATAGATTATCTTGTCTATTAACGTCAGTTTAGGATAAGA 12120 12240

NC_009488_1_Orientia_tsutsugamushi_Boryong_complete_genome probe_cstm_Rprow_021218_1x_1020 GTATAGACCACTTCCTATTCATACACGTTTTGGGTTGCGATTTCATTTCTAATTACTCACAGGTTTTATAGTTAGTAATTATTAGCTCATTAACAGGATTGCGTTGTTCATTGATTGAGT 122280 122400

NC_009488_1_Orientia_tsutsugamushi_Boryong_complete_genome probe_cstm_Rprow_021218_1x_1022 ATCTAATTTGATCTTTTTCATCAAATGGAAGTCTAGTGTAGAACCGTTCTCCAGATTTATGATAAGGAGGATCAAAATAAACAAAGTCACCTTTTTTAGGTTCTATAAATGAAAAATATG 122520 122640

NC_009488_1_Orientia_tsutsugamushi_Boryong_complete_genome probe_cstm_Rprow_021218_1x_10224 AACATCTTATTAATAACATTATCTTCATTTACAACTTTTAATTTACTAAACCATGATTTATCGATAGCTTGACCATAACTTTTAATCAAAGATTCTCGTACTTTGTACCAAGTTGAGTTG 1226760 1226880

NC_009488_1_Orientia_tsutsugamushi_Boryong_complete_genome probe_cstm_Rprow_021218_1x_10225 GAGTCCAATTGTTTACTCAGATTCAAGAGATACTCTTCTTCATTGATTTGTTTCTGTGAAACAGTTTTGAGTTGCTCAAATGGTGTAACTTGCAATTCTTGAATTTAGTATCCATATACA 1226880 1227000

NC_009488_1_Orientia_tsutsugamushi_Boryong_complete_genome probe_cstm_Rprow_021218_1x_10226 TCTTGTACAGCTTGTAATATCTTTGATTCATCACATTCTGTTAGTGTGATATTTTTAAGCAACTTAACAAAAAACCTAGTTCGAACCGCTGGCCCAAAACTACAAGAAGTCAAAATTTGA 1227000 1227120

NC_009488_1_Orientia_tsutsugamushi_Boryong_complete_genome probe_cstm_Rprow_021218_1x_10227 TAAGCCATAGCTGCTTCAAAAGAACCAGCAATTTTATGCTTCAACTGACTTTCCTTACTAAGATTTGCGCTTGTTTCAATTTGAGCAAGATATTTTTCCTTATTAAATTTTTCTACGTTA 1227120 1227240

NC_009488_1_Orientia_tsutsugamushi_Boryong_complete_genome probe_cstm_Rprow_021218_1x_10228 TCAAATTTACAGTTGCCACTATTTGCCTGATCAGTAGTTCGTAATTCATTTGCTAACGCTTGTGCTATATAGTTTAGAACTGATGCCTTACAGCCAAAATGATAATCCGGAGATCGACCT 1227240 1227360

NC_009488_1_Orientia_tsutsugamushi_Boryong_complete_genome probe_cstm_Rprow_021218_1x_10235 CCATTCTGGTGGAATGATATTTCCAATAAAGTTATAAAATATTGTGTCATTATTGTTACTATTATTTTTAATAATATTACAACTATTAGTTAAAAAATCGAATACTACAGGGCGCATTTT 1228080 1228200

NC_009488_1_Orientia_tsutsugamushi_Boryong_complete_genome probe_cstm_Rprow_021218_1x_10236 TCATCTCCAAAAATACAGCTATAGTAGGTGTTTTAGAAAATAAtttttttATAAAATTACAGTCAAATTTTTGTTCAAAAGGTTGAAAATAGGTTGaaaaaaaTCATTATGCCAAACATA 1228200 1228320

NC_009488_1_Orientia_tsutsugamushi_Boryong_complete_genome probe_cstm_Rprow_021218_1x_10237 TATAAATCTTCATACTGATACTATCTGCCTTAGAAGACCAATTTTTCCTATAGATCATGTTATTACATAGAAGATAAGCAGTTATTAAAAAACGTTCCTTAATTGAATCTGTCTTTAATA 1228320 1228440

NC_009488_1_Orientia_tsutsugamushi_Boryong_complete_genome probe_cstm_Rprow_021218_1x_10238 TACTGAAAAAACATATGCATTTAGAGCATACTAGACATCGCTCTCCTATTAATTTCTTTGTTCGTATAATTGCTTCTCTTGCTAGTTATTCTATCTCCAAACTTAATTCCAATCTTATCT 1228440 1228560

NC_009488_1_Orientia_tsutsugamushi_Boryong_complete_genome probe_cstm_Rprow_021218_1x_1024 TATAAATTCCCCTAAAAGAATATTTATTAAGATATATAAATTTTGCAGTAATTTCATTCGGATTATTACTATTGCATTGTTTATATGGATTATGACTATGAAAAAATTCGTATGATCGGA 122760 122880

NC_009488_1_Orientia_tsutsugamushi_Boryong_complete_genome probe_cstm_Rprow_021218_1x_10240 ATCCCCTTCGAAACTGGTTAACGTAGTTCAAAATTATAAATACCAGAGATCAAATTAAATCTAAGACCGAATCTTTTACGTCTATTTCGATATTTATCAGCAATAATTTTGAACTACGTT 1228680 1228800

NC_009488_1_Orientia_tsutsugamushi_Boryong_complete_genome probe_cstm_Rprow_021218_1x_10241 AACCAGTTTTGAAAGAGATCTAATGTCTATTCTAACGGTATCAATTTTTGATTGCAATGTTAATACTGCTATTTTTAACTGTTTGATAGAACAGGATTTAATTCAGAAATCACCTCATAA 1228800 1228920

NC_009488_1_Orientia_tsutsugamushi_Boryong_complete_genome probe_cstm_Rprow_021218_1x_10242 TTCTGTAGTTATGATAGACAATGCAAGTTTTCATAAACGTCATCATTTAAAAACCATCATAGAAAAGAGATATATAGTCATTTACAATGAGGTTTGAGCATAAAAAGAAGAGATAAAAAT 1228920 1229040

NC_009488_1_Orientia_tsutsugamushi_Boryong_complete_genome probe_cstm_Rprow_021218_1x_10243 TAATAGTATTGGTTATAAAGTTAtttttttGCTACCTTATTCTCCAGATTTAAACCTATTGAAAAAATAATTTTAAGCTAAATTAAGAAGAATCAAATATTACTATGACGTAGACACCAT 1229040 1229160

NC_009488_1_Orientia_tsutsugamushi_Boryong_complete_genome probe_cstm_Rprow_021218_1x_10244 TTTCGATTCAAAAAGTATATAACATAACTGTTTTACAGTGCTTTAGCTATAGAATTTGTACTATACGCTGAATTAGTATTATTAAATGTGCTATCTTTTATATTACTTAAATTTAGGTCA 1229160 1229280

NC_009488_1_Orientia_tsutsugamushi_Boryong_complete_genome probe_cstm_Rprow_021218_1x_10245 GTATTATTTAAGTTGGCATTATCAAAATTAGAATTTGATATCTTAGCTAATGTAAATTTAGTATTGGTAAAATTAGAGTTTTTAAAATCTGATTGATTAATTTTTGCTTCATGAAAATTA 1229280 1229400

NC_009488_1_Orientia_tsutsugamushi_Boryong_complete_genome probe_cstm_Rprow_021218_1x_10246 CAGTTGCTAAAGTCTGAATCAGTAATGCTTGTATTAGATATGCTAGTTCTTCTGAAAATACTATTTTGTAAATTTATATTAGATAAATTATATCCTGATATTTTTAACTCGCTAAAATTC 1229400 1229520

NC_009488_1_Orientia_tsutsugamushi_Boryong_complete_genome probe_cstm_Rprow_021218_1x_10247 ATTGAAGATAGATCTTGGTAGCTAAATGCTTGGTTTAAACTATAAGCAATGTTAGCAGTTATTGCATTCTCAATTTTATCAGAATTTAGAATAATACAATTTGTTAATTGGAGCTTATTA 1229520 1229640

NC_009488_1_Orientia_tsutsugamushi_Boryong_complete_genome probe_cstm_Rprow_021218_1x_10248 AAATCACAATTTTTATGAGTAACATTGTTCATTATTCCACCAATCATAACTGAATTTTTCAATGAGCTTGAGTCaaaaaaaTTATTAATAAAAGCTGCTTGTTGCAAATTTAGTAAATTT 1229640 1229760

NC_009488_1_Orientia_tsutsugamushi_Boryong_complete_genome probe_cstm_Rprow_021218_1x_10249 AAATAGCTATGATAAAATTTGTTGTCTCTAAATCTTGTTCTAATTAAAACAATATTATTAAAGTTTGACTCTTTAAAAGTTACATTAATAAGTTCACTATCATCCATATAGGAATCTTTA 1229760 1229880

NC_009488_1_Orientia_tsutsugamushi_Boryong_complete_genome probe_cstm_Rprow_021218_1x_1025 TATGGAGATGAAATACAGCAAATACTAACTCAGCTGCTTGATAATGCAAATCGATTTGGAGAAAAAGTTAAAGTAATACTCATTAAAGTTAAACTACTAAATCCCGAAATACGAGACAAA 122880 123000

NC_009488_1_Orientia_tsutsugamushi_Boryong_complete_genome probe_cstm_Rprow_021218_1x_10250 ATTACTAACTTACTAGATTCGCTATGCTTAATAGCTATATTATTTAGCTGCGATTGATTTATAGTAGTATTAGAAATTGCTCCTTGTGTAATAATAGCAGAGTTAGCTTGGCATTCATGA 1229880 1230000

NC_009488_1_Orientia_tsutsugamushi_Boryong_complete_genome probe_cstm_Rprow_021218_1x_10251 ATTACTGTATTATTTATGTTGCTCTTACTAAATGTTACTTTATTTAATTGATTTTTAATAAAAACCACCTTATCTAATGTAGTTGTTGAAAAATATGCTTGTTGTAGATCGGAGTGATCA 1230000 1230120

NC_009488_1_Orientia_tsutsugamushi_Boryong_complete_genome probe_cstm_Rprow_021218_1x_10252 AAAGTTGTATTTGTTATGACAGATAACAATATCGGTACATTAGTTAAAGATGATTGGCTAAACCTCGAATTGTTAATTTTAGTATTATCAATAACAGCATGTGATAAAATTGAAGaaaaa 1230120 1230240

NC_009488_1_Orientia_tsutsugamushi_Boryong_complete_genome probe_cstm_Rprow_021218_1x_10253 aaaGTAGAATTATCAATCGTGCTATAATTAAAATCGCTGGATTCAAGGTAGTTCGCAATAACTTTAGTAAAAGAAAAATGTGAATGCGTTAAATCTGCTCCTAAAAAATTATTGTTTATT 1230240 1230360

NC_009488_1_Orientia_tsutsugamushi_Boryong_complete_genome probe_cstm_Rprow_021218_1x_10254 AGTTTACTACGATGAAACTTTCCAAGGCTAATACTAGCACTTTGAAAGTTTGAATTAACAATAGTACTGTTTAAAAAATGAATGGATTTAGCTATAACATTTACAAATTTAGTATTTTGT 1230360 1230480

NC_009488_1_Orientia_tsutsugamushi_Boryong_complete_genome probe_cstm_Rprow_021218_1x_10255 ATATTACATGCATCAAAACGACTGTCTTCTAAGTTTGACCTAGAAAAATCAGAATTGTGAATCTCTGATTTAATAAATTTAGATGCTCCAAAATCAGCATTAATAGCTTGAAACTGAGAC 1230480 1230600

NC_009488_1_Orientia_tsutsugamushi_Boryong_complete_genome probe_cstm_Rprow_021218_1x_10256 ATCATAGATTGTTCCAGTTTACATTCCTGTATTACAGAGCCATTAGCTGTTGCATTAGTAAGATCGCAGTAAGATAAATTTACTCTTATGAATTGAGCATTGCTAATATCTACGTTTGAA 1230600 1230720

NC_009488_1_Orientia_tsutsugamushi_Boryong_complete_genome probe_cstm_Rprow_021218_1x_10257 AAATCAGTATGACTTAGGTCTAATGATATCAACCTTAAACCACGTAAATTTGTACCAAACTTTTTCTTTAGCTCTATTGGAGCCTTTTTAGCGTTTTGAGTATCAATATACTTTCTAATT 1230720 1230840

NC_009488_1_Orientia_tsutsugamushi_Boryong_complete_genome probe_cstm_Rprow_021218_1x_10258 TCTTCATTTTGTTTTGGAGTAAGTAATTTGTTTTTGGGCATAATGCCAACACGAGCTCTGTGTGCTGTTAGTAGCAGCAGAACCATCATTATATTAGTCAGTTTTAAAACCATTCTCGGT 1230840 1230960

NC_009488_1_Orientia_tsutsugamushi_Boryong_complete_genome probe_cstm_Rprow_021218_1x_10259 ATGTATTTACGTTTTTTAATATTAAGTGTTTTAAAATACCTAAAGAACAATCATCTGTCTATCATTAAAATATATCATTAAAGGAATATATTATTACCAGTTAAGCTAGACACTCTTTAT 1230960 1231080

NC_009488_1_Orientia_tsutsugamushi_Boryong_complete_genome probe_cstm_Rprow_021218_1x_1026 ACACTAGAACTTACAATTCGAAATAATGGAAACAACATaaaaaaattacaaaaaataaaTACTGCTTTTAAAACTCCAAATGAAGATAACATCTTAGGTTTAGGGCTGACATTTGTTAAG 123000 123120

NC_009488_1_Orientia_tsutsugamushi_Boryong_complete_genome probe_cstm_Rprow_021218_1x_10260 TTTGCTGTCCTGTTGCAAGTAAATTATCATCACTCGGATAATCATTATTATGTGCATCTTTATTACTACAATTAATGTTCAGCAATTTAAGCTTTCGATGTAATGCTGACCTTTCCATTC 1231080 1231200

NC_009488_1_Orientia_tsutsugamushi_Boryong_complete_genome probe_cstm_Rprow_021218_1x_10261 CAACAAAAGCAGAAGTTTTAGAAATATTATTATTAAATCTATTCATTTGAGCAACAAGATATTCACGCTCAAATACTTCTCGAGCTTTTCTTAAAGGCATAGACATCATATTAACCTTAG 1231200 1231320

NC_009488_1_Orientia_tsutsugamushi_Boryong_complete_genome probe_cstm_Rprow_021218_1x_10262 CCTTAGATTTTATAATACTTACTGCCTTATTATTGATAATATCATGTGGAAGCATATCTACCTCAATTTCATGGTTGCTATTTGATGTTAGTGGATTCATGATTAAGGTCCATTCTATTG 1231320 1231440

NC_009488_1_Orientia_tsutsugamushi_Boryong_complete_genome probe_cstm_Rprow_021218_1x_10263 CATTTCTCAATTGTCTTATATTTCCTAGCCAATCATAAGCTTGCAAAGCTGCAATTGCTTCATTTGAAAACTTTCTGGCCTTCAATCCAGATGACTTTGCTAGCTGCTGAACAAAATGTT 1231440 1231560

NC_009488_1_Orientia_tsutsugamushi_Boryong_complete_genome probe_cstm_Rprow_021218_1x_10264 GCACAAGCAGAGGAATATCTTCCTTACGCTTAGCAAGTGATGGAACATGAATTGGAATCACATTTAGCCTGTAATATAAATCCTGTCTAAATCTTCCTAGTGTTACTTCTTCTTGAATAT 1231560 1231680

NC_009488_1_Orientia_tsutsugamushi_Boryong_complete_genome probe_cstm_Rprow_021218_1x_10265 TTTTAGCAGTAGCGCTAATTAATCTAATATTAAGTTTAACTGATGTACTAGAACCAtttttttCGAAATTATTATCTTGTAAAAATTTCAGTAATCGAGATTGAAGGCTTAGAGGTAAAT 1231680 1231800

NC_009488_1_Orientia_tsutsugamushi_Boryong_complete_genome probe_cstm_Rprow_021218_1x_10266 CAGCAATTTCATCAATATAAAGTGTACCATTATTGGCAGCTTCAAGCGCGCAAACCTTATAAGTAAAATCTGAGTTGTTATTAATATTTCTCTCACCAAATAATTCGTATTTTGCTTGTT 1231800 1231920

NC_009488_1_Orientia_tsutsugamushi_Boryong_complete_genome probe_cstm_Rprow_021218_1x_10267 CTTCAGTAAGAGCTGTTGGACAAAAAGTAACAAATGGTCCTAATGCATATTTTGAACGCCGATGAATGAGTTGTGCTACTAGCTCCTTTCCACACCCAATATTACCAGTAATCATTACTC 1231920 1232040

NC_009488_1_Orientia_tsutsugamushi_Boryong_complete_genome probe_cstm_Rprow_021218_1x_10268 TGCCAGAAGTTGGCGAAATTTTCTCTATTTCTGCTTTAAGTTTGTTGATTGCAGGTGACTGCCCTATAATCTCTGATCTATCAATTACTTTGGTTTTAAGATCAATGTTTTCtttttttA 1232040 1232160

NC_009488_1_Orientia_tsutsugamushi_Boryong_complete_genome probe_cstm_Rprow_021218_1x_10269 ACCTTGCTGTTTCACATGCTCGCTTTAATACTGTAACTATTTTGTCAGCTGTAAATGGCTTCTCTACATAATCATATGCTCCCATTTTGATAGCAGTAACTGCTGTTTCAATTGTAGCAT 1232160 1232280

NC_009488_1_Orientia_tsutsugamushi_Boryong_complete_genome probe_cstm_Rprow_021218_1x_1027 CTGCTACTTCAAGCAATAAAGGGAGAAATTACATACGTAAAAAATGAGCATACAGAGGTTATATGCCGTGTACCGATATATGCTCACTATCTTTAATTTTCTAAAATAAGGGTGATTTAT 123120 123240

NC_009488_1_Orientia_tsutsugamushi_Boryong_complete_genome probe_cstm_Rprow_021218_1x_10270 GACCACTGATAATTATAACAGGAACTAAAGGATGtttttttCTTATAATTTCTAATATTCCTAATCCATCTAAATCACTGCCATGCAACCATACATCCAGAATTACTATTTTAGGTAGTT 1232280 1232400

NC_009488_1_Orientia_tsutsugamushi_Boryong_complete_genome probe_cstm_Rprow_021218_1x_10271 TTACATCTAGAATACTTAGTGCTTCAGAGCTATTTTCAGCAGTGGTTGGTGTAAATCCTTTACTTTTAACAATATCTGATAGCAGTGCTCTAACATCGTGTTCATCATCTATTATTAAAA 1232400 1232520

NC_009488_1_Orientia_tsutsugamushi_Boryong_complete_genome probe_cstm_Rprow_021218_1x_10272 CATGGCCCATAATTTATCTCCTGAGAATTGTACTAATGATTTAAGTGGTTAACTAAAATTTGCTTATAATCTTAAATTAGTACTCTATTCTAGATAAATCAATAAGAAATAATTATTTTA 1232520 1232640

NC_009488_1_Orientia_tsutsugamushi_Boryong_complete_genome probe_cstm_Rprow_021218_1x_10273 ACTATAAAATTGATAAAAATTAGTGCTATGCTACTCATAGCACCGATTAAGACAGGTTTGCAATTTTAAATTGTCAAATATAATTTGAACTGCTGCTCCTCCATTTGGTAAATTTCGAAT 1232640 1232760

NC_009488_1_Orientia_tsutsugamushi_Boryong_complete_genome probe_cstm_Rprow_021218_1x_10274 TTGTAATGTACCAAAGTGATCCTCAACAAttttttttACAATGGAAAGTCCTAAGCCACTACCTCGTAAGCGTGTTGTAACATATGGCTCAGTAATATAGTTAATTAAATCTAGAGAGAA 1232760 1232880

NC_009488_1_Orientia_tsutsugamushi_Boryong_complete_genome probe_cstm_Rprow_021218_1x_10275 TCCATTGCCAGTATCTTGAACCTCAATAGTTACAATTTCAAAGACAGGTTGTTGTAAGAATATTTTAATTATTTTAGTGGAATGAAACTTAGATTCCAAAGATTCTTCAGCATTGTTTAA 1232880 1233000

NC_009488_1_Orientia_tsutsugamushi_Boryong_complete_genome probe_cstm_Rprow_021218_1x_10276 TAAGTTCGCAATAATTTGATGAATTTGAGTAACATCTGCTACAAAATCTAGTTTGCTGAGTGTACTAATAAACTTATACTGTATTTTCTCATTTATTATTTGCCTTGATTCAACTAACCC 1233000 1233120

NC_009488_1_Orientia_tsutsugamushi_Boryong_complete_genome probe_cstm_Rprow_021218_1x_10277 TTTAATCACTGCAACTAGATCGCAGTTAGTAAACTTAGGAACTGGTATTTTAGCAAAATCAGCAAATTCTTTAATAATTGTTTTAATATCTTTAGCATGCCTTGAGATAGTGTTTAGATA 1233120 1233240

NC_009488_1_Orientia_tsutsugamushi_Boryong_complete_genome probe_cstm_Rprow_021218_1x_10278 CTTTGTAAACATAATAGGATCACTTACTTCTTTACCAAATTTATCTGCTAGCATACTAGCAGATAGCTGTATAGGAGTAAGTGGATTATTAATTTCATGAGCTACTTTTCTTGCTATATC 1233240 1233360

NC_009488_1_Orientia_tsutsugamushi_Boryong_complete_genome probe_cstm_Rprow_021218_1x_10279 AGACCAAACTAATGTTTTCTGCGTTATTGTTAAATCTTTGTTTTGATATTCTAATTGCTTTATCATTTGGTTAAATACAGAAGTTAGAAAATCTAATTCATTCTCAATATTTGTGTGCTT 1233360 1233480

NC_009488_1_Orientia_tsutsugamushi_Boryong_complete_genome probe_cstm_Rprow_021218_1x_1028 GATATTAGAAAAAGAATTCGATTCAGAAATAGAAATGAAGATTACTAAATCTGCTCTAAATGCAGTTGCAGAATATAAAGGAATAAAACTTAGTTGCGATTATTTAAGTACAATAAAAGA 123240 123360

NC_009488_1_Orientia_tsutsugamushi_Boryong_complete_genome probe_cstm_Rprow_021218_1x_10280 TAATTGAATATTTAGCTTTGCTAGTTTAATAGATTTAGTCGCAGTAACTAATAATTCTGTAACCGGTAGAAAAATTCTATTTGCTAAAATCAAGCTCATATGAATTGCTATTAGCAGCAT 1233480 1233600

NC_009488_1_Orientia_tsutsugamushi_Boryong_complete_genome probe_cstm_Rprow_021218_1x_10281 TAATAAAGCAATACAGAGAAAAGCAATTAAACATTTTACCTGTAGTAAAATTAGTTGATATTTTAGCTTAATATATTCTTGATTAGAATCAAAATTTTTAGTTAGATAATCTATAATTTC 1233600 1233720

NC_000963_1_Rickettsia_prowazekii_str__Madrid_E_chromosome_complete_genome probe_cstm_Rprow_021218_1x_102819 tattattaataataatattatagttataaatataataaGTGAGTATTTTAAAGCTTTAGGCATAGGAATGCATTTCTTTTAATAGGTTATTTTGATAATATTTTATAAAAGGTTATCTGT 427800 427920

NC_009488_1_Orientia_tsutsugamushi_Boryong_complete_genome probe_cstm_Rprow_021218_1x_10282 TTTATGTTTTAAAAAGTGCCAAAGCTTGTTAGTGTTAGTGTATAATTTTGCTATTTTCTTGCTAGTTAGCTCATTTGCTATATTCAACATCGTATTTGGTTCGTTAGAGTTGCTCTTTAA 1233720 1233840

NC_009488_1_Orientia_tsutsugamushi_Boryong_complete_genome probe_cstm_Rprow_021218_1x_10283 ATAAAGCCTGCTAATTGTTGATGAATAGCAAAAAATTTTGTTAATATATTGGTCAAAGTAATTTTGAATAGCAAAGTTaaaaaaaaaTATAAACAATACTGCAAGCGCAATTATTGGTAT 1233840 1233960

NC_009488_1_Orientia_tsutsugamushi_Boryong_complete_genome probe_cstm_Rprow_021218_1x_10284 TACAGCAGTTATACTAAAAGTCACTGCAACTTTTTGTTGTAACCTTTCAGTCACTGTTTTAACATTATATCTTAAGTATTTCCATACTACTTTAGCTAGTAATATTATGATAATTAATAA 1233960 1234080

NC_009488_1_Orientia_tsutsugamushi_Boryong_complete_genome probe_cstm_Rprow_021218_1x_10285 AGATGTGAAATCTAATAATAAAGAATAACTGATAAATTTAGAGTTATAGGTAATATAATCCTTAGTaaaaaaaaCAATAAAGAAGTATACTGAATTAAGCAGTAGAATTAGGCTATAAAT 1234080 1234200

NC_009488_1_Orientia_tsutsugamushi_Boryong_complete_genome probe_cstm_Rprow_021218_1x_10287 atgatatattaaattttttttATTTATAGCTGAAGCTAAATGTGTACTGTTGTTTTTATTACTTAACTGCTGTTATCAATAAGGTATCGAAAGAACACAAGTTCGATATAAGAATAGATT 1234320 1234440

NC_009488_1_Orientia_tsutsugamushi_Boryong_complete_genome probe_cstm_Rprow_021218_1x_10288 AATTAGTAGAAAGAGGTATTGCAACAACCGCTTAAACACTATAAAATAGAAAACAAGATAAGTAATATTAAAACCTGCTAGAGCTATAAAAAGAAACAAACATAACTGTGATGATCTATC 1234440 1234560

NC_009488_1_Orientia_tsutsugamushi_Boryong_complete_genome probe_cstm_Rprow_021218_1x_10289 TAGCAGATATTTTCTAGTGAATAGAAAGTTTTGAAAAGAAAGAATTGCAAAATAATGTTTAACACTATACATACATCTCAATATTTTGCACCACTTACTCTCCCTATATTTTGATACATT 1234560 1234680

NC_009488_1_Orientia_tsutsugamushi_Boryong_complete_genome probe_cstm_Rprow_021218_1x_1029 AGTAATTGGTGACAATATGTTACTAAGACATCTAGCAGCTGGTGATAAAGAGCTTTTAAAACTTACACATCAACTTAAAACACCAAACCTAAATATAATAGATGTGTTCAAACAGCTCAA 123360 123480

NC_009488_1_Orientia_tsutsugamushi_Boryong_complete_genome probe_cstm_Rprow_021218_1x_10291 taatgtatttttGGCTTGGTAGCTCAGTGGTAGAGCGTAGGACTGAAAATCCTTGCGTCGCCGGTTCAATCCCGGCTCAAGCCACCATATTTACTCATGCTATCTTGAGTACTTTTGAAT 1234800 1234920

NC_009488_1_Orientia_tsutsugamushi_Boryong_complete_genome probe_cstm_Rprow_021218_1x_10292 TTATTGACTTAGCTTGAATCTATTACTCTTCAATTTAGTCTTATATTCACATTCTTGTTTATAACAATATACTTCTACTATATTGGACTTATTCAATATAGCGGCATCAATTATCTTTGC 1234920 1235040

NC_009488_1_Orientia_tsutsugamushi_Boryong_complete_genome probe_cstm_Rprow_021218_1x_10293 TGTAGTGTATTTGCATTTATCATTTGGTATTACACTAATTATCAAATCAGCTGGTACGCATTCAGTATTTTTAAATCTAATAGATACTATTTTACCTGAGTTAGTAATAATTCTATGGTT 1235040 1235160

NC_009488_1_Orientia_tsutsugamushi_Boryong_complete_genome probe_cstm_Rprow_021218_1x_10294 CAATGTTTCTATTGCACTATCACGAAATAGAATTTCATCCTGCCCATACCAATTACTCCAATAATTTaaaaaaaaaTCTGGTATGTTAGAAGAAGCATGTATCTCGAATTTACTATTGGC 1235160 1235280

NC_009488_1_Orientia_tsutsugamushi_Boryong_complete_genome probe_cstm_Rprow_021218_1x_10295 ATTCTTAATACCAAGAGCTGTAAGTTCACCATTGAAAACAAGATCTGGTTTAGAAACTAATGCTGCTCTTATAATAGCTATAATAATTATTACTACTCCAATCATCCTCCAGCATTTATG 1235280 1235400

NC_009488_1_Orientia_tsutsugamushi_Boryong_complete_genome probe_cstm_Rprow_021218_1x_10296 CCAAATAACTACCCATAAGAATCCAAACAAATAGAGTAATAAGCTAACATCATCAATATGCCCAAAGTAAGTTACTGCATACGGAAGCGTTGAGATTATCTTAGCTATTTTAATTATAAC 1235400 1235520

NC_009488_1_Orientia_tsutsugamushi_Boryong_complete_genome probe_cstm_Rprow_021218_1x_10297 ATCAACTCCTTGCCCTGCTAATAACAAGGAATAGTTTTCTATGTTAAAAGGCATTAGTAATAAAGATACCAGAGTTAATGGCATAACCCAAAATGCTGTTAATGGCACTATCAATAGATT 1235520 1235640

NC_009488_1_Orientia_tsutsugamushi_Boryong_complete_genome probe_cstm_Rprow_021218_1x_10298 TGCTAATACTGAGTAGTTAGGGAAACTGTAAAAATGATATATTACAATTGGAGCAGTTGCTATGCTAGCAAAAGCACTTGAATATATATTTGACTCAAAAAATAGCTGTATTTTAGATAA 1235640 1235760

NC_009488_1_Orientia_tsutsugamushi_Boryong_complete_genome probe_cstm_Rprow_021218_1x_10299 TATATCTTTTTGATCTACATTGAATTTATATAGCTGTATAACACTTTGGAGCTCATAGCAACTAATCAATGATATCACAGCTATAAACGATAGCTGAAAACTCGGATGCATAATATATTC 1235760 1235880

NC_009488_1_Orientia_tsutsugamushi_Boryong_complete_genome probe_cstm_Rprow_021218_1x_103 AAAAGTATGAAAGGCATAATACAAAAAGTCTACAAGGGATAATGTACAATTATGATTATGTGAGATATTAAATATAGTTTCAATTAATAACCGTTTATTTATTGCTCATATTTCTACAAG 12240 12360

NC_009488_1_Orientia_tsutsugamushi_Boryong_complete_genome probe_cstm_Rprow_021218_1x_1030 AAGCGAGCCAGAAGAAGTTAGAAATTTTCAGTCCGAAATAACAGAAAATAAAAAATCAATATTAAGCAAACACTTAAACTGTTATAGCGAAGTATTAATGTATATTAAGACTGCATATAA 123480 123600

NC_009488_1_Orientia_tsutsugamushi_Boryong_complete_genome probe_cstm_Rprow_021218_1x_10300 TGGATTAAATAACAGAATTATAATAGCAGCTAAGCCAACTGATCGTAATGGAACTACTCCACGCTTTATTAGCACAACTACCATAGTAGTTGAAGTCATTATAAAAGCTCTAATTGTAGC 1235880 1236000

NC_000963_1_Rickettsia_prowazekii_str__Madrid_E_chromosome_complete_genome probe_cstm_Rprow_021218_1x_103007 ATATATCATCCTCAGTATTTATTATAATTTTAAGTTGTTTCTTCATTAAAATTTTCCATTCTTTTATATTATGTACTTACATAATTTGGTGTATCATAATCTTTCATTAAGAATTCTTGT 450360 450480

NC_009488_1_Orientia_tsutsugamushi_Boryong_complete_genome probe_cstm_Rprow_021218_1x_10301 AATATGCCCATCAGCTAATACTAGATAGCAAAAACTACCTATTAAAGACATTATTATTGCTATAGTTCTTACATCACAATTAAAAGCAACAGTGGTTGATAAATTTAAGATAAATCTGCA 1236000 1236120

NC_009488_1_Orientia_tsutsugamushi_Boryong_complete_genome probe_cstm_Rprow_021218_1x_10302 TCCAATAAACAATGCAATTGCAACCATAGAAAGATGTAATCCTGATATGCACAATATGTGTGATACTCCAGCTTGCCTCATACTGATAATAATATCTTTATTAAGTGCATGATTTTCTCC 1236120 1236240

NC_009488_1_Orientia_tsutsugamushi_Boryong_complete_genome probe_cstm_Rprow_021218_1x_10303 TAATAAAATCGCATTAATAAAACCACCTAAATAACTATCTAAAGCATCTATAACTCTATCATGTATTTTAATTCTAATATACTCAATAGTAGCAATTAATTTATGGCTTAGTAATCCATT 1236240 1236360

NC_009488_1_Orientia_tsutsugamushi_Boryong_complete_genome probe_cstm_Rprow_021218_1x_10304 GTTTTGATTATTACTTTGAACTAATTTCACTGATGATGTTGCATAACCTATTGCGCTGATATTGTTAAAATATGCAAGTATATTGAATGGTAATTCATTAATCATTAGTGTATTACTTGT 1236360 1236480

NC_009488_1_Orientia_tsutsugamushi_Boryong_complete_genome probe_cstm_Rprow_021218_1x_10305 TGGAGGATATAAAGTTACATTTAAAGTAACTTGATCTCCAACCGTAATATTATGCACTTGTTTTCTAAAAGCTGATATTTTTATACTTGCTAATGTATTATAATTATCAAGTGTTTCAAC 1236480 1236600

NC_009488_1_Orientia_tsutsugamushi_Boryong_complete_genome probe_cstm_Rprow_021218_1x_10306 ATAGCAGTTATCAATAACTAGTATCACTCCTCTAGAAGTAGGATGAAttttttttATTGTTCCATAAATTTTAGATTTTATAATCGTATCAATATGGTATGTGTTTGCGGTTAAATATCT 1236600 1236720

NC_009488_1_Orientia_tsutsugamushi_Boryong_complete_genome probe_cstm_Rprow_021218_1x_10307 TATTTTACCAACTATGATACCTAAATAGCAACAAAACAGAATTAACAGTAATGTTCGGCTTAAAAAGCTATTTCTTATAACTATGAAAATAACTATGAAGATTGGTAATACAAGCAAAAT 1236720 1236840

NC_009488_1_Orientia_tsutsugamushi_Boryong_complete_genome probe_cstm_Rprow_021218_1x_10308 TTGCCAAATCTGGAGTTCAAAAGGCAAAGCAAAATATAGCGCTATGCCAAATATAAAACTAACAATAAACCATAAACCAATATTATGATACTCTTCTGCTATTAATTTATTAACTGAAGA 1236840 1236960

NC_009488_1_Orientia_tsutsugamushi_Boryong_complete_genome probe_cstm_Rprow_021218_1x_10309 TATAATCATTAATTTGTTATTAGGTTGTGTTGTAAAATCAAAATTTGATCTGACTAGATAAAATATTATAGAAACACAGAAATTATACTTCTTTCATTTCTTCTATGTTTCTATCTTAAT 1236960 1237080

NC_009488_1_Orientia_tsutsugamushi_Boryong_complete_genome probe_cstm_Rprow_021218_1x_1031 ACTGTTGACAGGAAGAACTGAATCATTTGTACAAGCAAAGAAAGAAGTACTTTATTTAGACGGAATATTAAACAATGCTATTAAAAATTATAATGAATCGAAATCAAAGTTTTTATATAA 123600 123720

NC_009488_1_Orientia_tsutsugamushi_Boryong_complete_genome probe_cstm_Rprow_021218_1x_10310 ATTTATGCTATAATACTACTTTGATACTAACATAAATAGATAAAAAACTTAAGATATACTACTATCTTTCACTGCTCCACATACTTCATATTGAGATTCCATTACTGGTTCTATTACTGG 1237080 1237200

NC_009488_1_Orientia_tsutsugamushi_Boryong_complete_genome probe_cstm_Rprow_021218_1x_10311 TTCCATTGGTAGTGGCCTGTTTTCGTACTTTTTCGTTTCTTCAATGCTATCAATATTAATAGTTATATCTGCATTTTCTCTATATTTTTTGTATGCTAAAGTAGCAGCGTAACCAGCTAA 1237200 1237320

NC_009488_1_Orientia_tsutsugamushi_Boryong_complete_genome probe_cstm_Rprow_021218_1x_10312 TAGACCTACTACACATCCACTAGATATCCATAATAGCACTGTTGACAAATTGTTTTTATCATTATTAGCATTCATTTCATTATAACTAGCTACTAATAACTTTATACTACAGTTTGGTGC 1237320 1237440

NC_009488_1_Orientia_tsutsugamushi_Boryong_complete_genome probe_cstm_Rprow_021218_1x_10313 TACTACACATGCTCCTCTGCTCCACTCTGTTAGAGTAGCATTTATATCATAATTATTACTAGCATATACTTTAGCAACAACTATGTTGTTATCATTTATCCCTGACGTAAAATTAGCAGG 1237440 1237560

NC_009488_1_Orientia_tsutsugamushi_Boryong_complete_genome probe_cstm_Rprow_021218_1x_10314 AAAAATTAACTTCAAGTTTTGGTCATCTTTGATGATTGCATCTTTATTAGAAACAAGGTTATGTAAAACTGTCATAGGAATATAAGGACCATCAATTCTATTGATATAACTATTATGAAT 1237560 1237680

NC_009488_1_Orientia_tsutsugamushi_Boryong_complete_genome probe_cstm_Rprow_021218_1x_10315 TTTATTGAGATAATGAATAAACATAGATCCACATATGCTGTAACATTTGAAAGCCATCTTACCAGCAGCATCAGCGCCAGAAATTAATACTTCGCTGATACTGTCACCTGGAATAAGTAC 1237680 1237800

NC_009488_1_Orientia_tsutsugamushi_Boryong_complete_genome probe_cstm_Rprow_021218_1x_10316 GCAATCTTTCACCCAACTAGTAGTGTTGGATGTAACTTGTTGAACTGCTTTTGTAATAAAACTATAAACATCTCTCATTGCAATCACTCCGttaattaattaaaaatttatagaagtaat 1237800 1237920

NC_009488_1_Orientia_tsutsugamushi_Boryong_complete_genome probe_cstm_Rprow_021218_1x_1032 GATCAAGTGCGATAAGAATAGTATTTTCATAGGCTATAGTTATCAAATACAGCAAATACTACATCAGTTAATAGATAATGCTAATCGCTTTAATAAAAACAGCAAAAATAAAGAAGTAGT 123720 123840

NC_009488_1_Orientia_tsutsugamushi_Boryong_complete_genome probe_cstm_Rprow_021218_1x_1033 AATTACAGTAGAATTATTACCTCCTACAATTTCAGAAAATACAGAAAGAATACTACAATTTACTGTTCATGATAATGGACAAGGCATATCAAAAGAAACTCTACAACAAATCAATAATGA 123840 123960

NC_009488_1_Orientia_tsutsugamushi_Boryong_complete_genome probe_cstm_Rprow_021218_1x_1034 TTTTAGAGATATAAGTCAGAATAGTGAAGCATTAAGATTAAGCTTAACGTTTGTTAAGCTGATGCTTCAAGAAATACGAGGGGAAATTACTATGAAAAGTGAAGaaaaaaaGTATACGGA 123960 124080

NC_009488_1_Orientia_tsutsugamushi_Boryong_complete_genome probe_cstm_Rprow_021218_1x_10348 ACAATAGATTAATTCTAGGAAATAGAATTGACAAAAAGTATAATTAACATTAACTTTATAAAAATATTAAATTAGATAGATACTGATATGAGTATTTTAAAATCACTTTTACAACCGTGT 1241640 1241760

NC_009488_1_Orientia_tsutsugamushi_Boryong_complete_genome probe_cstm_Rprow_021218_1x_10349 TATGCTGTTATATTAGCATTCGTTGTATTATCTTCACCATCAATATGTACAGCGAATAATAATGTAAACTTCAATACTTCTAAAGTTAGTATTGATACTTCACAATTCTATATAAAATTT 1241760 1241880

NC_009488_1_Orientia_tsutsugamushi_Boryong_complete_genome probe_cstm_Rprow_021218_1x_1035 AGCCATATTTCATATACCAGTCAGCTTAGCTAATTGTTAGCTAAGCTTGCTATTTTAAATTTCTAAAAAACAAAGTTAATAAACTTAACCGTAGGAGAATAATGTGGAAAATAACAATAT 124080 124200

NC_009488_1_Orientia_tsutsugamushi_Boryong_complete_genome probe_cstm_Rprow_021218_1x_10350 TCAACTGGTATTGCTACTAACTACGATACATTAGAAGCTGGAATTGGTTATAGATTAGATCGGCACAGAATGGATATAAGATTAGGATTTATTGGGCATCACAACTGTAGAGATAACTTT 1241880 1242000

NC_009488_1_Orientia_tsutsugamushi_Boryong_complete_genome probe_cstm_Rprow_021218_1x_10351 GTTCAAGGAAATTATTATTACAATATAATTGAAGGTAACAAAGCATCAATTTTTGTTACAGGTGGCCTAGTATCAGTTTTCAATGGTGATAGTGGTACTGGTATAGATGTAGGAGTTGGT 1242000 1242120

NC_009488_1_Orientia_tsutsugamushi_Boryong_complete_genome probe_cstm_Rprow_021218_1x_10352 ACAACAATAAACTTATCAAGAGATACATATTTAGACATTGAATGTAGCACAATGGCTAACTATAGACCACTTCCTATTCATATACGTTTTGGATGGCGGGTTCACATTTAATAACTAATA 1242120 1242240

NC_009488_1_Orientia_tsutsugamushi_Boryong_complete_genome probe_cstm_Rprow_021218_1x_10353 AGTTTTATAGTTGGTAATTATTAGCCCATTAACGAGATTGCGTTGTTCATTAATTGAGTATGTAACTCCAATATGAGTGATGAAGAGGTCTTTGTATAAAGCTTTAATAAAGGTAGTGTT 1242240 1242360

NC_009488_1_Orientia_tsutsugamushi_Boryong_complete_genome probe_cstm_Rprow_021218_1x_10354 ATTATTTGAAAGCATAATTTTAACACCTTTATTGTTGAGTTCATAAACAAAATCTCGTAGTCTGATTTGCTCTTTTTCATCAAATGGGACTCTAGTGTAGAACCTTTCTCCTGATTGGTG 1242360 1242480

NC_009488_1_Orientia_tsutsugamushi_Boryong_complete_genome probe_cstm_Rprow_021218_1x_10355 GTAAGGAGGATCGAAATAAACAAAGTCATTTTGTTGAGGCCCTATAAATGAAAAATCCATTGCGCAAATTGATACATCGGTTAAAAGTTTACTACATTTGTTTATTCTAGAGCAAATATG 1242480 1242600

NC_009488_1_Orientia_tsutsugamushi_Boryong_complete_genome probe_cstm_Rprow_021218_1x_10356 AAGCTTAAGATATTACTTATCAGAAAATGTTTGAGCGGATGTGCCGTCTCTGTTTAGCCTATAAATTCCCCTAAAGGAATATTTATTAAGATATATAAATTTTGCAGTAATATCGTTAGG 1242600 1242720

NC_009488_1_Orientia_tsutsugamushi_Boryong_complete_genome probe_cstm_Rprow_021218_1x_10357 ATCATTACTATAATAATTGTCCCTTATTTTATAGTAATAATTTTCAGAATGGTTTTTGTGATATAGATTTAGTAATCTATTAACCTCATTTGGATTCTTTTTTACAGCGTTATAACTAGT 1242720 1242840

NC_009488_1_Orientia_tsutsugamushi_Boryong_complete_genome probe_cstm_Rprow_021218_1x_10358 AATTAAGTTGAGATTAATATCAGACAAAAAACATTGTTTAAACAGATGCCTAACTTGAAAAAATAAAGCCCCACCTCCAAGGAATGGTTCATAGTAATTATAGTATGGCCCTTGAAGGAA 1242840 1242960

NC_009488_1_Orientia_tsutsugamushi_Boryong_complete_genome probe_cstm_Rprow_021218_1x_10359 GATGCTCTATTAATTTATTAACAATTCTCCTTTTGCCTCCAACCCAGTGAAGAAAAGGCTTTGGTTCATTAGAAATTGCTATTGACACAAGAATTTTTGATTAATTAAATCTCTACTATG 1242960 1243080

NC_009488_1_Orientia_tsutsugamushi_Boryong_complete_genome probe_cstm_Rprow_021218_1x_10360 AATTATAGTAGAAAATAAGCCTTAAAGCTAGTAATAATAACTGCTGCAGAGCtttttttATAAATCTTTTTTGAATGATGTTTTTGAAGCTAGAGATACAATGTGATAATTTCGCATCTA 1243080 1243200

NC_009488_1_Orientia_tsutsugamushi_Boryong_complete_genome probe_cstm_Rprow_021218_1x_10362 AGGTTATTTCATTAAAACTTCATAATCTGCTATTAAAGGTATAAATGAATCTCTAGCTACTCTTTAATCACTACTTCAAATAGCAAAAGTAATGCTATGGTTATTAATGGCCTAATTATT 1243320 1243440

NC_009488_1_Orientia_tsutsugamushi_Boryong_complete_genome probe_cstm_Rprow_021218_1x_10370 ATATACTAAATTATCAAAGTCTTTTTATAACTCAGAATCAATTGTATATCTCATTTTGAAAGATATTTTGttttttttAGATGTAAATAACAATGCGATAATTTCGCATCAATGTGTAAT 1244280 1244400

NC_009488_1_Orientia_tsutsugamushi_Boryong_complete_genome probe_cstm_Rprow_021218_1x_10371 ATTATGaaaaaaaaTTTCATAAATATGACAAAAAGTCATGTACTGACAAAAATCGAAATGATACTGTATACCTGATATTATCTGAGATTTAAGATTTAGATAATATTCCGATAGAGATTA 1244400 1244520

NC_009488_1_Orientia_tsutsugamushi_Boryong_complete_genome probe_cstm_Rprow_021218_1x_10372 AAAGCATATAAACAACTAAATTTATCTTTTTAATCTCTAGCATAACAGTTTTAACACTTAAAACAGCAGGGTAATTCTATGCCTATAGAAGACGAAGGTCAAAATAAAATCAATAAATTA 1244520 1244640

NC_009488_1_Orientia_tsutsugamushi_Boryong_complete_genome probe_cstm_Rprow_021218_1x_10373 ACTGAAAAATTATCTACAGAGGGAATTAATAGCACAACAATAAAACAGATAGATGCTGAAATGCAAAAACTATACTGCCAGCTAGAGGAATCTGAGGAAGTAAGCATAAATTGCATAGAG 1244640 1244760

NC_009488_1_Orientia_tsutsugamushi_Boryong_complete_genome probe_cstm_Rprow_021218_1x_10374 TGGATACAATATTTTAGGCTAATAGTAAATAACTACGACAGaaaaaaaGTAAATATAATAGCTTCTCTAAGTAGAGTGAttttttttATTTAGACAATACATAGCATCAACAAATGTAAG 1244760 1244880

NC_009488_1_Orientia_tsutsugamushi_Boryong_complete_genome probe_cstm_Rprow_021218_1x_10375 GATTGAAACATTTAACATAGAATTGCTAATAAATAATACCGTTATCAGAATGAGGAAACATTTTGAAAATGAGAATATAAAGCTAAATGTTCAAAATGACATAAAGACGGTTCTGATTGG 1244880 1245000

NC_009488_1_Orientia_tsutsugamushi_Boryong_complete_genome probe_cstm_Rprow_021218_1x_10376 AGATAGTTTTAGAATAAAAGCAGTAATAAGTCAGTTAATTGGTAGTGCTATTATAAATAGTAGTAAGCATAGCAAGATTACTATTAACATCAATCAGTATTCAGAAATATTACAATTTAC 1245000 1245120

NC_009488_1_Orientia_tsutsugamushi_Boryong_complete_genome probe_cstm_Rprow_021218_1x_10377 AGTACAAAATATAGTACTAAGCCCTTCTAAAGAAAAATTAGAAAGAATAAATTCCGAACTAGAGAATTTGAATTTGGTAACATATCAAGAACTAGGAGAAGGATTAGCATTTATTAAACA 1245120 1245240

NC_009488_1_Orientia_tsutsugamushi_Boryong_complete_genome probe_cstm_Rprow_021218_1x_10378 TCTTATACATCAGCTAAAAGGAAGGCTAAGAGCAAAAGAGGAAAATAATTACATAACTTTTGCATTTGAAGTTCAAATAACTAGTTTTAACTCTTCTTTTGGCGAATGTTATGaaaaaaa 1245240 1245360

NC_009488_1_Orientia_tsutsugamushi_Boryong_complete_genome probe_cstm_Rprow_021218_1x_10379 aaTAAGTAACCAATGGATAGTACAAATCCCTTCAATGCTAATTACGTTGGTAAATGGAGAAAGGGAGAATATCGATGAATATATAGAGATAATAACAAAGTTAAGAAAAGCTAAGTATCA 1245360 1245480

NC_009488_1_Orientia_tsutsugamushi_Boryong_complete_genome probe_cstm_Rprow_021218_1x_10380 GGTCGAGGTAGCTGAATCATTGAAAGAATATTGTGTGAATCTGATACAGAATATCAAATATAGATTTAATATTGCAACGAGTGAAATTGTAAGGCTTGCAAGCGAGATCATGTTAAATGA 1245480 1245600

NC_009488_1_Orientia_tsutsugamushi_Boryong_complete_genome probe_cstm_Rprow_021218_1x_10381 TTCTGAAAATAAAGATAAGCTGAAAACAATATTAAATCGATCAGCAAATCTCCAAGAGTACTGTAACGATGTAGTTTACACGCTTAGAAGCGAAATTGAGAATGAAAATTTATGTTTaaa 1245600 1245720

NC_009488_1_Orientia_tsutsugamushi_Boryong_complete_genome probe_cstm_Rprow_021218_1x_10384 CGATAACATACTACAATTTAGAATACACGATAAAGGAAGCGGTATTTCAAAAGAAAAATTAGGGAATATAAAAGCTAAATTAGCTGATTTTGACTTGGTAAGAGACTGTCCGCTAATGCT 1245960 1246080

NC_009488_1_Orientia_tsutsugamushi_Boryong_complete_genome probe_cstm_Rprow_021218_1x_10385 TGAATCAGGATTATGGTTTGTAAATTACCTTATTAATCAACTTAATGGAGAAATGGAAATAGAGAGTGAAAAAGACAAGTTTACAACCATTACTTGCAATATTCCAGTACAACTTTTTTA 1246080 1246200

NC_009488_1_Orientia_tsutsugamushi_Boryong_complete_genome probe_cstm_Rprow_021218_1x_10386 ATCAAAAATAACACATTTTAATGACTTAAAAATTCACAATTTATTTTGCTTCTTTCCTAGATTTTGTCTGAGATTGAAGTATATAAAAAGTTTAAATTATGGTATAATTTATTAAAGATT 1246200 1246320

NC_009488_1_Orientia_tsutsugamushi_Boryong_complete_genome probe_cstm_Rprow_021218_1x_10387 ATTTGGAGATGGAAAATGATAGACTTTTATAGCGAGAGCTTACTAAATAAGCTGTTTAGAATCAACGTAAGATTTAACACTAAAATTGATCTTGATAAAGTTGAAAAAGCTATATTTTAT 1246320 1246440

NC_009488_1_Orientia_tsutsugamushi_Boryong_complete_genome probe_cstm_Rprow_021218_1x_10388 GCCCAAAAATATCATGGTCAGCAAAAGCGAGATACTGGAGAACTATACTACACACATCAATTAGAAGTAGCTTATATGGTTGCAGACTACAGCTTTGAAACTGATACGATTATTACAGCA 1246440 1246560

NC_009488_1_Orientia_tsutsugamushi_Boryong_complete_genome probe_cstm_Rprow_021218_1x_10389 ATACTACATGATACACTCGAAGACACAACACTAACCAAAGAAATAATAAGTCAAGAATTTGGTAATAATATTGCAGAACAGGTTTTAGACCTCACCAGGATTAAGGATAATaaaaaaaTC 1246560 1246680

NC_009488_1_Orientia_tsutsugamushi_Boryong_complete_genome probe_cstm_Rprow_021218_1x_10390 AGTTCTAGAGAAATGATTCAAACATTTTATAGGCAAAATAAAATAGAACTATTATTAATTAAGCTTTTCGATCGATTCCATAATATTCAAACCATACATATAAAACCTTATGaaaaaaga 1246680 1246800

NC_009488_1_Orientia_tsutsugamushi_Boryong_complete_genome probe_cstm_Rprow_021218_1x_10391 caaaaaaaTACTCATTGAAACTCAGCAAGAATTTATACCTCTTGCTCAATACCTTAATCTACCAAAGATTGCTGACAAATTAAGTGGATACTGTTTACTTAATACTTATCTGGAATAAAT 1246800 1246920

NC_009488_1_Orientia_tsutsugamushi_Boryong_complete_genome probe_cstm_Rprow_021218_1x_10392 GCTAATTCAAAAGAACAACAATATTTTATTTTTAAATAAAATAGCTGGTTCaaaaaaaaGTAGCAttttttttATTTTGAACATCTGATTCTCTGTACCTTGATTCTAAAAATTAATCAG 1246920 1247040

NC_009488_1_Orientia_tsutsugamushi_Boryong_complete_genome probe_cstm_Rprow_021218_1x_10393 TTATGATGCTGAGATTGCtttttttaattttttttGCAAGTTCAGTTGCTAGCTGGTATGCATACACATTATCAGAATCATATTTAATAGCCAGGTCATAACTTTCCATTGCTTCTTGAT 1247040 1247160

NC_009488_1_Orientia_tsutsugamushi_Boryong_complete_genome probe_cstm_Rprow_021218_1x_10394 GTCGCCCCATTCCTTCAAAGCAATTCCTTTATTATAGTAAGATTCTGCATCAGTTGGGTTATATCTAACAAGCTGCATCAAAATTCTCTATTGTTTCTTGATGTTGTCCTAATTCCTTCA 1247160 1247280

NC_009488_1_Orientia_tsutsugamushi_Boryong_complete_genome probe_cstm_Rprow_021218_1x_10395 AAGACATTACTTCATTATAAGCATCTGCATCATTTGGGCTGTATTTAATAGCTGTATCAAAATTCTCTATTGCTTCTAGTAGCTTTCCAAGATTCTTTAAAGCTATTCCTTTATTATAGT 1247280 1247400

NC_009488_1_Orientia_tsutsugamushi_Boryong_complete_genome probe_cstm_Rprow_021218_1x_10396 AAGCTGTTGCAAAATCAGGTTTATATCTAATAGCGATATCAAAATTCTCCATTGCCTCTTGATGTTTACCTAATCTGCCTAAAGCAGTTCCTTTATTATAGTAAGCGTTTACAAAATCAG 1247400 1247520

NC_009488_1_Orientia_tsutsugamushi_Boryong_complete_genome probe_cstm_Rprow_021218_1x_10397 GTTTATATCTAATAGCGGTATCAAAATTCTCTATTGCCTCTTGATGTTTACCTAACTTTTCTAAAGTAGTTCCTTTATTATAGTAAGCTGTTGCAAAATCAGGTTTATATCTAATAGCTG 1247520 1247640

NC_009488_1_Orientia_tsutsugamushi_Boryong_complete_genome probe_cstm_Rprow_021218_1x_10398 TATCAAAATTCTCTATTGCCTCTTGATGTTTACCTAATTCATCTAAAGCTATTCCTTTATTAATATAAGCTGCTATAAAATCAGGTTTATATCTAATAGCGGTATCAAAATTCTCTATTG 1247640 1247760

NC_009488_1_Orientia_tsutsugamushi_Boryong_complete_genome probe_cstm_Rprow_021218_1x_10399 CCTCTTGATGTTTATCTAATTCGTCTAAAGCATTTCCTTTATTATAGTAAGCTGTTGCAAAATCAGGTTTATACCGAATAGCAGTATCATAATTTTTTATTGCCTCTTGATGTTTACCTA 1247760 1247880

NC_009488_1_Orientia_tsutsugamushi_Boryong_complete_genome probe_cstm_Rprow_021218_1x_104 TATTAGTTCATAATACTATACTTAATAACCTGAACATTGACTATATTGTAACACTTGTATATTATCTCTTTCTAACTGTTTCTTATTCTAAAATTGTGTTTGCAGATATGTGAGCAATAA 12360 12480

NC_009488_1_Orientia_tsutsugamushi_Boryong_complete_genome probe_cstm_Rprow_021218_1x_10400 ATTCATCTAAAGCATTTCCTTTATTATAGTAAGCTGTTGCAAAATCAGGTTTATATCTAATAGCTGTATCAAAATTCTCTATTGCCTCTTGATGTTTACCTAATCTGCCTAAAGCTATTC 1247880 1248000

NC_009488_1_Orientia_tsutsugamushi_Boryong_complete_genome probe_cstm_Rprow_021218_1x_10401 CTTTATTATAGTAAGCTGTTGCAAAATCAGGTTTATATCTAATAGCGGTATCAAAATTCTCTATTGCCTCTTGATGTTTACCTAATCTGCCTAAAGCTATTCCTTTATTAATATAAGCTG 1248000 1248120

NC_009488_1_Orientia_tsutsugamushi_Boryong_complete_genome probe_cstm_Rprow_021218_1x_10402 CTATAAAATCAGGTTCATATCTAATAGCAGTATCATAATTTTCAATTGCCTTTTGATATTGACCTAACTCTTGAAAAGTTATTCCTTTAGCATAATAACCTTCTGCCAAATCTGGTTTAT 1248120 1248240

NC_009488_1_Orientia_tsutsugamushi_Boryong_complete_genome probe_cstm_Rprow_021218_1x_10403 ATCGAATAGTAGTATCAAAAATTTCTATTGCCTTTTGATGTTGTCCTAATGCCTTTAAAGCCATTCCTTTATTAATATAAGCTTCTATACAATCTGGATTACACTTAATAGCTATATCAA 1248240 1248360

NC_009488_1_Orientia_tsutsugamushi_Boryong_complete_genome probe_cstm_Rprow_021218_1x_10404 AATTCTTTATTGCTTTTTGATATTTTCTTAACTGAAAAAATGAATTCCCTTTATTAAAATATTTGTCTGCTAGCATATCTTTACCCTAAATTGAATACTGTTATATGAGCAGATTATTTT 1248360 1248480

NC_009488_1_Orientia_tsutsugamushi_Boryong_complete_genome probe_cstm_Rprow_021218_1x_10405 GATGCAATAGATGTAACTGTATTAATAATACTGTCATATATTTCATATGATTTATAAAAATTCCCCTTTAAATTTTTGATTAGCTATGATAATTGCTAAATCAAATGTGCTGTTATCAAa 1248480 1248600

NC_000963_1_Rickettsia_prowazekii_str__Madrid_E_chromosome_complete_genome probe_cstm_Rprow_021218_1x_104054 TGATAAATACAATAAGCTTATAGAATATGAAAAAACTTCAGCGTAATTTTTTGAGAGTACAATATAAATTGAGCACTTAAATTAGAACAGAAACAAGAACTATAAATTTCTTTATCAAAT 576000 576120

NC_000963_1_Rickettsia_prowazekii_str__Madrid_E_chromosome_complete_genome probe_cstm_Rprow_021218_1x_104055 TATAACTTGAATAAGAGTGAAGCACTAATTATAGATTTGATATTTTAAATGATAAGAATTCTTACTTAGTCAAAAAGAAGAAAATGAGAAACTGTAACTTTAGTAAGTAATGATGTAAGG 576120 576240

NC_000963_1_Rickettsia_prowazekii_str__Madrid_E_chromosome_complete_genome probe_cstm_Rprow_021218_1x_104056 TAAATAGTGGAGACAGATATTACCTATTTCAGAAATTATTAAATATCAGCCTCATTGATTTTATCTTAAGTTAAGGGTCGCTATAACTAATAAAATTGCTCGAAGATCTTTTTAAATGCT 576240 576360

NC_000963_1_Rickettsia_prowazekii_str__Madrid_E_chromosome_complete_genome probe_cstm_Rprow_021218_1x_104058 ACACTATCTTATACAAAATTCAGGATGTTAATGACATTTAATTTTATAACATTCATTTGTATTAAGTTAGGTATGAAATGCTTCTTTAACTCGATAAAAATATTGTTTGTTTCttttttt 576480 576600

NC_000963_1_Rickettsia_prowazekii_str__Madrid_E_chromosome_complete_genome probe_cstm_Rprow_021218_1x_104059 CTCGTTATTATTATTTGCATAGAAAATTTCTATAATTTAACTCTATGCCAAATATAGATCTCCTCATTTTTACTAGCTTTTTAGTAATTAATTTACTATTTGGACTTCTAAACATCAAAA 576600 576720

NC_000963_1_Rickettsia_prowazekii_str__Madrid_E_chromosome_complete_genome probe_cstm_Rprow_021218_1x_104060 ATATAAAAAATAATCGTGAATATGCTGTAGGTGAAAGGAATTTTTCTACAGGCACAATAGTCGCAACTCTGATTGCAACTTGGATCGGGACTAGTACTTTTTTAATAGATAATTCAATAA 576720 576840

NC_000963_1_Rickettsia_prowazekii_str__Madrid_E_chromosome_complete_genome probe_cstm_Rprow_021218_1x_104061 TATATCCAGATGGATTATTTTACTTATTACCGAGTATATTTGGTAGCGTTGTGAGTTGGTTATTAATGAGTTACTTTATAGCACCTCGTTTTGAGAATTTTTTAGGTAGTTTATCGGTAG 576840 576960

NC_000963_1_Rickettsia_prowazekii_str__Madrid_E_chromosome_complete_genome probe_cstm_Rprow_021218_1x_104062 CAGAAATAATAGGTGCAGCGTATGGTAACAAAGTGCGTATTCTTACTTCAATAGTTAGTATATTAATGTCTATAGGTAGGATTGCTATGCAATTCCACGTAGCAGGTTTAATATTACAGC 576960 577080

NC_000963_1_Rickettsia_prowazekii_str__Madrid_E_chromosome_complete_genome probe_cstm_Rprow_021218_1x_104063 TATTCTTTAATATTTCAAATTTTTATATAAATTTATGTATCGCAACTGTAATAATAAGTTATTCTGCTTTTGGTGGCGTAAAATCAGTTACTTTCACTGAAGCCGTACAGTTTTTTACAT 577080 577200

NC_000963_1_Rickettsia_prowazekii_str__Madrid_E_chromosome_complete_genome probe_cstm_Rprow_021218_1x_104064 ATAGTGCAATTATTCCGGTAATAGGAATCATTATATGGAATGTTTTTAGTGATCCGTATATAGTGATCAACTCAAATGTACAGAATCATTTAATTGATTTACAAGGATTATTTCATTGTA 577200 577320

NC_000963_1_Rickettsia_prowazekii_str__Madrid_E_chromosome_complete_genome probe_cstm_Rprow_021218_1x_104065 CTAGTCCTAAATTTTGGATTGCTTTAAATATTTTTATATATTTTGCTATTCCATCGTTTGGTCCATCAATATTTCAAAGAATATTGATGGCAAAAGATACAAAGCAAATCTCACATACAT 577320 577440

NC_000963_1_Rickettsia_prowazekii_str__Madrid_E_chromosome_complete_genome probe_cstm_Rprow_021218_1x_104066 TTTTTATTGCTGCAGTTATTTGTCTTTGTTGGTCCATTTTATTCATTTGGATTACAATATTATTATTTTCTCAAAATTCTGTAATTGAAACAAATGAGCTGTTTTTACGAATCATTAAAA 577440 577560

NC_000963_1_Rickettsia_prowazekii_str__Madrid_E_chromosome_complete_genome probe_cstm_Rprow_021218_1x_104067 GTTATATAGGATTTAAGGGGTTAATAGTTGGAGTTGTTATGGCAATGATAATTGCAAGTACTAACTCTGATATTAATGTTGCTGCGGTAACTTTTACTAATGATATAATTAAAAAGGCTT 577560 577680

NC_000963_1_Rickettsia_prowazekii_str__Madrid_E_chromosome_complete_genome probe_cstm_Rprow_021218_1x_104068 CATTGCATTATAATTATATAACCGCAATTATTATAGGATTAATCGGTTTTATTATATCACTCTATACGCAAGATCTAATAAATCTGTTTTTACTATTAACTAGTATCTATTTGCCGTTAA 577680 577800

NC_000963_1_Rickettsia_prowazekii_str__Madrid_E_chromosome_complete_genome probe_cstm_Rprow_021218_1x_104069 TTACTTTACCGTTAATTTTATTAATATATGGTTTTAAATCTACTACTAAAAGtttttttATCAGCACTATTGCTGCAATATTCACTATTATAGCATGGCAAATATTTTCTTTAAGGACGG 577800 577920

NC_009488_1_Orientia_tsutsugamushi_Boryong_complete_genome probe_cstm_Rprow_021218_1x_10407 TAGTGATATTGCTAGCTGCGCATATAGTTTGAACTATTATCTAAGACACAAGCTCTAGTTTGCACTGAACATTGAGAAGGCAATTCTTTAAGGCCTCAATGTCCTTAACATTGCCGCACC 1248720 1248840

NC_000963_1_Rickettsia_prowazekii_str__Madrid_E_chromosome_complete_genome probe_cstm_Rprow_021218_1x_104070 TTTTAGGCATTTATCCTATACTACCAGCAACTATTGTAACTTTAATCTTTTTTCTTGGAAGACATTATATTTTAAAGCAGCCTGGGGGATTTATTGCTTTAAAAGAGCCACTATCAGTGA 577920 578040

NC_000963_1_Rickettsia_prowazekii_str__Madrid_E_chromosome_complete_genome probe_cstm_Rprow_021218_1x_104071 AAATAATCAGGCAAGAGAGAAAAAGAAAATTTTTAACTTTATTTCGTGATATTAAGAATTTTAACATATTTAAATATTGGAAAAGTACCTTACCAAATCAAGAAATTACTTATAGTCATG 578040 578160

NC_000963_1_Rickettsia_prowazekii_str__Madrid_E_chromosome_complete_genome probe_cstm_Rprow_021218_1x_104072 TTGGAATATTTATTTTAATATCAATGTTCTCATCTTTATATACTATATCAGGTAGCAGAATAATGACCCATTTTGCAATATATAATATTATTTATCATACTACACTTATAGCTGCTTCAA 578160 578280

NC_000963_1_Rickettsia_prowazekii_str__Madrid_E_chromosome_complete_genome probe_cstm_Rprow_021218_1x_104073 TATTAATAACTTATCCTTTATGGTCTTTAAAATTCaaaaaaaaTTATATTATATCTTTCTCTTGGTTTATCGCTACTTTTGTTATATTAATATTTTTTGGTAGTTTACTTGTTATATTAA 578280 578400

NC_000963_1_Rickettsia_prowazekii_str__Madrid_E_chromosome_complete_genome probe_cstm_Rprow_021218_1x_104074 GTAATTTTGATCAGTTACAATTAATGTGTTTAGTAATTAACTCACTTATAATTGCAACAATATTTAGGTGGCAAGCTACTTTACTTATGACTGTTACAGGCGTGTTTGCTAGTATAGAAT 578400 578520

NC_000963_1_Rickettsia_prowazekii_str__Madrid_E_chromosome_complete_genome probe_cstm_Rprow_021218_1x_104075 TTTATAAATATTTTATAGATGAGAAGTTATTAATTAGTATAGATAGCTTTCAATTTAAAGTAGTATATTTCTTAATCTTATTTAGTAGCCTTTTAATAGTATTTTTAAAACCAAGACAAG 578520 578640

NC_000963_1_Rickettsia_prowazekii_str__Madrid_E_chromosome_complete_genome probe_cstm_Rprow_021218_1x_104076 ATCAAGAAATATTAATTAATCTGAAAAATACTCATTTAGGTAATAGAGTTAATTATAGAGAACAAGAGTTAGAGAAACTACTTGATTTAAAACATGAGTTCTTACGTAATATAAATCATG 578640 578760

NC_000963_1_Rickettsia_prowazekii_str__Madrid_E_chromosome_complete_genome probe_cstm_Rprow_021218_1x_104077 AAATCAATACACCTCTAACAGGTATTATTAGTCTTGGTGAAACATTATGGGCTAATTATGATAAGTTTAATGAGGATCAGCGTCGCAATGCTGTAGAGGTTATAGCTAAAAGCTCTATTA 578760 578880

NC_000963_1_Rickettsia_prowazekii_str__Madrid_E_chromosome_complete_genome probe_cstm_Rprow_021218_1x_104078 GATTGAATAGCTTTATAAATAATATTCTAGATTTTTCAAAATTATCCAGTCTTAATTATGAATTAAATAAAAATTATATCAACTTAAGCGAATTGTTATATGAAAGGATTAAAATATGTA 578880 579000

NC_000963_1_Rickettsia_prowazekii_str__Madrid_E_chromosome_complete_genome probe_cstm_Rprow_021218_1x_104079 AAAAATTATATCTAAACGGCAAAACCTTAAATTTTGTATTTGATATAGAAGAAAATATAATATTTAACTGTGATCCGCATTATATTAAACACACATTCGATAATCTCATAATCAATGCTA 579000 579120

NC_000963_1_Rickettsia_prowazekii_str__Madrid_E_chromosome_complete_genome probe_cstm_Rprow_021218_1x_104080 TTAGCTATTGTAGTGAAGGAACAATAAAAATTGATTTACAAAAACAAACAGATAGTATTTTATTCATGATAAAAGACGATGGGATAGGAGTACCAAAGGAAGAATTGCCAAATATATTCG 579120 579240

NC_000963_1_Rickettsia_prowazekii_str__Madrid_E_chromosome_complete_genome probe_cstm_Rprow_021218_1x_104081 GCGTATTTGTTGTAAGTTCTAAAACACGTACAAAAGCAGGAGGACGGGGAGTAGGGCTTGCCTTATGTAAAAAAGTTATAGAACTGCACTCTGGAAAAATTTGGGCAGAAAATGATAAGC 579240 579360

NC_000963_1_Rickettsia_prowazekii_str__Madrid_E_chromosome_complete_genome probe_cstm_Rprow_021218_1x_104082 AAGGGAAAACACATTTTATTGTTACTATGCCTTTGAATACTCCTTTGTAAGTTATGTACAACAATTTAGTTTTATTATTGCAAAAGGAATAGATTTAGTACTTGTTATTACTCCCTTTGT 579360 579480

NC_000963_1_Rickettsia_prowazekii_str__Madrid_E_chromosome_complete_genome probe_cstm_Rprow_021218_1x_104083 ACTATGCTACTCGTATAGTATTATTACGTGTCACTAGTGCAAAATATTGCAGTTTAAACCTCTTACAAATGATGAAACAATTAAAGAAATGAACATTGCAAGAAGTTGTGAGTTAGTTCA 579480 579600

NC_000963_1_Rickettsia_prowazekii_str__Madrid_E_chromosome_complete_genome probe_cstm_Rprow_021218_1x_104084 TATCGATAAAATTTGTTATAAATGAGAATGCTTAAATATACTGCGCAGTTTAAATGTGATTATATGCGTGAAATAAATATAAAACCTATATAGATGTGGATGATATTATTGAAAGCAGTT 579600 579720

NC_000963_1_Rickettsia_prowazekii_str__Madrid_E_chromosome_complete_genome probe_cstm_Rprow_021218_1x_104085 ATTTACATATGATCAGATATCATAATACTCTATTGTTAATAGAAAGATTGTAGAGACCTGCATATTAACTCTGTGATTAATTGAAAATACAATACTAATATATTAGATATTAGCAGCATA 579720 579840

NC_000963_1_Rickettsia_prowazekii_str__Madrid_E_chromosome_complete_genome probe_cstm_Rprow_021218_1x_104086 TATTGGACTTAATTTCAAATTTATGTTTTTGATTAGTGAACATTTGCAATGCGTAAATCCTTATTTTATTTTTAACAAACAATAAAATTTTATATCAATAATAGCATTAAATCAAGTTAT 579840 579960

NC_000963_1_Rickettsia_prowazekii_str__Madrid_E_chromosome_complete_genome probe_cstm_Rprow_021218_1x_104087 CATGATGCATAAATTAAAGCCAAAAATTCACTTTTCCGTGTTTTAAAACTTAAGTATATAATAAAACCATGTCATGGATTGACTTTTTTATGATCCGAAAAAGGAACGTCTAAAAAATAT 579960 580080

NC_000963_1_Rickettsia_prowazekii_str__Madrid_E_chromosome_complete_genome probe_cstm_Rprow_021218_1x_104088 TTAAAGAGGGCATATACATACTAAAATCATTTTTTGGTTATATATAGCTACAGTAAATTTGTTAAATTGTTTTAATATAGAAAAAAGTTGATAAAATAGTGAAAGATTATAAAAGATATT 580080 580200

NC_000963_1_Rickettsia_prowazekii_str__Madrid_E_chromosome_complete_genome probe_cstm_Rprow_021218_1x_104089 AAATCTTCTTATTATTATTAAAGCAAAAGCAAATATAACATTTAAAATTACAATTTTATAGTCACTTAATGGAGTTTATCAAAGAGTAATAGATGTTAATAAAAAACTATGCAGCACATT 580200 580320

NC_009488_1_Orientia_tsutsugamushi_Boryong_complete_genome probe_cstm_Rprow_021218_1x_10409 AAATATCTTTACCGATTGTAGTCCAATGATTCTGAACATTTGGATGTTGTATTAAAGTTTCTTTTAGTGAAACAATTGCACCAAATAAAGTATTTGTTGGTAAGTGATCAAGTTTAGCCT 1248960 1249080

NC_000963_1_Rickettsia_prowazekii_str__Madrid_E_chromosome_complete_genome probe_cstm_Rprow_021218_1x_104090 ATAATCTAGTGCAATAAAAATGTCGGTATATTTTAGTACGTAAGAATTGTCCGTGATTTTACTATTGTAGAATACACTAAAAAATTTTTATTTAATTATGTTCAATTCCATATATAGAGT 580320 580440

NC_000963_1_Rickettsia_prowazekii_str__Madrid_E_chromosome_complete_genome probe_cstm_Rprow_021218_1x_104091 TTATAAGGCTAATGAAATAGTATATGGGATTAATTATAAATATTAATGTTTGAAATTTTACTAGCCATGTTAAAATAGTAGAAAACACATGATAAATTCTATCAGAATAGGTACAAGAAA 580440 580560

NC_000963_1_Rickettsia_prowazekii_str__Madrid_E_chromosome_complete_genome probe_cstm_Rprow_021218_1x_104092 TAGTACACTTGCTTTAATACAAACTAATTTAGTTATTGAGCAAATCAAACAAATTTTTCCTGATATTAATTGTGAAATAGTACCTATTATTACTAGCGGTGACTTAATTCAAAATAAACC 580560 580680

NC_000963_1_Rickettsia_prowazekii_str__Madrid_E_chromosome_complete_genome probe_cstm_Rprow_021218_1x_104093 ATTATACGATATAGGTGGTAAGGCTTTGTTCTTAAAGGAAATAGAGCAAGCATTGCTTGACaaaaaaaTTGACTTAGCTGTTCATTCTTTGAAAGATATACCTGGTAAAATACCTGTAGA 580680 580800

NC_000963_1_Rickettsia_prowazekii_str__Madrid_E_chromosome_complete_genome probe_cstm_Rprow_021218_1x_104094 GCTAGTAATAGCTGCAGTTTTAGAGCGTGAAGATCCAAGAGATGTTTTGGTATGTTTAAATTATCAATCTATAGAAACACTTCCGCAAAATGCCGTAATAGGTAGCTCGGCAGTGCGAAG 580800 580920

NC_000963_1_Rickettsia_prowazekii_str__Madrid_E_chromosome_complete_genome probe_cstm_Rprow_021218_1x_104095 AAAAGCATTTATaaaaaaaaTAAGACCTGATTTAAATATAAAAGTTTTTAGAGGTAATGTTGATTCCAGAATAAAAAAGCTAATGACAGGTGAAGTTGATGCAATAATTTTATCATATGC 580920 581040

NC_000963_1_Rickettsia_prowazekii_str__Madrid_E_chromosome_complete_genome probe_cstm_Rprow_021218_1x_104096 AGGACTGAAAAGACTTAATGTGTTTAATCAGAAATATTGTCATCTTATAGAGTATTCGAAGATGTTACCGTGTATAGGGCAGGGTGTAATTGCAGTCGAGATCAGAAAAGACGATAATGC 581040 581160

NC_000963_1_Rickettsia_prowazekii_str__Madrid_E_chromosome_complete_genome probe_cstm_Rprow_021218_1x_104097 TATGTTTAATATATGTAGTCAAATTAATCATCTTCCAACCTTTGAATTAATAAAACCTGAAAGAGCATTTTTAGAATATCTAGATGCTAATTGCAGTACACCGATTGCTGCTTACGCACA 581160 581280

NC_000963_1_Rickettsia_prowazekii_str__Madrid_E_chromosome_complete_genome probe_cstm_Rprow_021218_1x_104098 ATACCTAGATGCGTATAATATACAAATAGATTTTATGCTTGGGAACCTTGATTGTACTAAAATAATATTCCAAACTGAAATTACCAATATCAAAACTTCAAAAATATGTGGCATCAAAGC 581280 581400

NC_000963_1_Rickettsia_prowazekii_str__Madrid_E_chromosome_complete_genome probe_cstm_Rprow_021218_1x_104099 TGCTAAAATGATGTTGGCACAACAATAAATAAATTAAACCGATTAAATGAGAATATAGTAAGTTTATCAAAATATAAATTTTAATAGTAATTATTATCAACATGCCAATTCATATACAAA 581400 581520

NC_009488_1_Orientia_tsutsugamushi_Boryong_complete_genome probe_cstm_Rprow_021218_1x_10410 TAGCAACTAGTCCAGCCATTTCGATAAGACGTCTAGTACGCATTTTACGTTCTTTAATTTTAAGGCTAACCTCATCCATGATTAGCTTAGCCtttttttGTTGGAGAGTAATTTTTTGCT 1249080 1249200

NC_000963_1_Rickettsia_prowazekii_str__Madrid_E_chromosome_complete_genome probe_cstm_Rprow_021218_1x_104100 ATGATATGTGTGTGATAACAAAATACTCAATTTATATAAAGATTTATTAAGTGAAACAAATAATAAATAGTTTTAAATATTAAAGTAGAGGTATATGTGCATGCTGACCACACGTGTTTA 581520 581640

NC_000963_1_Rickettsia_prowazekii_str__Madrid_E_chromosome_complete_genome probe_cstm_Rprow_021218_1x_104101 TTTCAATAACAAAATTCAGAAAGGTACATATGTGATTTATCTATAATTCAAATATTGGATTAATAAAAAATATTCAGTGGTGTGTAAAACAACTAAATAAATGATTCCTTATCAAGGATA 581640 581760

NC_000963_1_Rickettsia_prowazekii_str__Madrid_E_chromosome_complete_genome probe_cstm_Rprow_021218_1x_104102 CAAATTTTCTAACTGTTCAAATTGGCGGATAGGGTGGGATTCGAACCCACGGTACAGTTGCCCATACGCCAGTTTTCAAGACTAGTGCCTTAAACCGCTCGGCCACCTATCCAAGGAAAT 581760 581880

NC_000963_1_Rickettsia_prowazekii_str__Madrid_E_chromosome_complete_genome probe_cstm_Rprow_021218_1x_104103 TCATACCAAATAATCTAATATACTTCAAGGAAAATCTTAAAAAGATAAATTTAAATTATAAATCCAAATTGtttttttACTTCATTTACTGTTTCAGATGCTCTAATCCTTGCTTTTTGT 581880 582000

NC_000963_1_Rickettsia_prowazekii_str__Madrid_E_chromosome_complete_genome probe_cstm_Rprow_021218_1x_104104 GCTCCTTTGTGCAGTATTTTCAATAAATATTCTTTATCATTCATTAATTCTAAACATTTATTACGTATTGGCTGTAGATTTGTAATAATAATTTCTGCTAGATCTTCTTTAAATTTTGCA 582000 582120

NC_000963_1_Rickettsia_prowazekii_str__Madrid_E_chromosome_complete_genome probe_cstm_Rprow_021218_1x_104105 AAACCTTGGTTTTGATAATTATCAATTATTTTTTCTATACTTTCTTTAGAGAAACTTTTATAAATATCTAATAGATTGCTTATTTCCGGTCTTGtttttttATTATAGCTAATAAAACTT 582120 582240

NC_000963_1_Rickettsia_prowazekii_str__Madrid_E_chromosome_complete_genome probe_cstm_Rprow_021218_1x_104106 AAGTGATCAGTTTTAGCtttttttattttttGATGAATAAGATCATTACTATCTTTTAAATTGATACGAGAAAAGTCAGATATATCTGATTTACTCATTTTCTTTAATCCATTCCGTAAG 582240 582360

NC_000963_1_Rickettsia_prowazekii_str__Madrid_E_chromosome_complete_genome probe_cstm_Rprow_021218_1x_104107 CTCATGATTCTTGTACCTGTTTCACTAATTAAAATATCCGGTACTTTTAGAATTTCTTTATCAAATCTCCTATTTATCACCTCTGCAATATCTCGTGTTAACTCTAAATGCTGCTTCTGA 582360 582480

NC_000963_1_Rickettsia_prowazekii_str__Madrid_E_chromosome_complete_genome probe_cstm_Rprow_021218_1x_104108 TCTTCACCTACAGGCACTATATCTGCTTTATAGATTAAAATATCAGCTGCCATAAGTATCGGGTATGCAAATAAACCAAGACACGCTTTACATTGAGCACTACCAGCTTTATCTTTAAAT 582480 582600

NC_000963_1_Rickettsia_prowazekii_str__Madrid_E_chromosome_complete_genome probe_cstm_Rprow_021218_1x_104109 TGTGTCATACGCTTTAACCATCCAAGTGGTGTAACACAATTAAGTAACCAACTCAGCTCTACATGCTCTTTTACCATGCTTTGAGCAAAAATTGTTACTTTGTCTGGATTAAGACCTGCT 582600 582720

NC_000963_1_Rickettsia_prowazekii_str__Madrid_E_chromosome_complete_genome probe_cstm_Rprow_021218_1x_104110 GCTAAATAAATAGCAAGTACTTCCATAATTGCATCATTAAGTTCTGATGTCTTAATATCAATCGTAATAGCATGCAAATCTGCTAAGAAGAAAAAACAATTATATTCTTCCTGCATTTTA 582720 582840

NC_000963_1_Rickettsia_prowazekii_str__Madrid_E_chromosome_complete_genome probe_cstm_Rprow_021218_1x_104113 ACCTGTAACATTTTGTAATGTCAACTATCAAATAAAATATAGAATTGCCATTATCTTTTCTTACGCTTTTGTGTGGCTTGCCAAAATTTGTTGTGGTTTAAAATATGAAGTAGAGGGACT 583080 583200

NC_000963_1_Rickettsia_prowazekii_str__Madrid_E_chromosome_complete_genome probe_cstm_Rprow_021218_1x_104114 TGAGAAATTACCGAAGACTACTTCCATAGTTATATCTAATCATCAGTCCTTTTGGGATCAAATGTTTATGCAATTAATTATTCCGAAACATTCTTGGGTATTGAAACGTGAATTATTTAA 583200 583320

NC_000963_1_Rickettsia_prowazekii_str__Madrid_E_chromosome_complete_genome probe_cstm_Rprow_021218_1x_104115 TATACCATTACTTGGTTGGGGACTCCGTATGGTTAAACCGATTGCAGTTGATCGCGGTACTAATAGTTCAGTTGCTCAGATTTTAAGAGAAGGACAAGCAAAAATAAAAGAAGGTTTATG 583320 583440

NC_000963_1_Rickettsia_prowazekii_str__Madrid_E_chromosome_complete_genome probe_cstm_Rprow_021218_1x_104116 GTTAATAATATTTCCAGAATCAACTAAAGTGCCCCCTGATAAAACAGTAAAATTTAAGCCAAGTGCTGTAAAGCTGGCATCTGTTACTAGGGTTCCGATTGTAATGATTGCACATAATGC 583440 583560

NC_000963_1_Rickettsia_prowazekii_str__Madrid_E_chromosome_complete_genome probe_cstm_Rprow_021218_1x_104117 GGGTTTATTTTGGCCTAGAGGTTTTTGGTTTCAGCAGCCTGGAACTATAAGGGTAAAGATTATAGGAGTAATAGACAAAGCAGAGGTAGAGCAAACGGATGTACGTATTTTGAATGAGAA 583560 583680

NC_000963_1_Rickettsia_prowazekii_str__Madrid_E_chromosome_complete_genome probe_cstm_Rprow_021218_1x_104119 AGGTACAGCACTGTTAAAATTAATTGCGGTTATTGATAGTAAGCACATGATGCTTTATGATGCGTTAGGGATTAAAATTACCACTAATAAACCTTTAAAACTCACTTTAGACTTAGAAGA 583800 583920

NC_000963_1_Rickettsia_prowazekii_str__Madrid_E_chromosome_complete_genome probe_cstm_Rprow_021218_1x_104120 ACATCACCATCATCGTGAAAAAAGACAAAGCCTTTACCAAAATAAATCTACTCCTGGCTCACTTTTTGAGCCACATACTTCGCTAAAAGATATAGAACATAAAGAAGCAGCAAGAAGTGT 583920 584040

NC_000963_1_Rickettsia_prowazekii_str__Madrid_E_chromosome_complete_genome probe_cstm_Rprow_021218_1x_104121 TATTAAACATTTAGAGAAAGTAACTACAGCTAATCAAGCTAAATATAAGGAATTGATTATTATAGCAGAACCGAAAATGCTTGGATGTGTTAGACAAGAGCTAACAAGTGGTTTAAAAAA 584040 584160

NC_000963_1_Rickettsia_prowazekii_str__Madrid_E_chromosome_complete_genome probe_cstm_Rprow_021218_1x_104122 GATTGTTACTAAAGAAATCGCTAAGGATTTAGTGCAACATAATGTGGATGCAGTTGAAAGAGCAGTATTTGCTTAGCTGAATTATTGCGTAAAACAGCAAAGAGTCCATCTTACTATTAA 584160 584280

NC_000963_1_Rickettsia_prowazekii_str__Madrid_E_chromosome_complete_genome probe_cstm_Rprow_021218_1x_104123 TTTTAGTAATTTAAATTGAGCTCTATTTCTCTAGTTACTGAAATAGTAAAAGTAATGCATCCAACAAGATTTATTCATGCGGTCTCTGTGTTGTTGAATCATAATTAAATGTCTTTGATA 584280 584400

NC_000963_1_Rickettsia_prowazekii_str__Madrid_E_chromosome_complete_genome probe_cstm_Rprow_021218_1x_104124 CTATTTAAGCAAAAGCATGAATTACAAATAGAGTGTATACTTTATTCTAATGTGTAGTATCTTATACAAATTTTACCATAATTAGTGATGCTATGTAAAAAGTTTAATGCCTAATGACTT 584400 584520

NC_000963_1_Rickettsia_prowazekii_str__Madrid_E_chromosome_complete_genome probe_cstm_Rprow_021218_1x_104126 GGAAGATATGCCAAAGCGACATCGCCTAATGCCCCTATTGCATTAGTACTTCATCCTCATCCTTTATATGAGGGTAATATGAATAATAAAGTAGTTTATAATGCTTATAAAATCTTAGTA 584640 584760

NC_000963_1_Rickettsia_prowazekii_str__Madrid_E_chromosome_complete_genome probe_cstm_Rprow_021218_1x_104127 GATAATGGTTATACCGTATTACGTATTAATTTTAGAGGAGTAGGAGGATCACAAGGTAAATTTGATAATGGAGTCGGTGAAGTTGTTGATGCGGGGGCAGCTCTCGACTGGTTACAACAA 584760 584880

NC_000963_1_Rickettsia_prowazekii_str__Madrid_E_chromosome_complete_genome probe_cstm_Rprow_021218_1x_104128 AATAATCCAAATGCTCAGTCTAACCTAATATTAGGATTTTCATTTGGAGCATGGATAGCTATGCAACTTGTAATGCGACGTCCAGAAATAAATCATTTTCTGGCCATTTCACCACCAGTT 584880 585000

NC_000963_1_Rickettsia_prowazekii_str__Madrid_E_chromosome_complete_genome probe_cstm_Rprow_021218_1x_104129 AATACAATTCATAAATATGATTTTTCATTTCTTGCACCTTGTCCTATTCCAGGTTTTATATTACAGGGAGATAATGATAGTATAGTATCTGCAGATGATGTAAAAGATTTAGTAAATAGA 585000 585120

NC_000963_1_Rickettsia_prowazekii_str__Madrid_E_chromosome_complete_genome probe_cstm_Rprow_021218_1x_104130 TTATCTAATCAACAATCTCATATTAAAATTGATTATAAAATTATCAATGGTGCAGACCATTTTTTCCGTTATAAAACTGAAGAATTTTCTAAAGCAATAAATGATTATTTAATAACAATC 585120 585240

NC_000963_1_Rickettsia_prowazekii_str__Madrid_E_chromosome_complete_genome probe_cstm_Rprow_021218_1x_104131 CAATCTAATTATCACCACCATAATAATATAAATGAAGACAAGAGTaaaaatcaaaaaaaaTTATTTTTATATTAGTCTTTGTTATGTGATTAAAGTATATCTATGCTATACTTTTAATGT 585240 585360

NC_000963_1_Rickettsia_prowazekii_str__Madrid_E_chromosome_complete_genome probe_cstm_Rprow_021218_1x_104132 CCTTACAAATTTACTTGTGTGGTTCAATATCTACAGTTATCATCTTGTATCTTGGATTGATTCAAGGGACATAATAGAATGAATGTCACAATTCTAAATAAGCTTCTATTGCCAAAATAA 585360 585480

NC_000963_1_Rickettsia_prowazekii_str__Madrid_E_chromosome_complete_genome probe_cstm_Rprow_021218_1x_104133 ATATTTTATCTTTTTGCGTATCTAACACTTGAGTTAATTCTTCATAAATTTGCTTATCTGATAGAGAAGTGAtttttttAAATAGCTGCACTAATCTTAAAGAAGGTGCAATACGGAAAG 585480 585600

NC_000963_1_Rickettsia_prowazekii_str__Madrid_E_chromosome_complete_genome probe_cstm_Rprow_021218_1x_104134 CTTCTTTTAATATTTTAGTCTTTTGATTAACACTTAGCTTATCATTTAAATTAAAATAAAATTCTAGTAATTCGTCATTATAAAAATTTAAATCTATAGCTTTTTCCAAATAATCTATAG 585600 585720

NC_000963_1_Rickettsia_prowazekii_str__Madrid_E_chromosome_complete_genome probe_cstm_Rprow_021218_1x_104135 CATTAGCCGTATTATTATTTTCCACTTCTTGCTTCGCTATTAATAAATAATACTGAGTGATTTTTGGTACAAATTCATGTTTATGAAATTTTGCTAATTTATTCGTTATAAAAATAAATT 585720 585840

NC_000963_1_Rickettsia_prowazekii_str__Madrid_E_chromosome_complete_genome probe_cstm_Rprow_021218_1x_104136 TTGACCATAAAGAAAGTTTAGCATAACAATGCATTAAGGTAATTAAATTGTCACTATCAAGTTCATTTAAATTATAAGCCTTTATCGCATAATTTTCTGCTTTATCATACAAAGATTTAT 585840 585960

NC_000963_1_Rickettsia_prowazekii_str__Madrid_E_chromosome_complete_genome probe_cstm_Rprow_021218_1x_104137 CATAATAAAGTTTTGCTAGGTTTTTGCTAGCGTAAAAAACAAATGTTTTAGACGTAATAAGTTTTTGAAAATAAGAAATTTTGCTATCGATATCCTCTGCAGTTACAGCTAAGATAAAAT 585960 586080

NC_000963_1_Rickettsia_prowazekii_str__Madrid_E_chromosome_complete_genome probe_cstm_Rprow_021218_1x_104138 TATGGAATTCTTGAAAATCTTTTAAATCTTCAGAAGATAAATTTTTACGTGCTATAGATGCAGCTTTCATCTTATTACCTATAATATATTCTGCAAAAGCTAAAATTACAGCATGCCTAT 586080 586200

NC_000963_1_Rickettsia_prowazekii_str__Madrid_E_chromosome_complete_genome probe_cstm_Rprow_021218_1x_104140 ttaagaataaagtagtttcaatattataatcatataaaCTAATGACCACTTTTGAATCAAAGTGCTTTATAAAAGTAAATCCGAAATAGAGCGCTAAAAAAGTAATACATATTAATAATA 586320 586440

NC_000963_1_Rickettsia_prowazekii_str__Madrid_E_chromosome_complete_genome probe_cstm_Rprow_021218_1x_104141 ACCTAAACATCTTTATTTACAAACTCCTGCATAAATTTTTCTGAATACAAAAAATAGATTAGATAGTTTAATTTCTCTAAAATGAGAGTTTTATCTTGTTCTTTTACTATCATAACAGGA 586440 586560

NC_000963_1_Rickettsia_prowazekii_str__Madrid_E_chromosome_complete_genome probe_cstm_Rprow_021218_1x_104142 GATTTTTTCTCAATTTTTATTAATTGTTCTAACCATTTATGGTGTATAGGGAAAATTACTACTGTACTATTTGATTTAGATACAAGataattattattatatttttctaaattatttaaa 586560 586680

NC_000963_1_Rickettsia_prowazekii_str__Madrid_E_chromosome_complete_genome probe_cstm_Rprow_021218_1x_104143 atattccttatattttGAGGTAATGGATAGGATTTTAGTATTCTTAGTTCTTTACTATAATCCTTATCTTGTATGAAATTATATACTAATAGATTTACATTTAATAAGTAATGTGTATAC 586680 586800

NC_000963_1_Rickettsia_prowazekii_str__Madrid_E_chromosome_complete_genome probe_cstm_Rprow_021218_1x_104144 CTTATAGGTACTTCTACAATATTATCCGTCACTTCTACACTCTCATGAATTATTGCTTCAGAATCCGGTATTGATGTTTTAGAGGACTTTAAAGAATTTACGTAATTTAAAAGCGACTTG 586800 586920

NC_000963_1_Rickettsia_prowazekii_str__Madrid_E_chromosome_complete_genome probe_cstm_Rprow_021218_1x_104146 GACCTATTATGATAAAGAATAACAGAAGATAAGATAAATATTACTATTGCTATAAAAAAGATGTTTTGACTTCTAAATAAACTATCAAATTTTTGCATTTTCGTATAATAACTTTATTAT 587040 587160

NC_000963_1_Rickettsia_prowazekii_str__Madrid_E_chromosome_complete_genome probe_cstm_Rprow_021218_1x_104147 ATCGTGAGGGCTTTGATTATCACAATAAACCACATTTTTTATAAAAGGTCTAACTATATTAGCGACTTTTAAACTTATTGCTATAACTAAACTATCTTGTAAGTACTGTAGTAAATTATT 587160 587280

NC_000963_1_Rickettsia_prowazekii_str__Madrid_E_chromosome_complete_genome probe_cstm_Rprow_021218_1x_104148 TTGTAGTAATAATCTTACTAAAGTCTTAGCACTATTCTGAGAATAAAGTAAAATAAAATCAATAGACATCTTTCCAAGCGGTACTTCTAGAGGATACGTTGAGCCTAGACTAGGATCATT 587280 587400

NC_000963_1_Rickettsia_prowazekii_str__Madrid_E_chromosome_complete_genome probe_cstm_Rprow_021218_1x_104149 AAATACTTTTTTATACGCTGTGGTGGCACGTATTGTATTCGCTTTAAATGCGTTTCTATAAGCTGGTTTAGAAAAATATCTAATATTTTCAAATTCTTGTATAATTGATATTGGAAGCTC 587400 587520

NC_000963_1_Rickettsia_prowazekii_str__Madrid_E_chromosome_complete_genome probe_cstm_Rprow_021218_1x_104150 ATTTAAATATTCTACATTATAAATGATATGTCTAGCTATTTTATTAGGTAAATCTTGAGTAATTTCATTCGAAGATAAATATATAGTATGTTTATATAAATCAGTTGGAAAGTGTTGAAT 587520 587640

NC_000963_1_Rickettsia_prowazekii_str__Madrid_E_chromosome_complete_genome probe_cstm_Rprow_021218_1x_104151 CAAATCTGCAATGTTGTTAGCGGTATATATTACTTTTTTGCCTAATAATTGTTTTGTTTTATTGCCTACTACCCAAATGTCTTGTTTTAAGTTATAATCTGCTAAGATGTGTGCTGCATA 587640 587760

NC_000963_1_Rickettsia_prowazekii_str__Madrid_E_chromosome_complete_genome probe_cstm_Rprow_021218_1x_104153 CTGTATAGTTTCGTTATTTTCTTGAATATTTCTAGTCAATAAAACTGATTTCATTTGTATTACAATATAAAAATTAATTGATAATAATAGAAGCTATATTATGGTATAAATTATACTATC 587880 588000

NC_000963_1_Rickettsia_prowazekii_str__Madrid_E_chromosome_complete_genome probe_cstm_Rprow_021218_1x_104154 TGTATTTAGCTTTTTGCTATTAAAATCTATCCGTAACTTCTAAAGTAAAAATCATATTTAAGATATAACTATACAAGTTATAATATAACTTACTCTACCTATTACTATAATCGTATCTAT 588000 588120

NC_000963_1_Rickettsia_prowazekii_str__Madrid_E_chromosome_complete_genome probe_cstm_Rprow_021218_1x_104155 AATGTATGCATATTGAATATTAACCATTATACGAGAAAAAGCATTATTAAATTTTAGATGTTTTGTTTCTTGATTAAATATAAAAATATTCTGTATCTCAGTGGTACTAATTCATATCCA 588120 588240

NC_000963_1_Rickettsia_prowazekii_str__Madrid_E_chromosome_complete_genome probe_cstm_Rprow_021218_1x_104156 AAGGTACTTCAGtttattttagtttttatttttaATAATATTAATAAAGATGGGATAGTTATAATAGTAGTAAATAAAAAGAAATTTTGCCAACCAAAATTTACTACCATATAGCCTGAA 588240 588360

NC_000963_1_Rickettsia_prowazekii_str__Madrid_E_chromosome_complete_genome probe_cstm_Rprow_021218_1x_104157 ATTATAGGAAAAATTGAACGCGAAATTCCCATCATTGATGAAAGCAAAGAATATTGAGTAGCGCGGAATTTACCTTGACACAGCGAAGAAATAAAAGCAATATACGCAGTCATAGTCATA 588360 588480

NC_000963_1_Rickettsia_prowazekii_str__Madrid_E_chromosome_complete_genome probe_cstm_Rprow_021218_1x_104158 CCGCCAGTAATGCTTTCTACTCCTATCGTAATAAATAATATTAAAGAATTTTTACCGTTTATTTCAAGCAAAATAAAGAAAATATGACTTAAAGCGTGAATTATACCAAACaataatata 588480 588600

NC_000963_1_Rickettsia_prowazekii_str__Madrid_E_chromosome_complete_genome probe_cstm_Rprow_021218_1x_104159 ctatataatatatttttatatttcataataataCCACCGATGAGTCCACCGATTATAGCTCCCATAACGCCACAAAACTTGCAAACACTTGCTATCTCAAAAGCATTATACCCAAGGTGA 588600 588720

NC_000963_1_Rickettsia_prowazekii_str__Madrid_E_chromosome_complete_genome probe_cstm_Rprow_021218_1x_104160 AGTAAAAATGGATTAATCATAACATTAATTAAGTTATCTGGTAATCTATATAATACTAGAAAAATTAATATAAGAATAATGTAGTATGCTAGGTTTATATCATTCCTATGGAAGtaaaaa 588720 588840

NC_000963_1_Rickettsia_prowazekii_str__Madrid_E_chromosome_complete_genome probe_cstm_Rprow_021218_1x_104162 GAATAAGATGTGTTTTCTTCCATAACATCAAAAGAATTAGTATATCTAGCAACTAAAATAAGTAAAATTAAATAGACAAATATTACACCGGCAAAGATTTTATATATTGCATTAAATGTT 588960 589080

NC_000963_1_Rickettsia_prowazekii_str__Madrid_E_chromosome_complete_genome probe_cstm_Rprow_021218_1x_104163 AAATAAATAGATAAATAAATAGCTCCGGAACTTGCTAAAAGCATACCGACTCGATAACCTAATATATATATTCCTGAAGTAAACCCAAGTGACTCTTTAGGTACTATTTTTGTTCTTAAA 589080 589200

NC_000963_1_Rickettsia_prowazekii_str__Madrid_E_chromosome_complete_genome probe_cstm_Rprow_021218_1x_104164 GCGCTGAGTATAGTATCTTGTGCTGCACTaaaaaaaGATATAATGAAAGCAATAAATGACAATAATACTAGATCAGTACTAGGATCTAGAAAGCTGAGAATAAATATAAGAGATATTAAT 589200 589320

NC_000963_1_Rickettsia_prowazekii_str__Madrid_E_chromosome_complete_genome probe_cstm_Rprow_021218_1x_104165 GTTGTACTCGTTAGGCAAATCCAAGATAATCTATGACCTAATATTTTGTTTAAATACTTAATTTGCACTGTATCAAAAACTGGTGCTAATAAAAAATTAATAGAATATGGGAGTGTTATG 589320 589440

NC_000963_1_Rickettsia_prowazekii_str__Madrid_E_chromosome_complete_genome probe_cstm_Rprow_021218_1x_104166 AATGATAACATGCCTATTGTTTGCAATGCGATATCTTTTTTAGCAAACCAATAATTTAAAGTATTACCGGTAATCATTACATTAAAACCGCTAATAAATCCAAATAACCAAATAATATAT 589440 589560

NC_000963_1_Rickettsia_prowazekii_str__Madrid_E_chromosome_complete_genome probe_cstm_Rprow_021218_1x_104167 ATATGAGAGTTTTTTAACATGAATTTTATATATGCACTTTTTAACATAAGTGCTTATTAGGAATAATATATAAAAATGTGAGTATTCTGTTCATTGAATCCAGTTATGAGCTAAGCAAAT 589560 589680

NC_000963_1_Rickettsia_prowazekii_str__Madrid_E_chromosome_complete_genome probe_cstm_Rprow_021218_1x_104168 ATTTCCTATAGTTTAGAATGATAGTTAAAAATGTTTACTATTCGTATTTTAGCATTTTCCTTAATCTTATTCAAGGTAAAGATCGGCTATTGCTTGCAATCTCTTTAATAAGACTTGTGC 589680 589800

NC_000963_1_Rickettsia_prowazekii_str__Madrid_E_chromosome_complete_genome probe_cstm_Rprow_021218_1x_104169 ATATTTTAAAAAGTACATAATTTGAAGCCTCTCCTTTTTCTTTTAATTTTGTATCAAAAATATTTTGATTATTCATTAATAATCTAAGTTGTTTTTAAAAAATACACTATAGACTAGTGG 589800 589920

NC_000963_1_Rickettsia_prowazekii_str__Madrid_E_chromosome_complete_genome probe_cstm_Rprow_021218_1x_104171 AAATTTAATATTATTCttttttatttttCTAATGGTAATTGTTATTTCCGttttttttATAAGTAATTATAGAATGCATAAAAATACAAATTGTATTATGGATATTGGCACTTATAGTTA 590040 590160

NC_000963_1_Rickettsia_prowazekii_str__Madrid_E_chromosome_complete_genome probe_cstm_Rprow_021218_1x_104172 TAAAAAATTCTATAGTACAATACGTGAAGATCAATTAAATCTTGATTTAGCACTGGAACGATTAGAAGATATTGAATCTTTAAATACCAAAGATAAATCTAGATTAGAAAAAATTTGTTC 590160 590280

NC_000963_1_Rickettsia_prowazekii_str__Madrid_E_chromosome_complete_genome probe_cstm_Rprow_021218_1x_104173 TTTTCAAGTAGAAGATGGAATGGTACGTTTAGATGTAGCAAAAGGTTATGATTGCTTACTACGTTTTGCTCTAGATAAAAATGCAAATTTAGTCCAGCAATATAAAGCGATTAAAAACGG 590280 590400

NC_000963_1_Rickettsia_prowazekii_str__Madrid_E_chromosome_complete_genome probe_cstm_Rprow_021218_1x_104174 TAGTTATAGTTTAGATACTTTATATACTAAAACAGCTGCATTTAATTTAGCTACACTCATCATTTCAGGTTACAAAGAAAAAGTAAAAGAAATATTTGAACTAAAAAATGATGATGATTT 590400 590520

NC_000963_1_Rickettsia_prowazekii_str__Madrid_E_chromosome_complete_genome probe_cstm_Rprow_021218_1x_104175 ACTCAAATATTATAGCGGTTATACAGAAGATGAGGTTCCATTTTTAAAGGGTTTAATTTCTCTTAATATACCAGAACTTACTGCTAGATGTTTATATAGATTGAGCTTAATTCATATACT 590520 590640

NC_000963_1_Rickettsia_prowazekii_str__Madrid_E_chromosome_complete_genome probe_cstm_Rprow_021218_1x_104176 AGGTAGGTCATCTTTTAATGAAAAAACTATACGAACTGATTACAAGCTTGCAATAAAAGAGATACAAAACGTAATTAAATTAACTGGTATCAAGCAAAACAACATATTAGATATAGCATT 590640 590760

NC_000963_1_Rickettsia_prowazekii_str__Madrid_E_chromosome_complete_genome probe_cstm_Rprow_021218_1x_104177 AACTATTAATGATAAATATGCTATTCATGCTGCTGCAGTAATAGCATCCAGTTTACTAAATAGCGATTTAGATAGTTTCTATAGATTCCATATTGTTATGGATTCTAATGACCCTATTAG 590760 590880

NC_000963_1_Rickettsia_prowazekii_str__Madrid_E_chromosome_complete_genome probe_cstm_Rprow_021218_1x_104178 TCAAGAATCAATAAATAAATTATCTTCTATGCAATATATTAGAGATTATTCTATAGATTTCACTACTTTCCCTGACAATATATTAAATAAAGCTTTAACATATAAGGAAATAAAATTTTC 590880 591000

NC_000963_1_Rickettsia_prowazekii_str__Madrid_E_chromosome_complete_genome probe_cstm_Rprow_021218_1x_104179 AAATAATTGGCCCAGTTTAGTAATGTATAGATTATATTTTGATAAAATTTTTCCTAATCTAGATTCTATACTATATCTTGATGCAGATATTGTAGTGCTACGTGATTTGAATTCTCTGaa 591000 591120

NC_000963_1_Rickettsia_prowazekii_str__Madrid_E_chromosome_complete_genome probe_cstm_Rprow_021218_1x_104180 aaaaaTAGATATGAACAATTATATTGCCGCATGTAGTCTAGATACAGCTATTACATACTGTATTCATAAAGTACAAGAAGAATGTAAAAGGAATGTGGCTCATTCTTATAAAAATAGTGG 591120 591240

NC_000963_1_Rickettsia_prowazekii_str__Madrid_E_chromosome_complete_genome probe_cstm_Rprow_021218_1x_104183 TGAAAAGGAAGAATTATGGAAAACAAATCATGATAAATTAGATAAGATTACACAATATTATTGGCGATATAGAGAAATAACACCTTGGAATAGTATCAATTGATGATTTTAATTGACTTT 591480 591600

NC_000963_1_Rickettsia_prowazekii_str__Madrid_E_chromosome_complete_genome probe_cstm_Rprow_021218_1x_104184 AAAACTTAAGCGATTTAAAAGTTAACTTTAATATGTAAGGTTACAATAAAAATGTTACCGCCTAAAATTTTCTTTGAAAAAGTTAAAGAAATAATTTGGCCTATAGAAAGGAAAGAATTA 591600 591720

NC_000963_1_Rickettsia_prowazekii_str__Madrid_E_chromosome_complete_genome probe_cstm_Rprow_021218_1x_104185 AAGCTATTTATACCAATGGCTTTAATGATGTTATGTATCCTGTTTAATTTTGGGGCTTTAAGATCTATTAAAGATAGTTTAGTAGTACCCTCTATGGGGGCTGAAATTATTAGTTTCTTA 591720 591840

NC_000963_1_Rickettsia_prowazekii_str__Madrid_E_chromosome_complete_genome probe_cstm_Rprow_021218_1x_104187 ttattatttgcctatattatttatCCAAATCAAGATATTTATCATCCTAATGATGCAATGATAAATAATTTAATTGCTTCATACCCTAATTTAAAGTGGTTTATTAAAATAGGTAGTAAA 591960 592080

NC_000963_1_Rickettsia_prowazekii_str__Madrid_E_chromosome_complete_genome probe_cstm_Rprow_021218_1x_104188 TGGAGTTATGCGCTAATGTATATTTTCTCAGAATTATGGAGTGCAGTAGTTATAAACTTAATGTTTTGGCAATTTGCTAATCACATTTTTGATACTGCTAAAGCTAAACGATTTTATCCT 592080 592200

NC_000963_1_Rickettsia_prowazekii_str__Madrid_E_chromosome_complete_genome probe_cstm_Rprow_021218_1x_104189 GTTCTTGGGATGGTTGGTAATATCGGTCTTATAATAGCAGGCAGCGTACTTGtttttttttCAAGTGGGCAGTACATCATTGATTCAGAATTATTAACGGATTCTTATAATTCATCTTCT 592200 592320

NC_009488_1_Orientia_tsutsugamushi_Boryong_complete_genome probe_cstm_Rprow_021218_1x_10419 GAATTAGGGGTTTAATTAATGAAGTTGCAAATGAAAATGAGTTACGTAAAGAGGCTAATTTAAAAATTATTAAGGATGCTGATGTAATAACGGATTCTCTATAACACATTACAAATCTAT 1250160 1250280

NC_000963_1_Rickettsia_prowazekii_str__Madrid_E_chromosome_complete_genome probe_cstm_Rprow_021218_1x_104190 AACAATTCTATCATGCTTCAGCCAATCATATCAATTATTGTTACTGCAGGAATAATTGCTATGTTTTTATTTAGAATAATAAATAAATTTATTTTAACTAATTCTATAAATGTTTTAGAT 592320 592440

NC_000963_1_Rickettsia_prowazekii_str__Madrid_E_chromosome_complete_genome probe_cstm_Rprow_021218_1x_104191 GTaaaaaaagttgctgctaaaacaaaaacaaaaCTTGCATTAATTGAAAGTATAAAATTAATAATTCATTCAAAATATATAGGTCGTATTGCATTATTAATAATCTGTTATGGATTACTA 592440 592560

NC_000963_1_Rickettsia_prowazekii_str__Madrid_E_chromosome_complete_genome probe_cstm_Rprow_021218_1x_104192 ATAAATATAGTTGAAGGACCTTGGAAAGCGAAAATAAAAGAATTACATCCAAATACTGTAGATTATGTTAATTTTATGGGCATGTTTAATATTTGGATGGGGATCTCATGTGTTACTTTC 592560 592680

NC_000963_1_Rickettsia_prowazekii_str__Madrid_E_chromosome_complete_genome probe_cstm_Rprow_021218_1x_104193 ATGATAATAGGTAGTAATATTCTTAGAAGGCTTGGTTGGCTCATTTCTGCATTATTAACTCCTATTATGTTATCTATTACAGGCTTCATGTTTTTTATCTTTATAATTTTTATTGAAGAA 592680 592800

NC_000963_1_Rickettsia_prowazekii_str__Madrid_E_chromosome_complete_genome probe_cstm_Rprow_021218_1x_104194 ATAGGTACATGTTTTGGTGATTTTAATCTTCTATATGTAGCGATTATTGTCGGAGCAATTCAGAATATACTTAGTAAATCGTCTAAATATTCATTATTCGATTCAACAAAAGAAATGGCA 592800 592920

NC_000963_1_Rickettsia_prowazekii_str__Madrid_E_chromosome_complete_genome probe_cstm_Rprow_021218_1x_104195 TATATTCCTTTATCTTTAGAACTGAGAACTAAGGGAAAAGCCGCTGTAGAGGTAATAGGAACGAAATTTGGTAAATCACTTGGAGCATTTATCCAGTCTTTGATATTTATTATTATTCCA 592920 593040

NC_000963_1_Rickettsia_prowazekii_str__Madrid_E_chromosome_complete_genome probe_cstm_Rprow_021218_1x_104196 ACGGCTACCTTTGATTCTATTATAATATATTTACTAGTAATTTTTATAGTGATGATGAATTTATGGATTTGGAATATTATAAAATTAAATAAGGAATATATAAAGCTGTGTCAATAATAG 593040 593160

NC_000963_1_Rickettsia_prowazekii_str__Madrid_E_chromosome_complete_genome probe_cstm_Rprow_021218_1x_104197 TATTACTGCATTGTGGATAATCATATGCTACTTGTTTTACTAAAATCTAATTTTTCTTGCAATTATAAACTAGCAAATCATACATCAATGTTAATTTAAAATGaaaaaaaTTCttataat 593160 593280

NC_000963_1_Rickettsia_prowazekii_str__Madrid_E_chromosome_complete_genome probe_cstm_Rprow_021218_1x_104198 aatattatttataattatatttattgtactaatatataGTGGACTGTGGTTTGTTATTATGTTTTCGTTATCACACTCTATAAATCAAAAATATTCTGGTGTACATCTTAATATAGGAAA 593280 593400

NC_000963_1_Rickettsia_prowazekii_str__Madrid_E_chromosome_complete_genome probe_cstm_Rprow_021218_1x_104199 GGGTAATAATAATCCTCATCAGCAATATCTTGTTAAATTTTCTAAAGTACAACCATATGGTTTCCCTTTTAAATTAGGTATTATGGTAATTAATTGGCAAGAAGAAAGTATTAATAGAGC 593400 593520

NC_000963_1_Rickettsia_prowazekii_str__Madrid_E_chromosome_complete_genome probe_cstm_Rprow_021218_1x_104200 AATTGAATTTACCAAACCAATCAATATTGGTTATGATTTACTTGGACAGAAATTATTTATAAATTTTTCTGGAGAAGCATTAGGAAAATTTAAGCCTGTGCAAAGAGGATTTGGAGTAAA 593520 593640

NC_000963_1_Rickettsia_prowazekii_str__Madrid_E_chromosome_complete_genome probe_cstm_Rprow_021218_1x_104201 ATTTTATAATGAGAACTGCATATTATCGGCAAAGATACCATTAAACTTAAAATTATTTAAAATGGTACTGTTaaaaaaaaaTTTATTTGAATTTCTAAATTTAATAGAAAATATTAAATT 593640 593760

NC_000963_1_Rickettsia_prowazekii_str__Madrid_E_chromosome_complete_genome probe_cstm_Rprow_021218_1x_104202 TATTTCAGATAAGACACAAATTTTTGACTTAGTAGATAATCAAAAATTATATGAAGAAGATCATACTATACTTACTATGTTAGTTGATAAGAGGCAGTATTATACAAGTAAACAGGATTT 593760 593880

NC_000963_1_Rickettsia_prowazekii_str__Madrid_E_chromosome_complete_genome probe_cstm_Rprow_021218_1x_104203 TCTTAACAATATACCACAAAAATTAGAATTTTATTATGAAACCGAAATAATACAAAGTAATCTTGAAGATAGAATAATACCTGCAGGATTATTATTATATAGACCGGCTTGGAATAATAA 593880 594000

NC_000963_1_Rickettsia_prowazekii_str__Madrid_E_chromosome_complete_genome probe_cstm_Rprow_021218_1x_104204 TTTTAAATTTTCTGGTAATTTCTTGATTAGTACTAGTAGCTTACATTTTAAAGATATTGCTAAAGATTTAACAATTAAAGTTAATAACGCAAAAATAAATAGTAATAACTTTGAAAATAA 594000 594120

NC_000963_1_Rickettsia_prowazekii_str__Madrid_E_chromosome_complete_genome probe_cstm_Rprow_021218_1x_104205 TATGAATTTATTATATAAAGGTAAGTTAAATGATTTTGGTAACAGTAATATTCATCTTTCGATAGAATCGCAATTTAAGCTAAAACCTGGTTTTATAATAGGGTTTTTAGAATTTTTAAA 594120 594240

NC_000963_1_Rickettsia_prowazekii_str__Madrid_E_chromosome_complete_genome probe_cstm_Rprow_021218_1x_104207 AGAAGATAGACCATATCATTTTAATTTTAATATTAATTTAGTTACAGAATTAAAGAAATTAACTAGAGTACAGATAAATACTTTAAGTTTATATTCCAACACATCAGGATTTAATATAAC 594360 594480

NC_000963_1_Rickettsia_prowazekii_str__Madrid_E_chromosome_complete_genome probe_cstm_Rprow_021218_1x_104208 AAATGAAACTATAATAAATGATCTTAAAGATTCGTACACTAAAGGTATAATAGTAATTAATAATTACTCTAAAATAATAGAGATTTTAAGTTTCTATATATATGGAGTAGGGAGTTTTAA 594480 594600

NC_000963_1_Rickettsia_prowazekii_str__Madrid_E_chromosome_complete_genome probe_cstm_Rprow_021218_1x_104209 AAATTTATCTAAAGAGAGTCAGATAGTACATATAGAGGCTTTGCAATCATTTTTAAAAACTATATCCGATCACCCTAATTCATCTAATTTAATTGATACTAGTATTAAGTATGAGTTTAA 594600 594720

NC_000963_1_Rickettsia_prowazekii_str__Madrid_E_chromosome_complete_genome probe_cstm_Rprow_021218_1x_104210 TTTATCAGATTTAAATAAAGCTAAAATAGGTAATATAGATGATATTAATAAACTTATACCTTTGTATTATTTATCTTTATATCAAGCAGCAGTaaaaaaaaTGGAGCCTGGAGCAAACGT 594720 594840

NC_000963_1_Rickettsia_prowazekii_str__Madrid_E_chromosome_complete_genome probe_cstm_Rprow_021218_1x_104211 AAAAGAAAAAATTCTTGAGTTAATTCCAAGTATTAATCAAAAAATATTAGAGGAATGTGTATTATCTGATATTGTAACACAGTAATCTGATCACAAAATATGGATATAGCGGTTAAGCTA 594840 594960

NC_000963_1_Rickettsia_prowazekii_str__Madrid_E_chromosome_complete_genome probe_cstm_Rprow_021218_1x_104212 TAGTATGACAACTAATACTGGAATTTAGCAATAAAAAATATCATGAATATTATAGTAAAAATACAGCAAAATTTAAAAGATGAAGTAACTCAGTTAAACGATTTAATTATTAGTTGTTTA 594960 595080

NC_000963_1_Rickettsia_prowazekii_str__Madrid_E_chromosome_complete_genome probe_cstm_Rprow_021218_1x_104213 AAAAGTGATGCAGAATTAATAGAAAAGGTCGGCAAATATTTAGTAGAGGCAGGTGGTAAAAGAATTCGTCCACTTTTAACTATAATCACGGCTAAAATGTTCGATTATAAAGGCAATAAT 595080 595200

NC_000963_1_Rickettsia_prowazekii_str__Madrid_E_chromosome_complete_genome probe_cstm_Rprow_021218_1x_104214 CATATCAAACTTGCAAGTGCCGTGGAATTCATTCACGCCGCTACTTTGCTTCATGATGATGTGGTAGACAACAGCACTTTAAGAAGATTTAAACCTACAGCTAACGTTATTTGGGGCAGT 595200 595320

NC_000963_1_Rickettsia_prowazekii_str__Madrid_E_chromosome_complete_genome probe_cstm_Rprow_021218_1x_104215 AAAACAAGTATTTTAGTTGGCGATTTCCTTTTTAGTCAATCTTTTAAGTTAATGGTTGCTTCTGGCTGTATTAAAGCTATGAATGTTCTAGCTAAAGCTTCGGTTATTATTTCCGAAGGT 595320 595440

NC_000963_1_Rickettsia_prowazekii_str__Madrid_E_chromosome_complete_genome probe_cstm_Rprow_021218_1x_104216 GAGGTAGTACAATTAGTTAAGTTAAATGAGCGACGTATTATCACTATAGATGAATATCAGCAAATAGTGAAATCTAAAACGGCTGAGCTATTTGGAGCTGCTTGTGAAGTCGGAGCTATT 595440 595560

NC_000963_1_Rickettsia_prowazekii_str__Madrid_E_chromosome_complete_genome probe_cstm_Rprow_021218_1x_104217 ATAGCAGAGCAGGTCGATCGCGTTTCTAAAGATGTGCAGAATTTTGGTAGGTTACTTGGTACAATATTTCAGGTTATAGATGACTTACTTGATTATTTAGGTAGTGATAAACAGGTAGGA 595560 595680

NC_000963_1_Rickettsia_prowazekii_str__Madrid_E_chromosome_complete_genome probe_cstm_Rprow_021218_1x_104218 AAAAACATCGGTGATGATTTCTTAGAAGGAAAAGTAACATTACCACTGAtttttttATATCATAAGCTAGAGCAAGATAAGCAGCTTTGGCTAGAAAATATGCTTAAATCTGATAAGCGT 595680 595800

NC_000963_1_Rickettsia_prowazekii_str__Madrid_E_chromosome_complete_genome probe_cstm_Rprow_021218_1x_104219 ACTAAAGATGACTTTGTAAAAATACGTGACTTAATGTTAAAACATGCAATTTACAATGAAACAGTTAATTATTTGAGTAGCTTAGAAAACGAAGCAaataatttattaaataaaattccc 595800 595920

NC_000963_1_Rickettsia_prowazekii_str__Madrid_E_chromosome_complete_genome probe_cstm_Rprow_021218_1x_104221 GAAGGCAAAGGTCATTGGTTCAAATCCAATCGAATGCACCATCAATAAAAATTGTTCTATAATATGTATTATGGAAAATAAACAGATTTATGGCTGGGACGGGAGGATTCGAACCTCCGT 596040 596160

NC_000963_1_Rickettsia_prowazekii_str__Madrid_E_chromosome_complete_genome probe_cstm_Rprow_021218_1x_104222 ATGACGATACCAAAAACCGTTGCCTTACCACTTGGCGACGTCCCAATTTAAAATGCAGGAATAGAACTTATATCAATAAATAAGAGAAACGTCAATTATAATAATGATTGTAAAAATAAA 596160 596280

NC_000963_1_Rickettsia_prowazekii_str__Madrid_E_chromosome_complete_genome probe_cstm_Rprow_021218_1x_104224 TCAATTAATTTACTAGCATAAGGCACTAAACTTAAGGTTTCAAATTCAAGCCATCCATTATTTTCTTTTACATATATTAAATTCTCAATTCTAATACCATATTTTCCTGGAATATAAAAT 596400 596520

NC_000963_1_Rickettsia_prowazekii_str__Madrid_E_chromosome_complete_genome probe_cstm_Rprow_021218_1x_104225 CCAGGTTCGTTAGATAAGATCATACCTGCTTTAAGTATTATTTTATTACTAAGATTTATACTTTGTGGCCCTTCATGAACGCTTAAGAAACTTCCTACTCCATGCCCGGTACCATGCGGA 596520 596640

NC_000963_1_Rickettsia_prowazekii_str__Madrid_E_chromosome_complete_genome probe_cstm_Rprow_021218_1x_104226 TAATCTATCATGTCTTGCCATAAATATTGCCGTGCAAGTATATCTAGGTTCGCTCCTGTTACGATATTTTTTGGGAATTTAGCTCTAGTTAAAGCAATATGTCCTTTAAGTACTTGCGTA 596640 596760

NC_000963_1_Rickettsia_prowazekii_str__Madrid_E_chromosome_complete_genome probe_cstm_Rprow_021218_1x_104227 TAACGTTTTTTCTGTTCACATGTTGGTATACCTACAATGATTGTCCTAGTTATATCAGTGGTAGCACCTTTATATTGAGCACCAGAATCAATCAGTAGTATACCATGTCCTTCAATTTTC 596760 596880

NC_000963_1_Rickettsia_prowazekii_str__Madrid_E_chromosome_complete_genome probe_cstm_Rprow_021218_1x_104228 TTAGCAGTTTTTGGGTTAGCTCTATAATGAATAATAGCACTATTTTCTTGAAATCCGCAAATAGCATGAAAACTATCAGAAACATATCCCTCTTGTTTTGCTCTTTGCGCTGTTAGTTTT 596880 597000

NC_000963_1_Rickettsia_prowazekii_str__Madrid_E_chromosome_complete_genome probe_cstm_Rprow_021218_1x_104229 AAACAGAGAGAATGTTCTGTTATTATCTCATGACTGTGAAAGCAGGAATTCACGTTTTCACTTGAATGATATAGAAAAAACTCAGCAAAAAACTCACATAAAGCTACTGCATCTTTAATA 597000 597120

NC_009488_1_Orientia_tsutsugamushi_Boryong_complete_genome probe_cstm_Rprow_021218_1x_10423 TTATGTTTTAGCAAAAGCGTATGAGCTTGCAACAAATTGTAGACAAAAAGTTATTGGCCTTGCTCCCACTCATAAGGCAGTATTAGAGCTGAAGAACAAAGGTTATACAGATGTCTATAC 1250640 1250760

NC_000963_1_Rickettsia_prowazekii_str__Madrid_E_chromosome_complete_genome probe_cstm_Rprow_021218_1x_104230 TGAAAATCAATTGCATGTTTAATTTCTATATCATTTTTACAAGCTTTTGCTAATAAACAAGGTTCTACGAttttttttacttttttATTAGCTATTAAATCCATTATGTGAACTGATGTT 597120 597240

NC_000963_1_Rickettsia_prowazekii_str__Madrid_E_chromosome_complete_genome probe_cstm_Rprow_021218_1x_104231 ATACTGTCATCGATAAGATATCTATTCTTACTATCTCTTAAAATATTTTCAAATTCTTTTTCCGGTAGAATTGTGATTTCAGGACGTGCATTAATAATTTCAGTATCAATTCTTATAGGG 597240 597360

NC_000963_1_Rickettsia_prowazekii_str__Madrid_E_chromosome_complete_genome probe_cstm_Rprow_021218_1x_104232 TTGATAAATAGATATAATTTTGTAGAAGTAAGTATAACTTTTGCAAACATTAGTGGTGTATAGTTTACATCACTAGCACGCAAATTTAATAACCAACATATGGAAGCGCTATCTAGAATG 597360 597480

NC_000963_1_Rickettsia_prowazekii_str__Madrid_E_chromosome_complete_genome probe_cstm_Rprow_021218_1x_104233 ACTAGAATGTCATCGTTATTTTTATTGCATCCCACGGTCACGCTGCTGGATGATAATATGGTTTCACGACATTTACGTATTTTATCATTATGACTAACCCCTGCAAATTTAATATCATGT 597480 597600

NC_000963_1_Rickettsia_prowazekii_str__Madrid_E_chromosome_complete_genome probe_cstm_Rprow_021218_1x_104234 AAATAGACTTTAGAATTTGGTTCTAGAGGCTTGTTATACCAAATTTTATCAACTAAATTTTCGTTTATTTTATGAAAATTGATATTTAAATTTGCTAAGACTTGGTAAGTAAATAATTGA 597600 597720

NC_000963_1_Rickettsia_prowazekii_str__Madrid_E_chromosome_complete_genome probe_cstm_Rprow_021218_1x_104236 AAGAAGAATGCAGCATCTTTACATATAATAGCTATACCACTTGAGCCGGTAAAACCTGTTATATATTCAAGCCTTTTAGCATACTCAGGTACATATTCGTTCATATATTTATCATTAGAT 597840 597960

NC_000963_1_Rickettsia_prowazekii_str__Madrid_E_chromosome_complete_genome probe_cstm_Rprow_021218_1x_104237 GGTATGATATAACCTTCTATATTATATTCTATAAATAATTTTCTAAGTAGATTAATCCGATCGTGTCTCATATTCACACTATATACAACAATTTGATCTCAGTATCTAAAAATATTAAAT 597960 598080

NC_000963_1_Rickettsia_prowazekii_str__Madrid_E_chromosome_complete_genome probe_cstm_Rprow_021218_1x_104238 GGACACAATGAATAAGTCACTTTATTATATAGACTATAATTTAATTCTATTCATCAATCTATACCATCCATAATACACAGGAATACCAAGGATAGTAAAAGAACAAGCTATAATTAGAGT 598080 598200

NC_000963_1_Rickettsia_prowazekii_str__Madrid_E_chromosome_complete_genome probe_cstm_Rprow_021218_1x_104239 TTCAAAAGGTGTTTTATAAATGACCCAAGTACAAAATATAATGGATATTATGGCTACAAATAAATAATAATACGAGAAATTTTCTTTTGAACtaaaaattacttttaaaaaagctaagct 598200 598320

NC_000963_1_Rickettsia_prowazekii_str__Madrid_E_chromosome_complete_genome probe_cstm_Rprow_021218_1x_104241 AATTATTCCATAAGTTGGAGCATTATTACTGttttttttAGCAAAAAATTTTGGCAATAACCCATCTTCTGCAAGTCCTAGTGCAATCTGTCCACTAGTTAGAACCCAAGCGTTAAGAGT 598440 598560

NC_000963_1_Rickettsia_prowazekii_str__Madrid_E_chromosome_complete_genome probe_cstm_Rprow_021218_1x_104242 ACCTATACATATAACAGAAGCTATCACTGTAATTACTTTTGACCATGTACCACCGAATAATAATGTAGCGGCATCTGCATAAGGAGCTTTAGAATTAATAAGTTCAGAAGCTGGAATTAA 598560 598680

NC_000963_1_Rickettsia_prowazekii_str__Madrid_E_chromosome_complete_genome probe_cstm_Rprow_021218_1x_104243 TCCAATGATACCTATGCTATTAATAATATATAAGAACGCTACACAACAAGTTCCAATTATTATGGCTCTTGGAATAGTTTTCGCCGGATCTTTTACTGTTCCTGCTGTAGTAGTTGCACA 598680 598800

NC_000963_1_Rickettsia_prowazekii_str__Madrid_E_chromosome_complete_genome probe_cstm_Rprow_021218_1x_104244 CTCTATTCCAATAAATCCCCAAAAAGTCAGAAGTGCAACTCTTCCCATAATAGTCGGGATAGTGAAATTTTCTACTTCTTTAGCAATCACTATATTATCTATATTAAAATGGAATAATGC 598800 598920

NC_000963_1_Rickettsia_prowazekii_str__Madrid_E_chromosome_complete_genome probe_cstm_Rprow_021218_1x_104245 AGCTAAACCTACTACAAGTAGTGGGACAAATTTTAAGAGAGTTAAATAAAACTCTACCTTTCCTGCTATTTTAGGACCTTTTAGATTTAAAATTGCAATAGCAGCTAATAATATTAGCTG 598920 599040

NC_000963_1_Rickettsia_prowazekii_str__Madrid_E_chromosome_complete_genome probe_cstm_Rprow_021218_1x_104246 TAATATCAAATCCAAAATCGTTTGTGATTTAAAGAAAGGCGTTAAATAACCTATTGCTGAGATAACGACTATACTCGTACTGACAAAAGATATAATCCAATAAGTCCAACCAGTAAAGAA 599040 599160

NC_000963_1_Rickettsia_prowazekii_str__Madrid_E_chromosome_complete_genome probe_cstm_Rprow_021218_1x_104247 TGCTATTGTATCTCCAAAACTTGCTCGTACATAAACGTGAGGGCCACCTGTTTTAGGAAATTTTGTACATAAACAAGAAAATACAAGTGCTATACTCATAGCACCAAATAATGACAGAAT 599160 599280

NC_000963_1_Rickettsia_prowazekii_str__Madrid_E_chromosome_complete_genome probe_cstm_Rprow_021218_1x_104248 CCAGCCCCAAATGCTGTATACACCGAATGGTGCTAAACTTAAAGGCAATATAAAAACACTAGTACCGATTTGACTACCTGTCACCAATGCAAATACAGCCCAAAAACTTAATTTTTGTGA 599280 599400

NC_000963_1_Rickettsia_prowazekii_str__Madrid_E_chromosome_complete_genome probe_cstm_Rprow_021218_1x_104249 CATATCAAAATTTGATTTAAAGAAATATTTAAAGAAATAGCTTTATAATAGATGAAATAATACTTTTTAGCAATAGTTAAACTACTATTAATTAATTTTATTCGTGTTTTCTAAACAAAA 599400 599520

NC_000963_1_Rickettsia_prowazekii_str__Madrid_E_chromosome_complete_genome probe_cstm_Rprow_021218_1x_104250 ACTTTATATTGAATAGTATTAATATCATTATTTTTATCTAAATGAAGTTGGATTACACACTAAAGGATTTACCGCAGCCACAATTAGCTTTTTCGTTAGGATTAGTAAAAGTGAACTGTG 599520 599640

NC_000963_1_Rickettsia_prowazekii_str__Madrid_E_chromosome_complete_genome probe_cstm_Rprow_021218_1x_104251 ACTTAAAATTAGTCTCTACATAATCCATTTCAGAACCTAAGATATACATCAATGTTTTTGGGTCAATTAGTATACGTACACCCTTTTCTTCCACTACTTCATCGAATTGATTTTTATTAT 599640 599760

NC_000963_1_Rickettsia_prowazekii_str__Madrid_E_chromosome_complete_genome probe_cstm_Rprow_021218_1x_104252 CAGCATATTCAACGTAATAAGTCTGACCGGCACAACCGCCTGACTTAATACCTACCCTAATACCAAAGGTAGGTTTAGCGCGCTTTTCTATGAGTAATTTTACTTGCTTTGCAGCAGCAT 599760 599880

NC_000963_1_Rickettsia_prowazekii_str__Madrid_E_chromosome_complete_genome probe_cstm_Rprow_021218_1x_104254 CCGGCGGCAGTGATAATTCTTTTGCTATTTCAGTATTTTTAATCTCTTTTGCATCTTCTACTGATTTTCCTTTAATCCACTCTGTGACTAGAGAACTTGAAGCAATAGCCGAACCGCAAC 600000 600120

NC_000963_1_Rickettsia_prowazekii_str__Madrid_E_chromosome_complete_genome probe_cstm_Rprow_021218_1x_104255 CGAATGTTTTAAATTTAGCATCTGTAATAATTTCGTCATCGCCAACTTCGATTTGTAATTTCATAACGTCACCGCAAGCAGGAGCTCCAACGAGTCCTGTACCAACAttttttttctttt 600120 600240

NC_000963_1_Rickettsia_prowazekii_str__Madrid_E_chromosome_complete_genome probe_cstm_Rprow_021218_1x_104256 tATCAAGTGACCCAACATTACGAGGGTTTTCATAATGATCTATCACTTTTTTGCTATAAGCCATTATTATTCTCTTTTAATTGTTATATTATATCATTTCATGACTTGACAAAATAGTAT 600240 600360

NC_000963_1_Rickettsia_prowazekii_str__Madrid_E_chromosome_complete_genome probe_cstm_Rprow_021218_1x_104257 GCAACTCTATCAATGAGCTGTCCACCTAATCTTCTTCAAATCAACTCCTTCTTGCATCATTTCCCAAAGAGGGCTTAATCTCCTTAACTTATCAATTTTTGAGCATACTAAATTTACTGC 600360 600480

NC_000963_1_Rickettsia_prowazekii_str__Madrid_E_chromosome_complete_genome probe_cstm_Rprow_021218_1x_104258 GTAATCAATTTCCTGCTCAGTAGTGAATCTACCTATGCCAAACCTAATTGAAGTATGTGCAAGTTCTTCACTGATGCCTATGGACCGTAAAACATATGATGGCTCTAGAGATGCAGAAGT 600480 600600

NC_000963_1_Rickettsia_prowazekii_str__Madrid_E_chromosome_complete_genome probe_cstm_Rprow_021218_1x_104259 ACAAGCAGAACCAGAAGAAACAGCTAAATCTTTAATAGCAAGAATAATTGATTCGCCTTCTACTCCAGCAAAGCTTAGATTTAGATTGCCTTTATATCTTTGATCTTTATCACCGTTTAA 600600 600720

NC_000963_1_Rickettsia_prowazekii_str__Madrid_E_chromosome_complete_genome probe_cstm_Rprow_021218_1x_104260 ATAAACTTCTGAAATTTTACTATGTATATTATTTAAAAATCTATCAAATAAGTAATTTACATGCTGAGTATCTTTTTCCATCTCATTATATGCTATTTCAGAAGCTATGCCAAGACCTAC 600720 600840

NC_000963_1_Rickettsia_prowazekii_str__Madrid_E_chromosome_complete_genome probe_cstm_Rprow_021218_1x_104261 AATTAAAGGAGTAGGCAAAGTACCTGAACGCATTCCTCGCTCTTGTCCACCACCATTTATTAATGGTGTAACACGAACACGAGGtttttttCTTATATATAATGCCCCTATTCCTTTCGG 600840 600960

NC_000963_1_Rickettsia_prowazekii_str__Madrid_E_chromosome_complete_genome probe_cstm_Rprow_021218_1x_104262 GCCATAAATTTTATGTCCTGAAATACTTGCAAGATCAATATTGCACTCGTTAACATTAATTGGAATTTTACCGAATCCTTGAGCAATATCGGAATGAAAAAATACATTTCTTTCACGACA 600960 601080

NC_000963_1_Rickettsia_prowazekii_str__Madrid_E_chromosome_complete_genome probe_cstm_Rprow_021218_1x_104263 AATTTTTCCAATTTCCTTTAAAGGTTGAATAACACCTATTTCATTATTCACTGCCATAACTGACACTAATAAAGTCTGATCAGTAATTGCATTTTTGAGGGTTTCTAAATCTATTATTCC 601080 601200

NC_009488_1_Orientia_tsutsugamushi_Boryong_complete_genome probe_cstm_Rprow_021218_1x_1043 TTCATTATTACTTCAAATCAGATCTTTATAACACCATTTTACATCATTGTATAGTTTCGAAAGAAGTCTAATGAAGTTTGCGTATAAAATATCCTGTTAGTAAATCTTATGATGCTATTG 125040 125160

NC_009488_1_Orientia_tsutsugamushi_Boryong_complete_genome probe_cstm_Rprow_021218_1x_10431 CAATAAGGAAGCAACTAGAAGCATTAACAGCTTAATAAATCAGCTTAGCAGACCAAATGATAAATCAGCAAGCATAAACTTAAAAACTAATGAAGATTTAAATAAAGAAAATCCAACTAT 1251600 1251720

NC_009488_1_Orientia_tsutsugamushi_Boryong_complete_genome probe_cstm_Rprow_021218_1x_10432 TTTAAGTAAAATTTGTGGTTGGTTTAAGTCTATAGTAACTGATGTAGGAGACAGATTTCATAAAAACGCTAAATATTATCAGTACGATAAAAAACCAGAGCAATTAGCTGACATTAAGAT 1251720 1251840

NC_009488_1_Orientia_tsutsugamushi_Boryong_complete_genome probe_cstm_Rprow_021218_1x_10433 CGAACAACCTAATATAGTTAATGATATAAGAAAATCAGAAATAAGTCCAAATAATGAATTAAAGGCTACAAAGACCGCAAACGATATTTCAACTACAGAAAAAATCAACACAAAGCCTGA 1251840 1251960

NC_009488_1_Orientia_tsutsugamushi_Boryong_complete_genome probe_cstm_Rprow_021218_1x_10434 ACAACAAAATCCAAGAATTAGTATTCGAAGGTAAAATGAGCTGCATAGAGCAGCTCAAGGGTAATAAAAATACGACTTTGCTAAGCAGAATGCGAAAGTCCTGAAACTTAGAGAGATTCG 1251960 1252080

NC_009488_1_Orientia_tsutsugamushi_Boryong_complete_genome probe_cstm_Rprow_021218_1x_10435 TTAACAGAAATACTATCTCAACAATCACTCAGCAAAAATATTATACCATTTAAACGGTAAAAATACAAATAAATTTAGCGAAGGTGAGGTTCTGTATGACTGATAAAATCAATAATTCAA 1252080 1252200

NC_009488_1_Orientia_tsutsugamushi_Boryong_complete_genome probe_cstm_Rprow_021218_1x_10436 ACAATCATAATTTAATGGAAGAATTGAGAAATAAAATAGCTTCTCATGCAGAAACCATAGCCTGTGATTTGCTTGGAGAGCCTAATAAACATTTCTCTAGACGTGGAGAAATACGTTGGG 1252200 1252320

NC_009488_1_Orientia_tsutsugamushi_Boryong_complete_genome probe_cstm_Rprow_021218_1x_10437 GAGATACAGGGAAAATTGTCGTCAATACTAGTGGCAAGCATGCTGGAAAATGGTATGACTTTAGTTCTGGCGAAGGTGGAGATTTATTTGACTTAGCCAGAAAAGAACGTGGCGGTGATT 1252320 1252440

NC_009488_1_Orientia_tsutsugamushi_Boryong_complete_genome probe_cstm_Rprow_021218_1x_10438 TTGTTAAAGCCAAGGAATATCTAACAAGCATGGTAGGAATAACAAGTTATAAGCAGAATTACCAACGTAAAGAAACCACTCAAAACGCTGCTCAAAATGAGCTAGCTAAAATTCGAAAAG 1252440 1252560

NC_009488_1_Orientia_tsutsugamushi_Boryong_complete_genome probe_cstm_Rprow_021218_1x_10439 TTCAATACTTTTATAATCAGTCTTCTCCATTATATTTTACTAATAATACTGAAGTTCAAATCGTAAAAAGATATCTTGAACAACATAGAAGAATTGATTGTTTTACAATGAATTCTGACT 1252560 1252680

NC_009488_1_Orientia_tsutsugamushi_Boryong_complete_genome probe_cstm_Rprow_021218_1x_1044 TCGCtttttttATGCTCAAACCTCATTGTAAATAACTATAGTTTATAATTCTAGAATAATAAAACATTCTTAGCTTATGAACATCTGATTCTCTGTGCCTTGATTCTAAAAATTAATCAG 125160 125280

NC_009488_1_Orientia_tsutsugamushi_Boryong_complete_genome probe_cstm_Rprow_021218_1x_10440 TGAGAGCAAGCGTAATTCTTGATAGAGAAACGAATGAAAATTATCCAGCATTTTCAGCATTTGCTAGAAATGCGAAAGGTGAAATTACTGGTGTACAAGTTGTATATTTGAATTCGCAAA 1252680 1252800

NC_009488_1_Orientia_tsutsugamushi_Boryong_complete_genome probe_cstm_Rprow_021218_1x_10441 CGTGCGATAAGGCAGATATTTCAGTCCCTAGGCGAGCTTTTGGCAAAATTTAGCGAATCGTTTGTTAGAATTAGTACATTGGCACCACATGATTCGCCTATAACAATCATAACAGAAGGC 1252800 1252920

NC_009488_1_Orientia_tsutsugamushi_Boryong_complete_genome probe_cstm_Rprow_021218_1x_10442 GTTGAAACAGCTTTAAGTCTAAAACAAGCAGGAATTAATGGAAAAATTATCGCTGCTGTTGGTATACATAATTTCAAGAACTATCAACCATTTGAAGGAGAAAAAATAATCATAGCGGCA 1252920 1253040

NC_009488_1_Orientia_tsutsugamushi_Boryong_complete_genome probe_cstm_Rprow_021218_1x_10443 GATAATGATGGACAAAATTCTATAACAATGAATACTGTTGATAAAGCGaaaaaaaCACTCGAAAATAGTGGAGCAAAGGTGTTAAAAGTGATGCCAACACAAGAAGGTGATTTCACGACC 1253040 1253160

NC_009488_1_Orientia_tsutsugamushi_Boryong_complete_genome probe_cstm_Rprow_021218_1x_10444 TATTACAAATTCATGGGGCTGAAGCTATTAGACAGATCATAATACCTGAAATTGCTAAACTTACTAAATTTAATGAAATACAAAGTGAATTAATAACGAATCAAAGTGAGGTTAAAGAAT 1253160 1253280

NC_009488_1_Orientia_tsutsugamushi_Boryong_complete_genome probe_cstm_Rprow_021218_1x_10445 TATATGCAAAATCTTTACCCCTATACGACTATAACAAAAAGGAAAAAGCTAATGCGGAAGTAACAACAGTCAACAAATTTTTAGAAAATCATACAGAAATTTATAGTTCAAAAATCTTTG 1253280 1253400

NC_009488_1_Orientia_tsutsugamushi_Boryong_complete_genome probe_cstm_Rprow_021218_1x_10446 ACAATCCTAATTTAAGAGCAAATATGGTTTTTGATGAAAAGACTCAAAAATCCTGGCCTGCACTCACTATTTTTGTTAAAAATGACAAAGATGAAATTACTGGAGCTAAGATATTAGCCT 1253400 1253520

NC_000963_1_Rickettsia_prowazekii_str__Madrid_E_chromosome_complete_genome probe_cstm_Rprow_021218_1x_104467 ACTCTGTTTTGTCCAAAATACAAATTTAATAACGCATAGAGCCAATGGTCAAAAAACGTGGTTAAATGGAGGTATTACCGGaaacacataaataaaaagatggatagtttacaaattaaa 625560 625680

NC_009488_1_Orientia_tsutsugamushi_Boryong_complete_genome probe_cstm_Rprow_021218_1x_10447 TGAATTCAAAAACATGTAATAAAGCGGATGCAGCTGAAAATTCTGTTGGTACAATTAGTGGATCATTTGTTGAAATTGCTCAACAAAATTCAGACTACTCTTCTATAACAATTCTTACAG 1253520 1253640

NC_009488_1_Orientia_tsutsugamushi_Boryong_complete_genome probe_cstm_Rprow_021218_1x_10448 AGAATATTGAAACTGCTCTAAGCATTAAACATGCTGGAGTCGAAGGAAAAATCTTATGTGCCATTGAAGCCCAAAATTTGCAAAACTATAATCCTGCCTCAAAAGAAAAGATCATTCTAG 1253640 1253760

NC_009488_1_Orientia_tsutsugamushi_Boryong_complete_genome probe_cstm_Rprow_021218_1x_10449 CAGTTAAAAATGACGTAAATACTGAAAAAGCAGAAAAAGTTTTAGATGATAAGGGAGCAACAGTCTGCACAGTCAAAAATGACTTCAATAATCTATTAAAAACTCAAGGGTTATATGCTG 1253760 1253880

NC_009488_1_Orientia_tsutsugamushi_Boryong_complete_genome probe_cstm_Rprow_021218_1x_1045 TTATGATGCGGAGATTGCtttttttaattttttttGTAAGTTCATTTGCTAACTGGTATGCGTATACATTATCAGAATCATATTTGATAGCCAGGCTACAACTTTCAATTGCTTCTTGAT 125280 125400

NC_009488_1_Orientia_tsutsugamushi_Boryong_complete_genome probe_cstm_Rprow_021218_1x_10450 TTAGAAATATTATCAGCCATGAAATAAGAAAACTTACTGAAAAGAATGAATCCATACAAACTAATATACAACCAAGATTATGTCTGAAAATTTAAAACACGATGGGCTATTTAAAGATTT 1253880 1254000

NC_009488_1_Orientia_tsutsugamushi_Boryong_complete_genome probe_cstm_Rprow_021218_1x_10451 AATGAATGAACCAAAAGCAGCTCTGGATTTTATAAATGACTTTTTACCAAATGAAGTTAAAAACGTACTAGATTTAAATACTATAAAAGTTGAGCAAGAATCGTTTGTTGAAGCCAATCT 1254000 1254120

NC_009488_1_Orientia_tsutsugamushi_Boryong_complete_genome probe_cstm_Rprow_021218_1x_10452 ACGCCGTAGTATGTGTGACGTACTATTTTCAGTTAAAACAAAAAATAATAATAATGCATTTATATATGTACTTATTGAGGCAGAATTAAGATCTGATTATTGGATTGCGTTCAAATTATG 1254120 1254240

NC_000963_1_Rickettsia_prowazekii_str__Madrid_E_chromosome_complete_genome probe_cstm_Rprow_021218_1x_104529 CTCTTGAGTGCTTTATTAGCTTTAGATGAAGATAAAACTTTTTCTACAACTTTTTGATAGTTTTTAGTATTTACGACAGCCataaaattaattttaacaaaaattctaaatctattttaa 633000 633120

NC_009488_1_Orientia_tsutsugamushi_Boryong_complete_genome probe_cstm_Rprow_021218_1x_10454 TGCTACGAGAAGCTTATGGGAACTGTTTGATGATCCTAAATTAGCTAAAGGAGTTGATGAGCTCTGAATACTTATTAATCGATTGGCAAGCAATGCCAGATAGTGAAATAAAAAGAAAAG 1254360 1254480

NC_009488_1_Orientia_tsutsugamushi_Boryong_complete_genome probe_cstm_Rprow_021218_1x_10455 CAACTGCTGCACTAGTTCATTTTATGAAGTATATTCATAATCAACAGGATATAATAGAGCTATGGGCAAAATTTTTTAATACACTACACGAAATAGCACAAAAAGATAAAGAAAATGGCT 1254480 1254600

NC_009488_1_Orientia_tsutsugamushi_Boryong_complete_genome probe_cstm_Rprow_021218_1x_10456 TTCTTTACATAAAAGCGTTATTGCATTATACTATAAGTAAGGTTAGTAAAAATGAGCAACCTAGGTTAAAACAATTACTGGACGAAAATTTATCAATTGAGGATAGAAAAAGGATTATGG 1254600 1254720

NC_009488_1_Orientia_tsutsugamushi_Boryong_complete_genome probe_cstm_Rprow_021218_1x_10457 AAACAATTGCTGCACAATATATTGATGAAGGCATAGCTAAAGGCAGAGCTGAAGCTGCACAAGGGCTTGCAAGGAACTTATTAAACGCTGGCTTTTCAGTTGAATTTATTGCTGAAAATA 1254720 1254840

NC_009488_1_Orientia_tsutsugamushi_Boryong_complete_genome probe_cstm_Rprow_021218_1x_1046 GTCGCCCCAATTCATTCAAAGCAATTCCTTTATTATAGTAAGCCTCTGGAAAATCTGGTTGATACTTAATAGCCAAATCAAAATTCTCTATTACCTCTTGATATTGTTCTAGTATACATA 125400 125520

NC_009488_1_Orientia_tsutsugamushi_Boryong_complete_genome probe_cstm_Rprow_021218_1x_10460 ATTATTCATATTTACTCCATCTCGCATAGTATTTGATCCATATGACAAGTTTTCTTGAGTTTCAATTATCTCTTGCTCACCTAGTGTTAATGCTGATTTATAAGCTGTAACCTGATCGCT 1255080 1255200

NC_009488_1_Orientia_tsutsugamushi_Boryong_complete_genome probe_cstm_Rprow_021218_1x_1047 AAGCAGATCCTTTATTATTGTAAGAGCCTGCGTAATTTGTGCTGTATCGAATAGCTTGATCATAATTGTTAATTGCTTCTAGTAGCTTTCCAAGATTTTTCAAAGCATTTCCTTTAGCAT 125520 125640

NC_009488_1_Orientia_tsutsugamushi_Boryong_complete_genome probe_cstm_Rprow_021218_1x_10471 TAGCTATTAATTCTGATGAGATCTCTAAAATTGTGACTCCAGAAATAGCTTCTAATGTGATATAATCATAAATTGGGAAATATACTCTCCAATCCGAAGATAAAAAGGCTATTTCAGAGT 1256400 1256520

NC_009488_1_Orientia_tsutsugamushi_Boryong_complete_genome probe_cstm_Rprow_021218_1x_1048 GATAAGCTTCTATCAAATCTGGTTTATACTTAATAGCTATATCATAATTTTCAATTGCCTCTTGATTGCGCCCTAATTCCTTTAAAGACGCTCCTTTATTAACATAAGCTTCTACAAAAT 125640 125760

NC_000963_1_Rickettsia_prowazekii_str__Madrid_E_chromosome_complete_genome probe_cstm_Rprow_021218_1x_104889 TTGGAAATGATTTTATTAAAGTAACATATTACATTAGTAGCAAATTTAAGACAAGTACTTAAAGACCAAGGACTTGTCAGAacaatacacaaaaaatcaaagtaaacaaaataagtatat 676200 676320

NC_009488_1_Orientia_tsutsugamushi_Boryong_complete_genome probe_cstm_Rprow_021218_1x_1049 CTGGTTTATACTTAATAGCTACATCATAATTTTTTATTGCTTCTTGATATTGCCCTAGTTCGTATAAAGAAGTTCCTTTACCAATATAATAGTCTGCATCATTTGGATTGTATTGAATAG 125760 125880

NC_000963_1_Rickettsia_prowazekii_str__Madrid_E_chromosome_complete_genome probe_cstm_Rprow_021218_1x_104974 AATTGATACTTCTTGTTCTGCACATTTTAAATATATTTTATTGTTCTCAGCTTTAATTATTTTACCTTGGATAAAGTGTTTTTCCATTCAATAATTCTTTCAGTTTGATTTTAACTTCTC 686400 686520

NC_000963_1_Rickettsia_prowazekii_str__Madrid_E_chromosome_complete_genome probe_cstm_Rprow_021218_1x_104975 TTCCTAAAAACCTATTATAATTTTCAAATTTTACTAACGTACGTTCAATACCACTTGATGATACCTCTAAAGAATACGCCTCCTCTATTAAATCTTCAACATCTAGAATAGCAGAAATAG 686520 686640

NC_000963_1_Rickettsia_prowazekii_str__Madrid_E_chromosome_complete_genome probe_cstm_Rprow_021218_1x_104976 TCCTACTTACGTTAGTACAATCTTCTATAGATATTTTGTTACCATTTAAGCTATCAATCAATACCTCAACTACTTTAGGACTAACACCTTTAAACTTCACAAGAACTAACTCAAACCCCA 686640 686760

NC_000963_1_Rickettsia_prowazekii_str__Madrid_E_chromosome_complete_genome probe_cstm_Rprow_021218_1x_104977 TATCCGTTAAGGATTCTTCTATTATGTTTGTTATTTGTTGTGCAATAGTCTGCATATGTGATGTTTATATAATAAAAAAGGTGGAAAACCCACCCTGCTGTTTATAATTTATATAATGCT 686760 686880

NC_000963_1_Rickettsia_prowazekii_str__Madrid_E_chromosome_complete_genome probe_cstm_Rprow_021218_1x_104978 TATCCGTAATAAAAATACACTAATTAGATTATATAAGCAATATTTAACTTTATATTTTTATAAAACTACATACTATAAATTGCAAATACTCTATAAATCACTCTTACACACTTAGAGGCT 686880 687000

NC_000963_1_Rickettsia_prowazekii_str__Madrid_E_chromosome_complete_genome probe_cstm_Rprow_021218_1x_104979 TCTTATCCTGATTTGATACTATATTGAGTCGTCATACTACTATTTCCTCTGCTAATAATGTATGTTGTACATGATTATAAAAAGAATGAGGGCTACAAATTTAATAGATTTTCCCTTTTG 687000 687120

NC_000963_1_Rickettsia_prowazekii_str__Madrid_E_chromosome_complete_genome probe_cstm_Rprow_021218_1x_104980 aaaaaaaaGCCTAATAAAAATTAGACAGTGATTATACAAGTATAATAATTATAATACTAATGAAATTAGTCAACTAAAATATATCATTATCTCATTTGCAATATCACATATTTATTACTG 687120 687240

NC_000963_1_Rickettsia_prowazekii_str__Madrid_E_chromosome_complete_genome probe_cstm_Rprow_021218_1x_104981 CTATATCAGTTAATTTTATTGCATTCATCACGATTTCTAATAGCATGAATAACTTCTACAACTTAGGATTTGGCTCATTTGTTTTCTAGTAATAAGATGACTTTCTTACCAAAAGTGCGT 687240 687360

NC_000963_1_Rickettsia_prowazekii_str__Madrid_E_chromosome_complete_genome probe_cstm_Rprow_021218_1x_104982 TGATAAGCATAAGTGCTTTATTATAGAATAAAAAATAATCTTTTTCATCAACACTGTTTGATATCGCAAACTCACTATATTTTACTAACTTTAACAGCAATAATTTTGCACAATATATTT 687360 687480

NC_000963_1_Rickettsia_prowazekii_str__Madrid_E_chromosome_complete_genome probe_cstm_Rprow_021218_1x_104983 ATTATGTATTACTTTTTGTTCATTTTATCCATAAAGGACTTTCTACTGCTATATTAGAATTTCATAATATTCTTTCCTTTTCAAGGCTTATAAAATACTTCCTACTACTAGTAGGATATA 687480 687600

NC_000963_1_Rickettsia_prowazekii_str__Madrid_E_chromosome_complete_genome probe_cstm_Rprow_021218_1x_104984 TTTCTTCTTACACAACCTATATTTGATAGAAGCTCTTTAATAACAGGTTACACTATAACTCTGTAAGAATCTACCTGTAAGattttaaatttgcattgcatttttatttattcttttctg 687600 687720

NC_000963_1_Rickettsia_prowazekii_str__Madrid_E_chromosome_complete_genome probe_cstm_Rprow_021218_1x_104985 aggtttttctatattaaaactattaATATTCAGTTTTAGTTGCTAAATTGAAATAAAGATTTTAAATTTCTTAAATAACGTAGCAATTTTTCTGATTCCTTTCTTGTTTTAATAATGATT 687720 687840

NC_000963_1_Rickettsia_prowazekii_str__Madrid_E_chromosome_complete_genome probe_cstm_Rprow_021218_1x_104988 agtaaatgaagaatatgtaatgattgataaaataatTCTCCCTATAAATTATAGGGAAATAGGCATATTTCTTTTTCCACATATTAAAAACTCTTTATTCCCCTTAGTACCTAATATAGG 688080 688200

NC_000963_1_Rickettsia_prowazekii_str__Madrid_E_chromosome_complete_genome probe_cstm_Rprow_021218_1x_104989 ACTTGCTATAATACCAAATATTTGAAAATGATGCTCTTGCTCAAGCCAATTCTTAATTTTATCACATACCTTTTGATGTAAGAGAGGATTTGTAATAATTCCTCCATTCTCTACTTCATT 688200 688320

NC_000963_1_Rickettsia_prowazekii_str__Madrid_E_chromosome_complete_genome probe_cstm_Rprow_021218_1x_104990 CTTTTTGACCTCAAACTGTGGTTTTATTAAAGCAATAAGAATGCAATCTTCCTTAGCTAAATTTAGCGGAGTCGGTAATATAGTAGTTAAGCTAATAAAACTAGCATCGCAAACAATTAA 688320 688440

NC_000963_1_Rickettsia_prowazekii_str__Madrid_E_chromosome_complete_genome probe_cstm_Rprow_021218_1x_104991 ATCAGGCTTCGTTGTAATTTGTTTATCTGTTAAATATCGTGCATTGGTCTTTTCAAGCACTTTAATTTGTGGATTAAATCGTAATTTAGAATGAAGTTCACCATAACCTACATCTACGGC 688440 688560

NC_000963_1_Rickettsia_prowazekii_str__Madrid_E_chromosome_complete_genome probe_cstm_Rprow_021218_1x_104992 AAAAATTAATTCTGCTTTACGCTCAAATAATACTTCGGTAAAACCACCAGTGCTACTACCGATATCAATACAAACTAAATTTTGGGGGTCAATTTTAAAATAATCCAAAGCTGTAATTAA 688560 688680

NC_000963_1_Rickettsia_prowazekii_str__Madrid_E_chromosome_complete_genome probe_cstm_Rprow_021218_1x_104993 TTTTAATGCCCCTCTTGAAACATAATTATGCTGCGGTAGCTTTACCTTAATATCTGGATCATGTCTATTCACCTTAATCCCAGACTTAATTAATTTCTCATGTTTGTTATGTACTTTACC 688680 688800

NC_000963_1_Rickettsia_prowazekii_str__Madrid_E_chromosome_complete_genome probe_cstm_Rprow_021218_1x_104994 TTGGATAATCAAGCTTCGTGCTATAGCAATATCTGTTACAAGACCTTTTTGCAGCAAATATTCATCAAGTCTTATTTTAGTCATTACTTTTTAAACCTAAATTTAAGTTATGTTTAAGGA 688800 688920

NC_000963_1_Rickettsia_prowazekii_str__Madrid_E_chromosome_complete_genome probe_cstm_Rprow_021218_1x_104995 TGCATAAGATAGAAAATATACAATATGTTTAATTGAAAATGAGAATTTTTAATTTTAAGTTATACGCATTTTACAACTGAAGTATATATCGCAATAAAGAAAAATATTTAAACCTTAACT 688920 689040

NC_000963_1_Rickettsia_prowazekii_str__Madrid_E_chromosome_complete_genome probe_cstm_Rprow_021218_1x_104996 AATATATGTCTTTTCTTACCAACAGAGAGCTTTATAACTTTTCTGTTACGCAAGAAATTAGTATCAATTGTCATATTCTCATCTTCAACTAATTGATCATTTATTTTAGCTCCCTTGCCG 689040 689160

NC_000963_1_Rickettsia_prowazekii_str__Madrid_E_chromosome_complete_genome probe_cstm_Rprow_021218_1x_104997 CGTATAATCTTACGTGCCTCTGACTTAGATCTAGCAAGATTTGCGTTGTAGAATAAGTTATATGCACTAATCCCGGATTGTAATATTTCTGGCGCTAAAATAAATGTATGTAGATTTTCA 689160 689280

NC_000963_1_Rickettsia_prowazekii_str__Madrid_E_chromosome_complete_genome probe_cstm_Rprow_021218_1x_104998 TCGATATCTCCTTGCTCAAATATCTTTACTGCAGTTTCTAGGGCTAATTTAGCTAATCGCTCGCCGTGGCAAAGCTTCGTCAATTCATAAGCAAGTTGTTTCTTAGCAGCATTAATATCT 689280 689400

NC_000963_1_Rickettsia_prowazekii_str__Madrid_E_chromosome_complete_genome probe_cstm_Rprow_021218_1x_104999 TCAGATACCAAGCTTTCAAATTTATTAAGCTCTACTATATCTAATTCACTATATAATTTTGCAAATCTCATTACATCAGCATCTTCACAATTACGCCAATATTGGTAATAATCATATGGG 689400 689520

NC_009488_1_Orientia_tsutsugamushi_Boryong_complete_genome probe_cstm_Rprow_021218_1x_105 ATAAACTGTTATTAATTGAAACTATATTTAAAGATATTACATAATATCTCTTGTAGACCTTTTGTATTATGCCTTTCATACTTTTTCTTATCCTAAACTGACGTATTCAGAATAAAAACT 12480 12600

NC_009488_1_Orientia_tsutsugamushi_Boryong_complete_genome probe_cstm_Rprow_021218_1x_1050 CTGTATCATAATTCTTTATTGCCTCTTGATATTGTCCTAGTTCGTATAAAGAAATTCCTTTAGCATAATAAGTTTCTGAAAAATCAGGTTTATATCAAATAGTAGTATCAAAAGTTTCTA 125880 126000

NC_000963_1_Rickettsia_prowazekii_str__Madrid_E_chromosome_complete_genome probe_cstm_Rprow_021218_1x_105000 CTTAACAGATCTTCATTAAGCCATACTGCACCTGCAGCAGTTTTGCCCATTTTAGCACCTGAAGCGGTTGTAAGTAGCGGCGTTGTCATACCAAATACTTCTTTACCACTGATTTTACGT 689520 689640

NC_000963_1_Rickettsia_prowazekii_str__Madrid_E_chromosome_complete_genome probe_cstm_Rprow_021218_1x_105001 ATTAAATCAGCACCAATAACTATATTGCCCCACTGATCACTCCCCCCAAGTTGCAGGATACAATTATAATTCTTACTTAAATAGTAAAAATCATATGATTGCAATAACATATAATTTAAT 689640 689760

NC_000963_1_Rickettsia_prowazekii_str__Madrid_E_chromosome_complete_genome probe_cstm_Rprow_021218_1x_105002 TCTAGAAAACTTAAATGATGAGAACGGTCAAGCCTAAGTTTTACCGAATCCATAGTTAACATACGGTTTACCGAGAAATAGCTACCGAAATCACGTAAAAAATCTAGGTAATTGAGCGAA 689760 689880

NC_000963_1_Rickettsia_prowazekii_str__Madrid_E_chromosome_complete_genome probe_cstm_Rprow_021218_1x_105003 TCTAACCATTCTGCATTATCGAGTATTATTGCATCACCTTGATCTTTTCCAAACTTGATAAACTTTGACAATGATTTTTTAATCCCTTCAGCATTTCTTTTAATATCCTCTTGTGTTAAA 689880 690000

NC_000963_1_Rickettsia_prowazekii_str__Madrid_E_chromosome_complete_genome probe_cstm_Rprow_021218_1x_105004 GCTTTTCGTGTTACATCTTTACCAGCAGGATCACCGATTTTACTCGTGCCACCCCCAATAATCACAATAGGGGTATGTCCGTGTTTCTGAAGTAACCGTAATATCATTATCTGCATTAAA 690000 690120

NC_000963_1_Rickettsia_prowazekii_str__Madrid_E_chromosome_complete_genome probe_cstm_Rprow_021218_1x_105005 CTACCGATATGTAGTGATGTAGCAGTACAATCAAAACCTATATAAGCAGCAATCTTTGCTTCTTGTGTTATAGCACTTAACCGAGCCAAATCAGTACATTGATTAAGATATCCTTTGTAT 690120 690240

NC_000963_1_Rickettsia_prowazekii_str__Madrid_E_chromosome_complete_genome probe_cstm_Rprow_021218_1x_105006 ATAAATTCTTCAATAAAGGTCATATTATTTATATTGTTTTTGGTTTTTCTATAAACAGTTTTCTCTACAGACACTTTATGGTCTTTTTAGAGATTTTGACAAATAAAGCTATTTTCCTGC 690240 690360

NC_000963_1_Rickettsia_prowazekii_str__Madrid_E_chromosome_complete_genome probe_cstm_Rprow_021218_1x_105007 TAATAAATTTATTGCTGCTTTTTAAAATTAACCTTAAGGCCTTATTTTAAAGGGTCTAGTTTTATAGCAATAACATTACCATTATATTTAATTTATTATAAAGTATATGAAAACTTTGTC 690360 690480

NC_000963_1_Rickettsia_prowazekii_str__Madrid_E_chromosome_complete_genome probe_cstm_Rprow_021218_1x_105008 TATACTTGATATATTGATTATCATACAAGCTCATATTAATTAACTCACTTAATTTATAAATCATGTTAATATAATATATAGTGTCGTCATAAAGATATGATAATATTCAATAATGTATTG 690480 690600

NC_000963_1_Rickettsia_prowazekii_str__Madrid_E_chromosome_complete_genome probe_cstm_Rprow_021218_1x_105009 AATTGAACAATTTTACGTTACTCACACACTAGGCATTGCATGTGTAGATAACTACATAATTGCAAGCAACTATAATCTGTGCTGAATGACtttttttATATCATTACTGTAAGGTTTTAT 690600 690720

NC_000963_1_Rickettsia_prowazekii_str__Madrid_E_chromosome_complete_genome probe_cstm_Rprow_021218_1x_105010 TGCATCAGTAAAACCTGCTGTGGTCACCTAGTGCTATTAAGTATTAGGCCTATAAAAATAATAATGTTGAGTATTTTGCTAGACACTAAGGATAGAGACGAAGTTGAGCACACAGTAATA 690720 690840

NC_000963_1_Rickettsia_prowazekii_str__Madrid_E_chromosome_complete_genome probe_cstm_Rprow_021218_1x_105011 TGATTACATAACAATCTGCCATAATAATCCAATCGATAAAATGCCTTCACAGGATAAATGAAAATATTACTGACCTGAATCGGATCAGATCTTTCATTTTACATTTCTCTATACCATGAC 690840 690960

NC_000963_1_Rickettsia_prowazekii_str__Madrid_E_chromosome_complete_genome probe_cstm_Rprow_021218_1x_105012 TACTTGGTACTACAACAACAATATTCCAGGACCTTTGCGTCTACTCCAATATACTAAACTTACTTAATTTGATAAAGTATTACACTTTATCGCTTTCAGAATAAGAGATTTCAGAATGAG 690960 691080

NC_000963_1_Rickettsia_prowazekii_str__Madrid_E_chromosome_complete_genome probe_cstm_Rprow_021218_1x_105013 GAAAGTAGAAACTTATTTGAAAGCTGAACTACTAAAACATACACCGAAACAAGTACAATAAGTCAGATTCAGATACTGTAACATATGGGAGTTATATTTTTGCTTCTAATAAAGTCGTAC 691080 691200

NC_000963_1_Rickettsia_prowazekii_str__Madrid_E_chromosome_complete_genome probe_cstm_Rprow_021218_1x_105014 ATAACTTACAATTAAATTACCTTTGAACTACTTTACCTCAACACAAAAGTTCATTAACTCTATTTGCATCTATATTCTCTGCTTATACATGAATTAATCTAAATTAAGTAAAATGTGATT 691200 691320

NC_000963_1_Rickettsia_prowazekii_str__Madrid_E_chromosome_complete_genome probe_cstm_Rprow_021218_1x_105015 ATTTATTATTTGAAACATCAGAACATGCTATCAGAACATGCTATATTTCTTGACAAATGCTATGCTTTTATCTATACTTCTTTAGTATTATTAAGTATTAATATACAAGTACTTCAAGCT 691320 691440

NC_000963_1_Rickettsia_prowazekii_str__Madrid_E_chromosome_complete_genome probe_cstm_Rprow_021218_1x_105016 TAGCACCCGTAGCTCAATTGGATAGAGTATATGACTACGGATCATAAGGTTAGGGGTTCGACTCCTCTCGGGTGCGCCACCATTATTGATCTATCTGTATTCCCCAAGTGTATAAAACTA 691440 691560

NC_000963_1_Rickettsia_prowazekii_str__Madrid_E_chromosome_complete_genome probe_cstm_Rprow_021218_1x_105017 AAACGCATTATAAAGGTAGATTTTCAGTTTTAGATATTAAGTTAGCAAAAACTAGTAGCAAAAGATTAGAACAATATGATACTACGAATATAATTAATTACGCTAGTCATAATATTGAGA 691560 691680

NC_000963_1_Rickettsia_prowazekii_str__Madrid_E_chromosome_complete_genome probe_cstm_Rprow_021218_1x_105018 TTTTTCTTCTAAATTTTTAGCACTTTAATTCTATTTACTTTTACCATCTTATATAAAAGAGTTTTAGTATAATAAAGTATTTAAAATAGTGCATGACTTAATCTACTAACATATACTAAT 691680 691800

NC_000963_1_Rickettsia_prowazekii_str__Madrid_E_chromosome_complete_genome probe_cstm_Rprow_021218_1x_105019 AATGCGTTGATCATAAATAAATATGGCATATCCTTCACCGATATTATTAAACCTATTAAGAACAAAAACATTGTGAGATATTCAAATCAAAGTTATTGAAATAACAATTATAGCTAAATT 691800 691920

NC_000963_1_Rickettsia_prowazekii_str__Madrid_E_chromosome_complete_genome probe_cstm_Rprow_021218_1x_105021 TAACACTGCTAAAAAGATATAAATAATCTCTTTATTGTTCAAATAGCATCTATAATTGTAAAATAATACTAACCTTAATGATAGATATCATAAATCACCACAAATCCGATATTATTGATT 692040 692160

NC_000963_1_Rickettsia_prowazekii_str__Madrid_E_chromosome_complete_genome probe_cstm_Rprow_021218_1x_105022 AATAAGGATCTAATATTATCAATCGAAATACTATGACAGTAATATAAGCTATAGACAGTAAAAACCTTTAAATATTAGAGAAAGATTGAAAGGTATTGCAaatatatatttgaataatat 692160 692280

NC_000963_1_Rickettsia_prowazekii_str__Madrid_E_chromosome_complete_genome probe_cstm_Rprow_021218_1x_105023 aatatatatttgaacaatatatTGTAGTGCGAATGTCTTTAGTACTGGTATTATGATCACCTCATCACAATTCTTGTCCAAACAACaaaaaaaTTTTGTTTAATATATATCCTCATTCAT 692280 692400

NC_000963_1_Rickettsia_prowazekii_str__Madrid_E_chromosome_complete_genome probe_cstm_Rprow_021218_1x_105024 TGTTTTACTTAAtttttttAACATTTAATATCATAAATAATAAGCTATAGATATGAAAAATATTTACTGCAAATTTGTATTGACTATTTTCTTATGTTTAATAAATTTACAAGTAATCGC 692400 692520

NC_000963_1_Rickettsia_prowazekii_str__Madrid_E_chromosome_complete_genome probe_cstm_Rprow_021218_1x_105025 TACAAGCTTTAATGAAAAAATACCTGTATATTTAAAATTAACTAACGCAAAGATGTTAGCAGGGAAAAATTCGTTAAATATTGAGCTTTGTGATTATAGAATTAAAGAATGGTGTATAAA 692520 692640

NC_000963_1_Rickettsia_prowazekii_str__Madrid_E_chromosome_complete_genome probe_cstm_Rprow_021218_1x_105026 GCCACAAAGAGAGATGGGCCTTAACGGGCAAAAAATAAATGAGTATATATCTATTTCACCTGATATTAAAGGAGAATGGAGATTCGGCTGGAGTTATAATATATATTTTATACCCGAAGA 692640 692760

NC_000963_1_Rickettsia_prowazekii_str__Madrid_E_chromosome_complete_genome probe_cstm_Rprow_021218_1x_105027 AAACTTTTTACCTAATCAAACTTATAAAATTACTATCAAAGATTATATATTTCCTAATTTTATTAGTTTaaaaaaaaaTAATATAAGCTTCACGACTTTACCGCTACTTTCTATGATTAA 692760 692880

NC_000963_1_Rickettsia_prowazekii_str__Madrid_E_chromosome_complete_genome probe_cstm_Rprow_021218_1x_105028 AGAAATGAATTATCTACAAGATAATATCGATATTTCTAAAAAATTTATCCAAACGAGAATAGCTTTTAACTATCCAATTGATCCTAAAACTCTAAAAGAAAGAATAAATTTCATCAAATC 692880 693000

NC_000963_1_Rickettsia_prowazekii_str__Madrid_E_chromosome_complete_genome probe_cstm_Rprow_021218_1x_105029 TTCCACTAAAGAAAAATTACCATTTTCTATCAAATTCAATACAGATAATACAGAAGCTATATCAATTACTAATATACCACCGCTTACAGATAAAAAAGATATAGTATCTATTATTATAAA 693000 693120

NC_000963_1_Rickettsia_prowazekii_str__Madrid_E_chromosome_complete_genome probe_cstm_Rprow_021218_1x_105031 TCTATCCTCATATTTTAAAATCACTAATAGTACTGCTACAATTGTTAAAGACGCAAAACTAAAACCGGAACAGATAATTATAATTACTACAAATACACCAGTTTCAGGTGAAGAGGTaaa 693240 693360

NC_000963_1_Rickettsia_prowazekii_str__Madrid_E_chromosome_complete_genome probe_cstm_Rprow_021218_1x_105032 aaaaCATTTAGAGCTATTTTTATTACCAAAAAATAAACCGCATTTCTTAGGAGTTACAGGTAAGAAAAACTATAAATGGCAAAGTCCAAAAGAAATAACGGATGATATTCTTAAATTAAG 693360 693480

NC_000963_1_Rickettsia_prowazekii_str__Madrid_E_chromosome_complete_genome probe_cstm_Rprow_021218_1x_105033 TGAAAAAATAAATTTTGAACTACTGCCTTCTGTCCCGAAAATTACTACTGTACATAACTTCAAAGTAAATACGGTTGCCTCAAGAACTGTTCTCATTAAAATaaaaaaaGGGATTAGGAC 693480 693600

NC_000963_1_Rickettsia_prowazekii_str__Madrid_E_chromosome_complete_genome probe_cstm_Rprow_021218_1x_105034 ATCGGATAATTTAACGCTTGGCTCAGATTACACTCAAATAATACAAATACCTGATAATCCTAAAGAAGTGAAGCTCATGTCAGATGGATCTATTCTTTCATTATCTGGTGAAAAGAAACT 693600 693720

NC_000963_1_Rickettsia_prowazekii_str__Madrid_E_chromosome_complete_genome probe_cstm_Rprow_021218_1x_105035 TCCCGTATATTCACTTGGCATAGATAAATTATATGTAGAAATCGATAAGATTCATCAGCAAGAAATTAATCACTTAATAAGCCAGACAAATAGATATAATATTTTCCAAAACCCAACTTT 693720 693840

NC_000963_1_Rickettsia_prowazekii_str__Madrid_E_chromosome_complete_genome probe_cstm_Rprow_021218_1x_105036 CATAAATGAATATTCTTTTAATGAGTATAATATTTCAGATGTATTCCAAGAAGAGGTAATAATTAATTCTCCAAATCTCGATTTACCTCATTATACCAATTTAGATTTTGGTAAGTATTT 693840 693960

NC_000963_1_Rickettsia_prowazekii_str__Madrid_E_chromosome_complete_genome probe_cstm_Rprow_021218_1x_105037 TTATTCAGAGCAAGCAGGGAGATATTCTAAGGGACTATTTTTAGCAAAAGTGTACTTTAAAGATAAAAATAATAATATTATATCTCAAGATAAAAGATTAATTTTAGTTACTAATCTTGG 693960 694080

NC_000963_1_Rickettsia_prowazekii_str__Madrid_E_chromosome_complete_genome probe_cstm_Rprow_021218_1x_105038 TTTTATAGTAAAAACCGATAAAACAGGAACGCATCATATATTTGTTTCTTATATCAGTAATGGTAAACCTGCAGAGGGTGTAAAAGTAGATATTATAGGACTTAATGGTGAGATATTAGT 694080 694200

NC_000963_1_Rickettsia_prowazekii_str__Madrid_E_chromosome_complete_genome probe_cstm_Rprow_021218_1x_105039 CAGCAGTAAAACTGATAGCCAAGGACATACTATTTTATCAAACATACATGATTTAAATAAAGCTAAAATTCCAATAGCTTACGTATTAACTACTCGAGACGATTTCTCGTTTATGCCATA 694200 694320

NC_000963_1_Rickettsia_prowazekii_str__Madrid_E_chromosome_complete_genome probe_cstm_Rprow_021218_1x_105040 TAATAGAGTGGATCAGCAAGTTAATTATTCTCGGTTTGATATATCAGGGACTGTAAGTTCAAATGAAGGATTAAAAGCTTATTTATTTTCCGATCGCGGCATTTATAGACCAAGCGAGCA 694320 694440

NC_000963_1_Rickettsia_prowazekii_str__Madrid_E_chromosome_complete_genome probe_cstm_Rprow_021218_1x_105041 AGGCCATATAGGTATTATGCTCAAACAAAACGATTGGCATGGAAAATTTGATGGGTTACCTTTAGAAATCCAAGTTACTAATCCTCATGGGAAAGTAATAGATAAAAATAAAATCGTTCT 694440 694560

NC_000963_1_Rickettsia_prowazekii_str__Madrid_E_chromosome_complete_genome probe_cstm_Rprow_021218_1x_105042 AAACTCAGAAGGGCTTGGTGAATATTTATTCACAACATTTGATGATTCTTTAACAGGCTTATATAATATAAGCTTATATTTAGGTGATCAAGGAATAAATAATTATCTTAATAGCATATC 694560 694680

NC_000963_1_Rickettsia_prowazekii_str__Madrid_E_chromosome_complete_genome probe_cstm_Rprow_021218_1x_105043 TGTCAGAGTCGGAGATTTTCAACCTGACCGCATGAAAATAAATATAAACTTCAATAACCTAAAAGATACGTTATGGACTCATCCAAAAGATCTTAAAGCAACAGTTAAACTTATAAATCT 694680 694800

NC_000963_1_Rickettsia_prowazekii_str__Madrid_E_chromosome_complete_genome probe_cstm_Rprow_021218_1x_105044 TTATGGCATTCCTGCAGAAAATAGAAAAATTAGAGGTTTAATCGATATTAAACCTACAGAGTTTTTTGTCACTAACTTTAAAGAGTATACATTCTATCGTAGTAAAAGCAATGAAGAGTT 694800 694920

NC_000963_1_Rickettsia_prowazekii_str__Madrid_E_chromosome_complete_genome probe_cstm_Rprow_021218_1x_105045 TTTCAATGAGCATTTAGGAGATGTTACTACAGATTCTACAGGTACCGCAAATTTTGACGTTAACCTTGAGAAATATTATAATGCTACTTTTAATCTAACATTTTCAGCAGAAGGGTTTGA 694920 695040

NC_000963_1_Rickettsia_prowazekii_str__Madrid_E_chromosome_complete_genome probe_cstm_Rprow_021218_1x_105046 ACTTGACTCAGGAAGAAGCGTAACCAGTAGTAAATCTCTTATAATTTCACCTCTGCCTTATATTATAGGTTTTAGAAGCGATAGCAACTCTAAGTATATTAAGAAACAATCAATTTCAAC 695040 695160

NC_000963_1_Rickettsia_prowazekii_str__Madrid_E_chromosome_complete_genome probe_cstm_Rprow_021218_1x_105047 AATAAAATTTATAGCTATATCCAATAAAGCAGAAAAAGTATCTGCGCATAATTTAACTCTTAAGTTAAACAAGATTAATTACGTAAATAATTTAGTATCTGATGCTAATGGTAATTATTC 695160 695280

NC_000963_1_Rickettsia_prowazekii_str__Madrid_E_chromosome_complete_genome probe_cstm_Rprow_021218_1x_105048 TTACAATACAGTACCTGTTGAAACGAATATATCTTCCGATAATGTTAATATCACAGCAAACGAAGGTTATATTTATAATGTGCCTACTAAAGAAGAAGGCGATTACGTTATTTACCTAAC 695280 695400

NC_000963_1_Rickettsia_prowazekii_str__Madrid_E_chromosome_complete_genome probe_cstm_Rprow_021218_1x_105049 AGATACAAAAAATAGAGTATTTGCTCAAACTGAATTTTCAGTAATAGGTGAAGGAAATGTTACAGCTAATTTAACAGATAAAGCAAATTTAAAAGTTAAACTTGATAAAGATGATTATAA 695400 695520

NC_000963_1_Rickettsia_prowazekii_str__Madrid_E_chromosome_complete_genome probe_cstm_Rprow_021218_1x_105050 AGCCGGTGATACTATTCTTTTAAATATTAAAACTCCTTATACAGGTCATGGATTAATTACTATTGAGACTGATAAAGTACATAATTTTAAATGGTTTAAAACTGATAAAAATAATAGTAT 695520 695640

NC_000963_1_Rickettsia_prowazekii_str__Madrid_E_chromosome_complete_genome probe_cstm_Rprow_021218_1x_105051 TCAAGCAATAAAAATACTGACGGTTTTGAAGGTAAAGGATACGTAAATGTGCAATTTATAAGAGACATCACAGCTACGGAAATCTTTATGTCACCGTTTAGCTATGCAGTACTACCGTTT 695640 695760

NC_009488_1_Orientia_tsutsugamushi_Boryong_complete_genome probe_cstm_Rprow_021218_1x_1051 TTGCTTTTTGATGTTGCCATAATGCCTTTAAAGCCATTCCTTTATTAATATAAGCTTCTATACAATCTGGATTACACTTAATAGCTATATCAAAATTCTTTATTGCTTTTTGATATTTTC 126000 126120

NC_000963_1_Rickettsia_prowazekii_str__Madrid_E_chromosome_complete_genome probe_cstm_Rprow_021218_1x_105108 CTTGATTAAGTCCTTGTCCTGGCAAGCGGATGTACGATGTGTGCAGTGTCGGCAATAAGCACTATACGATTATGAAAATATCTGTTTGCTATAAAAGCTTTTAGTGGAAAGCTACTAATC 702480 702600

NC_000963_1_Rickettsia_prowazekii_str__Madrid_E_chromosome_complete_genome probe_cstm_Rprow_021218_1x_105109 TCACTCTCAATAGTAATTTGACCTAAGAAATTACCAACATTTCTTTGAATTAAAAAATCAACTTCTTTTGTAGGCAAATTAATAATTAAAGCAGCTTGAACAACAGAAGTGGACCATATT 702600 702720

NC_000963_1_Rickettsia_prowazekii_str__Madrid_E_chromosome_complete_genome probe_cstm_Rprow_021218_1x_105110 ACGGAAGAACTATATTGATCTTTTAAAGGTAGCAAAGCAAAAGGACCAAGAGGAAGAAAATGCTCCATCGCACAATTTTCATGCGGCTTTTCATGCTTAACATTAAAAGTTAGAGCAGTT 702720 702840

NC_000963_1_Rickettsia_prowazekii_str__Madrid_E_chromosome_complete_genome probe_cstm_Rprow_021218_1x_105111 TGATAAGGTTTATCAATTTCATTAGTGAAATAATAAGATCTTACTTTTGAATTTGCTCCGTCACATATAATTAATAAATCACACTTAATTTGTTTATCATTATTAAATTTTATAATCGAA 702840 702960

NC_000963_1_Rickettsia_prowazekii_str__Madrid_E_chromosome_complete_genome probe_cstm_Rprow_021218_1x_105112 TAATCATTATGGCTTATCACTTCTTGATATTGATTATTATCAATTAATGTTATCAACGAATTATTAGTTATTTCTGACAATAATATCTTTTTAAAATCACTATTCTTAACAACATATCCA 702960 703080

NC_000963_1_Rickettsia_prowazekii_str__Madrid_E_chromosome_complete_genome probe_cstm_Rprow_021218_1x_105113 AGTACAGCATCATTCTTATTACATAAATCTAATATTTCTGAAGCCTTATTATCTACAACATATACGTCTTGCATTTCTGCTACAAACTGTTCCAGCTCTTTCCATATCCCAATAGAGAAT 703080 703200

NC_000963_1_Rickettsia_prowazekii_str__Madrid_E_chromosome_complete_genome probe_cstm_Rprow_021218_1x_105114 AAAAACTTTTTAGAATACGGCGTTAACGCAGTAGTTCTTATATCTTTTAAAAAATCTTGGCTTTTTACCAGTTTACGCTCTAATATAGTAGTTTTTATACCTTTGTGTGCAAAAGAAAGT 703200 703320

NC_000963_1_Rickettsia_prowazekii_str__Madrid_E_chromosome_complete_genome probe_cstm_Rprow_021218_1x_105116 aaaataaataatTGCTATATCCAAATATGAGCTATTTCCTGAATACAAACCTAAACAACAAATAAATAAGCAAGTAATAGTTGTACAACTATTTAGAATTAATAAATTCGTAAAAATATC 703440 703560

NC_000963_1_Rickettsia_prowazekii_str__Madrid_E_chromosome_complete_genome probe_cstm_Rprow_021218_1x_105118 TACATAATCACTATTATTACATCAGTGAATCCAGTTAAAAACACTAAAATTATCAGTATTCTTATTATTTTATGTCACAAACACACTAGTTACACAATGACTACTACAGTATCCACATAA 703680 703800

NC_000963_1_Rickettsia_prowazekii_str__Madrid_E_chromosome_complete_genome probe_cstm_Rprow_021218_1x_105120 aaatattgtgacaaaaatgttataaaaaaaTGTTGCTTTCTTTTACATTCAGCATTAACCTATACATTTAGGTTTTTAATATTTTTAAGGTAAATTGTCATGTCACAATTAGATGTTTTA 703920 704040

NC_000963_1_Rickettsia_prowazekii_str__Madrid_E_chromosome_complete_genome probe_cstm_Rprow_021218_1x_105121 ATAGTAGACGATGAAGAAAGTATACGCAATCTCATTGCTGCAAATTTAAAAGATGAAGGTTTTAATCCTAAGATTGCTGCTAATAGTACTCAAGCTCTTAAAATAATTTCCGAGAAACCG 704040 704160

NC_000963_1_Rickettsia_prowazekii_str__Madrid_E_chromosome_complete_genome probe_cstm_Rprow_021218_1x_105122 GTCTCTGCAGTGATACTTGATATTTGGCTTCAAGGTAGCGAAATTGACGGGCTTGGAGTATTAGAGATAATTAAAAAACGTTATCCTTTAATGCCAGTAATCATTATTAGCGGCCACGGT 704160 704280

NC_000963_1_Rickettsia_prowazekii_str__Madrid_E_chromosome_complete_genome probe_cstm_Rprow_021218_1x_105123 ACTATAGAAACAGCAGTTAATGCGATAAAAATGGGTGCTTACGATTATATTGAGAAACCATTTAATAATGATAAATTAATTATTTTACTTAAAAGAGCTTGCGAAGTAACAAAGTTAAAA 704280 704400

NC_000963_1_Rickettsia_prowazekii_str__Madrid_E_chromosome_complete_genome probe_cstm_Rprow_021218_1x_105124 CGCGAAAACATAGATTTAAAATCAAAAGTTATAGATAAAACTGAATTAGTTGGAGGATGTCCGGTAACTTTAAAATATAAAATGGAAATAGAAAAAGCAGCTAGCTCTAGCAGTCGTATA 704400 704520

NC_000963_1_Rickettsia_prowazekii_str__Madrid_E_chromosome_complete_genome probe_cstm_Rprow_021218_1x_105125 ATGATTCATGGTAAAGTCGGTAGCGGCAAAGAACTTGCAGCAAGGTTAATTCATAAACAATCTAAGAGAGTTAATAATCCATTCATTATTTTTAGTCCAACCTGCATGACTACAGAAAAA 704520 704640

NC_000963_1_Rickettsia_prowazekii_str__Madrid_E_chromosome_complete_genome probe_cstm_Rprow_021218_1x_105126 ATTAATCAAGAATTATTTGGAGAATCGGAAAAGCAGGAGAATAATAATAAACGTCCTACTATCTTAGAATTTGCAAATAACGGTACCTTATATATAGATGAGATCAGTAATATTCCTATT 704640 704760

NC_000963_1_Rickettsia_prowazekii_str__Madrid_E_chromosome_complete_genome probe_cstm_Rprow_021218_1x_105127 CCTATCCAAGTAAAATTATTAAAATTCCTTAAAGATCAAACTATTACAAAACCTTGCGGAAAAAAGACTAAAGTTGATATAAAAATTATTACCAGTACTGCTAAAAATATCCAAGATGAA 704760 704880

NC_000963_1_Rickettsia_prowazekii_str__Madrid_E_chromosome_complete_genome probe_cstm_Rprow_021218_1x_105128 GTTAATAACGGAAAATTTCTAGAAGATCTATATTATCGCCTTAATGTTTTTTCTTTAAAAGTACCTTCATTATATGAAAGAAAAGAAGATATACCACTATTAGTTAAATATTTTGTTAAG 704880 705000

NC_000963_1_Rickettsia_prowazekii_str__Madrid_E_chromosome_complete_genome probe_cstm_Rprow_021218_1x_105129 CAACTTTCAAAATTTTCAGGTTTAAAAGAACGTCACTTTTCTGATGAAGCTATTGCAGCTCTTCAATCATACGAATGGCCTGGTAATATTAGACAATTACGTAACGTTGTTGAATGGACT 705000 705120

NC_000963_1_Rickettsia_prowazekii_str__Madrid_E_chromosome_complete_genome probe_cstm_Rprow_021218_1x_105130 TTAATTATGAATCCGTTAACTACAGGTAATAATGAAATTATAAAACCTTATATGATTCCTTCAGAAATATTAGCAAATAGTGCTAATCTTACTAAACTTGAAGATAGCTTTGATATGTTA 705120 705240

NC_000963_1_Rickettsia_prowazekii_str__Madrid_E_chromosome_complete_genome probe_cstm_Rprow_021218_1x_105131 TCTATGCCGCTTAGAGAAGCTAGAGAAGTTTTTGAACGTCAATATCTATCAGCACAAATGAGTCGTTTTAATAATAATATTTCAAAAACTTCTTCATTTGTCGGTATGGAAAGATCGGCT 705240 705360

NC_000963_1_Rickettsia_prowazekii_str__Madrid_E_chromosome_complete_genome probe_cstm_Rprow_021218_1x_105132 TTACATCGAAAACTAAAATTATTAAGCCTACATATACCGCCCACAAATAGAATAAATGAAGAAGAATATGAGAAAGCAAATGCTTAAAATCATATCAATCATTATTATTTCTCTTTTATT 705360 705480

NC_000963_1_Rickettsia_prowazekii_str__Madrid_E_chromosome_complete_genome probe_cstm_Rprow_021218_1x_105133 AAGCAGTTGCTCCGAATCTACGCGTGATGAAAATGGATTACTTACAGATAGTCAAAGTACTATAATTCGAGATTATATAATATCGCAAAATTCTAAAAATCTTAAAGTGAACCTTAAAGA 705480 705600

NC_000963_1_Rickettsia_prowazekii_str__Madrid_E_chromosome_complete_genome probe_cstm_Rprow_021218_1x_105134 AAAGTTTGGTTCCAATTTAAAAGGAGTAAAATTAATAGGAATAAAGTTAACAAATGAAGATTTATCGGGAATAGATTTCACTTCATGCGAAATATTACGGACTGACTTCATGGGTAGCAA 705600 705720

NC_000963_1_Rickettsia_prowazekii_str__Madrid_E_chromosome_complete_genome probe_cstm_Rprow_021218_1x_105135 CTTAGAAAAAGCAATACTTACAAATTCGGTAATTCAAGAAAGTAATTTTGCGGATTCAGTAATAAAAAATATTTCAGGCTATAATGCTGATTTTCAAGGTTCAATTTTTAATAATATAAC 705720 705840

NC_000963_1_Rickettsia_prowazekii_str__Madrid_E_chromosome_complete_genome probe_cstm_Rprow_021218_1x_105136 ATTACAAAATACAAATTTTGTTCAATCAAATTTCAGTGATACTGCTTTTAATAAAAGTACTATAATCAATGTCAATTTTGAAAATTCTAAATTTAGTAATGTATTATGGTGTCACAGTAA 705840 705960

NC_000963_1_Rickettsia_prowazekii_str__Madrid_E_chromosome_complete_genome probe_cstm_Rprow_021218_1x_105137 TATTGACAGTAGTAATTTTCAAAAAACTCATCTAAAAAATAATAGCTTTAAAAATACTAATGTAATGAATTCAATATTTTATGGTGCAGATTTAGGCAAAAGTGTAATAAATAATACAAA 705960 706080

NC_000963_1_Rickettsia_prowazekii_str__Madrid_E_chromosome_complete_genome probe_cstm_Rprow_021218_1x_105138 TTTTACTAATAATTATTTTGAATCTAGTGACCTAAGTAACACTAAATTCACATCAGTAATCATTAAAGATTCTAACTTCACACAAAGTATTTTTAATTCAGTAAATTTCAATAATATACA 706080 706200

NC_000963_1_Rickettsia_prowazekii_str__Madrid_E_chromosome_complete_genome probe_cstm_Rprow_021218_1x_105139 AAGTAATAACTCttttttttCATATACTTCCTTTGAAGATTCAACATTACACAATATTCACCTTACTAAATGTGATTTACAAAACAGCACAATTAATAGTTCAGTTTTCAATAATTTTAA 706200 706320

NC_000963_1_Rickettsia_prowazekii_str__Madrid_E_chromosome_complete_genome probe_cstm_Rprow_021218_1x_105140 AATCGACAATGCTATATTAACAAATATGAGTCTCAACGATAATACATTTAATAATTTATCAATAAAAAATAGTAATACTAATTTTGTAAGGATTAATAAATCCAAAGGGTTTAATATTAC 706320 706440

NC_000963_1_Rickettsia_prowazekii_str__Madrid_E_chromosome_complete_genome probe_cstm_Rprow_021218_1x_105141 TTTACTCAATACTAACTATAGTAATGCTATTTTTAGCAATAATGATTTAAAAGAATTTAAAGTCATTAATACTGATTTAAACAACAGTGAAATAATAAACTCAAATTTCACTAATGGACA 706440 706560

NC_000963_1_Rickettsia_prowazekii_str__Madrid_E_chromosome_complete_genome probe_cstm_Rprow_021218_1x_105142 ATTTAATAATGTAAATTTTTCTCAATCTTTAATACAAAACGTAAATTTTACAGACGTGAAAATTACTTTAGGCAATTTAAATCAAGTAGCTCTAATAAATTCCAATCTAATAAACACTAA 706560 706680

NC_000963_1_Rickettsia_prowazekii_str__Madrid_E_chromosome_complete_genome probe_cstm_Rprow_021218_1x_105143 TATTATTAACTCAGTCCTTTCTAATTCACAAATAAATAATATTAACTACCAAGCATATTATAGTTTTATCAATACTAATGTTTCTAATAATATTGTTATAAATGATAATTCGAATCAAAT 706680 706800

NC_000963_1_Rickettsia_prowazekii_str__Madrid_E_chromosome_complete_genome probe_cstm_Rprow_021218_1x_105144 TCCACCAAATAATATAGTAATCAATTCTGAAAAAGATTTACAAAACATATCTAATTTAGCAAATATGAATTTAACAAATTTTAACTTAAGTAATTTAGTGTTTAATGGAGTAGATTTTTC 706800 706920

NC_000963_1_Rickettsia_prowazekii_str__Madrid_E_chromosome_complete_genome probe_cstm_Rprow_021218_1x_105146 AGGTAGTATATTTAAGTTTGCTCAAATTGATCAGACATGTTTTAGTAATTCCGACTTAACAAATACTGATTTTACTGAAGCAACAATTAAAAATACTGCATTTGATAATGCTAATACACA 707040 707160

NC_000963_1_Rickettsia_prowazekii_str__Madrid_E_chromosome_complete_genome probe_cstm_Rprow_021218_1x_105147 CGGTATAAAAGGATTAGAATAATCTATAATCACGTTTTACGTTTTATGGTTTTGTTGCCACAGCATCAATATCACATATCTTAATAAATGGAATTTAGTTAATTAGTACTACATTTATTA 707160 707280

NC_000963_1_Rickettsia_prowazekii_str__Madrid_E_chromosome_complete_genome probe_cstm_Rprow_021218_1x_105148 GACCACGTAGTCAATATACACACAATAACGTTACACTTATTCAAGCATGACACAAAATTTACCTTTGGTTGCTCTTTGAAGATTTTACTTTATTAGTAGGAGTATGAAACGGTGTAAATG 707280 707400

NC_000963_1_Rickettsia_prowazekii_str__Madrid_E_chromosome_complete_genome probe_cstm_Rprow_021218_1x_105149 AATTCTTATTTAGAAGACTCGAAGGACTTTCATTCTGCATTCTATTCATAATTTGTTGTTGTTTAAGATTATTTATCTTTTGTTTTCCTACTATACTTATAGATTTAGTAATTTTTGCAG 707400 707520

NC_000963_1_Rickettsia_prowazekii_str__Madrid_E_chromosome_complete_genome probe_cstm_Rprow_021218_1x_105152 TTTTCTTTATTCCTTCATAAATTATAGCGAGTCCACTAGCGATCACATCAAATATTTTCGTATCATATTCTTTAGATATAGATGGTGTATCCATAATAATATCTCTAGCTTCAATTAGAT 707760 707880

NC_000963_1_Rickettsia_prowazekii_str__Madrid_E_chromosome_complete_genome probe_cstm_Rprow_021218_1x_105153 TATTAGATAAATGAAGTAATGCTTTTTTATCCTCTAACTCGGTAGCACGATTAGCATATGCACTAACTACATCTGCTTGAGCAGATATAGTATCAGTAATTATTGATTTAGTGTCATTAA 707880 708000

NC_000963_1_Rickettsia_prowazekii_str__Madrid_E_chromosome_complete_genome probe_cstm_Rprow_021218_1x_105154 AAGAAAAATCTATAAATGTTTCTTTTACAGGACTATTCCCTCTTAATTTATCTAACCTATCACGTATTACCTGGTCTTTTTGATCATTGAGGCTTTTACTTTTTTGTTCTAGTTTAAGCT 708000 708120

NC_000963_1_Rickettsia_prowazekii_str__Madrid_E_chromosome_complete_genome probe_cstm_Rprow_021218_1x_105155 CAGATTGCATCTGCTCAATTAAATTGTCTACTTCATTACTAGAtttttttttGTATATATAAGGTGTATCTTGAATCTTCGCCAACCTTTCTTCTAATTCTTCAATTGATGGAGGAGTAC 708120 708240

NC_000963_1_Rickettsia_prowazekii_str__Madrid_E_chromosome_complete_genome probe_cstm_Rprow_021218_1x_105156 CTTTAAGTGTAGCTAGTCTTTCTTCTAACTCTTTGATTATTTGATCATCTTGCTCGTTTGTAGACATATATACCTCTGTCATGATTCTCAATTATTAATAACATAAACACTAACTGAAAT 708240 708360

NC_000963_1_Rickettsia_prowazekii_str__Madrid_E_chromosome_complete_genome probe_cstm_Rprow_021218_1x_105157 AAAGTATTTTTAAATATGAGCTAAAATATAATTAAATCAAGATACATAATAGACATATAAATCTCAACTTTAAAAAGTAAAGTGTTTTAAAACTTTTATTTAACTGCAGAATACATAACT 708360 708480

NC_000963_1_Rickettsia_prowazekii_str__Madrid_E_chromosome_complete_genome probe_cstm_Rprow_021218_1x_105158 AAGCAGCACGTACATCAACATCACTAAATTCTATGATAATTAAAAGTCAAAAAATAACGACACTATTTTTATTGCATATACTTATCAAGAACATCAGACATTATTTTACGAGCTATGGGT 708480 708600

NC_000963_1_Rickettsia_prowazekii_str__Madrid_E_chromosome_complete_genome probe_cstm_Rprow_021218_1x_105159 GCAGCTGCAATACTACCGCCTCCTCCATGATCAACAAAAACGGTAACAGAATAACGAGGATCTGGATACGGAGCGAATCCTAAGAATAAAGCATGATTACGTCTTTCCCATGCAATAGAA 708600 708720

NC_000963_1_Rickettsia_prowazekii_str__Madrid_E_chromosome_complete_genome probe_cstm_Rprow_021218_1x_105160 TCACGGTTTAAATCATCTTTAGCATTTAGttttttttGCACCTGAGCAGTACCTGTTTTACCAGCAAGCTTCCTATTTTTAGCAAAAATTCTATTATAATACGCCGTACCTCCAGCAACA 708720 708840

NC_000963_1_Rickettsia_prowazekii_str__Madrid_E_chromosome_complete_genome probe_cstm_Rprow_021218_1x_105161 TTCACAGTATTATACAAACTTTCTTGTATTATTTTGATATTTTCAGTCTTAATATTCACATTATAAAACTCTGGATCATTTTTTAATATTCTAGGCGTATATAGCTTGCCATTACTTGCA 708840 708960

NC_000963_1_Rickettsia_prowazekii_str__Madrid_E_chromosome_complete_genome probe_cstm_Rprow_021218_1x_105162 ATAGCCGTAATAAATCTTGCCAACTGAATTGGCGTTACTCCTAAAAACCCTTGCCCAATTGCTAAATTAAAACTATCACCTATTGACCAAGGGAGTTTGAAttttttctttttCCATTCT 708960 709080

NC_000963_1_Rickettsia_prowazekii_str__Madrid_E_chromosome_complete_genome probe_cstm_Rprow_021218_1x_105163 TTTGAAGGTATAAAACCACTACTTTCAGGTGATACATCAATACCAGTTTTTGAACCAAAGCCAAATTCTCTTGCAACTTCAAGAATTTTATCCGGTCCTACTATTTTAGCAAGTTCATAC 709080 709200

NC_000963_1_Rickettsia_prowazekii_str__Madrid_E_chromosome_complete_genome probe_cstm_Rprow_021218_1x_105164 ATATAAATATTACAGGAATACTTTAAAGCAGACATCATATCTAGCGTACCGTGCCCTCGATGATTCCAACATCGGAAACTATTAGTACCAAGAATAGAACTACCATCACAAAAAACTGTT 709200 709320

NC_000963_1_Rickettsia_prowazekii_str__Madrid_E_chromosome_complete_genome probe_cstm_Rprow_021218_1x_105165 TTATTAGGATTAATCCCTACTTCAAGTGCTGCAAGTACGGTAATTATTTTAAACACTGAACCAGGAGGATAAGAATTTTGTATTACCTTATTAACTAACGGCTTATAAGGATCACTCATT 709320 709440

NC_000963_1_Rickettsia_prowazekii_str__Madrid_E_chromosome_complete_genome probe_cstm_Rprow_021218_1x_105166 AAACTTTGCCAATAATTTTCTGATAATTTACTGAAATTATTGGATTCAAAACCCGGGGTAGAAGCACAAATTAGCACATTTCCAGTTCTAATATCCATAACTATTGCAGAAGAACCTTTA 709440 709560

NC_000963_1_Rickettsia_prowazekii_str__Madrid_E_chromosome_complete_genome probe_cstm_Rprow_021218_1x_105167 GGATTTAAGTATTGCTGGATATGTTGCTGAAGAGAAGCATCAATATTTAAATGCATATCCTCACCTGATTGAGTAGGTGTCTCTGATATTGTCCTTACCTGCTTACCATAAGCGTTAACT 709560 709680

NC_000963_1_Rickettsia_prowazekii_str__Madrid_E_chromosome_complete_genome probe_cstm_Rprow_021218_1x_105168 TCAACTTTTTTATAACCAAATTCTCCTCGTAATTTATTATCATAATATTTTTCAATACCGGATTTACCTATATTAAAATCACTTAAACTACGTATATTTAACTGTTGCTTATCTTGCTCA 709680 709800

NC_000963_1_Rickettsia_prowazekii_str__Madrid_E_chromosome_complete_genome probe_cstm_Rprow_021218_1x_105169 TTGATCTGACCAAGATAACCTATTAAATGAGAAGTAGTGCTTGAAAAAGGATAAAATCTTAAATACCCTACATCAATAAATATCGCAGCTAGTTTATGCTTTTGCTCTTCAATCACCGAA 709800 709920

NC_000963_1_Rickettsia_prowazekii_str__Madrid_E_chromosome_complete_genome probe_cstm_Rprow_021218_1x_105170 ACTTGTTTCCAATCAAGATGATCAAGTATTGTTAAAATTGTGTGACGACTAGAtttttttattttttGCTTAATATAATTATATTTATCTGGAGAGAAATTTAAAATGTTACTAATTATT 709920 710040

NC_000963_1_Rickettsia_prowazekii_str__Madrid_E_chromosome_complete_genome probe_cstm_Rprow_021218_1x_105171 TCCAATTCATCTCTATAATTATTATTAATACTCTTATCTATTACTAACTGATAACATGGTTTATTAGTAGCTAAGATATTACCATCTAAATCATAAATTTTTCCTCTAATCGGCGGAAGT 710040 710160

NC_000963_1_Rickettsia_prowazekii_str__Madrid_E_chromosome_complete_genome probe_cstm_Rprow_021218_1x_105172 ACAACAAAATTAATACGATTTTTATCTGATAAAGTCCTATATTCTTCACTTTTAATAAGCTGTAAATAAAACATTCTTATACCAAGCAGTGATAAAAACCAAAGTTTACCTAATCCAATG 710160 710280

NC_000963_1_Rickettsia_prowazekii_str__Madrid_E_chromosome_complete_genome probe_cstm_Rprow_021218_1x_105173 ATAAAGGCTCTTCGTGAAATCAACTCACCATGTAGTAtttttttATTTAGCATACTTTTTAAAATAATCCATAGGAGAGTCAAGTATAAGACGAATTATATTATAAGAAAATATAGTTGT 710280 710400

NC_000963_1_Rickettsia_prowazekii_str__Madrid_E_chromosome_complete_genome probe_cstm_Rprow_021218_1x_105178 GTGTTTCCTTCTTTACGTGAAAATTACTTGCTAATGAACTAAGCAATATCAAGACAtttttttAGCAAAGTTTCACCTCTCGGAATAATTGCTCTAGTATCCATTACATTACAAAATATA 710880 711000

NC_000963_1_Rickettsia_prowazekii_str__Madrid_E_chromosome_complete_genome probe_cstm_Rprow_021218_1x_105179 AATTAATAATTTTCTTTTTGTACAATTTTATTCTTGTATCATCATCAGCACTAATGCTTCTTGTTCTTTGGAATTTGTAGAGCTAAACAGTTTTTCAAATTATTTCTTTAGTATAACAAA 711000 711120

NC_000963_1_Rickettsia_prowazekii_str__Madrid_E_chromosome_complete_genome probe_cstm_Rprow_021218_1x_105180 AAGACTTAAGCTGTAAATATTAGGTTTATTTATTATATTTTTCTTACTCAATGTCATTCCTATACAATTCGGAATCCAACATAAAATGCGATATAAATATACTGCTAAAGCAAGAATATA 711120 711240

NC_000963_1_Rickettsia_prowazekii_str__Madrid_E_chromosome_complete_genome probe_cstm_Rprow_021218_1x_105181 AACGAGCTATAATACAAATGTTTTAAATTAAATGAGTATATCAACTCTCTTCCTCATTTTGTTCTATCGGTTCTAAACCATATAAAGATATCATACGTTCAAAAACTCTACCAGCAGTAG 711240 711360

NC_000963_1_Rickettsia_prowazekii_str__Madrid_E_chromosome_complete_genome probe_cstm_Rprow_021218_1x_105182 GTACAGCAGTCCAGCTTGCCGTTGCAAAGCCAAAGCTTTCTTTAATTGGTTTTGGCTCATCAAATCGAATAAAAAGAACATATTGCGGCTTAGATGAAGGCAGTATACCTAAAAATGATG 711360 711480

NC_000963_1_Rickettsia_prowazekii_str__Madrid_E_chromosome_complete_genome probe_cstm_Rprow_021218_1x_105183 AAGCTCTACTATTTTTAAGATATTTCTTTTTTCCTCCTATACCTTGGGAAAGTTTTTCTGCCGTACCGGTCTTACCGCCGATAAGATATCCTTTAATCTCTGCTCGTTTACCGTTACCTT 711480 711600

NC_000963_1_Rickettsia_prowazekii_str__Madrid_E_chromosome_complete_genome probe_cstm_Rprow_021218_1x_105184 CTTTCACTACTGACCTAAACAATTTATTCATTTGGGTAGAGGTATTTTCACTAAAAACTTTTGTACCGATTACTTTTTTAGTCTTTCTTTTTAATAAAGTAAGATCATATAAAGTACCAC 711600 711720

NC_000963_1_Rickettsia_prowazekii_str__Madrid_E_chromosome_complete_genome probe_cstm_Rprow_021218_1x_105185 CATTAACAACTGGTAAAATTGCTTTAACAAAATGCAAAGGGCTAATTGAAATACCATAGCCGTACGACATAGTAACACTAGTTAGTTCATTCCATCTTTTTTCTGAAGGAAATAATGGTG 711720 711840

NC_000963_1_Rickettsia_prowazekii_str__Madrid_E_chromosome_complete_genome probe_cstm_Rprow_021218_1x_105186 TACCTCGTTCAGGCAATTCTATTTGTAATTGATCTAATAACCCTAATTTCTTTAGGTATTGTTTAAAATCATTTTTACCGATTTCAAGCATGATTTGACTTGTACCAATATTTGAAGAAT 711840 711960

NC_000963_1_Rickettsia_prowazekii_str__Madrid_E_chromosome_complete_genome probe_cstm_Rprow_021218_1x_105187 ATaaaaaaaTCTCAGGTACACTATGCCATCCTTGCCGTGGAGTATAATCCTTAAGCTGGAATCCGCCTACTTTCATATAGCTTATATCATAAGCATCATTCATCTTGATTACACCGGTAT 711960 712080

NC_000963_1_Rickettsia_prowazekii_str__Madrid_E_chromosome_complete_genome probe_cstm_Rprow_021218_1x_105188 CAAAACCAACAGCCATAGTTAATGCTTTAAACACAGAACCCATTTCATAAATGCCAAGACTTGCTGTATTAAACAGCTCCTCCGGTTTTGCTAAACTTGGATAATGAGGATCAAAATCAG 712080 712200

NC_000963_1_Rickettsia_prowazekii_str__Madrid_E_chromosome_complete_genome probe_cstm_Rprow_021218_1x_105189 GCTTATTTACTAATGCCAAAATCTCACCGTTATTAGGATCCGCAATAATTCCTACTGCTCCTATTGCCTTAAATTTCTTTAAAGTTTTATCAATCTCCTCATTTAAAATACTTTGTAATC 712200 712320

NC_000963_1_Rickettsia_prowazekii_str__Madrid_E_chromosome_complete_genome probe_cstm_Rprow_021218_1x_105190 TGATATCAATAGATAATTGAAGTGGTGCTTTCTGATCCTTTGGATCATTTAATTCATAATCAGTATTAGTTAAATATTTATCATATGCAAGTTCTAAGCCACTCAATCCTACTGAATCTC 712320 712440

NC_000963_1_Rickettsia_prowazekii_str__Madrid_E_chromosome_complete_genome probe_cstm_Rprow_021218_1x_105191 TACCTACATAACCAATAATATGTGACAATAAATTTGAAAAGATATAAATTCGTTTCTGCTCTTCTTCAAATTCAAAACCAAGTAACCCAAGGCTCATTATCTTTTCTTGTTGACTAGGTA 712440 712560

NC_000963_1_Rickettsia_prowazekii_str__Madrid_E_chromosome_complete_genome probe_cstm_Rprow_021218_1x_105192 ATAAATCTCTTTTGACCCATATAAAGCTTTTATTTGACTTAAGTTCTTTAATTAATTTAGCTTTATTAATATCAGGTAAAATTTCTGCAAGCTTGTTTACAGAAGTTTCAGGATCAAGTA 712560 712680

NC_000963_1_Rickettsia_prowazekii_str__Madrid_E_chromosome_complete_genome probe_cstm_Rprow_021218_1x_105193 CTATTTGTGGATTAGCAAATAATGAGGCAGATGGTAGATTCATGGCTAATAAATTACCATTTCTATCAACTATTTCTTTTCTAAATTGATGtttttttttGAAGGAATTAATATTTTGAT 712680 712800

NC_000963_1_Rickettsia_prowazekii_str__Madrid_E_chromosome_complete_genome probe_cstm_Rprow_021218_1x_105194 CATAAACATTTGTAGCAACAATTATCAATTTATAAGATACAGTAGCaaaaagaaaagaaaaaCCACAAATAACCATTAATAATCGAATTTTTGTATTTTTACTACAAATATTCCAAATGA 712800 712920

NC_000963_1_Rickettsia_prowazekii_str__Madrid_E_chromosome_complete_genome probe_cstm_Rprow_021218_1x_105195 TTAGACTTTTAATTGCAGGCTTCCATTTTTCTAAAATTTGCTTCATTTTATTTATAGTGTCTTAAGTAGCAGTCATGTCATTGTAAGTGCAAACTATAAGTTTTGAATCAACAATCTTAT 712920 713040

NC_000963_1_Rickettsia_prowazekii_str__Madrid_E_chromosome_complete_genome probe_cstm_Rprow_021218_1x_105196 AAAAAATTCCTGAGACGACAATATAGAATTATTGCTAACTAGCTTATGACAATTATTTAACTCTACTTGAAACAGTCTGTATATACTTATTATTCATAATTCTTTTGTAACGCCATTTAT 713040 713160

NC_000963_1_Rickettsia_prowazekii_str__Madrid_E_chromosome_complete_genome probe_cstm_Rprow_021218_1x_105197 TACTCTTTGAAATACTAATATTATGATTAAATTTAATATTTTGTTCGATATTTGGTGCTAACGGATCATTTATCATTTGATATGATTTCACTATTTCTAATTTTAAATAAGCTGCAGCGA 713160 713280

NC_000963_1_Rickettsia_prowazekii_str__Madrid_E_chromosome_complete_genome probe_cstm_Rprow_021218_1x_105198 GTTTTTCCAACCTAGTTGGCAAAAGTAGATAAGCTTGCTCTGCTTTCAAAATATTAATATTGTTATTCTCACTATTGATCTGTTTTATCACACTCCTTAATTGATAATTCAGAGTTGAGA 713280 713400

NC_000963_1_Rickettsia_prowazekii_str__Madrid_E_chromosome_complete_genome probe_cstm_Rprow_021218_1x_105199 CTCGATCCTTTATCCGGAATAAACTACATATTGCTATAATTGTTATAAATAATATTAAATAGTGAAACTTTCTTATAGTCATATAGTCATAACTAAATTACATATAATACTTCTTAGACA 713400 713520

NC_009488_1_Orientia_tsutsugamushi_Boryong_complete_genome probe_cstm_Rprow_021218_1x_1052 TTAACTGAAAAAATGAATTCCCTTTATTAAAATATTTGTCTGCTAGCATATCTTTACCCTAAATTGAATACTGTTATATGAGCAGATTATTTTGATGCAATAGATGTAACTGTATTAATA 126120 126240

NC_000963_1_Rickettsia_prowazekii_str__Madrid_E_chromosome_complete_genome probe_cstm_Rprow_021218_1x_105200 ATTTTTTAGAACATGTGATATCATTCAATCAAAGTGTTACCTGACTACTAGATACCCAGCACGTCATTGCTATGAAACGGCGCTACGAAGCGATCTCATGCAGTAATAATAAATTTTTCG 713520 713640

NC_000963_1_Rickettsia_prowazekii_str__Madrid_E_chromosome_complete_genome probe_cstm_Rprow_021218_1x_105201 ATAATGTCTTAATTACCTCATAATTTTCTTATTAATGATAAGAAAAATAGTAAATTCAACCATGACTTATATTATCTTTTTTGCGGCTCGAAGTTTTGCTGATCTAGCTCTAACATTAAG 713640 713760

NC_000963_1_Rickettsia_prowazekii_str__Madrid_E_chromosome_complete_genome probe_cstm_Rprow_021218_1x_105202 CCTTATCTCTTGACTTGACGGTGTTAGAACTTTGGGCGTAATAATCTTAAGCCATTTATTTGGATCAATTTTTATTTCATCTTTAGCATATTTAGATCTTGCTACCTGCTTTTCTGAATT 713760 713880

NC_000963_1_Rickettsia_prowazekii_str__Madrid_E_chromosome_complete_genome probe_cstm_Rprow_021218_1x_105203 TTCTTTaaaaaaaTTCTTAACTATTCTGTCTTCTAAAGAGTGAAAAGATACCACAACTAAACGTCCATCTTTCTTTAAGATATTTTTTACATTCGCCAAAAACTGCTCTAATTCCTCAAG 713880 714000

NC_000963_1_Rickettsia_prowazekii_str__Madrid_E_chromosome_complete_genome probe_cstm_Rprow_021218_1x_105204 CTCATTGTTTATATAAATCCTAATAGCTTGAAAAGTTTTAGTTGCAGAATCAATTTTTCCTTTTCTAAATCCTATACTATACCTTACAATTTCAGCTAGCTTGCGAGTACTATCAATTCT 714000 714120

NC_000963_1_Rickettsia_prowazekii_str__Madrid_E_chromosome_complete_genome probe_cstm_Rprow_021218_1x_105205 TGCAGTTTTTCTATATTCGACAATACTCTTAGCTATTCTTCGTGACAAACTCTCATTACCATATTTATAAATTACATCTGCAATCTCTTCTTCATCTGCAGTATTTACAAATTCCTCAGC 714120 714240

NC_000963_1_Rickettsia_prowazekii_str__Madrid_E_chromosome_complete_genome probe_cstm_Rprow_021218_1x_105206 ACTAAACCCTTGCACACTCATACGCATATCTAATGGTCCATCATACAAAAAAGAAAATCCTCTATCTGCGATATCTAACTGCATTGATGACACACCTAAATCCATCACTATCCCATCAAA 714240 714360

NC_000963_1_Rickettsia_prowazekii_str__Madrid_E_chromosome_complete_genome probe_cstm_Rprow_021218_1x_105207 TTTTTTGTGCTTAAGTTTTCTAAAGCTATCAGCAAAATTTGTTTTTATAAAATTAAATCTCTCACCGTAATCTTGCTTAATTTTTTCAACGCTTTCAATTACGTTTGGATCACGATCCAG 714360 714480

NC_000963_1_Rickettsia_prowazekii_str__Madrid_E_chromosome_complete_genome probe_cstm_Rprow_021218_1x_105208 AGACGTGACAGAACAGTAACAACTATTCAATATTGCGTTACTATATCCTCCTGCACCGAACGTGCAATCTAAATAAGATTCATAGCCTTTGGGCGCTAAAACCGCTAATACTTCACTTAA 714480 714600

NC_000963_1_Rickettsia_prowazekii_str__Madrid_E_chromosome_complete_genome probe_cstm_Rprow_021218_1x_105209 CATTACAGGTATGTGGTACTGTGACATTAATGCGCATTTCTTAGAGTTAAACGCTTTTCATGAGCGATATTTTGAGCGTAACTTAAATATTTTTCAAAATTCTTAGGTTGCCAAATCTCA 714600 714720

NC_000963_1_Rickettsia_prowazekii_str__Madrid_E_chromosome_complete_genome probe_cstm_Rprow_021218_1x_105210 AAAATTATCCCCTTACCTACAAAACATGCTTGCTCTTCTATACCTGCATGTTTCATTAAAGACTGTGGCAATATAATTCTTCCATCACCATCGAAGGCAAGCTGTACAGCTTCCCCAAAT 714720 714840

NC_000963_1_Rickettsia_prowazekii_str__Madrid_E_chromosome_complete_genome probe_cstm_Rprow_021218_1x_105211 ATCATAGTCTCGAAAGCATCACGTTCCTCAGAATACGGATCAAGCGTCTCAATCATCTTCCTTAATTTCTCAATATGCGCGATACCACACACTTCTATACAATTATTCCTAATTGACGGA 714840 714960

NC_000963_1_Rickettsia_prowazekii_str__Madrid_E_chromosome_complete_genome probe_cstm_Rprow_021218_1x_105212 TAAGCAATAACACCGTTAAATAATTCTTTCCCTAGTACTGCACGGTAATTTGCCGGCACTGTTACCCTACTCTTTTTATCAACACCATTGATATACTTAGATAGAAATACATTCATTGGT 714960 715080

NC_000963_1_Rickettsia_prowazekii_str__Madrid_E_chromosome_complete_genome probe_cstm_Rprow_021218_1x_105213 ATAATTCGGGTTACCTTGGGATTTAATGGTAATTTATAGGATATGATAGGACTATAACGTAGTCAAGCGTTTTTAACAGCTAAGACTTCAATTATTAACTTGTGTCTTTATTATCATTAA 715080 715200

NC_000963_1_Rickettsia_prowazekii_str__Madrid_E_chromosome_complete_genome probe_cstm_Rprow_021218_1x_105214 GTACTTGCATTATTAATGTTTTCAGTCATTTCGAGAAAATTGCATTACAATTACAAAACGATCTCATACAAAAATCCTTTGTACTACTTTTAGTCATACGAAAGACTCAAATCTTAATAA 715200 715320

NC_000963_1_Rickettsia_prowazekii_str__Madrid_E_chromosome_complete_genome probe_cstm_Rprow_021218_1x_105215 ATGATTAAATTAAATGAAACAAATCAAGTAAATCAGTTAAGAATTCCAATACTTAATAAAATTCATAGTACCAAAATCATTGATAAATTAGATAAATCTATTGTTAACCTTGTAGGATAT 715320 715440

NC_000963_1_Rickettsia_prowazekii_str__Madrid_E_chromosome_complete_genome probe_cstm_Rprow_021218_1x_105216 AATCCTCAAAAACAAATGCCTAAACTATGAAATTGAAAGATAAAATCGTTATGTTTTTTGATAAGTGATAAGATAATTGTATGGATTTTTAAAGTAATGTTCTCAAATTCAAAATATACA 715440 715560

NC_000963_1_Rickettsia_prowazekii_str__Madrid_E_chromosome_complete_genome probe_cstm_Rprow_021218_1x_105217 TATTTTTATTGCAGTGGTTAAATAATTATAGCACTTTGAAAAATAGCGAAAATTTATGAAATCTAGAAGATAAAAATAATAAGTTACATTGCCGTAATATCACAAATACACCTATGATAG 715560 715680

NC_000963_1_Rickettsia_prowazekii_str__Madrid_E_chromosome_complete_genome probe_cstm_Rprow_021218_1x_105219 aatGTAATATATGTTGCAATCTAAAATAGGTATTTTAAAACTATCCCTTAATCACACACTAACTATTCATTaaagaaaaaactactaaaaaataaaaTATTTAATATGATCTAAAACTAC 715800 715920

NC_000963_1_Rickettsia_prowazekii_str__Madrid_E_chromosome_complete_genome probe_cstm_Rprow_021218_1x_105220 TAGAACATATTATTACACATAATAATTACTAAAAATATATTATGAACAAATTCTAAGTTTTATCTTTAAAATCTAATTTATGTTTAATTTACctatttataatataattgtattcatata 715920 716040

NC_000963_1_Rickettsia_prowazekii_str__Madrid_E_chromosome_complete_genome probe_cstm_Rprow_021218_1x_105221 tctaatatctattctagttattggtatatattACCGAGCTAAACATAGTAGTTTTAAGCACTATGCAAATGTTGAGCGCAAATCACAACATAATAAACTATTACTAATAGCTACTATATT 716040 716160

NC_000963_1_Rickettsia_prowazekii_str__Madrid_E_chromosome_complete_genome probe_cstm_Rprow_021218_1x_105222 TACAAGTTCTGTCGGAGGGGCTACCACCTTCGGCATTATGGAAAAAGTATTTTTAGGACATGGATATTATGCTTACGCCCTAATAGTTACAATACCTATAGATATATTAATTGCATTCTA 716160 716280

NC_000963_1_Rickettsia_prowazekii_str__Madrid_E_chromosome_complete_genome probe_cstm_Rprow_021218_1x_105223 TATGGTGCCTTTAATTGCAAAACATTACGGGGCCGAAAGTATTGGTGACATAATGAGTAGATATTATGGTAATACAGGTCGTTTTATCTGTGGTATTAGCACGGTTATCGTTTCAGTAGG 716280 716400

NC_000963_1_Rickettsia_prowazekii_str__Madrid_E_chromosome_complete_genome probe_cstm_Rprow_021218_1x_105224 TTTTTTAGCCGCTCAGATAAGCGTAAGCGGCTATATTTTCAAATATATTTTAGGAATACATTATGTAAATGGGGTGGTTTTAAGCTATAGTATAGTACTTATATATACTACAATAGGTGG 716400 716520

NC_000963_1_Rickettsia_prowazekii_str__Madrid_E_chromosome_complete_genome probe_cstm_Rprow_021218_1x_105225 TTTACAATCAATTATTTTTACTAATTTATTACAATTTTTTGCAATGATAATAGCTATACCTACTATAACTTTTATAAGTTTAAATAAAATCGGTGTTGTATATTTTATAGATAATTTTAC 716520 716640

NC_000963_1_Rickettsia_prowazekii_str__Madrid_E_chromosome_complete_genome probe_cstm_Rprow_021218_1x_105226 AAACACAAATTTTGATCAAAATAATCGACTATATTACACTATCACAGCAGCTTTAAACTTTAGCGTGATGAATCTATATCCTACATTTATACAAAGAGCGTTAATTAATAAAAATCCTAC 716640 716760

NC_000963_1_Rickettsia_prowazekii_str__Madrid_E_chromosome_complete_genome probe_cstm_Rprow_021218_1x_105227 ACATACTACTAAAGCAATATATGTAAAATCTGCTATATATTTTTTCTTCTTGATTTGCATTACTTTAAACGGCTTAATTGCATTTAAGCTTTACCCAAATCAACCATCAAATTTAGTATT 716760 716880

NC_000963_1_Rickettsia_prowazekii_str__Madrid_E_chromosome_complete_genome probe_cstm_Rprow_021218_1x_105228 ACCATATTTAATCAACCAAATCATCCCACCTGTAATGCAAGGATTTGTTATAAGTGGACTACTAGCTGCAGTTATGTCTACTGCAGATTCTGATTTAAATGTTACTTCTATATCtattgt 716880 717000

NC_000963_1_Rickettsia_prowazekii_str__Madrid_E_chromosome_complete_genome probe_cstm_Rprow_021218_1x_105230 TGTAATCGATTTAGTAATGTTCTTTACAGGTTTCTGGGGCCCTGTAATATTAGTACCGTTAATAACAACACTTTTTGATATCAGAACATCTAAAATTATAATTGTCTTATCATCATTTAG 717120 717240

NC_000963_1_Rickettsia_prowazekii_str__Madrid_E_chromosome_complete_genome probe_cstm_Rprow_021218_1x_105231 TGGAGCCACAACTTTTTTAACGTGGGAATATTATTCTTTCTCATTGCAATATTTCAACCTTAGAGGAGTGTTTGTAGGCACCATCATGAGTCTTGTGATATTTATTTTAGGACAAATAAT 717240 717360

NC_000963_1_Rickettsia_prowazekii_str__Madrid_E_chromosome_complete_genome probe_cstm_Rprow_021218_1x_105232 TATTTCTAGAAAATTACTCAAGTAATGATCCGTAAGAAAATTTATTTGCTGAGATCACAAAACTAATTATGAGCACATTCTTGTTTCTTGTTGCATTGCTCATTTATATCATTCTTGTAA 717360 717480

NC_000963_1_Rickettsia_prowazekii_str__Madrid_E_chromosome_complete_genome probe_cstm_Rprow_021218_1x_105233 TAACAGAAATCTATTCACAACTAATATTATTCTACAAACTTAATCAAGTTTAAAATACTTGAATTATTAGTAtttttttATCGCTAAATCTTAAGATATGTATGTGTCTATTTCAGTTAA 717480 717600

NC_000963_1_Rickettsia_prowazekii_str__Madrid_E_chromosome_complete_genome probe_cstm_Rprow_021218_1x_105234 GCAATCTTTAATGATTCCAATGCCATTTCTCATGAATTATCAAGTGAGATTTTGCGATTTAAAATATTAAAAATCATACTAGCAAGTGATTTACTAATGCCTTTTACTTTAGTAAGCTCA 717600 717720

NC_000963_1_Rickettsia_prowazekii_str__Madrid_E_chromosome_complete_genome probe_cstm_Rprow_021218_1x_105235 TCTATGGTTGCATTACAGACTGCTTTATATGAACCAAAATAATGTAGCAATGCTGTTTTACGAGTTTCACCTATACCATCTATCTCATCAAGGCTTGATATTTTTATAGCACGTGATCTA 717720 717840

NC_000963_1_Rickettsia_prowazekii_str__Madrid_E_chromosome_complete_genome probe_cstm_Rprow_021218_1x_105236 CCTAGTCTATGATTCTTTATGGCAAAATTATGGGCTTCATCTCGTAGGATTTGTAAATATTTCATAATTGGCAAATTTTTATCTAAAGTCAAGACTTCTTTACCTTGCATATGTAATTGT 717840 717960

NC_000963_1_Rickettsia_prowazekii_str__Madrid_E_chromosome_complete_genome probe_cstm_Rprow_021218_1x_105237 TCAAGACCAGCATTTCTATCTCTGCCTTTCGACATGCACACAAAAGGGATATTTATTTCAAATTTATCCATTACCTCTTTAACAATAGTTAAATGTCCTTTACCACCATCTATAATCATT 717960 718080

NC_000963_1_Rickettsia_prowazekii_str__Madrid_E_chromosome_complete_genome probe_cstm_Rprow_021218_1x_105238 AAGCTTGGCAATTTATGCGGCTCATTTTTAAGTCTAGTGAATCGTCGTGTTAAAACTTGCCGCAGCATCCCATAATCATCACCTTTTCTGTCATCTCGCAGTTCAATCTCTGAACCTATT 718080 718200

NC_000963_1_Rickettsia_prowazekii_str__Madrid_E_chromosome_complete_genome probe_cstm_Rprow_021218_1x_105239 GTCAAACTACGGGATGATAATGAGAAAACCCTATATTCTTTTTTATCAAAACCGATTTGACCAGCAACTACCATCACTCCCACTGCAAACATACCTTGAAGATGACTATTATCATAAATC 718200 718320

NC_000963_1_Rickettsia_prowazekii_str__Madrid_E_chromosome_complete_genome probe_cstm_Rprow_021218_1x_105240 TCAATTCTTTCCGGAATTTCAGGAAGATCAAATAGCTTTTTAAGTGCAAACATAATATCTGTATTCTTAATACTTTTCTTTAAATACTGTTCTAGAGATAACAAAGCATTGACCTCGGCC 718320 718440

NC_000963_1_Rickettsia_prowazekii_str__Madrid_E_chromosome_complete_genome probe_cstm_Rprow_021218_1x_105241 TTTTGGACTAATTTAGCTTTACCACCTTTATGAGGTACTGTAATACTAAGATCAGATATATTATTGAtttttttAATAGCTTCTATCATATTCTCTTTAGAATTAATTTCATGATTCATA 718440 718560

NC_000963_1_Rickettsia_prowazekii_str__Madrid_E_chromosome_complete_genome probe_cstm_Rprow_021218_1x_105242 ATAATTTCTGAAGGTACTTGTTGTTTTTGATAAAATTGTAATAAAAAATATTCTAATACTTCTTCGTGGGTACTATTTTCAGTAGAAGaaaaaaaaTAAGGAATAGCACCATATGGTTGC 718560 718680

NC_000963_1_Rickettsia_prowazekii_str__Madrid_E_chromosome_complete_genome probe_cstm_Rprow_021218_1x_105243 CCAGCCCTATATAAAGATACCTCAATACAATAATGCAAATTCTTATGCACAATAGCGATTATATCTGCATCTCTAACAACATCTAAAATTCCAGACTTAAGCTGAACATAACTAAGCGCC 718680 718800

NC_000963_1_Rickettsia_prowazekii_str__Madrid_E_chromosome_complete_genome probe_cstm_Rprow_021218_1x_105244 TTAATACGATCTCTGATTTCTGCTGCCTCTTCAAAACGCATCTGACTACTTAACTCTTGCATCTTTTTAGATAGATTTGCTTGTAGTGCTATAGTACGGCATTGTAGAAAATCCTTTACC 718800 718920

NC_000963_1_Rickettsia_prowazekii_str__Madrid_E_chromosome_complete_genome probe_cstm_Rprow_021218_1x_105245 TGCATTACTAGTTCTCTATAATCTTCCTTATTTATTTTACCGACACAAGGAGCATAACAACGTTTTATTTCATATTGCAAGCAAGGACGAGTACGAGAATTAAAATAATTATCTGTACAG 718920 719040

NC_000963_1_Rickettsia_prowazekii_str__Madrid_E_chromosome_complete_genome probe_cstm_Rprow_021218_1x_105246 GAACGTAATTTAAAAATTTTTTGCAATTCCGTTAAAGTAGTATTAACTTGTGTATTTGATGCAAAAGGACCAAAAAACTTTCCGTCACTTAGAGCTTTACCTCGATATTTTATTAATTGT 719040 719160

NC_000963_1_Rickettsia_prowazekii_str__Madrid_E_chromosome_complete_genome probe_cstm_Rprow_021218_1x_105247 GGGAAATCATGATCTAAACTTAACTTAATGAAAGGGAAAGATTTATCATCTTTAAGGAGAATATTAAATTTCGGCTGAAATTTCTTAATGAGCTGAGCTTCTAATAGTAATGCCTCAACT 719160 719280

NC_000963_1_Rickettsia_prowazekii_str__Madrid_E_chromosome_complete_genome probe_cstm_Rprow_021218_1x_105248 TCAGAATTAGTGATAATGTACTCTAAAAAATAAGTATTTGAAATCATCCGTAGAGTCTTATTATCTAAATCGCTTTTAATATAATTAGTAAGACGtttttttAAAATTTTAGCTTTACCG 719280 719400

NC_000963_1_Rickettsia_prowazekii_str__Madrid_E_chromosome_complete_genome probe_cstm_Rprow_021218_1x_105249 ATATAAATAACCTGCTTATTAACATCTAGCATTTTATAAACTCCTGAACATTCAGGAGCATCTATAATTTGCGCTTTAATTAACTTACTTCCGCTAATTTCTAAACTCATCTTTTATTTA 719400 719520

NC_000963_1_Rickettsia_prowazekii_str__Madrid_E_chromosome_complete_genome probe_cstm_Rprow_021218_1x_105250 TCTATCATTGCAAAGGACAATTGTCAGCTTGGGCGGAATACCATAAAGATGGTGGGGAAGATTAAAACAATGTACAATACATCACAATAAAACTTTAACTAATTCCCTATTTTATTATAA 719520 719640

NC_000963_1_Rickettsia_prowazekii_str__Madrid_E_chromosome_complete_genome probe_cstm_Rprow_021218_1x_105251 GGATTATCATTTGCTACTTTGACATTCACTATTTTTTGTGTATTGTCATCAAAGAAAAAGGTTATTTGGAACTCATCGCCTGCATTTAAATCTACTTTTGGGTCATAAAGCATGATATGC 719640 719760

NC_000963_1_Rickettsia_prowazekii_str__Madrid_E_chromosome_complete_genome probe_cstm_Rprow_021218_1x_105252 CTGCTACCAGGCTTAAAATCAACATTAATATTACCAGAGATTAAAAATGGATAATCTACTTTTACCATTTTATTAACCCCTTGATCATTAATTGTTTGATGGATTTCTATCCCACCTATC 719760 719880

NC_000963_1_Rickettsia_prowazekii_str__Madrid_E_chromosome_complete_genome probe_cstm_Rprow_021218_1x_105253 TTATCTGAAGATATATTTACTAACTGATAACTTTTATTTCTAGTATTTATTAATGTAAAATACATAGCAGAGTTGCTAACTTTACCTTGAACATTTATTGTAGGTCTGGCCCAAGGCTGA 719880 720000

NC_000963_1_Rickettsia_prowazekii_str__Madrid_E_chromosome_complete_genome probe_cstm_Rprow_021218_1x_105254 ACAAAGTGCACTGCTGCTTCGGCAGGTAATAGATCATCGGACTGATTAGAAGCAACATCAACATTAGAAGCAACACTGGTAAGATTAGGATTTTGAGTCGTTTGATCAGCATAGCTTAAC 720000 720120

NC_000963_1_Rickettsia_prowazekii_str__Madrid_E_chromosome_complete_genome probe_cstm_Rprow_021218_1x_105255 GCACAAATTAAATTAATAAAACTAACTAATACAGTTTTTAACATAAAAGCACCTGTGATAAATTAAAATACGATATTATCATAAGTTATCTTTTTTGTAAAATATTCTTCTTTAGAAAAT 720120 720240

NC_000963_1_Rickettsia_prowazekii_str__Madrid_E_chromosome_complete_genome probe_cstm_Rprow_021218_1x_105256 TTTCCTATTGCATAATGCCCTCCATTTCTTAGGGTTTTCTTTATATAATAACCTTACTTACTACAGTAGTATCCAATATGCATAAAATATTTATCCTTCAGAAATGGTATCTAATTATTA 720240 720360

NC_000963_1_Rickettsia_prowazekii_str__Madrid_E_chromosome_complete_genome probe_cstm_Rprow_021218_1x_105257 TTAGAATAAAATATACATTTAAAGAATATAGCGATTAACATTAGACTAAACATTATATTAAATAATTATGATTATATGGAAAGCTGACGATTCTACTAAATTCATTCCAATGATTTATGT 720360 720480

NC_000963_1_Rickettsia_prowazekii_str__Madrid_E_chromosome_complete_genome probe_cstm_Rprow_021218_1x_105258 ATATTCTTATACTTTCTAAAAAATACTATTCATCAATTTTAGGTTGAAAACATAACTTAAATCTTCGTACGCTAAATTACAACACCTTTAATAATCGATACAATAAAACAAATTATCTCC 720480 720600

NC_000963_1_Rickettsia_prowazekii_str__Madrid_E_chromosome_complete_genome probe_cstm_Rprow_021218_1x_105259 ATCATTAGTATTAAAGAATAGCATTATACACTCCAAAAAATATCTACATTCTAAGTAAAATATCTCTAATTTGTGATAAATTTTCTTTTCAATAAACTATTTTTGAAAATATAATATAGA 720600 720720

NC_000963_1_Rickettsia_prowazekii_str__Madrid_E_chromosome_complete_genome probe_cstm_Rprow_021218_1x_105260 AATATCACCTAAATTTGCCATTGCGATCATAAGAAGCAATTATTACTAACTGATGCATGTTAGTTATAATCTAAAATACCTCTATAATATATAATTATACCAAAAGTTTTAGAATGATTT 720720 720840

NC_000963_1_Rickettsia_prowazekii_str__Madrid_E_chromosome_complete_genome probe_cstm_Rprow_021218_1x_105261 TAATTATTTAGTAAATCATGTTAATTAATAGTGACCAAACTATTTTTTGAAATGCGTTTCCTAAAAACATATAGTGGTATTACAGTTAAATTAATTATATTTTGCTCTGATCTTTTTAGC 720840 720960

NC_000963_1_Rickettsia_prowazekii_str__Madrid_E_chromosome_complete_genome probe_cstm_Rprow_021218_1x_105262 AAAATCACACCTTTCTTTATAACGCATTACACGCATTGAACCAAGAAGCACACAAGCAAAGTGTACTTTCAGCTCATTTCTTACTATAGTATACTCTCTAGTTTAATATTATATTTACAA 720960 721080

NC_000963_1_Rickettsia_prowazekii_str__Madrid_E_chromosome_complete_genome probe_cstm_Rprow_021218_1x_105263 TTTATAGGTAATAATTAAATACGTTAAAAACAGCACTTAATTATTTAGTGAATGCAGGATACTAAAATACCAGAATATTGATAATACAGCAACAGAAGACAAAACATAACGGAATAATAG 721080 721200

NC_000963_1_Rickettsia_prowazekii_str__Madrid_E_chromosome_complete_genome probe_cstm_Rprow_021218_1x_105264 ACAAGGATAAAATAGAGAAAATTACTACTATGTTGTATAGAAATAACAAATTGAAAAAGTATTTGGGACTACACTAAATAGTATATAAAAATCTTGAAAAATTGTATTAAAGTCTGTCAG 721200 721320

NC_000963_1_Rickettsia_prowazekii_str__Madrid_E_chromosome_complete_genome probe_cstm_Rprow_021218_1x_105265 TTTATGTATAAAAGCTAATAGAACTATTATAATATAACACAGATGAACACAGAGACACAAATTTCACCAAATATAGGTAATACAAACAATACCTTATAATAATTTGTAAAAAATATAATA 721320 721440

NC_000963_1_Rickettsia_prowazekii_str__Madrid_E_chromosome_complete_genome probe_cstm_Rprow_021218_1x_105266 GATCAAATTATCATTTCATTTTTAATGAACTTGGTAATTTAGATCAGAAGTCATTAAAACAACAGATCCAATTTCTACAAAAAATGCTTGGACACattattattcgacgataggaatata 721440 721560

NC_000963_1_Rickettsia_prowazekii_str__Madrid_E_chromosome_complete_genome probe_cstm_Rprow_021218_1x_105268 TCAAATTAATAATCATGCATTTCCACATAAAAAATTATTTTCTGCTTAATGATAATAGAGATAAAAAGAAATATGAATAGTATTTTTATAGTGATTTTGATTTGTTATACCCTGTaaaaa 721680 721800

NC_000963_1_Rickettsia_prowazekii_str__Madrid_E_chromosome_complete_genome probe_cstm_Rprow_021218_1x_105270 ttaatttcatttaatatattttatCTCATTACTAATTAGATAAGCTTTCTCCAAACTTACAATAGAATAATAAGTAGTTTTACAGTTAGAAGTTAATTAGAAGTATTCATAAGGTTGTAT 721920 722040

NC_000963_1_Rickettsia_prowazekii_str__Madrid_E_chromosome_complete_genome probe_cstm_Rprow_021218_1x_105271 CaaaaaaaTACTAGACGTTACTTATATAAACAGTAAGAAGCTCTAGTTTACAAGTATTTACTATTTATCAATTGGAATAGAAATGAAAATGGTGGAGGCAAAGGGAATCGAACCCTTGAC 722040 722160

NC_000963_1_Rickettsia_prowazekii_str__Madrid_E_chromosome_complete_genome probe_cstm_Rprow_021218_1x_105272 ACTCTGCGTGCAAAGCAGATGCTCTACCCCTGAGCTATGCCCCCAATTGTGTAGAAGATACTCTATGTAAGAAAATTAAGATAAGTTTTTCACTAATCCCCAAGGTAATGGCTGATTATT 722160 722280

NC_000963_1_Rickettsia_prowazekii_str__Madrid_E_chromosome_complete_genome probe_cstm_Rprow_021218_1x_105273 ACTGCCTACCACTAAATCTCCTGAAGATTCATACTCAGCAGCCATAAAAGAATTTTGAGCAGAAATAAGCTTTACCGGTTTAATTTTCTGACCGTTATGCAAATACTCCTTAGCCTGCTC 722280 722400

NC_000963_1_Rickettsia_prowazekii_str__Madrid_E_chromosome_complete_genome probe_cstm_Rprow_021218_1x_105274 CCTAGAAGTCGGATTATTTTTAGAAGCTGTTTTTGCTTTAGCTTTAGACATATACTATTCACTTTGTTAGACTTGTTAATAATATAAGACTTTTCACTAAACTTGTCAATCACTACCTTT 722400 722520

NC_000963_1_Rickettsia_prowazekii_str__Madrid_E_chromosome_complete_genome probe_cstm_Rprow_021218_1x_105275 TAGTATCTACAGAATATTATTATTCTTGCGTTATTATTTCACTCTCAGCAGCTAATGCTATTTCAAATTTTACTTGTGTTATTATCATATATACTAACCTCTACTTCTCTAAAGTCTAAT 722520 722640

NC_000963_1_Rickettsia_prowazekii_str__Madrid_E_chromosome_complete_genome probe_cstm_Rprow_021218_1x_105276 TGTGCGTTGTTATTAGTACAACACCAATTTTATATATTGCGAAGCACATATAAAATTTTTAGAAATGGCAAAATTGATTTTCAGAACTTTCTATAACCAACTACAATATCTTAAGTCATA 722640 722760

NC_000963_1_Rickettsia_prowazekii_str__Madrid_E_chromosome_complete_genome probe_cstm_Rprow_021218_1x_105277 ATTTTGTTATATCCAAGCACTTACTGATTCATATATAATGTTATAATGTAATCCTAGCTATACTATTATGACGCCTTATTAATATTTGATATTCTGATATTTTATATAAGTTATGTGACA 722760 722880

NC_000963_1_Rickettsia_prowazekii_str__Madrid_E_chromosome_complete_genome probe_cstm_Rprow_021218_1x_105278 TGCATCTAAATTGCAAAGAGTATCATGATCATTGATTATACTAACACATATGAACATTTCCTTTTTTCTGCAATAACCATATAGCAATTTTAAATGCATGATATTAAAATGTCGTGGACA 722880 723000

NC_000963_1_Rickettsia_prowazekii_str__Madrid_E_chromosome_complete_genome probe_cstm_Rprow_021218_1x_105279 ATTCAGTAATAGATACGCCACACACACTATATCAAAAACACTATTGCCGAAAAACTAAATTGTTTTAATAAATTGTATATATAAAACAATAGGATAAGTTATCATTTACTATCTTCATGG 723000 723120

NC_000963_1_Rickettsia_prowazekii_str__Madrid_E_chromosome_complete_genome probe_cstm_Rprow_021218_1x_105280 TAATATAATTACTACTGTAAATAAAAGGATGCTTATTTAAGTGTAAATCAGGCATACCGTATTATACTAATATAAATCAGGCAATAAATTACCTTGAACAAACGTACCATACAAGATAAT 723120 723240

NC_000963_1_Rickettsia_prowazekii_str__Madrid_E_chromosome_complete_genome probe_cstm_Rprow_021218_1x_105281 AATAGCGCTTTTACTCTTCAATATCTCAATCTACTACATTATAAAATTCAGCGTAGCTTAGATTTTTGAAAACACTAACGTAAAGTTTATTTGATTGATTTATTCATTTACACCATGACA 723240 723360

NC_000963_1_Rickettsia_prowazekii_str__Madrid_E_chromosome_complete_genome probe_cstm_Rprow_021218_1x_105282 ATATTTATATTTCTTACCTGAACCACACGGACACAGCTCATTGCGTGATACTCTACCCCAACTCATAGGATCATCAGGTTTTCTATCTTTTGGATCAACACGTGAGACGACCGGTTTAAG 723360 723480

NC_000963_1_Rickettsia_prowazekii_str__Madrid_E_chromosome_complete_genome probe_cstm_Rprow_021218_1x_105283 ATAAGTTTCAATACTATTTCCTGCATTATATTTACTAAATGCTGGATCTTCCCGACTCTCACGCATAtttttttGAAGCTTTTTATGTTCAAGCGAAATATCTTCTTTTTGTAGATGTTT 723480 723600

NC_000963_1_Rickettsia_prowazekii_str__Madrid_E_chromosome_complete_genome probe_cstm_Rprow_021218_1x_105284 TAAGTCGATATGGAAGTGATATACAGTTTGAATAAATAATTCTTTTAAATTATTAAGCATCTGCTCAAATAAATTAAATGCTTCTCTTTTATACTCGCTCAGAGGATCTTTTTGTGCATA 723600 723720

NC_000963_1_Rickettsia_prowazekii_str__Madrid_E_chromosome_complete_genome probe_cstm_Rprow_021218_1x_105285 AGCTCGAAGTGAAATACCTTGCCTTAAATGATCTAAGCTATATAAATGATCTTTCCAAACTTGATCAAGAGTAGTTAGTAAAATATATTTTACCGCATTATGCATTAACTCACTACTATA 723720 723840

NC_000963_1_Rickettsia_prowazekii_str__Madrid_E_chromosome_complete_genome probe_cstm_Rprow_021218_1x_105286 TGCTTCTTCTTTTGACTTATATATATCATGAGCCATTTGTATAACAAGCTTCGTGATATCTTCTTCTGTTACATCATTTTTATTTACTAAATTATGATCAAATTTTATAGAAAAGATACG 723840 723960

NC_000963_1_Rickettsia_prowazekii_str__Madrid_E_chromosome_complete_genome probe_cstm_Rprow_021218_1x_105287 ATGTAATTCTACAGTTAAATTTTCTATATCCCAATCTTCTCTATAAGAGCCCACTGGAATAAAAGTTAATACTAtttttttAGCGAGTTCCTCAGTAGCACTAGTTAAGAAACCATAACT 723960 724080

NC_000963_1_Rickettsia_prowazekii_str__Madrid_E_chromosome_complete_genome probe_cstm_Rprow_021218_1x_105288 ATCTTTAGATTTAATAATTTCAGTACGTTGCTCATATATTATCTTACGTTGATCATTCATTACATCATCAAAACGTAATAAATTTTTACGCATCTCGTAGTTATATTCTTCCACCTTTTG 724080 724200

NC_000963_1_Rickettsia_prowazekii_str__Madrid_E_chromosome_complete_genome probe_cstm_Rprow_021218_1x_105289 CTGAGCTTTTTCAAGTGAGCGACTAATCATTGGATGGTGAATAGCTTCTCCATCTTTTAAGCCAAGTGTTCTAAGAACTCCTGATATACGATCTGATGCAAAAATACGCATTAAATCATC 724200 724320

NC_000963_1_Rickettsia_prowazekii_str__Madrid_E_chromosome_complete_genome probe_cstm_Rprow_021218_1x_105290 ATCAAGTGATaaaaaaaaTTGTGTTTTACCAGGATCACCTTGTCTTCCTGATCTTCCTCTTAACTGATTATCTATCCTGCGGCTTTCATGCCTTTCCGTACCTATCACAAATAAGCCACC 724320 724440

NC_000963_1_Rickettsia_prowazekii_str__Madrid_E_chromosome_complete_genome probe_cstm_Rprow_021218_1x_105291 TGTTTCAATAACCTTCTGCTTTTCCTCAGCAATTTGAGCTTTGATTTCAGCTGTTTTAGCTACATAATTATGATCTTTATTAAACTGTTCTATTAACATTTCAGGATTACCGCCAAGCAT 724440 724560

NC_000963_1_Rickettsia_prowazekii_str__Madrid_E_chromosome_complete_genome probe_cstm_Rprow_021218_1x_105292 AATATCAGTACCTCGCCCTGCCATATTGGTTGCAATCGTTACAGCTTTAAACCTACCTGCTTGAGCAATAATGAAAGCTTCTTGCTCATGAAATTTAGCATTTAATACTTTATGAGGTAT 724560 724680

NC_000963_1_Rickettsia_prowazekii_str__Madrid_E_chromosome_complete_genome probe_cstm_Rprow_021218_1x_105293 TTTTTCTTTATTTAAAATACTTGAAAGTTCTTCTGACTTTTCAATGCTTATAGTACCAACAAGTATAGGCTGACCACGATTGTAACAATCCTTAATCAGCTTTAAGATAGCATCATATTT 724680 724800

NC_000963_1_Rickettsia_prowazekii_str__Madrid_E_chromosome_complete_genome probe_cstm_Rprow_021218_1x_105294 TTCTTGTTTACTTCCGTAAATTTCATCATCAAGGTCAATCCTTGTAACTTTATTATGTGTTGGGACCGCGACCACATCCAAATTATATATATCTTTTAATTCTGATGCTTCAGTCATAGA 724800 724920

NC_000963_1_Rickettsia_prowazekii_str__Madrid_E_chromosome_complete_genome probe_cstm_Rprow_021218_1x_105295 TGTACCAGTCATGCCAGCTAATTTAGGATAATTACGAAAGTAATTTTGGAATGTAATAGATGCCAAAGTTTGATTCTCATTTTGAATTTTCACATTTTCTTTTGCCTCTAATGCTTGGTG 724920 725040

NC_000963_1_Rickettsia_prowazekii_str__Madrid_E_chromosome_complete_genome probe_cstm_Rprow_021218_1x_105296 CAAGCCTTCTGAATATCTACGACCTTCCATTACACGCCCAGTAAATTCGTCTATAATCATTACTTTACCTTCACGCACCAAATAATCAACATCAATAGTAAACATATGATGAGCTCTTAA 725040 725160

NC_000963_1_Rickettsia_prowazekii_str__Madrid_E_chromosome_complete_genome probe_cstm_Rprow_021218_1x_105297 TGCTTGATTAACGTAATGTACTAAATTTAAATTCTCAAAATCATATAAACCAGTATCAGGTTTTATAATACCATCTTTAATTAATAACGACTCTATATGAGTAATACCTGCTTCTGTTAA 725160 725280

NC_000963_1_Rickettsia_prowazekii_str__Madrid_E_chromosome_complete_genome probe_cstm_Rprow_021218_1x_105298 GTTAATAGTTTTCAGTTTTTCATCTTTTTCAAAATCACTTACATTAAGTAAACGTACAATTTTATCAATTTTACCGTATAATGCTGAATTATCATTAACTGGACCTGAGATAACTATCGG 725280 725400

NC_000963_1_Rickettsia_prowazekii_str__Madrid_E_chromosome_complete_genome probe_cstm_Rprow_021218_1x_105299 GGTTCTTGCTTCATCTATCAAAATCGAATCAACTTCATCAATAATAGCAAAATTAAAAGGTCGAAGTACGCGCTCTTGCATACTATACTTCATATTATCCCTTAAATAGTCAAAACCAAG 725400 725520

NC_009488_1_Orientia_tsutsugamushi_Boryong_complete_genome probe_cstm_Rprow_021218_1x_1053 ATACTGTCATATATTTCATATGATTTATAAAAATTCCCCTTTAAATTTTTGATTAGCTATGATAATTGCTAAATCAAATGTGCTGTTATCAAattttcttttttacagccttatttttaa 126240 126360

NC_000963_1_Rickettsia_prowazekii_str__Madrid_E_chromosome_complete_genome probe_cstm_Rprow_021218_1x_105300 TTCATTATTTGTTGCATGAGTAATATCGGAATTATATGCAATCCGTTTTGCTTCATCGGTCATACCTGCGACAATACATCCAACCGATAATCCTAAAAAATTATAAATTTTACCCATAGA 725520 725640

NC_000963_1_Rickettsia_prowazekii_str__Madrid_E_chromosome_complete_genome probe_cstm_Rprow_021218_1x_105301 AGCAGAATCACGGAGTGCTAAATAATCATTCACAGTTACAACATGTACACCTTTTTCAGTCAAAGCGTTTAAATATGCAGGAAGCGTTGCAACTAAGGTTTTACCCTCACCAGTACGCAT 725640 725760

NC_000963_1_Rickettsia_prowazekii_str__Madrid_E_chromosome_complete_genome probe_cstm_Rprow_021218_1x_105302 TTCCGCAATCATGCCACGATGTAATATAATACCGCCTATAAGCTGAACATCAAAATGTCGCATACCGCAAACTCTGCTAGCCGCTTCTCTCACTACCGCAAACGCTTCATATAAGATATC 725760 725880

NC_000963_1_Rickettsia_prowazekii_str__Madrid_E_chromosome_complete_genome probe_cstm_Rprow_021218_1x_105303 ATCTAAAGTAGCTCCGTTTTTAAGTTTTTCTTTAAACTCTACGGTTTTATTCTTCAACGCCGCATCTGATAATATTTTTATTGCAGGCTCAAATGAGTTAATTTTTGTAATTTCAGAAAA 725880 726000

NC_000963_1_Rickettsia_prowazekii_str__Madrid_E_chromosome_complete_genome probe_cstm_Rprow_021218_1x_105306 TTTATAAAATTTAAGCTTATGTTTAACACAATGATGAGTCATAAATGAAAAAATTATCTGTTATATTTTTATCAGTTAGCATGCTTTCCAGTATTGCTTTTGGTGACGAAGATAAAGTAG 726240 726360

NC_000963_1_Rickettsia_prowazekii_str__Madrid_E_chromosome_complete_genome probe_cstm_Rprow_021218_1x_105307 TAGCTACATATAAAGGTGGTGAAGTAAAAGAATCGCAAATTATGCAAGAATTTAAGCCTCAACTCAATCTTCAATCCGGCGAAACAATTAAAAATTTCGATGATTTTCCACTACAAGATC 726360 726480

NC_000963_1_Rickettsia_prowazekii_str__Madrid_E_chromosome_complete_genome probe_cstm_Rprow_021218_1x_105308 AGGAAAAACTAATAAAAATTTATGTTAATAATCTTTTGTTAAAAGAAGAAGTTGCTAAATCAAGTATTACTTCATCTAAAGAGTTTCAAGAAAAACTTGAAAATGCAAAAAATCAATTAG 726480 726600

NC_000963_1_Rickettsia_prowazekii_str__Madrid_E_chromosome_complete_genome probe_cstm_Rprow_021218_1x_105309 CGCAGCAAGAATTACTAGCAAATTATATAAAATCTAACATTACAGATAAGATGTTTGATGACGAATATAATAAATATGTTGATAACCTTAAAGGTAAAGAACAAATAAAGGTTGCTCATA 726600 726720

NC_000963_1_Rickettsia_prowazekii_str__Madrid_E_chromosome_complete_genome probe_cstm_Rprow_021218_1x_105310 TTTTAGTTAAATCTCAAAAAGAAGCTAATACTGTAAAGACCAAATTAAGCAAAGGGGGGAATTTTACTAAGCTTGCAGAAGAATTCTCTCTTGATAAAGCTACAGCGTCAAATGGTGGGA 726720 726840

NC_000963_1_Rickettsia_prowazekii_str__Madrid_E_chromosome_complete_genome probe_cstm_Rprow_021218_1x_105311 TTATAGGTTATATTATACTAAATCAACCAGGGCAGTTAGTACCAGAATTTGAACAGAAAGCTTTTGCATTAAAAGTAAATGAAGTTTCAACTCCAGTCAAAACAAGTTTCGGTTGGCATA 726840 726960

NC_000963_1_Rickettsia_prowazekii_str__Madrid_E_chromosome_complete_genome probe_cstm_Rprow_021218_1x_105312 TTATAAAAGTGCTTGaaaaaaaaCCTGTGCCTATTCCAACAAAAGAAGAAGCAAAAGTAACTATTGATAATATATTAGCTGCAGAAATATTAAAGCAATATATTTCTGATTTAGAAGCTA 726960 727080

NC_000963_1_Rickettsia_prowazekii_str__Madrid_E_chromosome_complete_genome probe_cstm_Rprow_021218_1x_105313 AAGCTGATTTAAAAATTATGTTACCTAAAGCAAACAGCAAAACTGGATCTTAATTTATTTAATATTTCTTAGCTAAAAAGAGCAGCAGCGTATCGATTACATTACTGCGAAAGCAACAAA 727080 727200

NC_000963_1_Rickettsia_prowazekii_str__Madrid_E_chromosome_complete_genome probe_cstm_Rprow_021218_1x_105314 TCTAGCAAAATCTATaaaaaaaaCTAATTTTTATAGATTCTTGCCTTTGTATGAATGATATCGTATAGATTTTTAAATAATGCGACGCACTATAAGATTACCTAAAAGGTATACTTAACA 727200 727320

NC_000963_1_Rickettsia_prowazekii_str__Madrid_E_chromosome_complete_genome probe_cstm_Rprow_021218_1x_105315 ATTACTTTCAATTATGGCAAACGCTATGCATATAGGATAATCATCAGAAAGAGAAAGGTGAATATTAAAGGGGGCGAGTTTATTTGTATAGTGCGAACTTATTTCTACTGTAGGTTTGCC 727320 727440

NC_000963_1_Rickettsia_prowazekii_str__Madrid_E_chromosome_complete_genome probe_cstm_Rprow_021218_1x_105316 TAAATTATCATTTAGTATAGTAATATCTTTAAAATTTATACCTCGACCTATACCTACTCCAAATGCTTTACTAACAGCCTCTTTAGCAGAAAAACGCTTTGCTAAAAAAGTAGCATGGTT 727440 727560

NC_000963_1_Rickettsia_prowazekii_str__Madrid_E_chromosome_complete_genome probe_cstm_Rprow_021218_1x_105317 TGTTTTATTCAATAAAGTAAACTGTTTTAATTCTTTTAATGCGAGAATTTTCTTGGCAAAAAGTTCTTGATATATATTTAATATTTTTTCAATTCTAGGAATTTGTACTATATCAGTACC 727560 727680

NC_000963_1_Rickettsia_prowazekii_str__Madrid_E_chromosome_complete_genome probe_cstm_Rprow_021218_1x_105318 GACACCAATCAGCATTTACTTATCGGTTTCATAATCTTCGCCATTCTCATTAATAAAGATTTGATCATCTAAATCAGAATAATCTTCTCCGATATAAACTTCAGGTAAATATTCAAATTT 727680 727800

NC_000963_1_Rickettsia_prowazekii_str__Madrid_E_chromosome_complete_genome probe_cstm_Rprow_021218_1x_105319 TTCTTCTACATCTTCTGATTCATCTTGATTAATAAGTGGCTCTATCATCATTCTGGTACGCAAACTATTTATTAAATCTTGTTCTAACTGAGGCACCGATACTTTACCTGCAGCAATTCT 727800 727920

NC_000963_1_Rickettsia_prowazekii_str__Madrid_E_chromosome_complete_genome probe_cstm_Rprow_021218_1x_105320 ACGTAATGAGATAACAGGAGGCTTATCACGCTTTTCTTTTTTAATTTGATTAGTTTCCACTTTATAATTTAATAATTTCGCATATCTTGTAGCTAGTACAACAAGTCGAAATCTATCAGG 727920 728040

NC_000963_1_Rickettsia_prowazekii_str__Madrid_E_chromosome_complete_genome probe_cstm_Rprow_021218_1x_105321 TATAATTTTATTACAATCTTCTGCTGTAATTCTTGCCATAATTTTCTCCGTTAAAAATTTTTCATTGTAATATAAATTGACTACAGTGTCTTACAAGCAACTCACAGTACAAATCCATTA 728040 728160

NC_000963_1_Rickettsia_prowazekii_str__Madrid_E_chromosome_complete_genome probe_cstm_Rprow_021218_1x_105322 TTAAATTAAGCTGGAGCCTGTATTCAAAGCTATAAACAACAGCTATAACCAACAATAATAGCATATTGGGTTATTATACCCTTTTTATATTAGCACCGCAATTACTTAAttttttttCTA 728160 728280

NC_000963_1_Rickettsia_prowazekii_str__Madrid_E_chromosome_complete_genome probe_cstm_Rprow_021218_1x_105323 AATCTTGAAAGCCACGATCTAAATGATATATTCGATGTAATACGGTCTTACTATTAGTACTAAGACCAGCAAGTATTAGCGATACCGAAGCTCTAAGGTCACTCGCCATAACTTCTGCTC 728280 728400

NC_000963_1_Rickettsia_prowazekii_str__Madrid_E_chromosome_complete_genome probe_cstm_Rprow_021218_1x_105324 CTTTTAACATCTCTACACCTCGAACTACCGCCTTATTACCTCGCACTACTATATCTGCACCCATTCTACATAACTCAGGCACATGCATAAAACGATTTTCAAAAATATTCTCAGTAATCA 728400 728520

NC_000963_1_Rickettsia_prowazekii_str__Madrid_E_chromosome_complete_genome probe_cstm_Rprow_021218_1x_105325 TTGAAACACCACTACTAATCGACATAAGACTCATAAATTGAGCTTGTAAATCAGTAGCAAAACCAGGATAGGGATTAGTTTCTAAATCAACTGAATTTAATTTTCCTTTATAAGTTACTT 728520 728640

NC_000963_1_Rickettsia_prowazekii_str__Madrid_E_chromosome_complete_genome probe_cstm_Rprow_021218_1x_105326 GAACTCCATTATTGATCTGCACAACTTTTATACCAGTTTCAATAAGTTTTAATGCTATATTTTCTATAATATTGTAATCAATCCCACAAATTTTTACATCACCTTTAGTAATAGCTGCAG 728640 728760

NC_000963_1_Rickettsia_prowazekii_str__Madrid_E_chromosome_complete_genome probe_cstm_Rprow_021218_1x_105327 CAAACATATAAGTACCTGCTTCAATACGATCGGAAAGTACTTTATAACTTGTTTCATTTAAACAATCTTTACCTTTAATCGTTATTGTACTACTTCCAACCCCTAAAATATCAGCACCCA 728760 728880

NC_000963_1_Rickettsia_prowazekii_str__Madrid_E_chromosome_complete_genome probe_cstm_Rprow_021218_1x_105328 TTTTTATAAGACAATAACAGAGATCTACTATCTCTGGTTCACGGCCACAATTAAAAAGCACAGTTTCCCCTTTTGCTAAAACTGCTGCAAGTATTGCATTAATAGTAGCCCCAACAGAAA 728880 729000

NC_000963_1_Rickettsia_prowazekii_str__Madrid_E_chromosome_complete_genome probe_cstm_Rprow_021218_1x_105329 CTTTATCAAAAATAAAATGTGTGCCTTTTAAACGTCCTTTACTTGAAGCATTAATATATCCATCCTCTATTTCAATCTCAGCACCCATAGATTTTAATACAGCGATATGTAAATCTACTT 729000 729120

NC_000963_1_Rickettsia_prowazekii_str__Madrid_E_chromosome_complete_genome probe_cstm_Rprow_021218_1x_105330 GTCTGGCTCCAATCGCACAACCACCAGGAAGTGATACTTTTGCTTTACCATATTTGGTAAGCAAAGGACCAAGAACCCAAATTGACGCTCTCATTTTACGTACTATTTCATAATCTGCAG 729120 729240

NC_000963_1_Rickettsia_prowazekii_str__Madrid_E_chromosome_complete_genome probe_cstm_Rprow_021218_1x_105331 TAAATTTATTTATATTTGTGGTATTAATTATAAGCTCGAATTCATCCGTGTGTTCTATTATTCTAATACATGCACCATGATTTCGGAGCAAATCTTTCATAGTACTAACATCTGTTAATT 729240 729360

NC_000963_1_Rickettsia_prowazekii_str__Madrid_E_chromosome_complete_genome probe_cstm_Rprow_021218_1x_105332 TTGGAACATTAGTAATATGTAGTTTATTTGTAAGAATAGACGCAGCCATAATAGGTAATACTGCATTTTTAGCACCGCTAATATTAATACTACCTTCAAGGGGCTTACCACCGTGAATAA 729360 729480

NC_000963_1_Rickettsia_prowazekii_str__Madrid_E_chromosome_complete_genome probe_cstm_Rprow_021218_1x_105333 TTAATTTTTGCATAAATTATTACTGTACAGATTGATTGTACTCTTTTATCATTACGTGATTAAGCCACATAATTATACCAAATAACTTTATTTATCTCAGTACAGGTATTAACATCATAC 729480 729600

NC_000963_1_Rickettsia_prowazekii_str__Madrid_E_chromosome_complete_genome probe_cstm_Rprow_021218_1x_105334 ATCTAAATTCATAACATTTAAAGCATTAGCTTGGATAAATAATCTACGAGGCTCAACAACATCACCCATTAAAGTAGAAAAAATCCCCTCTGCTTCATCTACTTCAGCAACTCTTACTTG 729600 729720

NC_000963_1_Rickettsia_prowazekii_str__Madrid_E_chromosome_complete_genome probe_cstm_Rprow_021218_1x_105335 AAGCAACGTTCGCTTTGTAGGATCAAGAGTAGTTTCCCACAGCTGATCGGAATTCATCTCTCCAAGACCTTTAAAACGCTGAATAGTTATACCTTTTTTACCGCATTCTATAATAGTATT 729720 729840

NC_000963_1_Rickettsia_prowazekii_str__Madrid_E_chromosome_complete_genome probe_cstm_Rprow_021218_1x_105336 TAACAACTGGCTTGGGGTTAAAATATCAAATGCCTTACTTTTTACTATAAGCTTTAATCGCTTACTAAATATATCAAAAATTGATAAAGCAAACTGTGATATTTCTACAAATTCAAATAA 729840 729960

NC_000963_1_Rickettsia_prowazekii_str__Madrid_E_chromosome_complete_genome probe_cstm_Rprow_021218_1x_105337 TTCTAACTGCTCTTTTAACAAAATTTTAGTTTCTTTTAAACCTCTACTAAAATGAAAAAATTCTATTTTATTCTCATGTTTTAACACTTGCAAATTAGTTTTATCTGGTGACTCCTCTAA 729960 730080

NC_000963_1_Rickettsia_prowazekii_str__Madrid_E_chromosome_complete_genome probe_cstm_Rprow_021218_1x_105338 ATTATTTAAAATATCTAAAGCTTTTTGAAGCCTTGCACTACTTTCAGGTTCAAATAtttttttATTAAATAAGTCATTAATTGCAAGAATTTCAGTAATTGAGCGATTAAATTTTTTACT 730080 730200

NC_000963_1_Rickettsia_prowazekii_str__Madrid_E_chromosome_complete_genome probe_cstm_Rprow_021218_1x_105339 AACATGATCAAGCAAATTGTTAAACTTAACTACTTTATTAATTAGATCTTCTAAATTCTCACCTACTAACTGCTCTTGACCGTCTAGAATTAAGTATGTATCATTAATAGTTGATTTTAT 730200 730320

NC_000963_1_Rickettsia_prowazekii_str__Madrid_E_chromosome_complete_genome probe_cstm_Rprow_021218_1x_105340 TAAATAATTTTGCAAAGCTTTTTCATTCTTTAAATAAAGCTCAGATGCACCACTTTTTACCTTATAAAGCGGTGGCTGTGCTATATATAGATAACCTTTATTTATAAGCTCTGGCATATG 730320 730440

NC_000963_1_Rickettsia_prowazekii_str__Madrid_E_chromosome_complete_genome probe_cstm_Rprow_021218_1x_105341 ACGATaaaaaaaTGTGAGCAACAAAGCTCTAATATGCGAACCATCAACATCAGCATCGGTCATAATAATAACTTTATGGTATCTCAGCTTTTCTAAAGAAAATTCTCGTTCAACACTAAT 730440 730560

NC_000963_1_Rickettsia_prowazekii_str__Madrid_E_chromosome_complete_genome probe_cstm_Rprow_021218_1x_105342 CCCAAGAGCTGTAATTAATGTACCAATTTGGTCCGAACCAAGCATTTTATCAAATCTTGCTCGTTCAACATTCAAAATTTTACCACGCAAAGGCAGTATAGCCTGAATTTTGCTATCCCT 730560 730680

NC_000963_1_Rickettsia_prowazekii_str__Madrid_E_chromosome_complete_genome probe_cstm_Rprow_021218_1x_105343 TCCCTGCTTTGCAGTACCACCCGCAGAGTCCCCCTCAACAATAAACAATTCTGAAATAGCAGGATCTTTGGCATGGCAATCTGCAAGTTTACCTGGTAAATTTGAAACTTCTAAAGCTGA 730680 730800

NC_000963_1_Rickettsia_prowazekii_str__Madrid_E_chromosome_complete_genome probe_cstm_Rprow_021218_1x_105344 TTTTCTTCGAGTAAGTTCTCTTGCTTTTCTAGCAGCTTCACGAGCATTGGCAGCTTCCATAATCTTAGCTATAATTGCCTTAGCTTCAGTAGGATGTTCTTCAAACCACTCAAGAACCTT 730800 730920

NC_000963_1_Rickettsia_prowazekii_str__Madrid_E_chromosome_complete_genome probe_cstm_Rprow_021218_1x_105345 TGTATAAACGGCATTCTCTACAACAGGTCGTACTTCAGAACTTACTAGCTTATCTTTTGTTTGAGAAGAAAATTTAGGATCAGGGACTTTAACAGATAATACACAGCAAATCCCTTCTCT 730920 731040

NC_000963_1_Rickettsia_prowazekii_str__Madrid_E_chromosome_complete_genome probe_cstm_Rprow_021218_1x_105346 AGTATCTTCACCACTAAAATCATGTTTAGtttttttATTAAGACCTGTAGTATCTAGATAAGAAGTGATAACACGTGTAATCGCTGATTTGAAAGCACTAAGATGAGTCCCACCGTCACG 731040 731160

NC_000963_1_Rickettsia_prowazekii_str__Madrid_E_chromosome_complete_genome probe_cstm_Rprow_021218_1x_105347 CTGCCTAATATTATTAGTGAAACATAGAATATTCTCATGATAAGAATCATTCCAATGAAGTGCAAGCTCAAGGCTTATACCTGACTCAACATGCACAGTATTTACTACTATACATGGATG 731160 731280

NC_000963_1_Rickettsia_prowazekii_str__Madrid_E_chromosome_complete_genome probe_cstm_Rprow_021218_1x_105348 AATAGCATGCTTCGCTCTGTCTATATATTGTACATAAGCTTCTATACCACCTGTATAATAAAACTCTACTTTTTTAACTTCTTCAAAGCGATTATCAACGAGTAAAATCTTAACGCCTGA 731280 731400

NC_000963_1_Rickettsia_prowazekii_str__Madrid_E_chromosome_complete_genome probe_cstm_Rprow_021218_1x_105349 ATTTAAAAATGCAAGCTCTCGAAGACGATGTTCTATAGTCACAAAATCAAATTCTATATTAGTGAATGTACCTACAGATGGGAAAAAAGTAACTTCTGTACCTTTTTTATCAATATTTTC 731400 731520

NC_000963_1_Rickettsia_prowazekii_str__Madrid_E_chromosome_complete_genome probe_cstm_Rprow_021218_1x_105350 TTTGACAATTGAAAGTGGAGCTTCAGTTATACCATTATTAAATCTAATAAAATATTCTTTATTATTACGCCAAATACGCAGCTCAAGCCACTCAGAAAGTGCATTTACTACCGATACACC 731520 731640

NC_000963_1_Rickettsia_prowazekii_str__Madrid_E_chromosome_complete_genome probe_cstm_Rprow_021218_1x_105351 AACACCATGCAGACCGCCTGAAATCTTGTAGGACTGCTGATCAAACTTACCACCCGCATGAAGCTGCGTCATAATTACTTCAGCTGCCGATATCCCTTCTTCTTCATGAATTTCAACAGG 731640 731760

NC_000963_1_Rickettsia_prowazekii_str__Madrid_E_chromosome_complete_genome probe_cstm_Rprow_021218_1x_105352 TATACCTCGTCCATTATCAGACACGGTTACTGAACCATTTTTATTTAATGTTACTTGTACTAAATCACAATAACCAGCAAGCGCTTCATCGATAGAATTGTCAACTACTTCATAAATCAT 731760 731880

NC_000963_1_Rickettsia_prowazekii_str__Madrid_E_chromosome_complete_genome probe_cstm_Rprow_021218_1x_105353 ATGATGTAGACCAGATCCATCTCCAACATCACCTATGTACATTCCTGGTCTTTTTCTTACTGCTTCAAGACCTTTTAATACCTTTATAGAATCAGCACTATATGATGATTCATTACATTT 731880 732000

NC_000963_1_Rickettsia_prowazekii_str__Madrid_E_chromosome_complete_genome probe_cstm_Rprow_021218_1x_105354 TTCTTCAATTACCGACATAATACATATACATATTGAACTTTATTACTTGTTGCTAGAAACAATAAAAATGACTATAACGTAACTTATTAAAATAGTAAAGTTTCTTGATTTAAAATTAGC 732000 732120

NC_000963_1_Rickettsia_prowazekii_str__Madrid_E_chromosome_complete_genome probe_cstm_Rprow_021218_1x_105355 GGGATAATATACATGAATACATTAAAGTTTAAAAATATTTTCGATGTAATCAATGATTATGATGTGTTTTTATTTGATCTGTGGGGAGTAATAATTGAAGGAAACCACACCTATCCAGGC 732120 732240

NC_000963_1_Rickettsia_prowazekii_str__Madrid_E_chromosome_complete_genome probe_cstm_Rprow_021218_1x_105356 GTCGTACAAAATATTAATAAAATTATTGCACAGAAAAAAGTATATTTTGTTACTAATGCCCCACGCAATATTTTTTCACTACATAAAACAATTAAATCTTGGGGAATAAATGCACTGCCA 732240 732360

NC_000963_1_Rickettsia_prowazekii_str__Madrid_E_chromosome_complete_genome probe_cstm_Rprow_021218_1x_105357 GAAATGATCATTAGCTCAGGTGAAATAGCAGTTCAAATGATACTAGAGAGTAAAAAGCGATTCGATATTACAAATCCGATAATATATCACCTAGGACATTTAGAAAATGATATAATAAAC 732360 732480

NC_000963_1_Rickettsia_prowazekii_str__Madrid_E_chromosome_complete_genome probe_cstm_Rprow_021218_1x_105358 CTAATGCAATGCTATACAACCGATGATATTAATAAAGCTAATATTTCCTTAATAACTATTTATAGAGACGAAAATGAAAAGTTAGATTTAAACGAATTCGATGAATTATTTAAAATCATT 732480 732600

NC_000963_1_Rickettsia_prowazekii_str__Madrid_E_chromosome_complete_genome probe_cstm_Rprow_021218_1x_105359 GTACAACGCAAAATTATTAATATTTGTGCTAATCCTGATCTTGGAATAAATCAACATGGTATTTATAGATATTGTAGCGGATATTATGCAGAAAAAATAAAGCAGCTTGGTGGAAAAGTA 732600 732720

NC_000963_1_Rickettsia_prowazekii_str__Madrid_E_chromosome_complete_genome probe_cstm_Rprow_021218_1x_105360 ATTTATAGTGGTAAACCATATGAAGAAATATATCACAAGATTTTAAAAGAATGTTCTAATATCCCTAAGAATCGTATGCTAATGATTGGAGATACTTTTTACACTGATATACTTGCAGCA 732720 732840

NC_000963_1_Rickettsia_prowazekii_str__Madrid_E_chromosome_complete_genome probe_cstm_Rprow_021218_1x_105361 AATCGCCTCGGTTTCGATTCTGCTCTTGTATTAACTGGAAATTCTAGAGAATATCATTTTGAATGTGATAACATTCATGAAAAATTAGATAGTTTAATGGAAGCTGCCATTAAACAATCA 732840 732960

NC_000963_1_Rickettsia_prowazekii_str__Madrid_E_chromosome_complete_genome probe_cstm_Rprow_021218_1x_105362 ATTATACCTAATTTTGTTATTGATTTATCTTGAATAACTTTATTATCCTTGTAAAATACCAAAATTGTTATATGTCTAGTTTATGCTATGGAAATTAGACATTGTTGTATAGTATCACTA 732960 733080

NC_000963_1_Rickettsia_prowazekii_str__Madrid_E_chromosome_complete_genome probe_cstm_Rprow_021218_1x_105363 CACTACTATCATAACGTGATTTATCCAAGAGATAAATACAGCGACTCTATTGATATTTTATTGATAATCCGTTATAGTTGTTATAGAGTAGAGTAAAATTTAAGCGGTCGTGGCGAAATT 733080 733200

NC_000963_1_Rickettsia_prowazekii_str__Madrid_E_chromosome_complete_genome probe_cstm_Rprow_021218_1x_105364 GGTAGACGCGCAGCATTGAGGGTGCTGTTCTGTAAAAGGATTGGAAGTTCAAATCTTCTCGACCGCACCATTCGACGATTTGTTACAAAGTTCAATTTTTCTGTCGTAACACTTCAGCTG 733200 733320

NC_000963_1_Rickettsia_prowazekii_str__Madrid_E_chromosome_complete_genome probe_cstm_Rprow_021218_1x_105365 TTATTATTGAATCCCAAGTTCTTATTGTGAAGAAATGTTTATGCTAGTTACATACTCATAATGAAAAGTAGACACAATAAAAACTATTGCTTATTTATAATCTGTGATTTATATTGATTA 733320 733440

NC_000963_1_Rickettsia_prowazekii_str__Madrid_E_chromosome_complete_genome probe_cstm_Rprow_021218_1x_105366 GCAAATTGTACACAATTCTGTACCAAGCCTAGCTAACTAACTTTAAATAATAAATAATTCTATAACAACTATTAGGTAAACAGGCTATGCCTAATTTCAATATGTTTAAAAGCATCTTAC 733440 733560

NC_000963_1_Rickettsia_prowazekii_str__Madrid_E_chromosome_complete_genome probe_cstm_Rprow_021218_1x_105367 CTGATCATCATAATCAATTTGCTGAGATTTTTGATCAAATTAACGATCTGATTGAAGATCACAATTATGATGCAGCTGCAAGTAGGCTTGCTAAGCTGCATTATGCAGATTTAGCAGATT 733560 733680

NC_000963_1_Rickettsia_prowazekii_str__Madrid_E_chromosome_complete_genome probe_cstm_Rprow_021218_1x_105368 TTTTAGATAATCTTAATAATAAAACATATAAAATCATTATTCCTTTATTACATGATAAAATTAAGCCTGAAACATTGGTATCTTTAAATGCTTATAGTAAACCTCTAATAATAGAAACTT 733680 733800

NC_000963_1_Rickettsia_prowazekii_str__Madrid_E_chromosome_complete_genome probe_cstm_Rprow_021218_1x_105369 TAGGCATCAAAAAATCTGCCGAATTAATCAATAAACTAGTTATAGAAGATGCAATTGAAGTAGTAATCGATCTAGACGATGATATAAAAGATCTGATACTAAGTAATCTGACTAAAGaaa 733800 733920

NC_009488_1_Orientia_tsutsugamushi_Boryong_complete_genome probe_cstm_Rprow_021218_1x_10537 TATAGAACCTACTTTTTGAGCTTGATCACGCAAAAACTCTGTTCTTTTGTTGGCTAACTCAATAAATATCTCTCCTTTACGATATAACTGCCAGTTGCTTAAAAAATGTTCGATATTATT 1264320 1264440

NC_000963_1_Rickettsia_prowazekii_str__Madrid_E_chromosome_complete_genome probe_cstm_Rprow_021218_1x_105370 aaaaaCACCAAATTACAGTAGGTTGTACATACCCTGAAAATACGGTAGGTAGAGTAATTGAACGAGATTTTATTAATCTTCAAGCAGGATGGACTGTTGAGGAATCATTAAATTTTATCA 733920 734040

NC_000963_1_Rickettsia_prowazekii_str__Madrid_E_chromosome_complete_genome probe_cstm_Rprow_021218_1x_105371 AGAACGTAAAGAATGAATTTTATGCAGCAATCGTTACAGATAATAAATTAAGACCTATTGGTATTATTTCTCTTAGTACACTAATTAAAGGCaaaaaaaaTGAATTGATAAAAGATTTGA 734040 734160

NC_000963_1_Rickettsia_prowazekii_str__Madrid_E_chromosome_complete_genome probe_cstm_Rprow_021218_1x_105372 TGAGCAAAGATTTTAAGCTTGCAGATACTTTTACCGACTTAAATGAACTAAGCTTCATTTTTAGACAATATGCTTTAACGATTGTACCTGTAGTAAATAAAAGCGGTAAGTTGGTAGGTA 734160 734280

NC_000963_1_Rickettsia_prowazekii_str__Madrid_E_chromosome_complete_genome probe_cstm_Rprow_021218_1x_105373 GTGTATCAATCGATAATATAATTTACATTATCGAAGAACAAGCAGGGAAAGATATATTATCATTAAGCGGGGTACACACACAAGATACTTTTTTCAATGTATTTTATACAGTCAAACATC 734280 734400

NC_000963_1_Rickettsia_prowazekii_str__Madrid_E_chromosome_complete_genome probe_cstm_Rprow_021218_1x_105374 GTTTTCCTTGGCTCTTTGTGAATCTCATTACTGCATGCATGACTTCACTTATTATTAACCATTTTAATGATACTATTGCAAAACTTGTAACACTTGCAGCAACTATGCCTATTGTAGCCT 734400 734520

NC_000963_1_Rickettsia_prowazekii_str__Madrid_E_chromosome_complete_genome probe_cstm_Rprow_021218_1x_105375 CAATGGGCGGAAATGCAGGAACACAAGCTATGACTGTTACTGTTAAAGCACTTGCTAATAAGGACATTCATTATAATAATGTCAATAAAGTAATATTAAAAGAAATCGCAGTATCAGCTT 734520 734640

NC_000963_1_Rickettsia_prowazekii_str__Madrid_E_chromosome_complete_genome probe_cstm_Rprow_021218_1x_105376 TTAACGGTTTTGTACTTGCAATTATCGGAGCAGGACTTAGCTTTGTCATGTTACTTGACTTAAATCTTAGCGTTATTTTTGCTATTGCAGTTATTTTAAATTTTTTAGTAGCAGGATTAT 734640 734760

NC_000963_1_Rickettsia_prowazekii_str__Madrid_E_chromosome_complete_genome probe_cstm_Rprow_021218_1x_105377 TTGGCTCTGCTATTCCTATAATACTGCATTATTTTGATATAGACCCAGCTGCAGGCTCAGGTGTATTTTTAACAACTATAACAGATGCGTTAGGGTTCCTAACTTTCTTAAGTCTTGCTA 734760 734880

NC_000963_1_Rickettsia_prowazekii_str__Madrid_E_chromosome_complete_genome probe_cstm_Rprow_021218_1x_105378 ATAtttttttAGTGTAATTGTTTATTAGCAAACCAACCTATAGAAATAATTATACGCAGTATTGCTACATACATAACAAAAAATGCAAAGCTAGGATCGACATAATATAAATTATTACAG 734880 735000

NC_000963_1_Rickettsia_prowazekii_str__Madrid_E_chromosome_complete_genome probe_cstm_Rprow_021218_1x_105379 CAAACAATATCTACAACTCAAGACATTTACATACAACTTGCATCATGATTTTTAAAAACGATAATTTTATCTTGGTATATTCCAATTATTATTAATAATCAATTCTTTGAACAATAAATT 735000 735120

NC_009488_1_Orientia_tsutsugamushi_Boryong_complete_genome probe_cstm_Rprow_021218_1x_10538 ACTACCAATCATTAAGACTTGCATGCTACTTTCAGCTGGTAAATTTTCATCACTTTTTAGAAATTCAGCAATTTCATTTTGAGCCGAAACACTAGCTTCAGCCAATGGGCATGCAAGCAA 1264440 1264560

NC_000963_1_Rickettsia_prowazekii_str__Madrid_E_chromosome_complete_genome probe_cstm_Rprow_021218_1x_105382 ATAAACTCGCATTTAGATTTAAATATGAATGGTGTAAGACCTTAGATATTCACGTTAAAAGGATCTACTAATGTAAGTATCAGTGTTCTAAACATAACCTTGGCAAATCAAAACTAAGAA 735360 735480

NC_000963_1_Rickettsia_prowazekii_str__Madrid_E_chromosome_complete_genome probe_cstm_Rprow_021218_1x_105383 ACTTTCCTAATGCTCATGATATAACGAAACCTAAAGTTACAGAAATAAGAAATAAAGCGCGCATAAAATAATATTGTATTAGATCAAGCTCAGCATAAAGCATACATAGAGAACATATAC 735480 735600

NC_000963_1_Rickettsia_prowazekii_str__Madrid_E_chromosome_complete_genome probe_cstm_Rprow_021218_1x_105384 TCAGTGATACGACACAAAATTACTGCAAAAATTGCTATAATACCAATTGCCAGTATGAATTTTTAAATTTAGTGATTATAAAAGATAACAAAGCGATATTTATAGTGCATATATACCAAC 735600 735720

NC_000963_1_Rickettsia_prowazekii_str__Madrid_E_chromosome_complete_genome probe_cstm_Rprow_021218_1x_105385 TGAATAATTAAAGCACATATATGTCTAGATACATATCTAGAACAATATACAATGACAGTATATAGAATAGAACATCAGCTTCAGGCAAAAATAACTCATTTAAAAGTACTGCCATGATAT 735720 735840

NC_000963_1_Rickettsia_prowazekii_str__Madrid_E_chromosome_complete_genome probe_cstm_Rprow_021218_1x_105386 ATTAAGAGAATTAGAAAAATATTCTAAACATACCTATTTATAATAGTAAAACTATTTACTATTTTTGATTACAGACTAAACTTTTTCCTTTGTAACTTAAAGTTACTACAAAGAGGCTAC 735840 735960

NC_009488_1_Orientia_tsutsugamushi_Boryong_complete_genome probe_cstm_Rprow_021218_1x_10539 TACAAAACCTATTGAACCACGATTAAAGAATAGCTGAGTCTCCTCATCATACGATTCATAGACAAAGTGTTTAGAAAATCTTTCTCGATCAAAATCCTTATGTATGCTTGCGTTCACCTT 1264560 1264680

NC_009488_1_Orientia_tsutsugamushi_Boryong_complete_genome probe_cstm_Rprow_021218_1x_1054 cactatcagaaatttcatcaaaattctttatttttttttATTATTACCATCAGTTATGTTACTCTGGAATTCTTACTCTATATTTTCTATCAGTAGTGATATTGCTAGCTGCGCATATAG 126360 126480

NC_009488_1_Orientia_tsutsugamushi_Boryong_complete_genome probe_cstm_Rprow_021218_1x_10540 GACTCTGTTTTGATTTAAATAAATTAATAAGATAtttttttggttttttCGGCGACTTGTGAACAACATCTACACCTTGAACTGCTTTAGATCGCTTACAGTATAGAACACAACGCCTTT 1264680 1264800

NC_009488_1_Orientia_tsutsugamushi_Boryong_complete_genome probe_cstm_Rprow_021218_1x_10541 TTGATTTAATTTTTGATGTTTCAACTTTCTCAAGATTATTAACGTCAGTTTAGGATAAGGGAAAGTATGAAAGATATAATGCAAAAGGTTTACAAAGGATAATATTTCGTTATGACTTTT 1264800 1264920

NC_009488_1_Orientia_tsutsugamushi_Boryong_complete_genome probe_cstm_Rprow_021218_1x_10542 AATACATTACATTAAATATAATCTTAATTCAAAACCATTTACTGCTCTAATATTTCTAGTAAATTCGCCTTTGTAATATCAATGTTTAATAACTAGCTATTCTTTATAAGCCTTCTGTAT 1264920 1265040

NC_009488_1_Orientia_tsutsugamushi_Boryong_complete_genome probe_cstm_Rprow_021218_1x_10543 TATGCTTTTCATACTTTTCCTTATCTTGATCTGGCGTTTATAGAACCTAAAAAAGGTGATTTCGTTTATCTTGATCCACCTTACCACCAATCAGGAGAACGGTTCTACACTAGAGTTCAA 1265040 1265160

NC_009488_1_Orientia_tsutsugamushi_Boryong_complete_genome probe_cstm_Rprow_021218_1x_10544 TTTGATGAAAAAGAGCAAATCAGACTACGAGATTTTGTTTATGAACTAAACAATAAAGGTGTTAAAATTATGCTTTCAAATAATAACACTACCTTCATTAAAGATTTATACAAAGATTTT 1265160 1265280

NC_009488_1_Orientia_tsutsugamushi_Boryong_complete_genome probe_cstm_Rprow_021218_1x_10545 TTCATCACTCATATTGGAGTTACATACTCAATCAATGAACATCGTAATCTCGTTAATGAGCTAATCATTACTAACTATAAAACTTATTAGTTATTAAACGCCAGATTAGGATAATGGGAA 1265280 1265400

NC_009488_1_Orientia_tsutsugamushi_Boryong_complete_genome probe_cstm_Rprow_021218_1x_10546 GTATGAAAAGCATAATGAAAAAGGTATACAAGAGATACTGTACAATTATAATTATGCAATATATTTAAATATAATTTCAATTAATGACGGTTTATTTATTGCTCATATTTCTACAAAAAT 1265400 1265520

NC_009488_1_Orientia_tsutsugamushi_Boryong_complete_genome probe_cstm_Rprow_021218_1x_10547 TAGTTCATAATACTATCTTGCTAACCTGAACATTGACCATTGTAACACTTCTATATTATCTCTTTCTAACTGTTTTTTATTCTAAATTTGCATTTACAGACATGTGAGCAATAAATAAAC 1265520 1265640

NC_009488_1_Orientia_tsutsugamushi_Boryong_complete_genome probe_cstm_Rprow_021218_1x_10548 TATTATTAATTAAAACTATATTTAAAGATATTACATAATATCTCTTGTAAACCTTTTGCATTATATCTTTCATACTTTCTCTTATCCTAAATTGGCGTTTAAAGATTCAGTATTACTGAA 1265640 1265760

NC_009488_1_Orientia_tsutsugamushi_Boryong_complete_genome probe_cstm_Rprow_021218_1x_10549 TACTACCTTCATGTCTGCTATTTGTCTTTCTACCTCATTTGGAGAAGTAGTAAATAAGAGTTTCGTCACATAAATTGCCCATTCCTTTAAATAGGTTTCATGGTAATTTTTCGATGAAAC 1265760 1265880

NC_009488_1_Orientia_tsutsugamushi_Boryong_complete_genome probe_cstm_Rprow_021218_1x_1055 TTTGAACTATTATCTAAGACACAAGCTCAAGTTTATACTGAACATTGAGAAGGCCATTCTTTAAGGCCTCAATGTCCTTAACATTGCCGCACCATTCTTGACGAAAACTGTTCCATTTTA 126480 126600

NC_009488_1_Orientia_tsutsugamushi_Boryong_complete_genome probe_cstm_Rprow_021218_1x_10550 CGTCATTTTACGATCAGGCTCTATTGCTGGAATTAACAACCACTTTTCTTCTTTGGTAATTGCAGCCATTATCGCAATTATATTAGCTGCAGCTAGCAATATAGTTACTGAAAGTAAGCA 1265880 1266000

NC_009488_1_Orientia_tsutsugamushi_Boryong_complete_genome probe_cstm_Rprow_021218_1x_10551 TTTATTATATTTAACCAGCTCTTGTATAGCATTTTACTTAAAGAGATGATTCATTACTTGCCAACTTTTTTGCCCAAAAGCCTTGGATATCCTAATGGAGCTGGCAATAAACCTTTAGCT 1266000 1266120

NC_009488_1_Orientia_tsutsugamushi_Boryong_complete_genome probe_cstm_Rprow_021218_1x_10562 TCTCTTTCCAGTTTATAGTATATATTTTGGCATACTCTTCAATAGACTTCTTTCGAAACTATACAATGATGTAAAATGGTGTTATAAAAATCTGATTTGAAGTAATAATGAAATTAGATC 1267320 1267440

NC_000963_1_Rickettsia_prowazekii_str__Madrid_E_chromosome_complete_genome probe_cstm_Rprow_021218_1x_105669 AGTGCTATGTGCAACTACAGCTTTTAAAATATCTAAATTGCCTTTATCTATAGTCTTATTAATAGTATAGAACTGTATATTAGTATTATCATAATTAAGTAAAGAAGTTAATAATTCAAA 769800 769920

NC_000963_1_Rickettsia_prowazekii_str__Madrid_E_chromosome_complete_genome probe_cstm_Rprow_021218_1x_105670 ATTTTCTTGCTCTATCGCTTGATCTACACTAAAATCAGTATTTAATCCTTTCTGATGTAACAGATTCTCTATCTTGCTTAGAGAAAATTTATTAAAATTATATGTTTCTAAAGTTAAAGC 769920 770040

NC_000963_1_Rickettsia_prowazekii_str__Madrid_E_chromosome_complete_genome probe_cstm_Rprow_021218_1x_105671 TCTAGTAAATACTCTTACTAAATCAGCGTATGTTAATCCTTTCGGTTTTGTTCCTATACTAATGTTAAATATTTCTGGATATGATACATGATATTTTGCATAAATATTTTGATAAAACTT 770040 770160

NC_000963_1_Rickettsia_prowazekii_str__Madrid_E_chromosome_complete_genome probe_cstm_Rprow_021218_1x_105672 TACAAAAGTTCCATATTCTTTATATaaaaaaaCAGGTAACTCTTCTATGCTTTTCATGTTTTTAATGTTATCGGAACTTAAAGAAAAATTATAAATTTCATTATCTAATTTATAACTTAT 770160 770280

NC_000963_1_Rickettsia_prowazekii_str__Madrid_E_chromosome_complete_genome probe_cstm_Rprow_021218_1x_105673 GCTAAAATCTGATGCAACTAATAAATGAAAATTATCATGTATATATTCTATCAGGGAAGAGTGtttttttATTCTAGTCTCATAATTACTATTAGCTAATTGTGTAACATTATTATCACT 770280 770400

NC_000963_1_Rickettsia_prowazekii_str__Madrid_E_chromosome_complete_genome probe_cstm_Rprow_021218_1x_105674 GGCTTTATTATAAGTGCATAAAACAATATTCCCCTCTATAAAATCTAAATGATGTTGAGCTGCACCTGAACAACAAGAAAATATATGAACAATATTACATTGATTTGCAGGAGTATTACG 770400 770520

NC_000963_1_Rickettsia_prowazekii_str__Madrid_E_chromosome_complete_genome probe_cstm_Rprow_021218_1x_105675 TTGTATTTTATTAAGCAGTGTAATATCTAACATAGGAGTTGCACCTCCATTAGATGACATAGTTAGCGATAAGTAAGAAGGATCAGGCATAAAATATGACTGAATCTTGCCTAATAAACC 770520 770640

NC_000963_1_Rickettsia_prowazekii_str__Madrid_E_chromosome_complete_genome probe_cstm_Rprow_021218_1x_105676 TAAATGAGAATTTTGATaaaaaaaCTCTGCACCATGTGCATGTATAAAATATTCACAAGCTTTAGCCGAAGGTAAGTGATCTAAGTTTCTAATTTCCTTGATATCAGGATTTGCATTATC 770640 770760

NC_000963_1_Rickettsia_prowazekii_str__Madrid_E_chromosome_complete_genome probe_cstm_Rprow_021218_1x_105677 CTTACTTATATCATAACCTTCAATTAATATTTTCTGCATATTTACATACCTTATAAAATATTATAATTACCATATAATATGGTAATTATTTAAAATGTCAAGAGCAGATTTAGAGCtaat 770760 770880

NC_000963_1_Rickettsia_prowazekii_str__Madrid_E_chromosome_complete_genome probe_cstm_Rprow_021218_1x_105679 TTATATTGAATCAATCTATAATGTAGGGTCATTAAATATATACTTCCTTCATTAGTGCGCATATCAAAATGTTTTCTTGTTTATAACGATTAATAAACTTGATACTTTAAAGCAAAGTAA 771000 771120

NC_000963_1_Rickettsia_prowazekii_str__Madrid_E_chromosome_complete_genome probe_cstm_Rprow_021218_1x_105680 AGCCTTTATTTTTGTAAATGATCCATATTATTAGTTTTTTACATAATCTACATTAAAAACTTGAGGAAAACACAAAAATACTTACATAGTATATATAAATACATGAGACAAGTACAGACG 771120 771240

NC_000963_1_Rickettsia_prowazekii_str__Madrid_E_chromosome_complete_genome probe_cstm_Rprow_021218_1x_105681 CTTGAAATATGAACCATTTTAGTAGAAAGTGATCAAAATAGTTTATTTTTTCAATAACAAATAATATACAATATAAAACCATCCGAAAAACCCATGAATTATAGCCCATAATATTGATTT 771240 771360

NC_000963_1_Rickettsia_prowazekii_str__Madrid_E_chromosome_complete_genome probe_cstm_Rprow_021218_1x_105684 AGCTAAAAAGAAGAAAGAAATGAGTAATAACAGCATATATTTAAATTAGGACATCAGATAAAATTGCTAAAAATTTTTTAAACTTTGAATATATACATGCATAAGTAGAATATATTATAA 771600 771720

NC_000963_1_Rickettsia_prowazekii_str__Madrid_E_chromosome_complete_genome probe_cstm_Rprow_021218_1x_105685 GAATTTATTGAGTAAAATTATTTATTATTTTATCTTGTACAAGAACAATAAATATTACTATCTTCCAGCAAGGATACAAAAATAATAAATAATTCATACATAATTGCACCATACTCTAAG 771720 771840

NC_000963_1_Rickettsia_prowazekii_str__Madrid_E_chromosome_complete_genome probe_cstm_Rprow_021218_1x_105686 ATCTAACTAGTATTTAGTAGCATACAATTTCAGAAAAGAATTTCTGCAAATCAAAATATGATATTTATATATCTTTTTTAAATAAACCGGTGTTATTAAATGAATGCTGCAAAATTATTT 771840 771960

NC_000963_1_Rickettsia_prowazekii_str__Madrid_E_chromosome_complete_genome probe_cstm_Rprow_021218_1x_105687 TTAGCCATAATTTAGCAATTTTTATTATAGATTTTGAGAATGAAGCAAATATAAAATTTTAATAAAAATATGTAAAAATTACTAGATTTTATGAATAAAGTCTTAGCAAGAAGATATGTA 771960 772080

NC_000963_1_Rickettsia_prowazekii_str__Madrid_E_chromosome_complete_genome probe_cstm_Rprow_021218_1x_105688 ATAAGTCTAATGAGTACATAACTCATTACTTTAATACTTTTTCCCCAGAAATGAAATTGATCTTAAGAAGAAAAAAGTGAGTATAGATTTAAAAGTGAACAATTAGTTAAAGATAGTGAC 772080 772200

NC_000963_1_Rickettsia_prowazekii_str__Madrid_E_chromosome_complete_genome probe_cstm_Rprow_021218_1x_105689 AGTAAATATTTGGACTGAGTAATTACTTATATAAATATAAGTTAATATACTAATTTAGTCTTTAAGGAGGTAATCCAGCCGCAGTTCCCCTACGGCTACCTTGTTACGACTTCACCCCAG 772200 772320

NC_009488_1_Orientia_tsutsugamushi_Boryong_complete_genome probe_cstm_Rprow_021218_1x_10569 ATTATTGCTGACAAATATCGAAATAGACGTAAAAGATTCGGTCTTAGATTTAATTTGATCTCTGGCATTTATAATTTTGATCTACCTTAACCAGTTTCGAAAGAGGTCTATTATACtttt 1268160 1268280

NC_000963_1_Rickettsia_prowazekii_str__Madrid_E_chromosome_complete_genome probe_cstm_Rprow_021218_1x_105690 TCGCTAATTTTACCGTGGTTGGCTGCCTCTTGCGTTAGCTCACCACCTTCAGGTAAAACCAACTCCCATGGCGTGACGGGCAGTGTGTACAAGGCCCGAGAACGTATTCACCGCGGCATG 772320 772440

NC_000963_1_Rickettsia_prowazekii_str__Madrid_E_chromosome_complete_genome probe_cstm_Rprow_021218_1x_105691 CTGATCCGCGATTACTAGCGATTCCAACTTCATGCTCTCGAGTTGCAGAGAACAATCCGAACTGAGATGTCTTTTAGGGATTTGCTCCACGTCACCGTATTGCTTCCCTCTGTAAACACC 772440 772560

NC_000963_1_Rickettsia_prowazekii_str__Madrid_E_chromosome_complete_genome probe_cstm_Rprow_021218_1x_105692 ATTGTAGCACGCGTGTAGCCCAACCCGTAAGGGCCATGATGACTTGACGTCGTCCCCACCTTCCTCCGGCTTATCACCGGCAGTTTTCTTATAGTTCCCGGCATTACCCACTGGCAAATA 772560 772680

NC_000963_1_Rickettsia_prowazekii_str__Madrid_E_chromosome_complete_genome probe_cstm_Rprow_021218_1x_105693 AGAATAAGGGTTGCGCTCGTTGCGGGACTTAACCCAACATCTCACGACACGAGCTGACGACAGCCATGCAACACCTGTGTGTGGCCCAGCCGAACTGAAGGAAAGCATCTCTGCAATCCG 772680 772800

NC_000963_1_Rickettsia_prowazekii_str__Madrid_E_chromosome_complete_genome probe_cstm_Rprow_021218_1x_105694 TAACCACCATGTCAAGGGTTGGTAAGGTTTTTCGCGTAACATCGAATTAAACCGCATGCTCCACCGCTTGTGCGAGCCCCCGTCAATTCCTTTGAGTTTTAATCTTGCGACCGTACTCCC 772800 772920

NC_000963_1_Rickettsia_prowazekii_str__Madrid_E_chromosome_complete_genome probe_cstm_Rprow_021218_1x_105695 CAGGCGGAGTGCTTAATGCGTTAGCTGCGAAACCGAAAGAGAATCCTCCGATATCTAGCACTCATCGTTTACGGCGTGGACTACCAGGGTATCTAATCCTGTTTGCTCCCCACGCTTTCG 772920 773040

NC_000963_1_Rickettsia_prowazekii_str__Madrid_E_chromosome_complete_genome probe_cstm_Rprow_021218_1x_105696 TGCATCAGCGTCAGTTGTAGCCCAGATGACCGCCTTCGCCACCGGTGTTCCTCCTAATATCTAAGAATTTCACCTCTACACTAGGAATTCCATCATCCCCTACTACACTCTAGATTAGTA 773040 773160

NC_000963_1_Rickettsia_prowazekii_str__Madrid_E_chromosome_complete_genome probe_cstm_Rprow_021218_1x_105697 GTTTTGAAAGCAATTCCGAGGTTAAGCCCCGGGCTTTCACTTCCAACTTACTAAACCGCCTACGCACTCTTTACGCCCAGTAATTCCGAACAACGCTAGCCCCCTCCGTCTTACCGCGGC 773160 773280

NC_000963_1_Rickettsia_prowazekii_str__Madrid_E_chromosome_complete_genome probe_cstm_Rprow_021218_1x_105698 TGCTGGCACGGAGTTAGCCGGGGCTTTTTCTGCAAGTAACGTCATTATCTTCCTTGCTAAAAGAGCTTTACAACCCTAAGGCCTTCATCACTCACTCGGTATTGCTGGATCAGGCTTTCG 773280 773400

NC_000963_1_Rickettsia_prowazekii_str__Madrid_E_chromosome_complete_genome probe_cstm_Rprow_021218_1x_105699 CCCATTGTCCAATATTCCCCACTGCTGCCTCCCGTAGGAGTCTGGGCCGTGTCTCAGTCCCAGTGTGGCTGATCATCCTCTCAGACCAGCTACAGATCGTCGGCTTGGTGAGCCATTACC 773400 773520

NC_009488_1_Orientia_tsutsugamushi_Boryong_complete_genome probe_cstm_Rprow_021218_1x_10570 tttGATATCAAATTTTTTATACTAATTTGTTCATAATACATATAACTTGAAAATCACTTATCAATCCTAGATTGTATATATTTTTCGATCATTAGATTAACCTCTAGATTGAAAACTGGC 1268280 1268400

NC_000963_1_Rickettsia_prowazekii_str__Madrid_E_chromosome_complete_genome probe_cstm_Rprow_021218_1x_105700 TCACCAACTACCTAATCTGACGCGGGCCCATCCATCAGCGATAAATCTTTCCTCCGTAGAGAATATACGGTATTAGCTTTTATTTCTAAAAGTTATTCCGTACTGATGGGTAGATTCCCA 773520 773640

NC_000963_1_Rickettsia_prowazekii_str__Madrid_E_chromosome_complete_genome probe_cstm_Rprow_021218_1x_105701 CGTGTTACTCACCCGTCTGCCACTAATTAACTAAAGCGAGCTCTAGTTAATCCGTTCGACTTGCATGTGTTAAGCATACCGATAGCGTTCGTTCTGAGCCAGGATCAAACTCTCAAGTTT 773640 773760

NC_000963_1_Rickettsia_prowazekii_str__Madrid_E_chromosome_complete_genome probe_cstm_Rprow_021218_1x_105702 GATTCTGTCAGTTTTATTTTCAAAATTGACAGGTAAATTATCACTTTTGTGATGTGTACATTTTTACTGTCACTATCTATATCTAATTATTTACTTTTAAAAACAGCTGCGACTTTCGCT 773760 773880

NC_000963_1_Rickettsia_prowazekii_str__Madrid_E_chromosome_complete_genome probe_cstm_Rprow_021218_1x_105703 TTGTATAGTTGCAGTTATATGCAATATAATCAGACCTGTCAACTAAAATTTCGAGATTATTTTAtttttttATTAAGAATATGTTTATGCTCTTATTTTAATTGATCACTAAAAATTAAT 773880 774000

NC_000963_1_Rickettsia_prowazekii_str__Madrid_E_chromosome_complete_genome probe_cstm_Rprow_021218_1x_105704 AAGATTTTTGGTGTCTCCaaaaaaaaTGGAATTAAAAAATTGGATACGATTGAAAATTTAGCTTACTTAGCTTTAAAATTAAGATCGTAAACAAACTATTAATCTTAGTTATTGATTTAG 774000 774120

NC_000963_1_Rickettsia_prowazekii_str__Madrid_E_chromosome_complete_genome probe_cstm_Rprow_021218_1x_105705 TAAATCCTTATATAGTTATAATACTACTCAATGAGTAGTACTAGGATCATCAAATTATTTCAAGCATAATTCAAGATACTGAGTTAAAATAAATCCACATTATTGCTGCTTATTAATTTT 774120 774240

NC_000963_1_Rickettsia_prowazekii_str__Madrid_E_chromosome_complete_genome probe_cstm_Rprow_021218_1x_105706 TTCTATCATTTATTCTGATCCAGCTTTCAATAACTTTGAATCTTGTGCTTTCCGCATGACAAGTAAAAAGTGACATACCTTAATTTAAAATATTTAGTACAATTATTCCGCTGTTGTAGT 774240 774360

NC_000963_1_Rickettsia_prowazekii_str__Madrid_E_chromosome_complete_genome probe_cstm_Rprow_021218_1x_105707 TAGTGAGCTCTGCAATATCTCGCAAGCACAAAAACTTTTGCATAAAAATTATAATGCGAGTACAATATATAATGACATACATAATAACTTTTAGGATCAGCAATTATTTAATTAGTACAA 774360 774480

NC_000963_1_Rickettsia_prowazekii_str__Madrid_E_chromosome_complete_genome probe_cstm_Rprow_021218_1x_105708 TTAGCAAATTGTATTTCAAAGACATTCCTCGTAACAGATACTTAAAAACTTTTTTCTAAGTCCTTATTACATCATAAAATTAATACTAAGAAGTGAAGAAATACAATTTAAATGTAATTA 774480 774600

NC_000963_1_Rickettsia_prowazekii_str__Madrid_E_chromosome_complete_genome probe_cstm_Rprow_021218_1x_105709 GTAATTTTGATTTTCATATCATCGCTATAAAGCTAATGATTTAGTACTCACTACTCTTTCGAAATAAAAATATACTGAATGATCATAGCCTTACATATCATTAAATTTAATGCATTTTCT 774600 774720

NC_000963_1_Rickettsia_prowazekii_str__Madrid_E_chromosome_complete_genome probe_cstm_Rprow_021218_1x_105710 GATTAAATAATACTGTACTCCTCTGTATCTGTATGAtttttttATACAATTTATGTTAGATAAGATAATCTATAAGATGGGGAAATTAATCAGTTAGTTGTAAATAAAAAACCTTTGCAA 774720 774840

NC_000963_1_Rickettsia_prowazekii_str__Madrid_E_chromosome_complete_genome probe_cstm_Rprow_021218_1x_105711 AACATAATTAACTAAAGACATTTTAAAGTTGTGTCATATATGCACATGATGGTAAAAATTTATGTATTATAAATTGAGATTGTAAAAAATATTGATATCAATAATCTTAGTTATACTGCA 774840 774960

NC_000963_1_Rickettsia_prowazekii_str__Madrid_E_chromosome_complete_genome probe_cstm_Rprow_021218_1x_105712 TAATAGCTATTATCAGTTCTTTGGAACTATAAGTAAATTTTCTGAGAACATCGGTAATACTCTAGAAAATAGCGTGGGGTCAAGTTAGTATTATCACTGACAAAAATCTAATGATCTTTT 774960 775080

NC_000963_1_Rickettsia_prowazekii_str__Madrid_E_chromosome_complete_genome probe_cstm_Rprow_021218_1x_105713 AGTGTGTGATTATAATCATTGTTACATTAATTAAATAAACTGAAATGCACATAATGATTTAAAGAATAAGTATTGTACCATCATATGATATAGACTGCACAGTAAATTATTGGGTTCTAT 775080 775200

NC_000963_1_Rickettsia_prowazekii_str__Madrid_E_chromosome_complete_genome probe_cstm_Rprow_021218_1x_105714 TACTTTATTAACCAAGATCTAACCACCAGAACGTCTAACCTTCAAATGCAGCTCTTTTAAATCAAATTTAATATCGATTATTGCACCTTCATCTTCTCTGTTTGCAATATCTAAAACACC 775200 775320

NC_000963_1_Rickettsia_prowazekii_str__Madrid_E_chromosome_complete_genome probe_cstm_Rprow_021218_1x_105715 ACAATGCTCTTCTACTATTCTTTTGACTATAGCAAGCCCTACACCCATACCTTTACTACTGGTTGTTACATAGCTTTCGGTAGCTTTGCCTATGAGCTCAGGTGGAAATCCTTTACCGTT 775320 775440

NC_000963_1_Rickettsia_prowazekii_str__Madrid_E_chromosome_complete_genome probe_cstm_Rprow_021218_1x_105716 ATCCATTACAATAACACTGATAAAATCATCTTTAGCATCTAAAATAACATCTATTTTTCCAAATTCTTGTCCTTCTATTGACTCTTCGGCATTTTTTAACACATTAATCATCACTTGATT 775440 775560

NC_000963_1_Rickettsia_prowazekii_str__Madrid_E_chromosome_complete_genome probe_cstm_Rprow_021218_1x_105717 TATTTGTGTAGCATCACACATAAAATCAAATTGATCTACATTAGAATCGTATGTATACACAATATTATCATTAAGTAATTTACGCGCTTCAATAATATGTTTTACTAAATACACTAATTC 775560 775680

NC_000963_1_Rickettsia_prowazekii_str__Madrid_E_chromosome_complete_genome probe_cstm_Rprow_021218_1x_105718 ACTCTTTGTAAATTTAGGAGCAGGAAGACGTGCAAACAGTACAAATTCGGATACTATATTTTTAATATCGTTAGTATGACGAATAATCATTTTTAAATAACTTTCAAATTCTGACTTCTC 775680 775800

NC_000963_1_Rickettsia_prowazekii_str__Madrid_E_chromosome_complete_genome probe_cstm_Rprow_021218_1x_105719 ATTAATTTCAGAACTAAATTTCTTAAGTAACCTTTCAGAAGCAAGTAAAATAGGAGTTAATGGGTTCTTGATTTCATGCGCTACtttttttGCAACATCAGACCAAGCCATAGCTCTCTG 775800 775920

NC_009488_1_Orientia_tsutsugamushi_Boryong_complete_genome probe_cstm_Rprow_021218_1x_10572 ATAAAAGTTAACACAAGTTAATAGTAGGTTCTATAAAGTGTAGAAGTTAATAAAAATTAACTTTTCTTCTTCCTTATATTTGATCTACCTTTGCACTTTTACCTATAGAACATCTTATTG 1268520 1268640

NC_000963_1_Rickettsia_prowazekii_str__Madrid_E_chromosome_complete_genome probe_cstm_Rprow_021218_1x_105720 TGCTATAACTAAATCACGCTGCTGACGCGAAAGCTGCTTAATCATTCTATTAAATGCCGCATAAAGCGTTCCTATTTCGTCTTTATCTACCTCATTTTCAGGCACCTGCACTGTTAAATC 775920 776040

NC_000963_1_Rickettsia_prowazekii_str__Madrid_E_chromosome_complete_genome probe_cstm_Rprow_021218_1x_105721 ACCATCTTTAACTTTGTCGGTTGCAGTGACTAATTTTTTAATCGGTTTTACTATTTTAGCAGTAAATAAAACACCAAAATTTATAGCTACAAAAAGGAGTAATAATGCAATAAAGATAAA 776040 776160

NC_000963_1_Rickettsia_prowazekii_str__Madrid_E_chromosome_complete_genome probe_cstm_Rprow_021218_1x_105722 CATTATAGAAAACTTAATCTGTATATTATCTATTTCATTTTTAAGGCTATTATATTCGGCAGCTGCTCCGTTAGTTGCATCTATATGATCGATAATTTTATTATCTACTAGTCTACCGAC 776160 776280

NC_000963_1_Rickettsia_prowazekii_str__Madrid_E_chromosome_complete_genome probe_cstm_Rprow_021218_1x_105723 TAATAAATACACATCATTATATTCTTTTAATTTAATTAACATCCTGATTGTAGTTGGGTCAGATTTTACTTCCACCGGTTCCCCTAAATCAGCTTTTTTAATTAAGTGTGCTGGAATAGT 776280 776400

NC_000963_1_Rickettsia_prowazekii_str__Madrid_E_chromosome_complete_genome probe_cstm_Rprow_021218_1x_105724 TGCAAATGATAATGAAAAACTTAAATAACTATTAGCTACTATAGTATTAGTGGATTTATTTAAAACTATCGCTTCATCAAGCGAACGCATATCTGCTTCGGTATTAAGTGTTTTAGTAAA 776400 776520

NC_000963_1_Rickettsia_prowazekii_str__Madrid_E_chromosome_complete_genome probe_cstm_Rprow_021218_1x_105725 TAAAGCAGGATTATGAATTAGATCGTAATACATATCGCTTAAATCTTCAGCAACTGCTAAAGCTGTTTCTTTGAGCTGTACTTTATGCTCAGCAATATAAGTTTCAGCTACTATTACAGA 776520 776640

NC_000963_1_Rickettsia_prowazekii_str__Madrid_E_chromosome_complete_genome probe_cstm_Rprow_021218_1x_105726 TTGATCAAGCACTGTAGAAATCTTCTTATCAAACCATGCCTTAACACTAAGATTaaaaaaaTAAACAGAAAATACAGAAACAATTATAGTGGGAATAGCAGCGACTAAACTAAATGCAAT 776640 776760

NC_000963_1_Rickettsia_prowazekii_str__Madrid_E_chromosome_complete_genome probe_cstm_Rprow_021218_1x_105728 cgtactaaaatTCTTTGCTTCTAAAGATATCACATAAAAAGTAGTACAAACTGAAATAATAGATGCTATTGCTAAAGCAAGAATTAGTATTCTACTAGAAAAATAAGAGCGCAAATTTTG 776880 777000

NC_000963_1_Rickettsia_prowazekii_str__Madrid_E_chromosome_complete_genome probe_cstm_Rprow_021218_1x_105729 TTTTAAATCACTAAGCATAGACATATTGCTATAACAGTTTCTTATAACAGAAAAAACTCATTGGAATAATGCCTACTTTATCATTCATACAGAAACAATGATCCAAAAAATGATATAAAT 777000 777120

NC_000963_1_Rickettsia_prowazekii_str__Madrid_E_chromosome_complete_genome probe_cstm_Rprow_021218_1x_105730 AAAGCTCTTAGAAAACTTTAGGGGATGACATCAAATTATAGGACAACTATAGAAAATTACCAATATAATCTATATAACACTAAAGCTAGAAATATTTCTAGCTTTAATTTAGTATTAAAA 777120 777240

NC_000963_1_Rickettsia_prowazekii_str__Madrid_E_chromosome_complete_genome probe_cstm_Rprow_021218_1x_105731 TCTTTTTGGTTTTACTCTTCATACATTTTCTTTCGAGCAAATTTTCTTATTCTTCTTGCTGCTTCTTGTGCTTTACGAACACGCTTCGCTGACGGTGTTTCATAATAACGCTGTTCTTTC 777240 777360

NC_000963_1_Rickettsia_prowazekii_str__Madrid_E_chromosome_complete_genome probe_cstm_Rprow_021218_1x_105732 ATCTTACGAAAATAAAGCTCTCTTTGTAAtttttttttAAAATTCTTAAGCGTATTGTCACAATTTCCTGCGTGAACATTGACTAGTATCACTAAAATTACTCTCCTAAAAAGATATTGT 777360 777480

NC_000963_1_Rickettsia_prowazekii_str__Madrid_E_chromosome_complete_genome probe_cstm_Rprow_021218_1x_105733 GGTTTATTGTACTATAACTTATAATTAACTTATATGTCAAGGTTAAAATATGAGTATCAAGTATGAGCATCAAGGAAGAATACAATATaaaaaaaaTAAACTTTGTGCAATCTTTACTAG 777480 777600

NC_000963_1_Rickettsia_prowazekii_str__Madrid_E_chromosome_complete_genome probe_cstm_Rprow_021218_1x_105734 AATTATTACCGTTTAATGAATGGAATAATAATCTACTTGAAGAAGCAGAAGAGAAATGTAGTTTTGAAAAGGGTTATGCTTTAATAATTTTCCCGGACGGATTATCTGAAATAATAGAAT 777600 777720

NC_000963_1_Rickettsia_prowazekii_str__Madrid_E_chromosome_complete_genome probe_cstm_Rprow_021218_1x_105736 TTATTCATAGTAAGAATGCTTCTTATTTTGCATTAAACCCAATACAAGGAATTAAAGTTGCTGTACGTAGCTGTGATGCTATATGGCGTTATGCCGGCGACAAATCTCTTGATTTTAATT 777840 777960

NC_000963_1_Rickettsia_prowazekii_str__Madrid_E_chromosome_complete_genome probe_cstm_Rprow_021218_1x_105737 ATTATACTAAAAGAGGACTATTACTATCAGTTTATATTTCCTCAATTCTTTTTTATATCCAAGATGAATCAGAACATTATATTGAAACTGACCAATTTATTGAGACCTCTataaaaaata 777960 778080

NC_000963_1_Rickettsia_prowazekii_str__Madrid_E_chromosome_complete_genome probe_cstm_Rprow_021218_1x_105738 tagtaaaaacttctgtccaaataaaaaaaaTACTTGACACTTCAAATATCCCAATAATTAGAATGTTTACGTAGAAAAGTTCTATGAACATTAATTAGATAATAAAACATTAACTCTTCT 778080 778200

NC_000963_1_Rickettsia_prowazekii_str__Madrid_E_chromosome_complete_genome probe_cstm_Rprow_021218_1x_105739 TACTCTTCATAAAATATCATTATTAAAACAATACTATTATCAATCAGGATCATTTATTGATGCATTTTTAAAATATCAAAAACAAAATCAAGAAATTAAGTATTATACGTATGAGATATA 778200 778320

NC_009488_1_Orientia_tsutsugamushi_Boryong_complete_genome probe_cstm_Rprow_021218_1x_10574 CTAGAATTTGCAATGGGAAATATCAGGCTAAACCTGCACGAATAACGGAAATTCCAAAAGAAGATGGAGTCAAAAGACCTTTGATAATATCATGTTTTTAAGATAAGATAATCGAGTCTA 1268760 1268880

NC_000963_1_Rickettsia_prowazekii_str__Madrid_E_chromosome_complete_genome probe_cstm_Rprow_021218_1x_105741 ACAAAAATACGCAACACAAGCAAAAATAACAAATATTAACTTTTAATTTAACTTATTACAAATTCATGTGTTCAATATTTGAATATATTTTTCTACAAAAATTATCTTCAAAAATATCAA 778440 778560

NC_000963_1_Rickettsia_prowazekii_str__Madrid_E_chromosome_complete_genome probe_cstm_Rprow_021218_1x_105742 TTTCGTATTACTGAAAAAGAAAAATTGTTATTTTCTTTTAAATATACCTTATACAAAAATATTACAACTTTAGCATTAGAAAGTTCTTGATTGTGCATTGCACTCTTGAAATTTATTAGT 778560 778680

NC_000963_1_Rickettsia_prowazekii_str__Madrid_E_chromosome_complete_genome probe_cstm_Rprow_021218_1x_105743 TTAGTATAAATTATGTATTTATCTCTTAGTCATTATCTATTATATTAAACCAAATTATAATAATTTCTAGAATAATGACAAATACTAAATATTACCCAGACGTTAGCGCTAATGTGGATT 778680 778800

NC_000963_1_Rickettsia_prowazekii_str__Madrid_E_chromosome_complete_genome probe_cstm_Rprow_021218_1x_105744 TCGCTGCTATAGAGCAAGAGATACTCAAATTTTGGCAAAATAATAATATATTCCAAAAATCTATTGATTATAGGAATGGAGAATCAGAATTTATTTTTTATGACGGACCACCTTTTGCAA 778800 778920

NC_000963_1_Rickettsia_prowazekii_str__Madrid_E_chromosome_complete_genome probe_cstm_Rprow_021218_1x_105745 ACGGTTTGCCACATTATGGTCATTTGCTTACTGGCTTTATTAAAGACGTATACGCTAGATACAAAACTATAAAAGGTAAGAAAGTTGAGCGTCGTTTTGGCTGGGATTGTCATGGTTTAC 778920 779040

NC_000963_1_Rickettsia_prowazekii_str__Madrid_E_chromosome_complete_genome probe_cstm_Rprow_021218_1x_105746 CTGCTGAAATGCAATCAGAGAAAGAACTTGGTATTTCAGGACGTATTGCAATCACCAACTTTGGTATTGAAAAATTCAATAATCACTGTAGAGCTTCTGTCATGCAATATGCCAGTGAGT 779040 779160

NC_000963_1_Rickettsia_prowazekii_str__Madrid_E_chromosome_complete_genome probe_cstm_Rprow_021218_1x_105747 GGGAGCAATACGTCACACGTCAAGCAAGATGGGTAGCTTTTAAAAACGCTTATAAAACGATGGATAAAAATTTTATGGAATCCGTTTTATGGGCATTCAAAGAACTATATAATAAAGACT 779160 779280

NC_000963_1_Rickettsia_prowazekii_str__Madrid_E_chromosome_complete_genome probe_cstm_Rprow_021218_1x_105748 TATTATATGAGTCAATGCGTGTAATGCCATATTCTTGGGCATGCGAAACACCACTTTCTAATTTTGAAACAAGGCTTGATAATGCATATCGTGAACGTACGGATAAAGCAATAACAGTGA 779280 779400

NC_000963_1_Rickettsia_prowazekii_str__Madrid_E_chromosome_complete_genome probe_cstm_Rprow_021218_1x_105749 GTTTTGTGCTAAATGAGGTTACTCTTATCAACGGCATTATATCGCAGAAGTCAGATATGAAAGAAGGCGATAATTTTAAAGAATATAGAATTCTTGCTTGGACGACGACTCCTTGGACAC 779400 779520

NC_009488_1_Orientia_tsutsugamushi_Boryong_complete_genome probe_cstm_Rprow_021218_1x_10575 CAGTAAGCAAGATACTAAACTCTGTGTTTGAGCCAATATTCTTAAAGTATTCCTATGGATTTCGACCTAAATTAAATGCACACGATGCTTTAAGGAAGTTAAATAGACTTACGTATAACT 1268880 1269000

NC_000963_1_Rickettsia_prowazekii_str__Madrid_E_chromosome_complete_genome probe_cstm_Rprow_021218_1x_105750 TTCCGGCAAATTTAGCACTTGCAGTTGGTAGTGATATTGATTATGCATTCGTCGATAAAAATGAGGTTTGTTATATTATAGCTGCCTCATCAGTCGCTAAATATGCAAAAGAATTAGGGC 779520 779640

NC_000963_1_Rickettsia_prowazekii_str__Madrid_E_chromosome_complete_genome probe_cstm_Rprow_021218_1x_105751 TTAGTGGTAAAGAAAATTTTGAGATAATTAAAGGTTTAAAACTTCAAGGATTAAGCTATAAACCTTTATTCAACTATTTTGAGAATCATCCAAATAGCTTTAAGATATTTGCTAGCGACT 779640 779760

NC_000963_1_Rickettsia_prowazekii_str__Madrid_E_chromosome_complete_genome probe_cstm_Rprow_021218_1x_105752 TTGTTGTTGAAGGCGACGGAACAGGAATAGTACATATGGCTCCAGGCTTTGGTGAAGATGACCAAATACTTTGTGAATCGAAAGGTATTGAACTTGTTTGCCCTGTTGATAATAGTGGTA 779760 779880

NC_000963_1_Rickettsia_prowazekii_str__Madrid_E_chromosome_complete_genome probe_cstm_Rprow_021218_1x_105753 AGTTTACAAAAGAGATTCCTGATTTAGAGGGAGTACAAGTATTCGATGCAAATGATAAAATAATAATAAAACTGAAAGAACAAGGAAATTGGATAAAAACTGAGCAGTATATTCATAATT 779880 780000

NC_000963_1_Rickettsia_prowazekii_str__Madrid_E_chromosome_complete_genome probe_cstm_Rprow_021218_1x_105754 ATCCTCATTGCTGGCGCACAGATACGCCTCTTATATATAAAGCTGTTCCATCATGGTATGTAAGGGTTACGAAGTTTAAAGATAGAATGGTTGAATTAAATCAGCAAATAAATTGGATTC 780000 780120

NC_000963_1_Rickettsia_prowazekii_str__Madrid_E_chromosome_complete_genome probe_cstm_Rprow_021218_1x_105755 CACATAACGTTAAAGATAATTTATTTGGTAAGTGGCTTGAAAATGCTCGTGATTGGTCAATAAGCCGTAATAGATTCTGGGGCACCCCATTACCAGTTTGGAAATCCGATGATCCAAAAT 780120 780240

NC_000963_1_Rickettsia_prowazekii_str__Madrid_E_chromosome_complete_genome probe_cstm_Rprow_021218_1x_105756 ATCCACGCATAGATGTTTACGGTTCTATAGAAGAGATTGAGAAAGATTTTGGTGTTAAAATTAATGATTTGCATCGTCCATTTATCGATGAACTTACTAGAACAAATCCCGATGATCCAA 780240 780360

NC_000963_1_Rickettsia_prowazekii_str__Madrid_E_chromosome_complete_genome probe_cstm_Rprow_021218_1x_105757 CAGGTAAGTCGACTATGCGTAGAATAGATGATGTCTTTGATTGCTGGTTTGAAAGCGGCTCCATGCCATATGGACAGGTACATTACCCTTTTGAGAATAAAAAATGGTTTGTAGAGCATT 780360 780480

NC_000963_1_Rickettsia_prowazekii_str__Madrid_E_chromosome_complete_genome probe_cstm_Rprow_021218_1x_105758 TTCCTGCTGATTTTATAGTTGAATACTCATCTCAAACACGTGGTTGGTTTTATACTTTAATGGTGTTATCTACAGCTTTATTTGATCGTCCACCATTTTTAAATTGTATATGCCACGGCG 780480 780600

NC_000963_1_Rickettsia_prowazekii_str__Madrid_E_chromosome_complete_genome probe_cstm_Rprow_021218_1x_105759 TAATTTTAGACGCTACAGGTCAAAAATTATCAAAACGTCTAAACAATTATGCCGATCCGCTGGAGCTATTTGATAAATACGGATCAGACGCATTAAGAGTTACAATGCTTTCCTCAAATG 780600 780720

NC_000963_1_Rickettsia_prowazekii_str__Madrid_E_chromosome_complete_genome probe_cstm_Rprow_021218_1x_105760 TTGTTAAGGGTCAAGAACTGTTAATAGACAAAGACGGTAAGATGGTATTTGATACTCTTCGTCTATTTATAAAACCCATATGGAATGCATACCATTTCTTTACAATATATGCTAACGCCG 780720 780840

NC_000963_1_Rickettsia_prowazekii_str__Madrid_E_chromosome_complete_genome probe_cstm_Rprow_021218_1x_105761 ATTCGCTTAAAGGCACACTTAACTTTGCTTCACAAAACGTACTTGATGTTTATATATTATCTAAGCTTAAAATAGCAGTAAATAAAATTGAAGAAAGTTTAGATAATTTCGATACACAAA 780840 780960

NC_000963_1_Rickettsia_prowazekii_str__Madrid_E_chromosome_complete_genome probe_cstm_Rprow_021218_1x_105762 CGGCTTATCATGCAGTTTCAGAGTTTTTTGAAGTATTAAATAACTGGTATATAAGGCGCAGCCGAGCTAGATTTTGGAAAAACGAAAAAGATACAGATAAGCAAAATGCTTATAATACCC 780960 781080

NC_000963_1_Rickettsia_prowazekii_str__Madrid_E_chromosome_complete_genome probe_cstm_Rprow_021218_1x_105763 TATATTCATGTTTAAAGATCATGACTATTGCGATGTCGGCACTAATACCTATGATTTCAGAAACTATATATCAGGGATTACATAATACTGCCATCACACAACTTAATTGCTTGCTTTCAG 781080 781200

NC_000963_1_Rickettsia_prowazekii_str__Madrid_E_chromosome_complete_genome probe_cstm_Rprow_021218_1x_105764 AAGGTAAACATATAGTACAAAATCCAATGTCTGATACACAGGATTACAACACGTCAGTACATCTCTGCAACTACCCAACGCTCTCAGATTTTGAAATAAATTACGAACTTGTGAGCACTA 781200 781320

NC_000963_1_Rickettsia_prowazekii_str__Madrid_E_chromosome_complete_genome probe_cstm_Rprow_021218_1x_105765 TGGATAACGTCCTTGATATTTGCAGTAATAGCCTATTTATTAGGAGCACTGAAAATATCAGAGTAAGGCAGCCACTTGCTTGCATAACTATTATTAGTAAACATAATAATAATCTCAAAG 781320 781440

NC_000963_1_Rickettsia_prowazekii_str__Madrid_E_chromosome_complete_genome probe_cstm_Rprow_021218_1x_105766 ATTTTGAAGATTTAATTAAAGATGAAATCAATGTTAAAACGGTAATATACCGTGATGATCTAGAGAATTATGCACGCAAAAAATTATCTCTTAATTTCGCAATACTTGGTAAACGTCTAC 781440 781560

NC_000963_1_Rickettsia_prowazekii_str__Madrid_E_chromosome_complete_genome probe_cstm_Rprow_021218_1x_105767 CACATAAAATGAAAGCAATTATAGATGCTGCTAAAAAAGGGGAATGGGAAGCTACTACTCTTGGTTTAGCTATATGCGGGGAAATCTTAAATAGTGACGAATATACGCTAATTTTAGAAC 781560 781680

NC_000963_1_Rickettsia_prowazekii_str__Madrid_E_chromosome_complete_genome probe_cstm_Rprow_021218_1x_105768 CATATTCACATATTAAAGGAACAGCAAATTTTGATAATAACAGTAGCTTACTCATACTTAATTTAGAGCTAACATCTGAGTTAATAGAAGAAGGTTACGCTAGAGACATTGTACGATTTA 781680 781800

NC_000963_1_Rickettsia_prowazekii_str__Madrid_E_chromosome_complete_genome probe_cstm_Rprow_021218_1x_105769 TACAATATGCTCGAAAAGAAGCTGATTTCTCTATAACTGACAGGATTTTAATTGAGATAATAAGCGAGTTTGATTTATCTAAGATCATTGATCATTATGGAGACTTTATTAAAGAACAAA 781800 781920

NC_000963_1_Rickettsia_prowazekii_str__Madrid_E_chromosome_complete_genome probe_cstm_Rprow_021218_1x_105770 CTTTAGGCGAGTTTGCTAAAAATTTTACACCTGATTATGTCAGTAAAGTAGCATTAGAGAACAATCAAATACAACTTAAAGTTAAGAGATTATAAATTGTTCATATTTTATTCAATAGAA 781920 782040

NC_000963_1_Rickettsia_prowazekii_str__Madrid_E_chromosome_complete_genome probe_cstm_Rprow_021218_1x_105771 ATTAATACAAAAATACTAAAAAGGAGCAAATATACATTTTTTGAGTCACTAAACATTGCTTCTTCCTCTTTATACAAACATAataaaaaagaaaatgagcggtccataaaaatataaacg 782040 782160

NC_000963_1_Rickettsia_prowazekii_str__Madrid_E_chromosome_complete_genome probe_cstm_Rprow_021218_1x_105773 AGAAGCGTATaaaaaaaTCAATAAATTTGTTATCCTTATGCTTTGTGTAATTTAATATTATATCAAAGTACTTTATTTAATTGCTGTAGTTCTTTTTACAATCTGGACAAATATCTAGAA 782280 782400

NC_000963_1_Rickettsia_prowazekii_str__Madrid_E_chromosome_complete_genome probe_cstm_Rprow_021218_1x_105774 GTTATAATGTTGATATATAAACTTTTATTATTATCAAAAAAGCATAATATTATTTTGATATTTCTAAAACTGTCTAATAAGACCAATAAATCATTGTTTTATATATTGAGTATTAAATTC 782400 782520

NC_000963_1_Rickettsia_prowazekii_str__Madrid_E_chromosome_complete_genome probe_cstm_Rprow_021218_1x_105775 AACTTAATAGAATAATATTCTCGAAGCACAAACAAGATTTATTTATATTGAACGATACTTACTGTAGTATTTAGTATGTTATGAGAAAATTTTGACTTAAATAGATAATAGATACTAGTT 782520 782640

NC_000963_1_Rickettsia_prowazekii_str__Madrid_E_chromosome_complete_genome probe_cstm_Rprow_021218_1x_105776 AATTTAAAAATTAAAATACACTTTACAATTTTAAAATTAGACGAATATATCAAGCAAATTCTAATAATATTTTCCCTCTTACAACATTATCTTTTTCATTAACAAAAATTTTAGCTATTT 782640 782760

NC_000963_1_Rickettsia_prowazekii_str__Madrid_E_chromosome_complete_genome probe_cstm_Rprow_021218_1x_105777 TTCCGTCTCTTTCAGCTAAAATAAGATTTTCCATTTTCATAGCAGTTAAAATCATAATTTCCTGTCCTATAGTCACCTCTTGCCCTTCTTTCACTTTTATTGCAGCAATCTGACCACTTA 782760 782880

NC_000963_1_Rickettsia_prowazekii_str__Madrid_E_chromosome_complete_genome probe_cstm_Rprow_021218_1x_105778 AAGGAGCTTGTAGTTCAGAATTTTCTTCTAACACTACTTTTGAAACCATCAACGCTTCAAGTTCTGATATACGCGGAGAGCGTACAAAAGCTTTTACACTAATACCGGCATGCGATAATA 782880 783000

NC_000963_1_Rickettsia_prowazekii_str__Madrid_E_chromosome_complete_genome probe_cstm_Rprow_021218_1x_105779 AATAACCAGTTCTAATATTTTCAATTTTTACATTTGTCTTCTTACCGTTAATCATAGCAGTAAAAAGTTCATTACCTAAATTCCAATTACTACGAATATATATTCTATCACTTTCGTGAC 783000 783120

NC_000963_1_Rickettsia_prowazekii_str__Madrid_E_chromosome_complete_genome probe_cstm_Rprow_021218_1x_105780 GAATATTATAGCCATTTTCAACAGGTGTAATTAATACTGGGAATAGTTTATCATCAATAGTTACTACCCATCTTGTACCTATTTTGTTAGCCTGATTATTAATATTACCAGAAATTAATG 783120 783240

NC_000963_1_Rickettsia_prowazekii_str__Madrid_E_chromosome_complete_genome probe_cstm_Rprow_021218_1x_105781 AAGCGCGTCTTTGTTCCGATATATAAATAAAAATAGCAGTTGCTAGAAATACGGTAGTTACTTCTGAAGTAAGACTAGCACCAGAAAAACCATCAGGATATTCTTCTTGAATAAAGGCAG 783240 783360

NC_000963_1_Rickettsia_prowazekii_str__Madrid_E_chromosome_complete_genome probe_cstm_Rprow_021218_1x_105782 TAGAAATATCACCGCTAACAAAGCGTGGATGTAGCATTACTGCTTCTAAGAAACTAATATTATGAGCGATGCCATTAATTATATAAGAACTTAAAGCAGAACGCATTACCTCAATTGCCT 783360 783480

NC_000963_1_Rickettsia_prowazekii_str__Madrid_E_chromosome_complete_genome probe_cstm_Rprow_021218_1x_105783 GCTCCCGTGTTTCACCGTAAGTACATAATTTTGCAATCATTGAGTCATAAAACATACTTACTTCACCACCAAGCCCAATCCCTGTATCAATACGGATATTAGGACTCTTTGCAGGTTCAG 783480 783600

NC_000963_1_Rickettsia_prowazekii_str__Madrid_E_chromosome_complete_genome probe_cstm_Rprow_021218_1x_105784 AATAAGCTATAATTCTACCACTAGAAGGTAAAAACCCTCGACTTGGGTTTTCAGCACAAATTCTTGATTCAAATGCCCAACCTTTTAATTTTATGTCATCCTGGGTAAATGATAACTTCT 783600 783720

NC_000963_1_Rickettsia_prowazekii_str__Madrid_E_chromosome_complete_genome probe_cstm_Rprow_021218_1x_105785 CACCTGCTGCAATTTTTATCATTTCTTCAACGATATCTATACCGGTAATTAACTCAGTAACAGGATGTTCTACTTGCAATCTAGTATTCATTTCTAAAAAATAGAAATTTTTATTGCTGT 783720 783840

NC_000963_1_Rickettsia_prowazekii_str__Madrid_E_chromosome_complete_genome probe_cstm_Rprow_021218_1x_105786 CAACTATAAATTCAACGGTACCAGCCGAATAATATCCAACTTTTTGAGACAAGGATATTACTTGTCGATACATTTCATGCCGTATATTTTCAGTAATAAAAGAACTTGGTGCTTCTTCAA 783840 783960

NC_000963_1_Rickettsia_prowazekii_str__Madrid_E_chromosome_complete_genome probe_cstm_Rprow_021218_1x_105787 TTACTTTTTGATGGTGACGCTGTATTGAACATTCACGTTCTCCGAGACATACACTATTGCCATATTGATCCGCAATTAGCTGAATCTCAATATGACGCGGGGTTTGAATTAACTTCTCAA 783960 784080

NC_000963_1_Rickettsia_prowazekii_str__Madrid_E_chromosome_complete_genome probe_cstm_Rprow_021218_1x_105788 TAAATAATCTATCATCACTAAAACTATTAGCAGCTTCAAGCTTTGCTGATTCAAAAGCATTTGCCATTTCAGCTGGATTATTCACCACTCTCATACCACGTCCACCACCACCGGCAGTTG 784080 784200

NC_000963_1_Rickettsia_prowazekii_str__Madrid_E_chromosome_complete_genome probe_cstm_Rprow_021218_1x_105789 CTTTTACTATTACTGGAAAACCAATTTCTTTAGCAATATCTATAGCTTGTTTAACATCTTTTATAGTACCCATATAACCTGGAACAGTACTCACTCCAGCCTCTATTGCAATTTTCTTTG 784200 784320

NC_009488_1_Orientia_tsutsugamushi_Boryong_complete_genome probe_cstm_Rprow_021218_1x_10579 TTTTGCCTAAAAGGTTAAATAAGTATGGGCTAAATATCAATGAAGCTAAATCACAAATGATTAAATCTGGTAGAGACCATGCTGCAAATTTAGCCAAACAAGACAAGAAGATCTCAAGTT 1269360 1269480

NC_000963_1_Rickettsia_prowazekii_str__Madrid_E_chromosome_complete_genome probe_cstm_Rprow_021218_1x_105790 CTTCGATTTTATCACCCATTTTCTTAATAGTTCCAGCGCTTGGCCCTATTAAAACTACTCCTTCTCTTTTAAGAATATTAGCAAAATTTGGATTTTCCGATAAAAAACCGTATCCGGGGT 784320 784440

NC_000963_1_Rickettsia_prowazekii_str__Madrid_E_chromosome_complete_genome probe_cstm_Rprow_021218_1x_105791 GTACAGCACTTGCTCCACTCTCACGAATCGCTGAGATAAGGTTTTTAACAGATAAATAGCTTGCAGTAGCAGGAGAATCACCTATATAATAAGCCTCGTCTGCATGCTGTACATACATTG 784440 784560

NC_000963_1_Rickettsia_prowazekii_str__Madrid_E_chromosome_complete_genome probe_cstm_Rprow_021218_1x_105792 AATTAGTATCAGCTTCAGAATATACAGCAACTGACCCTATGCCCATTTTCTTGAGAGTTCGAATTATCCTAACTGCAATTTCACTACGGTTAGCAATTAAAATTTTATCAAATAATGGTT 784560 784680

NC_000963_1_Rickettsia_prowazekii_str__Madrid_E_chromosome_complete_genome probe_cstm_Rprow_021218_1x_105793 TTGTCATAATTTTTTGTATCCTAATACCACGCTATATCTAACCACTAGATCTTTTACAGACGATCTTCTTATGTTTCGTTACGGAGCATATTATGGCGACTAAAGCGGCAAATTATCATG 784680 784800

NC_000963_1_Rickettsia_prowazekii_str__Madrid_E_chromosome_complete_genome probe_cstm_Rprow_021218_1x_105794 TTTCTTCCAAGGTAACGATACTTTTTTAGTACGTAAAAAATTTAACGCCTTACATATTCTCCATCTAGTATTTTGTGGTCTTATAATATCATCTAAATATCCTCTAGATGCTGCAACAAA 784800 784920

NC_000963_1_Rickettsia_prowazekii_str__Madrid_E_chromosome_complete_genome probe_cstm_Rprow_021218_1x_105795 AGGTGATGTGACTACTTTTCTATATTCATCGAttttttgctttttAGCTTCATTGTCTTTACATTCTTCCTTAAATATTATCTCAGCCGCACCCTCTGCACCCATAACAGCAATTTCAGA 784920 785040

NC_000963_1_Rickettsia_prowazekii_str__Madrid_E_chromosome_complete_genome probe_cstm_Rprow_021218_1x_105796 ATTAAACCAAGCATAGTTTATATCACCTCTAAGATGCTTTGAATTCATCACTATATAAGCACCTCCATAAGCCTTGCGTGTAATCACTGTAATCTTCGGCACAGTTGCTTCTGCATAAGC 785040 785160

NC_000963_1_Rickettsia_prowazekii_str__Madrid_E_chromosome_complete_genome probe_cstm_Rprow_021218_1x_105797 GTATAACAGCTTAGCCCCGTGTTTAATAATACCTTCATGTTCTTGAGATGTACCAGGCAAAAATCCAGGAACGTCAACTAGGCTTACTATGGAAATATTAAAAGCATCACAAAATCTGAT 785160 785280

NC_000963_1_Rickettsia_prowazekii_str__Madrid_E_chromosome_complete_genome probe_cstm_Rprow_021218_1x_105798 AAATCTAGCTGCTTTTCTTGAAGCATTTATATCCAGACATCCTGCTAAATGCAATGGTTGATTAGCGATAAATCCTACAGGATAACCTTCCATATAACCAAAACCGATAATAATATTCTT 785280 785400

NC_000963_1_Rickettsia_prowazekii_str__Madrid_E_chromosome_complete_genome probe_cstm_Rprow_021218_1x_105799 AGCAAAATCAGGCTGTAATTCAAAAAATTCACCTTCATCAACAATACGTTCAACAAGCTCTTTCATATCGTAAGGCTTATTAGGAGTGTTAGGTATTAAAGTACTCAGAGACATATCTAC 785400 785520

NC_000963_1_Rickettsia_prowazekii_str__Madrid_E_chromosome_complete_genome probe_cstm_Rprow_021218_1x_105800 TCTATCAGCAGGATCAACAGTCGGCCGGACAGGCAGAGTGCTACGATTAGATGAAGGTAGAAAATTAAAGAATTTACGAATTTCGAGTAATGCTTCTATATCATTATTAAATGCAAGGTC 785520 785640

NC_000963_1_Rickettsia_prowazekii_str__Madrid_E_chromosome_complete_genome probe_cstm_Rprow_021218_1x_105801 GGCAACACCACTCTTAGTAGTATGCATTCTAGCTCCACCAAGCTTTTCTTGAGTTACTTCTTCACCTGTAACAGTTTTTACTACATCAGGACCGGTTACAAACATATAAGAACTATTCTT 785640 785760

NC_000963_1_Rickettsia_prowazekii_str__Madrid_E_chromosome_complete_genome probe_cstm_Rprow_021218_1x_105802 AACCATGAATATAAAATCGGTTAATGCAGGAGAATAAACAGCACCACCAGCACAAGGTCCCATAATTAAGGTAATTTGTGGAATAACACCAGAAGCTAAAACATTACGCTGGAATAATTC 785760 785880

NC_000963_1_Rickettsia_prowazekii_str__Madrid_E_chromosome_complete_genome probe_cstm_Rprow_021218_1x_105803 ACCATATCCGGCAAGAGCATCAACACCTTCTTGAATTCTTGCGCCACCTGAGTCGTTAATACCGATGACTGGTGCACCAGTTGCTATAGCTTGGTCGATAATATCACAGAtttttttAGC 785880 786000

NC_000963_1_Rickettsia_prowazekii_str__Madrid_E_chromosome_complete_genome probe_cstm_Rprow_021218_1x_105804 ATGATATTCACCAAGAGAGCCTCCTAGTACAGTAAAATCTTGGCTATAAATAAATACCAACCTACCATTTATTGTACCGTATCCAGTTACAACACCATCACCTAAAAACTTTTTATTCTG 786000 786120

NC_000963_1_Rickettsia_prowazekii_str__Madrid_E_chromosome_complete_genome probe_cstm_Rprow_021218_1x_105805 CATCCCAAAATTATCGCATCTATGCGATACAAACATTCCTGTTTCAGTAAAACTATTTGGATCTAATAATACTTCTATGCGTTCACGTGCAGTTAACTTACCTTTTTGATGTTGTGCGTT 786120 786240

NC_000963_1_Rickettsia_prowazekii_str__Madrid_E_chromosome_complete_genome probe_cstm_Rprow_021218_1x_105806 GATCCTAGCTTCACCGCCCCCTTGCCTTGCAATATTTTTTCTCTCATCTAATAACTCAGGCGAGATTATATTACTTTGATTCATAGCGAGATTAATAGTTTTATTTTTCAAAATACCaat 786240 786360

NC_000963_1_Rickettsia_prowazekii_str__Madrid_E_chromosome_complete_genome probe_cstm_Rprow_021218_1x_105807 aatataatataataataataaAGTAAAAAGATAATTATTATGTCATCTACAGAAGATACAAAAGAAGTACTCAAAAAAGTCAAATCACTTGCTAAATTTGCTAATTTATCAACTCTCTTa 786360 786480

NC_000963_1_Rickettsia_prowazekii_str__Madrid_E_chromosome_complete_genome probe_cstm_Rprow_021218_1x_105810 atctaaatttgacatattaatataaattGATAATATTTGTTGGGTTTAGGCTGCATTACGAGTCCTATGTCATCTCTGTATAGTATTATCTACTTGGATCAGTTTTACGTCTGTCATTCT 786720 786840

NC_000963_1_Rickettsia_prowazekii_str__Madrid_E_chromosome_complete_genome probe_cstm_Rprow_021218_1x_105811 GTAATTTATTCACGGAATCTCGTTAAAAACACTAATTATTAGTATTTAGTTGCGTTCGTGATATAGTGGACAATCCACAGCACAACGCCGCGTATTTTTGAACTATGTTTTGAAATACCA 786840 786960

NC_000963_1_Rickettsia_prowazekii_str__Madrid_E_chromosome_complete_genome probe_cstm_Rprow_021218_1x_105812 AGACAGAACGATAAAATCCAATAAAATTGGCTTTTTAAATTATTGAATATCATGGACACAAACAAATTATATCTTTTTAAAGATAGACGATTTTTACCAAATTTTATAGTACAGTTATTC 786960 787080

NC_000963_1_Rickettsia_prowazekii_str__Madrid_E_chromosome_complete_genome probe_cstm_Rprow_021218_1x_105813 GGTTGTCTTAATGATAATATATTAAAAAATGCTCTCGTAATACTTATAACTTATGGCATTTCAGGAACTCTAAGTAAATATAATAATGTACTGGTTTTAATTACTAATGCTACTTTTGTA 787080 787200

NC_000963_1_Rickettsia_prowazekii_str__Madrid_E_chromosome_complete_genome probe_cstm_Rprow_021218_1x_105814 CTACCTTTCATAATATTCGCAAGCATAGCAGGACAAATTGCTGATAAATACGAACGTGCTAATCTTATGAAAATTCTTAAGATTTATGAAATCGGTATAATTGCCTTTGCAATTTACGGA 787200 787320

NC_000963_1_Rickettsia_prowazekii_str__Madrid_E_chromosome_complete_genome probe_cstm_Rprow_021218_1x_105815 TTTCATCATAATAATCTGTTAATCCTGTTCTGTTCTATTGGCTTAATGGGTATACATTCTACtttttttGGTCCAATCAAATATAGCGTATTACCTGATCACCTAAATAAAGATGAATTA 787320 787440

NC_000963_1_Rickettsia_prowazekii_str__Madrid_E_chromosome_complete_genome probe_cstm_Rprow_021218_1x_105816 CTTGGAGCTAATGGATTTGTTGAAGCTGGTACTTTTATAGGTATTTTCATTGGTACTATAATAGGAAGTTATTATACAATCAGCAATAATTTTATAATTTATTCATTAATTATTATTGCT 787440 787560

NC_000963_1_Rickettsia_prowazekii_str__Madrid_E_chromosome_complete_genome probe_cstm_Rprow_021218_1x_105817 TTTCTTGGCTTTATTACAAGCCTTTTTGCTCCAAAATCGGACAATGCAAATCATAATATTAAAATAAATTTTAATATTATAGATGAAAGTATAAGTATGATTAAATATTCTAAAGCaaaa 787560 787680

NC_000963_1_Rickettsia_prowazekii_str__Madrid_E_chromosome_complete_genome probe_cstm_Rprow_021218_1x_105818 aaaCAGATATATTTAGCTATACTCGGTATTTCATGGTTTTGGTTTATTGGCGCTGCGATCATTTCACAAATACCTTTGCTTGCTAAGATAACTTTTAAGGCTGATGAGAATGTGGCTAAC 787680 787800

NC_000963_1_Rickettsia_prowazekii_str__Madrid_E_chromosome_complete_genome probe_cstm_Rprow_021218_1x_105819 TTATTTTTAGCTGTTTTTTCTCTTGGTGTTGGAGTAGGATCATTTCTCTGTAGCAAAATATTTGAAAATGAGATTACGGTTAAATATCTATTTATTTCAGCTCTAGGTATTAGTATTTTC 787800 787920

NC_000963_1_Rickettsia_prowazekii_str__Madrid_E_chromosome_complete_genome probe_cstm_Rprow_021218_1x_105820 GGTATTGATTTATTCTTCGCAAGTAGAATTAGTTCAGTTAACTACGAACCTACTCAGCTAAAAAGTATTTTTGTATTTTTATCTAAAAGACATAATTGGCGAATAGTTATAGATTTATTT 787920 788040

NC_000963_1_Rickettsia_prowazekii_str__Madrid_E_chromosome_complete_genome probe_cstm_Rprow_021218_1x_105821 TTCTTAGCAATAATAGGCGGGTTATATATCGTTCCACTTTTTGCAATATTACAGCATTATGCGAATCCTGCTCATCGGAGCAGAATTATTGCAGCTAATAACCTCATTAATTCCATTTTC 788040 788160

NC_000963_1_Rickettsia_prowazekii_str__Madrid_E_chromosome_complete_genome probe_cstm_Rprow_021218_1x_105823 CCTGAAGTTAAAATTATTCCTTTTAAGCTATTACGTAGAATATTTCAAATCTGTTTTGATCTTATGTATAAAGTTGAAGTTAAAGGATTTGAAAATTTTCAAAAAGCTGGCAAAAAAGTA 788280 788400

NC_000963_1_Rickettsia_prowazekii_str__Madrid_E_chromosome_complete_genome probe_cstm_Rprow_021218_1x_105824 GTTGTTGTTGCTAATCATATTTCATATCTCGATCCACCGCTAATTGCTACTTACTTACGAGAAGAGATGATTTTTGCTATTAGTCCAGATATACAAAAAATATGGTGGATTAAACCATTT 788400 788520

NC_000963_1_Rickettsia_prowazekii_str__Madrid_E_chromosome_complete_genome probe_cstm_Rprow_021218_1x_105825 TTGCATATGGCTAAAACTTTGCCAGTTGATCCAAGTAATCCAATGGCTATCAAGACTTTAGTAAAAGAAGTACAAAAAGATCAGAAAATAGCTATTTTCCCGGAAGGTAGAATAAGTGTT 788520 788640

NC_000963_1_Rickettsia_prowazekii_str__Madrid_E_chromosome_complete_genome probe_cstm_Rprow_021218_1x_105826 ACTGGTTCTTTAATGAAAATCTACGAAGGACCTGGCATGATTGCCGATAAAGCCGATGCAACACTCTTACCTGTAAGAATAGACGGTACTCAATTTACACATCTATCaaaattaaaaaat 788640 788760

NC_000963_1_Rickettsia_prowazekii_str__Madrid_E_chromosome_complete_genome probe_cstm_Rprow_021218_1x_105827 atattaaaaaagaaaatatttcctaaaattACTATAACAGTGTTACCACCAGTAAAATTCGCTAATATGGATACTGCAAGTAATCAAGAACGACGTAGTTATATATCTAGGACTCTTTAT 788760 788880

NC_000963_1_Rickettsia_prowazekii_str__Madrid_E_chromosome_complete_genome probe_cstm_Rprow_021218_1x_105828 GATATTATGGCTGACATGATGTTTGAGAGTTCAGATTATAAAAATACTTTGTTCTCATCTCTTATAGAAGCTGCTAAAATTCATGGATTTaaaaaaaaaaTAGTTGATGATTTTGaaaaa 788880 789000

NC_000963_1_Rickettsia_prowazekii_str__Madrid_E_chromosome_complete_genome probe_cstm_Rprow_021218_1x_105829 aaTACAGTAACTTATCGCGATTTAATATTCAAATCTTTTATCCTAGGTGACTTAATCAAAAAGAATAATATCTTTGGAAGGAATTTAGGCCTAATGTTGCCGAATACTACAAATACATTA 789000 789120

NC_000963_1_Rickettsia_prowazekii_str__Madrid_E_chromosome_complete_genome probe_cstm_Rprow_021218_1x_105830 ATTACTTTTTATGCTATGCAATCTAGTGGTTATGTTCCTGCTATAATTAACTGGAGTAGCGCTATAGGTACTATTATTAACTGCTGTAAACTTGCACAAATTAAAGTAGTTTATACGTCA 789120 789240

NC_000963_1_Rickettsia_prowazekii_str__Madrid_E_chromosome_complete_genome probe_cstm_Rprow_021218_1x_105831 AAAGAATTTATTGAGAAAGCAAATTTACATAAATTAATAACTAACTTGTTAGATTTCGGAATTAAAATAATATATTTAGAAGATCTTAAAAATCAAATTAGTACAGCTCTAAAACTCAAA 789240 789360

NC_000963_1_Rickettsia_prowazekii_str__Madrid_E_chromosome_complete_genome probe_cstm_Rprow_021218_1x_105832 GCCAAAATAGGAGGTTATTTTACTCAAACTTATTACAATTATTTTTGTCATAATCGTGATGAAGAAAAACCGGCAGTAATAATTTTTACTTCTGGCACTGAAGGAGAACCTAAAGCAGTA 789360 789480

NC_000963_1_Rickettsia_prowazekii_str__Madrid_E_chromosome_complete_genome probe_cstm_Rprow_021218_1x_105833 TTACTATCTCACAGAAATTTACAAACTAATAGATATCAAATAACCGCTAAAGTACCTTTTAGCCCTGAAGATATAGTATTTAATGCACTACCACTATTTCATTGTTTTGGGCTTAGTGGA 789480 789600

NC_000963_1_Rickettsia_prowazekii_str__Madrid_E_chromosome_complete_genome probe_cstm_Rprow_021218_1x_105834 GCAATTATTACAACTTTAAACGGCATTAAACTATTTTTATATCCTCATGCATTAAATTATCGCAGTATTCCTGAAGCTATATACGATATTGGAGCAACTATATTAATTTCTACCGATACT 789600 789720

NC_000963_1_Rickettsia_prowazekii_str__Madrid_E_chromosome_complete_genome probe_cstm_Rprow_021218_1x_105835 TTTTTAAAGGGTTATGCTAATTACGCGCACCCATATGAttttttttCATTACGCTATATATTTGCTGGTAACGAGAAATTAAAAGAAACTACAAGACAATTTTGGCTTAATAAATACGGT 789720 789840

NC_000963_1_Rickettsia_prowazekii_str__Madrid_E_chromosome_complete_genome probe_cstm_Rprow_021218_1x_105836 ATACGTATTTTTGAAGGATATGGAATTACTGAAGCTGCACCTATTATAGCGTGTAATACCCCTATGCATAATAAAGCAGGCACTGTCGGAAGATTATTACCAAAAATCAATTATAAACTT 789840 789960

NC_000963_1_Rickettsia_prowazekii_str__Madrid_E_chromosome_complete_genome probe_cstm_Rprow_021218_1x_105837 GAAAAAGTAGCAGGTATAAATGAAGGGGGGCGTTTATTAATCAAAGGCCCTAACATCATGCTTGGTTACCTAGACTTAGAAGGGCAACGTAGTCATAAAGAATGGTACGATACAGGAGAT 789960 790080

NC_000963_1_Rickettsia_prowazekii_str__Madrid_E_chromosome_complete_genome probe_cstm_Rprow_021218_1x_105838 ATAGTCAAAATCGATTCTGAAGGATATATAACAATTTTAGGACGCTTAAAACGCTTTGCTAAAATAGCAGAGGAAATGATATCTCTTACAAGAATTGAAGAATTTGCAAGCGAAATCGAT 790080 790200

NC_000963_1_Rickettsia_prowazekii_str__Madrid_E_chromosome_complete_genome probe_cstm_Rprow_021218_1x_105839 CCTGATTCATTGCACGCTGCTATTTCTGTGCAAGATAAAAGACATGGGGAAAAAATTATTTTACTTACTACAGGTTCCGATATAAATCAAGAAAACTTTACAAATATGCTATCTAACGCT 790200 790320

NC_009488_1_Orientia_tsutsugamushi_Boryong_complete_genome probe_cstm_Rprow_021218_1x_10584 CTACTCACATAGATTTAATTTGATCTCTGGCATTTATAATTTTGAACTACATTAACCAGTTTCGAACGAGGTCTATTCTAGCAATCGATAGACATTATCATAAGTTGGAGACTACATGAG 1269960 1270080

NC_000963_1_Rickettsia_prowazekii_str__Madrid_E_chromosome_complete_genome probe_cstm_Rprow_021218_1x_105840 CAAATTTCTTTATTACATTTACCAAAATTAATCATTACCAATTCAGAAATACCACTGCTTGCAAGTGGAAAAATTGATTATATTGAGATTATGAAAAATATAAACCTACGCGATTTCACG 790320 790440

NC_000963_1_Rickettsia_prowazekii_str__Madrid_E_chromosome_complete_genome probe_cstm_Rprow_021218_1x_105841 CATTCTGAGTGTTGTGACCTTGATAGGTAAAACCAAAAACTATTGCACCTCATATCTTTCTAAATATAGTATTTTTCACTAGATAAATTAGTTAAGTTACGATATTGCCTTTATTTGTGT 790440 790560

NC_000963_1_Rickettsia_prowazekii_str__Madrid_E_chromosome_complete_genome probe_cstm_Rprow_021218_1x_105842 TGATTACAGTATTTAAAGCTATTTGAAATTACTTACCTAATAACTCTTGATACTCAATAATTTGCAATAATATTTTCGCATCATGATAAAGTTACTTTAGAAGCGAATCAAAACTTAAAT 790560 790680

NC_000963_1_Rickettsia_prowazekii_str__Madrid_E_chromosome_complete_genome probe_cstm_Rprow_021218_1x_105843 ATATAAATATTGATAACTACTTAGATGATTTAATATAAGTATTCCTACCATAATGCTAATATATAGCATACCAGTTAAATATTACACGAATATTAATGCACCATATTGGTTTAGCAATTT 790680 790800

NC_000963_1_Rickettsia_prowazekii_str__Madrid_E_chromosome_complete_genome probe_cstm_Rprow_021218_1x_105844 TTTGTTATATTAGTCAACTCATAAGAATCGCTTTAAATACAACACACTATAAATCAATGTACCTAATATAATAAACAATGGAGTTAACGGTAAATCAAAGTAGAAAGAGCTAAATGCAGC 790800 790920

NC_000963_1_Rickettsia_prowazekii_str__Madrid_E_chromosome_complete_genome probe_cstm_Rprow_021218_1x_105845 ACAAAAATTAATAAATAAAGAGATAAAAATTGATATTATAATCATCTGAGACGGGCTATATGATACAAACCTGGCTATCATCGCAGGAATAAGTAAAATAGCAGTAACCATAAGTACTCC 790920 791040

NC_000963_1_Rickettsia_prowazekii_str__Madrid_E_chromosome_complete_genome probe_cstm_Rprow_021218_1x_105846 AACAATCTTTATAGCAGCAAATACAGATAATGAGAGAAGAAGTAAAAATATTAGCTCAATAATATTAACCTTTAAGCCTTGTATAACTGCAATATCTCTATTGATAATTATAAGAAGAAT 791040 791160

NC_000963_1_Rickettsia_prowazekii_str__Madrid_E_chromosome_complete_genome probe_cstm_Rprow_021218_1x_105847 TTGATTATAAAAATAGACTACAAAACTTATTATAGTTATAAGCACTACCACGAGTATAATCAAATCATTAAAAGATACGGATAAAATATCACCAAATAACAAATGCACTATATTATTCTG 791160 791280

NC_000963_1_Rickettsia_prowazekii_str__Madrid_E_chromosome_complete_genome probe_cstm_Rprow_021218_1x_105848 CAAAGAAGTAAAATAATTAATAATCAAAGCGACAGCTAGCATAAAGCTAGAAATTAAATTAATAACTGCATTTTTCTCAGAATTATTTTTaaaaaaaaacacgaaaaatgaaaaaaGAAT 791280 791400

NC_000963_1_Rickettsia_prowazekii_str__Madrid_E_chromosome_complete_genome probe_cstm_Rprow_021218_1x_105849 TGCAACTATTATTCCTGAATATATTAATGGGAAATGGGCTATAATGCTTATGCTAGCCGCTAAAAAACTACTATGAGCAAGTCCATCACCAAAATAAATATATCGCTTCCATAGTGCTAT 791400 791520

NC_009488_1_Orientia_tsutsugamushi_Boryong_complete_genome probe_cstm_Rprow_021218_1x_10585 CATCAGAATCAGAGTTTATAGTATTGCAATACTATTATTGCTATAAGCTACAGTATCGCTATCTTGGAATATCGAAGACGTATTTCAAGGAATGAGTGTTAATGTTACTAGAGCTGGATC 1270080 1270200

NC_000963_1_Rickettsia_prowazekii_str__Madrid_E_chromosome_complete_genome probe_cstm_Rprow_021218_1x_105850 ACATCCTAGTGGGGCAAATATACAGCTAATTAAAATTAAGGCTAGTATTATTAAAGTCATCCGAGTATATTATATATAGTGTGTTGCGTCATCAAGTTTAAGACACGTGATGACATTAAA 791520 791640

NC_000963_1_Rickettsia_prowazekii_str__Madrid_E_chromosome_complete_genome probe_cstm_Rprow_021218_1x_105851 AAGAATCATCAACTATGTCTTCAATCAATAAAAAAGAATTAGAAAAATTTGAGAAAATTTCTCATAATTGGTGGAATAAGAATGGGGAATTCGGTATATTACACCGCATAAACCATATTC 791640 791760

NC_000963_1_Rickettsia_prowazekii_str__Madrid_E_chromosome_complete_genome probe_cstm_Rprow_021218_1x_105852 GCATTGAATATATCATAGAAAAAATAAAATCAAATTACAATGATATTTCTAAATTACAAATATTAGATGTCGGTTGTGGTGGTGGATTAATTGCAGCGCCTCTAGCATTGCAAGGTTTTA 791760 791880

NC_000963_1_Rickettsia_prowazekii_str__Madrid_E_chromosome_complete_genome probe_cstm_Rprow_021218_1x_105853 ACGTTACGGCCATTGATGCACTAAAAAGTAATGTTGAAACGGCAACTATTTATGCTCAGAAAAATGGCTTAAAGATAAATTATTTACAAGCTACTATAGAAGAATTAGAAAACGATAAGC 791880 792000

NC_000963_1_Rickettsia_prowazekii_str__Madrid_E_chromosome_complete_genome probe_cstm_Rprow_021218_1x_105854 TATATGATGTAGTAATTTGTCTTGAGGTTATTGAACATGTAGCAAATATACAGCAATTTATACTAAATTTGGTTCAACATATTAAACCAAATGGTATAGCAATAATTTCTACTATGAACC 792000 792120

NC_000963_1_Rickettsia_prowazekii_str__Madrid_E_chromosome_complete_genome probe_cstm_Rprow_021218_1x_105855 GCACTAAAAAAGCTTATTTATTTGGCATAATAGTTGCTGAATATATTTTAGGTTGGGTGCCAAAAAATACTCATGATTATAGCAAATTTGTAAAACCATCGGAAATTTATGAAATACTTA 792120 792240

NC_000963_1_Rickettsia_prowazekii_str__Madrid_E_chromosome_complete_genome probe_cstm_Rprow_021218_1x_105856 CGGATACTAATATTGAAATTAAAGAGCTGAAAGGTTTAGTATTTAATCTTGCTAAGAATGAATGGAAATTAAGTAATGATATAGATGTAAATTATTTTATGTGTTTGGAAAAGAAAATGA 792240 792360

NC_000963_1_Rickettsia_prowazekii_str__Madrid_E_chromosome_complete_genome probe_cstm_Rprow_021218_1x_105857 ATTCACTTTCCAGTACATGTAATGGCATTCCACAACTTAAGCGTGTGATTTAGTAAAATCTCAACAGTATATATTGCAAATTATTTTCCTAGATACTCTAATCaaattataatttaaaaa 792360 792480

NC_000963_1_Rickettsia_prowazekii_str__Madrid_E_chromosome_complete_genome probe_cstm_Rprow_021218_1x_105859 TTTTTAATTATTTGTTTGCAAGACATAATAATGGCAAGTTTTTTCTTCGAATTGAAGACACTGATAAGAAACGGTCAACTAAAGAGGCAGTTGAAGCAATATTCTCAGGTCTAAAATGGC 792600 792720

NC_009488_1_Orientia_tsutsugamushi_Boryong_complete_genome probe_cstm_Rprow_021218_1x_10586 ATATCAAGATCAGGCTGCTGGATATTATGCAGCTGGTGGATTGTCTGCTAGAACAAGCCAAACATCCTGTCAGCCATTTGCTATAATAAACATCAGCTGTAGCGGTATTGACGCATATCT 1270200 1270320

NC_000963_1_Rickettsia_prowazekii_str__Madrid_E_chromosome_complete_genome probe_cstm_Rprow_021218_1x_105860 TGGGGCTGAATTGGGACGGTGAAGTTATATTCCAGTCTAAACGTAATTCGCTTTATAAAGAGGCAGCACTAAAATTACTAAAAGAAGGTAAAGCATATTATTGTTTTACTAGACAAGAAG 792720 792840

NC_000963_1_Rickettsia_prowazekii_str__Madrid_E_chromosome_complete_genome probe_cstm_Rprow_021218_1x_105861 AAATAGCAAAACAACGACAACAAGCTTTAAAAGATAAACAACATTTTATTTTTAATAGCGAATGGCGTGATAAAGGACCATCTACCTACCCTGCTGATATAAAGCCGGTAATACGATTAA 792840 792960

NC_000963_1_Rickettsia_prowazekii_str__Madrid_E_chromosome_complete_genome probe_cstm_Rprow_021218_1x_105862 AAGTACCTCGTGAAGGCAGTATAACAATACATGATACTTTACAAGGTGAGATAGTAATCGAAAACTCACATATAGACGATATGATACTAATTAGAACAGATGGAACTGCTACTTACATGC 792960 793080

NC_000963_1_Rickettsia_prowazekii_str__Madrid_E_chromosome_complete_genome probe_cstm_Rprow_021218_1x_105863 TAGCCGTTATAGTAGATGATCATGATATGGGAATAACGCATATTATTAGGGGTGATGATCATTTAACCAATGCAGCAAGGCAAATCGCTATTTATCATGCTTTCGGTTATGAAGTCCCAA 793080 793200

NC_000963_1_Rickettsia_prowazekii_str__Madrid_E_chromosome_complete_genome probe_cstm_Rprow_021218_1x_105864 ATATGACTCATATACCGTTAATTCATGGCGCAGATGGAACAAAATTATCCAAAAGACACGGAGCTTTAGGCGTTGAAGCTTATAAAGATATGGGATATTTACCAGAAAGTTTATGTAATT 793200 793320

NC_000963_1_Rickettsia_prowazekii_str__Madrid_E_chromosome_complete_genome probe_cstm_Rprow_021218_1x_105865 ATTTATTGCGTCTCGGATGGAGTCACGGTGATGATGAAATTATATCAATGAATCAAGCTATAGAATGGTTTAATCTTGCTTCACTTGGTAAATCACCTTCTAAACTTGATTTTGCTAAGA 793320 793440

NC_000963_1_Rickettsia_prowazekii_str__Madrid_E_chromosome_complete_genome probe_cstm_Rprow_021218_1x_105866 TGAATAGTATTAACTCTCATTACTTAAGAATGCTGGACAATGATAGCTTAACTTCAAAAACTGTAGAGATTCTGAAACAAAATTATAAGATTAGTGAAAAAGAGGTAAGTTATATAAAAC 793440 793560

NC_000963_1_Rickettsia_prowazekii_str__Madrid_E_chromosome_complete_genome probe_cstm_Rprow_021218_1x_105867 AAGCAATGCCAAGCTTAATAGTGAGAAGCGAAACATTGAGAGATTTAGCACAGCTTGCTTACATTTATCTCGTAGATTCACCTATGATCTACAGTCAAGATGCGAAAGAAGTAATAAACA 793560 793680

NC_000963_1_Rickettsia_prowazekii_str__Madrid_E_chromosome_complete_genome probe_cstm_Rprow_021218_1x_105868 ATTGCGATAAAGATTTAATCAAACAAGTTATAGAAAACTTAAGCAAACTTGAGCAATTTAATAAGGAATGCGTACAGAATAAATTTAAAGAAATAGCAATTTACAATGGCTTAAAACTAA 793680 793800

NC_009488_1_Orientia_tsutsugamushi_Boryong_complete_genome probe_cstm_Rprow_021218_1x_10587 TGGTAGCTTCTCTGTTATTTCTGGAGAAGAATTAGTTCAACTAATGAAGAATATTGGTTCTGAGGCTAAAGTTTATGCATTTTCATTAAGATTAAAAACCTTTGCTCCTCAAATTGAGAA 1270320 1270440

NC_000963_1_Rickettsia_prowazekii_str__Madrid_E_chromosome_complete_genome probe_cstm_Rprow_021218_1x_105870 ataataatctgaatttttgaatttttcaaaaaaaTCTAAATTTTTCATTACTGCAACAAAAATACTGAGCTTTTATTTTAATATGAGTAACACAACTCTGCTTATTATAACAAAACACTA 793920 794040

NC_000963_1_Rickettsia_prowazekii_str__Madrid_E_chromosome_complete_genome probe_cstm_Rprow_021218_1x_105871 GGCAATATATTTCAATTTCAATGTGGAGCAAAAAATACAAGGATTAAAGATAATCGTAATAATTCACTAAAATGTTCAAATTCGCAATAGATACAGTTTAGCAGTTGTAAAACAAAAATA 794040 794160

NC_000963_1_Rickettsia_prowazekii_str__Madrid_E_chromosome_complete_genome probe_cstm_Rprow_021218_1x_105872 CATAAATTGTCTCAAACTTTATAACTATACAAAAATATCTAAACATGATTTTATCTATAATTTTAGAACGAATAACAAAAGAGAGAGCAAAAGCTCAAATCTAATTTAGAAAATATAGTA 794160 794280

NC_000963_1_Rickettsia_prowazekii_str__Madrid_E_chromosome_complete_genome probe_cstm_Rprow_021218_1x_105874 ATAATGTTTTTAGGGCCAACACAATATTCATTTATTGCTTGGTTATTTTCGACTATTTTTAGTTTATAATTCAAAAATGTTGAGATTGCACATAATATGTTCATTTTATAAATTTGTTCT 794400 794520

NC_000963_1_Rickettsia_prowazekii_str__Madrid_E_chromosome_complete_genome probe_cstm_Rprow_021218_1x_105875 ATACTTAATTGGGACACATCTCATTCATTTACTCTATAAGCGCATCTTTAACTTTCATCAGGATTTAGATGAGCTGTACATCTGCATCTACAAAAAGTTTGTACTTAAACCTCAACCAAA 794520 794640

NC_000963_1_Rickettsia_prowazekii_str__Madrid_E_chromosome_complete_genome probe_cstm_Rprow_021218_1x_105876 ATTTCATGAACATACATTAAGAAGTAATGTGGTAATACATAATAATTTTAAAAGAAAGATTAGTACAATGAAACCAGTAGCATATTTTATAGATAAAGTAGAAGAGATGTACTTGTTTAA 794640 794760

NC_000963_1_Rickettsia_prowazekii_str__Madrid_E_chromosome_complete_genome probe_cstm_Rprow_021218_1x_105877 CTTTGTTTTATTAGTTATTACTAAAATATGTAATTTAAAGATTCATTGTGAACAAAAAACTACATACAAAATCAAAATCTATTAAAGTTATTTGGTAATTATTTTTCAATTATTTCTATA 794760 794880

NC_000963_1_Rickettsia_prowazekii_str__Madrid_E_chromosome_complete_genome probe_cstm_Rprow_021218_1x_105878 ATAGATCACTACAATATTGGTATTAACAATGTTACGTATTAATACTTTTAATAACGCATCATTATGCCACTACAAATCGCAAGTATTAGCAAAGTAGTGTCATCAACAAAATGAGTGCAT 794880 795000

NC_000963_1_Rickettsia_prowazekii_str__Madrid_E_chromosome_complete_genome probe_cstm_Rprow_021218_1x_105879 TTAATTTGAGTATCAATTTAGAAATGTATACCACGACAGATATATTTGTGGAAGGAGCTTATTATGTGATCGAAAAAGAAGTATTACATACATAGTACTTTGCTGCAAAATTATAGTTTA 795000 795120

NC_009488_1_Orientia_tsutsugamushi_Boryong_complete_genome probe_cstm_Rprow_021218_1x_10588 TGCTCTTAAAGACTTACGTAATCTAGCAATGGAGATGAATCAATTTGCTAAGGAAGATTGTGAATTAACAAAAGCATTATTTGCTACAGCTTTACCAAAGAACTGGGCTATGAGAGAAGC 1270440 1270560

NC_000963_1_Rickettsia_prowazekii_str__Madrid_E_chromosome_complete_genome probe_cstm_Rprow_021218_1x_105880 TCTTTACCATAAAAACACCTAAATAAAGCACAAGCATATATGTTTTAGCAAATACTATAATACTCTCACCACGTTAATTAGACAATTATAATGTAATCCCTGTTTCAATCTGAATATTTT 795120 795240

NC_000963_1_Rickettsia_prowazekii_str__Madrid_E_chromosome_complete_genome probe_cstm_Rprow_021218_1x_105881 CtttttttGTACAGTAGTAAACCAATGATTGTAGTCCTAACAGGAAATATTACAATATCTTTTAACTATGTCAAAGTATTCAGAGTATTATGATCTTAAGATATTACTATATAACTCAAT 795240 795360

NC_000963_1_Rickettsia_prowazekii_str__Madrid_E_chromosome_complete_genome probe_cstm_Rprow_021218_1x_105882 ACTGGATTCAACAAAATATAAAACGAACACAAAATGTTTGCACTTTTAGACTTTAGTAGTGAAAAATATTCTGTTAAATTGTTTTTAACTACAAATGCATAAATATTTGCAAACTGTATA 795360 795480

NC_000963_1_Rickettsia_prowazekii_str__Madrid_E_chromosome_complete_genome probe_cstm_Rprow_021218_1x_105883 CGTTCTAACATCACAGCAATATTGTTGTACTCACGTCAAAAGTGATTTTAGGTGTATTTAGATCATAAACAGTGTTTTATTTTTGTAACATCTAATAAATTATACAAATTAGATTTTCGT 795480 795600

NC_000963_1_Rickettsia_prowazekii_str__Madrid_E_chromosome_complete_genome probe_cstm_Rprow_021218_1x_105884 TTTAATCATGTTCATTTAACGTCATATCTCTTAGTGTCATTGTTACAAATTATAAATATTTTATAAAATTTTATTaaaaaaaTTGCATAATAACAATTCATAATGGTGAGtaaattttta 795600 795720

NC_000963_1_Rickettsia_prowazekii_str__Madrid_E_chromosome_complete_genome probe_cstm_Rprow_021218_1x_105887 ttataaaaatattttatattaattgtatggatataaataCTCTTTTAAGTTTAAATAATTAGTAGGTTTATGGAAGATATTGATATTTGGCAGAAAAAATTTGAATTTTGTGATTATAGT 795960 796080

NC_000963_1_Rickettsia_prowazekii_str__Madrid_E_chromosome_complete_genome probe_cstm_Rprow_021218_1x_105888 AAAAAATTAATTGGTCGAATTGAATATTTGAATACTATAGTAGATTCACCTATAGATATAACAGAAGTTGAAAAAGGCATTTATTATACCCGTAAATATCATGCTACTCAAATGCGCCAA 796080 796200

NC_000963_1_Rickettsia_prowazekii_str__Madrid_E_chromosome_complete_genome probe_cstm_Rprow_021218_1x_105889 TCAGGAGAGCCATATTATTCTCATCCTATAGAAGTTGCAATCATGCTTGCAGATTTTACAGCTCTTGAAGCTCCTAAACTTTACAAATCATATATGATGAATGTAGCACTTCTGCATGAT 796200 796320

NC_009488_1_Orientia_tsutsugamushi_Boryong_complete_genome probe_cstm_Rprow_021218_1x_10589 TGCTTGCTGTGATATACAGTCACAAAGCTGGTTTGATTATTTTTCAGCTAGTAAAAAATGTCGTAATTATTTAGATCAAAAACAAACTCTGCAACAAGCACAAAATAAGGATTCAGAGCT 1270560 1270680

NC_000963_1_Rickettsia_prowazekii_str__Madrid_E_chromosome_complete_genome probe_cstm_Rprow_021218_1x_105890 ACTATTGAGGATACAATACTTACACATGCTGATATTAGCAAAATTTTTGATAAAAATATTGCAGATAACGTAGAACGTTTAACAAGAATTAAGCCATACGGTAAAATTAGCTCCGGTGAA 796320 796440

NC_000963_1_Rickettsia_prowazekii_str__Madrid_E_chromosome_complete_genome probe_cstm_Rprow_021218_1x_105891 ATGTTAAATTTATTAATCCAAGAACAAAGATATGATATTGCACTTATTAAAGTATTTGATCGACTGCATAATTTACAAACTATCAATGCTAAATCTACAGAAAAGGCTTTAGAAACAGTA 796440 796560

NC_000963_1_Rickettsia_prowazekii_str__Madrid_E_chromosome_complete_genome probe_cstm_Rprow_021218_1x_105892 AAAGAAACTATAGAAAGCTTCTTACTCATAGCAGCTTATTTAGAAATACGAACCGTAGAACAACAACTATTAAATGTTTGCAATAATTTTATCAAGCAACATTTTCCTTCAGAACAAAAG 796560 796680

NC_000963_1_Rickettsia_prowazekii_str__Madrid_E_chromosome_complete_genome probe_cstm_Rprow_021218_1x_105893 GCAATATTTCGATCGGTTTACGAAATTTCTTTTTTCAACTTCAAAATAATACAAAATAAATTAAAGAGGATGCAATACTACTTTAGAAAGAAAAAAGCTTAAATCAACTAAAAACTATTT 796680 796800

NC_000963_1_Rickettsia_prowazekii_str__Madrid_E_chromosome_complete_genome probe_cstm_Rprow_021218_1x_105894 TTATATTTCCAGTGTGCAGCGTCACCACTTTCTGCATTATAATGCATATTATAATCACGTATTTGTATCTCTATTTTATAATTATCCTCAGTTATAATAATAGTATGTAAAGATTGATAG 796800 796920

NC_000963_1_Rickettsia_prowazekii_str__Madrid_E_chromosome_complete_genome probe_cstm_Rprow_021218_1x_105895 CCATTGGGTTTAGGATTAAGGATATAATTTTTAAATTTATCCTTTTCATGATCATAAAGTTCATGAACTATTTTTAAAGCTTTATAACATTTTTCTTCATCTATTACTACTATTCTTATC 796920 797040

NC_000963_1_Rickettsia_prowazekii_str__Madrid_E_chromosome_complete_genome probe_cstm_Rprow_021218_1x_105896 GCAAAAATATCTGTTAATTCTTCTAACTTTATACCTTTACGATATAATTTATATAAAATTGATATTGGATGTTTTATCCTACCTGTTATTTGAGCCGCAATATCATGTTTAGATAAATTA 797040 797160

NC_000963_1_Rickettsia_prowazekii_str__Madrid_E_chromosome_complete_genome probe_cstm_Rprow_021218_1x_105897 TAACTTAAATGTTTAATAACCCTTTGGAGCAAAACTACCTCCTTAATTTATACTAATTCAGTTAATAGTTATACTTACGAATAACTAAACACCAATTAAAGCaataaatttgattaaatt 797160 797280

NC_009488_1_Orientia_tsutsugamushi_Boryong_complete_genome probe_cstm_Rprow_021218_1x_10590 AATGCTGGATGATTATAACATCTTCCGTATGATAAAAATAAGTGAAATTGCAATAAAATAAAAGATTATAGCAATAGCACAAAAGAAAGCTAGTATACAGTTATGCTCCTAAGGCTACTA 1270680 1270800

NC_000963_1_Rickettsia_prowazekii_str__Madrid_E_chromosome_complete_genome probe_cstm_Rprow_021218_1x_105902 TGTTAAAAAGAATTAAGCAAAGAATAGCTTTAAAACACATTGTTGCATAAATCAGTTTTTCAGTATACTACTGCAACTGTATAATGTGTTGTACAGAGCGTTGTTAATATATTACTAGAA 797760 797880

NC_000963_1_Rickettsia_prowazekii_str__Madrid_E_chromosome_complete_genome probe_cstm_Rprow_021218_1x_105903 GGCATTGCATGCGTGAACAGCTAGATAATTGTTCATCTCATAAACTTTTGTAGAGAAACCCAGTACAAATGTGCTATCTCTAAGTAACGGTATCCATTTGTACTAGGTCCCTAAGTCTAG 797880 798000

NC_000963_1_Rickettsia_prowazekii_str__Madrid_E_chromosome_complete_genome probe_cstm_Rprow_021218_1x_105904 GGGCGACAATTCTTACGAGAAGCAATACAACAAGAATAGTTTATTAGACTTATTGCATAACCTATCTTGAGTAGTTATAAACTTAGAAGTCCATACCACCCATGCCACCCATGCCGCCCA 798000 798120

NC_000963_1_Rickettsia_prowazekii_str__Madrid_E_chromosome_complete_genome probe_cstm_Rprow_021218_1x_105905 TGCCTCCACGCATTGGCATTGGTTCTTCTTTATCAGAAGGTTCATCCACAATTAAAGTTTCTGTGGTAATAATTAAAGAAGCAACAGAAGCAGCATCTTGAAGTGCAGTACGTACTACTT 798120 798240

NC_000963_1_Rickettsia_prowazekii_str__Madrid_E_chromosome_complete_genome probe_cstm_Rprow_021218_1x_105906 TAGCTGGATCAATAATTCCTGCTTTAATCATATCGACATATTGCATATCTTGAGCATTAAAGCCATAATTTTTATCATTATGTTCTAGTAATTTACCAACTACTACACCACCGTTTTCAC 798240 798360

NC_000963_1_Rickettsia_prowazekii_str__Madrid_E_chromosome_complete_genome probe_cstm_Rprow_021218_1x_105907 CGGCATTTTTAACAATCTGTTTTAGTGGATCTTTTAAAGCTTCTATTACTATTTCAATGCCAGCTTGTTGATCTTTATTTTCTACTTTAAGCTTTGTTAAAGTTTGTGAAGCATGAAGTA 798360 798480

NC_000963_1_Rickettsia_prowazekii_str__Madrid_E_chromosome_complete_genome probe_cstm_Rprow_021218_1x_105908 ACGTTACACCGCCACCAGCAACAACACCTTCCTCAACCGCTGCTCTGGTAGCAGCAAGTGCATCTTCAACACGATCTTTACGCTCTTTCACTTCAACTTCGGTAGCACCGCCAACCTTTA 798480 798600

NC_000963_1_Rickettsia_prowazekii_str__Madrid_E_chromosome_complete_genome probe_cstm_Rprow_021218_1x_105909 ATACTGCCACTCCTCCGGAAAGTTTAGCTAAACGCTCTTGAAGTTTTTCTTTATCATAATCAGAAGTAGTTTCAGCTATTTGAGATTTAATTTGCAATACTCTATCTTCAATATTTTTCT 798600 798720

NC_009488_1_Orientia_tsutsugamushi_Boryong_complete_genome probe_cstm_Rprow_021218_1x_10591 GCCTGAATTTTAGATAAGTAACTATCTTTTTTAACTAAAAACCGGACGGTATTCTTAGGCAAAAAAGCCTGTATTTACAAGGTTGGTTTTGAGATATTTAACATAAAATTTGGTGATTAT 1270800 1270920

NC_000963_1_Rickettsia_prowazekii_str__Madrid_E_chromosome_complete_genome probe_cstm_Rprow_021218_1x_105910 TATCACCGTTACCATCAACAATTACAGTATTTTCTTTAGAAATTGTAACTCTTTTTGCTGTACCTAAATTTTTAATATTTACATTTTCAAGCTTCATACCCAAATCTTCAGTAATAAGCT 798720 798840

NC_000963_1_Rickettsia_prowazekii_str__Madrid_E_chromosome_complete_genome probe_cstm_Rprow_021218_1x_105911 CACCTTTAGTGAGGATAGCAATATCTTCCATCATTGCTTTTCTTCTATCACCAAAACCAGGAGCTTTTACTGCTGCCACTTTTAAACCACCACGTAATCTATTGACTACAAGCGTCGCAA 798840 798960

NC_000963_1_Rickettsia_prowazekii_str__Madrid_E_chromosome_complete_genome probe_cstm_Rprow_021218_1x_105912 GTGCTTCACCTTCAACATCCTCAGCAATAATTAATAACGGACGTTGTGATTGCACTACAGCCTCGAGTATAGGCAACATCGGTTGTAAATTTGATAATTTCTTTTCAAATAGTAAGATAA 798960 799080

NC_000963_1_Rickettsia_prowazekii_str__Madrid_E_chromosome_complete_genome probe_cstm_Rprow_021218_1x_105913 AAGGATTTTCAAGCTCGGCAACCATTTTTTCAGAATTTGTGACAAAATATGGCGATAAATAACCCCTATCAAACATCATACCTTTAACAACTTCAACATCGAAGCTAAAATTCTTTGCCT 799080 799200

NC_000963_1_Rickettsia_prowazekii_str__Madrid_E_chromosome_complete_genome probe_cstm_Rprow_021218_1x_105914 CTTCAACGGTGATCACGCCTTCTTTGCCGACTTCCTCCATTGCCTTAGCAATTTTTTCGCCGATTTCTTTATCACCATTTGAAGATATAGTTCCAACTTGCGCGATTTCTTCTTGGCTAT 799200 799320

NC_000963_1_Rickettsia_prowazekii_str__Madrid_E_chromosome_complete_genome probe_cstm_Rprow_021218_1x_105915 TGAtttttttACTAGATCTTTTAATTTCTTCTACTACTGCATTTACTGCTAAATCCATACCACGCTTTAAATCCATAGGATTATAGCCTGCTGCAACCAACTTATTACCTTCACGAGCCA 799320 799440

NC_000963_1_Rickettsia_prowazekii_str__Madrid_E_chromosome_complete_genome probe_cstm_Rprow_021218_1x_105916 AAGCTCTAGCAAGTACCGTAGCAGTAGTAGTACCATCACCTGCTACCTCTGCAGCTTTTGTAGCAGCTGATTTTAATAGCTGAGCGCCTGCATTTTTAATTTTATCTTTTAACTCAATCG 799440 799560

NC_000963_1_Rickettsia_prowazekii_str__Madrid_E_chromosome_complete_genome probe_cstm_Rprow_021218_1x_105917 ATTTTGCAACAGTGACACCATCTTTTGTAATTTTCGGTGCACCAAATGATTGCTCGATAAGTACATTTCTACCTTTCGGACCTAAAGTCACCTTTACTGCATCTGCAAGTATATCAATAC 799560 799680

NC_000963_1_Rickettsia_prowazekii_str__Madrid_E_chromosome_complete_genome probe_cstm_Rprow_021218_1x_105918 CTTCGAGCATTTGCTCACGGGCTTTTGAACCGTGTTTAATAAGTTTCGTTGTCATATTTTTTCTCCTAAAAATAATTAATTAATAATACCAAATACATCGCTTTCTTTCATAACGATTAA 799680 799800

NC_000963_1_Rickettsia_prowazekii_str__Madrid_E_chromosome_complete_genome probe_cstm_Rprow_021218_1x_105919 TTTTTCTCCTTTTATTTCAATTTCGGTACCTGCCCATTTACCGTATAAAACTTTATCGCCTACTTTGAGCTCTAAAGGATGAATTTCACCGTTTTTATTAAGCACACCGTTACCTACAGC 799800 799920

NC_009488_1_Orientia_tsutsugamushi_Boryong_complete_genome probe_cstm_Rprow_021218_1x_10592 GAATAGTATAATTGTAGGAATTGATATTTCTAAAGAGACATTTGATGCCGCTGTGTTAATTAATCATAAAGTTCAAACAAGAAAATTTAATAATAATTCTGAAGGATTTAACAAATTAGT 1270920 1271040

NC_000963_1_Rickettsia_prowazekii_str__Madrid_E_chromosome_complete_genome probe_cstm_Rprow_021218_1x_105920 TACTATTTCACCTTGCATTGGTTTTTCTTTTGCTGTATCTGGAATAATAATTCCACCTTTAGTTTTCTCTTCATTTTCGATAGGCTTTATTGCAATTCTATCATGTAATGGTTTAAAAGA 799920 800040

NC_000963_1_Rickettsia_prowazekii_str__Madrid_E_chromosome_complete_genome probe_cstm_Rprow_021218_1x_105921 CATTTTAACCTCCAATTATTAAATCTTTAAATGTACATACTATATATATTTTATTTTTGTTAGTTCAAGGGGGAAAAATAAAAAttttttttATTAGAGATATAtttttttGCAAAATAC 800040 800160

NC_000963_1_Rickettsia_prowazekii_str__Madrid_E_chromosome_complete_genome probe_cstm_Rprow_021218_1x_105922 TCTCAACTAATCTAATATTTAGTTTTACTTCTATTTTAAAGTACAAAAAATCATTATAAATACCACTTTATATGTAGTAAAACAATGATACAGCATAAAAGTTATTATAATTTTGCAATT 800160 800280

NC_000963_1_Rickettsia_prowazekii_str__Madrid_E_chromosome_complete_genome probe_cstm_Rprow_021218_1x_105923 TTACTTTGCCACTTTTTAATTACTTAATATATATAGATTATTACTAATAAAGATGTAAAACAGGGAAATACCATTAATAAACCATATCATTCCTATCTATAAAAATAGCCAAGGAAAATG 800280 800400

NC_000963_1_Rickettsia_prowazekii_str__Madrid_E_chromosome_complete_genome probe_cstm_Rprow_021218_1x_105924 CACACTCCAAAAATTGCTATAATAAACCAATATAGATTGGTATCAAATATTGATGTTGCTTTAAATACAAAATGTTTACACCAGTAATCCAACATGATCTTTAGCCAATTACATCAACGA 800400 800520

NC_000963_1_Rickettsia_prowazekii_str__Madrid_E_chromosome_complete_genome probe_cstm_Rprow_021218_1x_105925 TTCAGTGTTTCTTCCATGATATCGGAAACAAGCATAGATGATTTATCAAAGTACTACACCTTCTGTGCCATTATTAAGCATCATATCTACAAAGCTTATCTAACAATACGTTTTGCATAG 800520 800640

NC_000963_1_Rickettsia_prowazekii_str__Madrid_E_chromosome_complete_genome probe_cstm_Rprow_021218_1x_105926 TAGGCAATTTTAGCAAACGTAATTTCTTACAAGATATACAAGCCATAACTAATATTGAATGAAATATCATGTTTATTATTATGAAGTTTAGCCTGAGGATAAGCATATTCTaaaaaaaaG 800640 800760

NC_000963_1_Rickettsia_prowazekii_str__Madrid_E_chromosome_complete_genome probe_cstm_Rprow_021218_1x_105927 TTTTACTAAGACTTTAAAAAATATGGTTTATGTTTATCATTCAGAATTCTATCCTACTAGAATATTCTAGAACATAATTAAGAGCATTGGTTTAGTACTTAGTTACTTTTTATGATAAGC 800760 800880

NC_000963_1_Rickettsia_prowazekii_str__Madrid_E_chromosome_complete_genome probe_cstm_Rprow_021218_1x_105928 ATTATATTAAACTAAGACGATATTATACGAGAATAATTTTTAGATAATTATCATATTGATGATAATTTATAGCTTATATTGTAGATAAGTAATTAATGCAATATTTTTGACATTCACTGA 800880 801000

NC_000963_1_Rickettsia_prowazekii_str__Madrid_E_chromosome_complete_genome probe_cstm_Rprow_021218_1x_105929 CTTAATGCTTCATATAACAGCACTAGAACAAGCACTTATAATACTTGCTCTACAAGATATATGTTGCATTGTTTTGTAAAGCAATTTACATATTGCATTATACCTAACTTGATTACAGTA 801000 801120

NC_009488_1_Orientia_tsutsugamushi_Boryong_complete_genome probe_cstm_Rprow_021218_1x_10593 AACATGGTTAAAAAGCAGAGAAACTGGACATGTTTGTATGGAAGCAACATGTATCTACTGGAAAAGCTTAGCGAAATATCTGTACGATTATGGCTATAAAGTGAGTGTAGTAAATCCTGC 1271040 1271160

NC_000963_1_Rickettsia_prowazekii_str__Madrid_E_chromosome_complete_genome probe_cstm_Rprow_021218_1x_105930 TCAAGAAAACCATTTTAAATGCTAAATATTAGTATAATCAAGTAATAGAACGACCAAAAACACGCTTGACAATTGATGCTAATGTACAGAAAACATACCTCTTACTACAAAGTATaaaaa 801120 801240

NC_000963_1_Rickettsia_prowazekii_str__Madrid_E_chromosome_complete_genome probe_cstm_Rprow_021218_1x_105931 acaaaaaaTTTTGCAAGAAAGAGCTTATATTATGCAATATGGAATAAAATCTGCAAATAAATCAATGTTGCAATAAATACAACAAAATATTATTATTTTAATACTGAACAATTAATTGTA 801240 801360

NC_000963_1_Rickettsia_prowazekii_str__Madrid_E_chromosome_complete_genome probe_cstm_Rprow_021218_1x_105932 ATTGGTACAACTTATAAATGCAAGACCATATTTTGCTTTACTAAAATTaaaaaaaTTCATTAGATTAATTTTGACCACTAACATCTGAATATATACAGTAACCATTCACTAAATTAAGAT 801360 801480

NC_000963_1_Rickettsia_prowazekii_str__Madrid_E_chromosome_complete_genome probe_cstm_Rprow_021218_1x_105933 TAGTATATAAGAATATTTGACGCTTAAAAATTTTAATTGTACTGTAATTATAAGTTAGATTTTTGTAGTAAAAATTTGACAATTTTACATACATTTAAAGAAATAAATATACACTAAAAT 801480 801600

NC_000963_1_Rickettsia_prowazekii_str__Madrid_E_chromosome_complete_genome probe_cstm_Rprow_021218_1x_105934 TATAAGGATAATACTTGTACAAAGTAGTAGAAATATTAATATATCAATTAATCAAATTATAATTAAAGCTAATCAGTACATCACGTAAATAGGTAATGGTCAATAATTAATTGTAGACCC 801600 801720

NC_000963_1_Rickettsia_prowazekii_str__Madrid_E_chromosome_complete_genome probe_cstm_Rprow_021218_1x_105935 GGTAGTAGTGTTAGTCTAAGAGAGGTATACTATTAATTAAGTAATCCAGTGTTAAAATGCTAATGTATAACATTTTTTACAATGCATACACGGGAATGGAAGATCCATATTATTCAGCCG 801720 801840

NC_000963_1_Rickettsia_prowazekii_str__Madrid_E_chromosome_complete_genome probe_cstm_Rprow_021218_1x_105936 CGATCAGCACTTGATTTTGTAGTTTGAATAATTCTACAACTCCACTTTTAGCAAGTTTTAACATTGCTAAAAACTGCTCTTCAGAAAATGGTTCTTTCTCTGCAGTCCCTTGTACTTCAA 801840 801960

NC_000963_1_Rickettsia_prowazekii_str__Madrid_E_chromosome_complete_genome probe_cstm_Rprow_021218_1x_105937 TTAAATTACCATTACATGCAAAAACAAAATTACTATCCACTTCCGCATCACTATCTTCTAAATAATCTAAATCTAGAACCGCATTACCTTTATATATACCGCAAGAAATAGCGGCAATTT 801960 802080

NC_000963_1_Rickettsia_prowazekii_str__Madrid_E_chromosome_complete_genome probe_cstm_Rprow_021218_1x_105938 GACTAATTAACGGGTTCACTTTTAAAATCCTCTTTTTCATCAACGATCTAATAGCTAGGTGCAATGCTACATAGCTTCCAGTTATCGCCGCAGTTCTAGTACCGCCATCTGCATTAATAA 802080 802200

NC_000963_1_Rickettsia_prowazekii_str__Madrid_E_chromosome_complete_genome probe_cstm_Rprow_021218_1x_105939 CGTCACAATCTATGATAATTTGTCTCTCCCCGAGTTTCTGCAAATTAATTACACATCGCATCGCCCTACCTATCAAACGCTGTATCTCTTGAGTTCTGCCACCTTGTTTTCCGAGTGCAG 802200 802320

NC_000963_1_Rickettsia_prowazekii_str__Madrid_E_chromosome_complete_genome probe_cstm_Rprow_021218_1x_105940 CTTCTCTTTTTATACGATGCGATGTAGACCCTGGGATCATACCATACTCAGCAGTAATCCATCCTTGATTTTGCCCCCTTAAGAATGGAGGCACAGTAGTTTCACAAGTAGCACTACACA 802320 802440

NC_000963_1_Rickettsia_prowazekii_str__Madrid_E_chromosome_complete_genome probe_cstm_Rprow_021218_1x_105941 TTACATGCGTATTACCGATTTTAATCAGACATGATCCTTCTGCATTAATCAGAGGAGATAATTCTAATGAAATAGGACGTAATTGGTTGCTTTTTCTTCCTGACTGTCTCATAATATAAT 802440 802560

NC_000963_1_Rickettsia_prowazekii_str__Madrid_E_chromosome_complete_genome probe_cstm_Rprow_021218_1x_105942 TTGTACTCCTAATAATATTAAAGAATCGGTATACGAAAGCAAATTTCAAAAAGAGCAAAATGTTTTATTGATCACAACATACATATTTAGTACGAAAAATAAAATAACGTAGCAAATTTT 802560 802680

NC_000963_1_Rickettsia_prowazekii_str__Madrid_E_chromosome_complete_genome probe_cstm_Rprow_021218_1x_105944 AAGAGAATCCTAACGTAGAAACTCAAGTAGTAGACAACGAAGAAATAGTACGCTTAAAAGCCGAGATTGAAGAGTTAAAAGATAAGCTAATTAGGACTACTGCAGAAATAGATAATACAA 802800 802920

NC_000963_1_Rickettsia_prowazekii_str__Madrid_E_chromosome_complete_genome probe_cstm_Rprow_021218_1x_105945 GAAAGCGGTTAGAAAAAGCCCGTGACGAAGCAAAAGATTACGCAATTGCTACATTTGCTAAAGAATTGCTAAATGTTAGTGATAATCTTGCAAGGGCATTAGCACATAAACCAGCAAATT 802920 803040

NC_000963_1_Rickettsia_prowazekii_str__Madrid_E_chromosome_complete_genome probe_cstm_Rprow_021218_1x_105946 CTGATGTAGAAGTCACGAATATTATTTCCGGTGTGCAGATGACTAAAGATGAGTTAGATAAAATCTTTCATAAGCATCATATTGAAGAAATCAAGCCAGCAATAGGTTCAATGTTCGACT 803040 803160

NC_000963_1_Rickettsia_prowazekii_str__Madrid_E_chromosome_complete_genome probe_cstm_Rprow_021218_1x_105947 ATAATTTACATAATGCAATTTCACATATAGAACATCCTGATCATGAACCAAATAGTATTATTACTTTAATGCAATCAGGGTATAAAATTAGGGATAGACTACTTCGACCTGCTGCAGTAC 803160 803280

NC_000963_1_Rickettsia_prowazekii_str__Madrid_E_chromosome_complete_genome probe_cstm_Rprow_021218_1x_105948 AAGTAGTTAAAAAACCTTAATGCTAAGGTAGAGCTGAAAGAATTTGCGTTGTAGTTCTACAAAAACTGAAGTATTCTAAGTACTGCATTCCTGCTCTTGTAAACTCTTTACGCTTTTTGA 803280 803400

NC_000963_1_Rickettsia_prowazekii_str__Madrid_E_chromosome_complete_genome probe_cstm_Rprow_021218_1x_105949 AGCTGATCTTTATATAGAAAATGAGAAGAAGAATATGAATAGAACCGCTGTATTTTGGCTAGTTTTCTTGACACTTTTTATAAGCGGTTTTATGCTAATTACGGATACAATAAAACCATT 803400 803520

NC_000963_1_Rickettsia_prowazekii_str__Madrid_E_chromosome_complete_genome probe_cstm_Rprow_021218_1x_105950 TTTTATTGCATTTATAATATCTTACTTACTACAACCTGCTATATATTTTATTGAATCAAAACTTGAAATATCAAATAAATTAGCATCAAGCATAATTTATCTGATATTTTTGAGTATATT 803520 803640

NC_000963_1_Rickettsia_prowazekii_str__Madrid_E_chromosome_complete_genome probe_cstm_Rprow_021218_1x_105951 TTTCTTAATATTCACTATATTAATACCAATAATTTATGGACAAATTTTTACTTTTATCAATAATATCCCAAAATATAAAAATTATTTACAATCAGAAATATTACCTCCAATTATGGGAAA 803640 803760

NC_000963_1_Rickettsia_prowazekii_str__Madrid_E_chromosome_complete_genome probe_cstm_Rprow_021218_1x_105952 AATTTATTCAATTGAACCCGATATCGCTGATAAAATCAAGCATTCTTTGAGTGATTTTATAAATAGTATATTTACTATACTTGGCAGTATTGCTAATAATTTTTGGCGttatacaattat 803760 803880

NC_000963_1_Rickettsia_prowazekii_str__Madrid_E_chromosome_complete_genome probe_cstm_Rprow_021218_1x_105954 AATCCTAGAAATATTATCGGCAATTAATAATTTATTATCTTCTTACATAAGAGGACAACTAAATATTTGCTTATTGTTATCTACTTACTATAGCATTGCATTTACCTTAATAGGTATCGA 804000 804120

NC_000963_1_Rickettsia_prowazekii_str__Madrid_E_chromosome_complete_genome probe_cstm_Rprow_021218_1x_105955 TCTTGCTCTTTTACTTGGCATTTTAACAGGATTTTTAGTTATTATTCCTTTGCTTGGTACTTTTATATCATTTCTTTTAACTCTAATTATTGGCTATTTAACTTTCGGCATAACTAGCAA 804120 804240

NC_000963_1_Rickettsia_prowazekii_str__Madrid_E_chromosome_complete_genome probe_cstm_Rprow_021218_1x_105956 ATTACTATATATAATGATAATTTATCTCATTGGAAATATTGGTGAATCTTATATTTTAACCCCTAAAATTATCGGAGATAAAATAGGTTTACATCCTCTTTGGATTATATTCGCTATTTT 804240 804360

NC_000963_1_Rickettsia_prowazekii_str__Madrid_E_chromosome_complete_genome probe_cstm_Rprow_021218_1x_105957 TGCTTGCGGCAGTTTATTTGGAATTATTGGAATATTTTTTGCTATACCAATTGCAGGAATTACAAAAATTTTACTCTTGAATCTCATTAAATTTTATAAATCTAGTAAGTTCTATAGAAC 804360 804480

NC_000963_1_Rickettsia_prowazekii_str__Madrid_E_chromosome_complete_genome probe_cstm_Rprow_021218_1x_105958 AGACGTCTAAAAAATTCTTATATAGTGCAGCAATATATATTTCATTTTACTCCTTCTAATAAATATCATCCTGATGAATTTATCGTTTCTAGTTCTAATGCGCAAGCTTATAATATTATA 804480 804600

NC_000963_1_Rickettsia_prowazekii_str__Madrid_E_chromosome_complete_genome probe_cstm_Rprow_021218_1x_105959 AAAAACTGGCAGTATTGCTTTGGCGCAAATCCTTATAAGTTTACTTTATTAATTAAAGGACCTTCTTCATCAGGTAAAACTTATTTAACAAAAATCTGGCAAAATTTAAGCAACGCATAT 804600 804720

NC_009488_1_Orientia_tsutsugamushi_Boryong_complete_genome probe_cstm_Rprow_021218_1x_10596 CATTGAATTTCTTGAAACACAAATTAAAGAAATTGAACAACTAATCAATGACCATATTAAAAATAACAAAGATCTTCACAATAAAGCTATGTTACTTGAATCAATACCAGGTATAGGAGC 1271400 1271520

NC_000963_1_Rickettsia_prowazekii_str__Madrid_E_chromosome_complete_genome probe_cstm_Rprow_021218_1x_105960 CTTATTAAAGATAttttttttAATGAAGAAATTCTAGAAAAATATAATACTTTTATTATTGAAGATATTGAAAACTGGCAAGAACCGACATTGCTTCATATATTTAATGTTATTAATGAA 804720 804840

NC_000963_1_Rickettsia_prowazekii_str__Madrid_E_chromosome_complete_genome probe_cstm_Rprow_021218_1x_105961 AAACAAAAATATCTTTTACTTACCTCATCAGATAAAAACAATAATTTTACTTTACCAGATTTATCTTCACGTATCAAATCAGTACTAAGTATTCTATTAAATGCACCTGATGATGAATTA 804840 804960

NC_000963_1_Rickettsia_prowazekii_str__Madrid_E_chromosome_complete_genome probe_cstm_Rprow_021218_1x_105962 ATTAAAATCCTAATATTTAAGCATTTTTCCATTTCTTCAGTAACAATATCTCAACAAATAATAGATTTTCTTTTAGTAAATTTGCCTAGGGAATATTCTAAAATCATAGAAATATTAGAA 804960 805080

NC_000963_1_Rickettsia_prowazekii_str__Madrid_E_chromosome_complete_genome probe_cstm_Rprow_021218_1x_105963 AATATTAACTATTTTGCTTTAATTTCCAAAAGAAAAATAACAATTTCATTAGTAAAAGAAGTTTTAAATAACTATAAGAATAAAATATGATAACTTTTTGTTTTACTTAAAAATATTCTA 805080 805200

NC_000963_1_Rickettsia_prowazekii_str__Madrid_E_chromosome_complete_genome probe_cstm_Rprow_021218_1x_105964 ACATAACATTCCTATATAGTAACCTTTTGAAAAAATTGTGTATGCATACaaaaaaaTTTTAATAGATCATAAAAACATACTGTAGGCTTACAATATATAAAGACTTTTTGAGAGTATTTT 805200 805320

NC_000963_1_Rickettsia_prowazekii_str__Madrid_E_chromosome_complete_genome probe_cstm_Rprow_021218_1x_105965 AATTTTTTACGAGATACACTATAGAATAGGATACTAGTTAATTTATGTATTTTTCTTTATCTAGAGCCCTTGGATCTCTATTACTCGGTTTGATATAAGATAATATATGTTAGAAATATA 805320 805440

NC_000963_1_Rickettsia_prowazekii_str__Madrid_E_chromosome_complete_genome probe_cstm_Rprow_021218_1x_105966 AATGATACCACTATTATATTTTCGTTTTCTTATAGGCTTATGGCTATTTCTACTTATGCAAAAGTAAGTCGTATGGACTACTTTTTTGATAGGAACAATATAGTGCCATGTATGTCATCT 805440 805560

NC_000963_1_Rickettsia_prowazekii_str__Madrid_E_chromosome_complete_genome probe_cstm_Rprow_021218_1x_105967 ATGTAAACTATATTAATAATAGGATTGTATTTAACGCAAAGTGTCACATTACTAAACGTTTGTCTATTGTAAAACTTGTATTTGTGTTGTGTATGATAACTTAGATTTCTATGATACTGA 805560 805680

NC_000963_1_Rickettsia_prowazekii_str__Madrid_E_chromosome_complete_genome probe_cstm_Rprow_021218_1x_105969 ATTAGTAATCATTTATTCTATTTAGTTCATAAATATTATGATAATATAAAACAATACTGATCTATACTTTTAATTCAACTAATTGTAGTGACTTAATACAATAAGCGATTCCAGATTAAC 805800 805920

NC_009488_1_Orientia_tsutsugamushi_Boryong_complete_genome probe_cstm_Rprow_021218_1x_10597 AAAAACACAAGCTATAGTTCTGGCTTTTTTAGCAGATATTGAGAAATTTAGTTCTGCTAAACAAGTTGTAGCTTTTGTAGGTCTTAATCCTAAGCATCGTCAATCTGGTAGTTCTGTGCG 1271520 1271640

NC_000963_1_Rickettsia_prowazekii_str__Madrid_E_chromosome_complete_genome probe_cstm_Rprow_021218_1x_105970 ATTTTAGTTATATACTTTATCTGAGTCATCTACTCCAGCTTTAATGATTTCAAGCAATGAAAAAACCTTACAATATTTTGCAAAGCTTTTCGTTGATTTGTTGTAGCTCATCAAAAGCAA 805920 806040

NC_000963_1_Rickettsia_prowazekii_str__Madrid_E_chromosome_complete_genome probe_cstm_Rprow_021218_1x_105971 TACTAAAATCTTGTTGTATTAAATAGGTTTCTATAGTTATGTTTTAAGTATTCTATAATAACTTAATTATAATGATTTTTATATTTACACTCATTCATGATAAAATTGAACTGAAATTTG 806040 806160

NC_000963_1_Rickettsia_prowazekii_str__Madrid_E_chromosome_complete_genome probe_cstm_Rprow_021218_1x_105972 CTACCCTCTAGGATTACAAATTATCTCATTAATTATTTTATCATTTTCTTGTAATGACATTAAATGACTAAATAATATTACTTAGCCATTATGCAAAAATACTATTTATTTAATAGATTG 806160 806280

NC_000963_1_Rickettsia_prowazekii_str__Madrid_E_chromosome_complete_genome probe_cstm_Rprow_021218_1x_105973 AACTATTGTCACATTTAAGCTTGAGTTTAAGATCGAATGATGTGTTTATTGATATACTAAAAATTTTCAATTGGTGGGCGATGACGGACTCGAACCGCCGACATTCTCGGTGTAAACGAG 806280 806400

NC_000963_1_Rickettsia_prowazekii_str__Madrid_E_chromosome_complete_genome probe_cstm_Rprow_021218_1x_105977 ATTACCTAGTCCATTTATATTTTAATTTTTAATTTCTTCATTAGCATCTTGATGCACATTGCCTTTAGCATTGATATCTCTATCTACAAATTCAATATATGCTATCGGTGCTAAATCACC 806760 806880

NC_000963_1_Rickettsia_prowazekii_str__Madrid_E_chromosome_complete_genome probe_cstm_Rprow_021218_1x_105978 ATAACGGAACCCAGACTTTACTATTCTAGTATAACCTCCAGGTCTATCTTTATATCGGACACCTAAAATGtttataattttttctactgctcttttatcttttatttttGATAAAACGCT 806880 807000

NC_000963_1_Rickettsia_prowazekii_str__Madrid_E_chromosome_complete_genome probe_cstm_Rprow_021218_1x_105979 TCTTCTCACTGCTAAATCAGGTTTTTTAGCTTTAGTAATTAGAGCTTCAATGTAAGGTCTTAATTCTTTAGCTTTTGGTAGAGTAGTTTTAATTTGTTCATGTATTACAAGTGATACAGC 807000 807120

NC_009488_1_Orientia_tsutsugamushi_Boryong_complete_genome probe_cstm_Rprow_021218_1x_10598 TAGCGCTAGTAGAATCTCTAGAACTGGTAATTCAGATCTACGTAAGTCTTTTTATATGCCTGCTATGTCTGCCTTAAGACATAATTGTATTATCAAGCATTTTTCTCAACGTTTATCTAA 1271640 1271760

NC_000963_1_Rickettsia_prowazekii_str__Madrid_E_chromosome_complete_genome probe_cstm_Rprow_021218_1x_105980 CATGTTAGCAAGCATTGATTGCCTATGACTACTTGTTACATTTAACCTTCTACCTTTAATTCTATGTCGCATTTTTATTAGTCCTTAATTATAAGAATCTTCATAACGTTTAGATAATTC 807120 807240

NC_000963_1_Rickettsia_prowazekii_str__Madrid_E_chromosome_complete_genome probe_cstm_Rprow_021218_1x_105981 TTGAATATTTTCTGGTGGCCAATCTGGTACATCCATACCAAATCTTAAATTAAACTTTGCCAATATTTCCTTAATCTCATTTAAAGATTTTCTACCAAAATTTGGAGTTCTTAACATATC 807240 807360

NC_000963_1_Rickettsia_prowazekii_str__Madrid_E_chromosome_complete_genome probe_cstm_Rprow_021218_1x_105982 GGATTCCGTTCTTTTTACTAGATCTCCtatatatattatattatCATTTTTTAAACAATTTGCAGATCTAACTGATAATTCCAATTCATCAACTCTCTTAAGTAAATATGGCGAAAATGG 807360 807480

NC_000963_1_Rickettsia_prowazekii_str__Madrid_E_chromosome_complete_genome probe_cstm_Rprow_021218_1x_105983 TAAAGAATCAGTTTTTACTTGCTTATCCTCTTCTTGCTCTTCAAAAGCAATAAATAATTGTAATTGCTCTTGTAAAATACGTGCAGCTAATCCTACAGCCATTTCAGGTAATACATCACC 807480 807600

NC_000963_1_Rickettsia_prowazekii_str__Madrid_E_chromosome_complete_genome probe_cstm_Rprow_021218_1x_105984 GTTAGTTTCTACAAACATAATCAATTTATCATAATCAGTAACTTGACCCACCCTAGTATTTTCGACTTTATAAGTAACACTTTTTACTGGGTTAAATAAAGCATCTATAGCTATTTCGCC 807600 807720

NC_000963_1_Rickettsia_prowazekii_str__Madrid_E_chromosome_complete_genome probe_cstm_Rprow_021218_1x_105985 AATAGGTAAGTTATCCTCATAGCTATTTGTGCTAAGTACATATCCTTTACCAACCTTACATGTTAACTCCATTTCAAGCTGCTTATTCTTAGCTAAATTACAAATTACATGATCCGGATT 807720 807840

NC_000963_1_Rickettsia_prowazekii_str__Madrid_E_chromosome_complete_genome probe_cstm_Rprow_021218_1x_105986 CAATATTTCTACATCATGCCCTGTATCAATCATACCTGCTGTTACAACACAAGGCCCCATTGCTTTTAATTTTATGATACGTTTTTCCGAAACATGCATTTTGACTTCAATACCCTTAAT 807840 807960

NC_000963_1_Rickettsia_prowazekii_str__Madrid_E_chromosome_complete_genome probe_cstm_Rprow_021218_1x_105987 GTTTAAAATCACTTCTGATACATCTTCTTGTACACCAGGTATAGAAGAAAACTCATGCTCTATAGCAGGAATCTTTATAGAAGTAATGGCCGCACCTTGTAAAGAAGATAATAACACTCT 807960 808080

NC_000963_1_Rickettsia_prowazekii_str__Madrid_E_chromosome_complete_genome probe_cstm_Rprow_021218_1x_105988 TCTCATAGCATTACCAAGTGTTAAACCAAAACCTCTTTCTAAAGGTTCAACTATAATTTTAGCTTTATTATTGGTTTCCGGGAAATTTTCATATGTGACTCTATTCGGCTTTATTAAAGT 808080 808200

NC_000963_1_Rickettsia_prowazekii_str__Madrid_E_chromosome_complete_genome probe_cstm_Rprow_021218_1x_105989 ATTCCAATTTTTACTTAATGATAACATTTAATACCTATGATTCTCTTATACTCTTCTTCTTTTTGGCGGTCTTACTCCATTATGAGCAATGGATGATACATCTAAAATTGATGTAACCAC 808200 808320

NC_009488_1_Orientia_tsutsugamushi_Boryong_complete_genome probe_cstm_Rprow_021218_1x_10599 CACTGGTAAACCCAAAATGCTTATCCTTACTGCTGCTATGCGTAAGTTATTACATAGAACATCTTATAATCAAAAGTAGGTTCCTTTCTTTTGTATCTCTAAACTGGATTATTTTTCTTC 1271760 1271880

NC_000963_1_Rickettsia_prowazekii_str__Madrid_E_chromosome_complete_genome probe_cstm_Rprow_021218_1x_105990 AAAATTTTGCCCGAATAAAGCACGCATTGCTGATTCACGCTGTGCTCCAGGGCCTCCAATCCTAATAGAAATAGTCTTTAGGCCACATTCTTTTGCCTTTTCCGATGCTTTATCAATCGT 808320 808440

NC_000963_1_Rickettsia_prowazekii_str__Madrid_E_chromosome_complete_genome probe_cstm_Rprow_021218_1x_105991 TACTTGAGCTGCATAAGGCGTTGCCTTTCTTGCACCCTTAAAACCATTGCCCCCTGCTGATGCAGAAGAAATAGTATTACCTTGAATATCAGTAAATGTTACTATAGTATTATTAAATGA 808440 808560

NC_000963_1_Rickettsia_prowazekii_str__Madrid_E_chromosome_complete_genome probe_cstm_Rprow_021218_1x_105992 CGCTCGGATATGTACAACACCAAGAGTGATAGTCTTTTTCTTTTTCTTAACTTTAATAGTCTGATTCATTTATTTACTCTATACTTAAACTATTATTTTACAGtttttttCTTTCCAGCT 808560 808680

NC_000963_1_Rickettsia_prowazekii_str__Madrid_E_chromosome_complete_genome probe_cstm_Rprow_021218_1x_105993 ATCGCAATAGCTTTACCTTTTCTAGTTCGAGCATTAGAATGTGTATTCTGCCCCCTAACAGGCAACTTACGTATATGCCTAAGCCCTTGATAACATCTTATATCTTTCTTCTTTTTAATA 808680 808800

NC_000963_1_Rickettsia_prowazekii_str__Madrid_E_chromosome_complete_genome probe_cstm_Rprow_021218_1x_105994 TTAAGCGTAACTTCTCGCTTTAAATCACCCTCAACTTTATACTCATTTTCAATAATATTACGTAAGCTTATTAACTCTTGATCAGTCAGCACTTTAACCTTTTTATCTTTTGATATTTTT 808800 808920

NC_000963_1_Rickettsia_prowazekii_str__Madrid_E_chromosome_complete_genome probe_cstm_Rprow_021218_1x_105995 GCTTTATTACAAATCTCTGCTGCCATAGTTGAACCAAGACCGTAAATATAAGTTAAACTCACAACTAAACGTTTATTATCAGGAATATTAACACTTGCAATTCTTGCCACAAATAATCTC 808920 809040

NC_000963_1_Rickettsia_prowazekii_str__Madrid_E_chromosome_complete_genome probe_cstm_Rprow_021218_1x_105997 AAATTACCACTATTTTTATAATAATCTATTAATGGATATGTTTCTGTTTTATATACTTTAATTCttttttttATTACTTCTTTATTATCGTCCTTCCTATAATCAAATACACTAGAACTG 809160 809280

NC_000963_1_Rickettsia_prowazekii_str__Madrid_E_chromosome_complete_genome probe_cstm_Rprow_021218_1x_105998 CAAACATCACATACGTGCTCAATTTTAGGCTGCAAGAAATGAATGTTATATATTTTACCGCAATTTTTACAACTATACCTTCCCAAGACCCTTTTAATTAATAATTCATCTGCAACATCA 809280 809400

NC_000963_1_Rickettsia_prowazekii_str__Madrid_E_chromosome_complete_genome probe_cstm_Rprow_021218_1x_105999 AAATATATTATTTTAATTTGTGGTTTTTGAATAAATGCTTCAAAGAATTTTGCTTGCTCTAAATTACGAGGATATCCATCTAATATATAGCCATTTTTATATTTAGAGGATAATaaaaaa 809400 809520

NC_009488_1_Orientia_tsutsugamushi_Boryong_complete_genome probe_cstm_Rprow_021218_1x_106 CTATTAATGGCATTAGTAGTGGACTATTTGCCCTTGATTCAAAGCTTGGAGGATTTAAAAATTCTGACCTAATAATATTAGCTGGCAGGCCTTCAATGGGTAAAACTTCTTTAGGAGTTA 12600 12720

NC_000963_1_Rickettsia_prowazekii_str__Madrid_E_chromosome_complete_genome probe_cstm_Rprow_021218_1x_106000 aCTTTGATTACCTGATTAACTATTTCATTAGGAATCAGTGCACCTTGCTTAACATAATTATTAATTAATTCTGCTTCACTAGTTGATGTTTTAATAATTGTTCTAAATATATCACCAACT 809520 809640

NC_000963_1_Rickettsia_prowazekii_str__Madrid_E_chromosome_complete_genome probe_cstm_Rprow_021218_1x_106001 GCTATATGTGGTAAATCAAtttttttAGCTATCTTTTTGCCTTGTGTTCCCTTTCCAGCTCCTGGAGGACCTaaaaaaaTTACTATCACTATTTAAGCTCatttttaattttttaatttt 809640 809760

NC_000963_1_Rickettsia_prowazekii_str__Madrid_E_chromosome_complete_genome probe_cstm_Rprow_021218_1x_106002 atttttttCATTAAACCTTCATACTTACTACTAAATAAGTAAGTTTGAATTTGAGTCATAGTATCAAGTACCACATTCACTACAATTAAAAAACTTGTACCTCCTAAAGAAAGAGAAATT 809760 809880

NC_000963_1_Rickettsia_prowazekii_str__Madrid_E_chromosome_complete_genome probe_cstm_Rprow_021218_1x_106003 ACATATTTATTCATTAATAGCTCCGGAATTACACATATTACACTTAAATATATCCCACCTATCACTGTAAGTCTTGTAAGTATATAATCAAAATAATCAGATGTATTTTTTCCTGGTCTT 809880 810000

NC_000963_1_Rickettsia_prowazekii_str__Madrid_E_chromosome_complete_genome probe_cstm_Rprow_021218_1x_106005 aCTGGTTTTCCATGACCTAAGTAATAAGTAAGCATACTCATAGTATCTGAATTACTATTAGAAAAACTAGCAAGCGTAGTAGGAAATAGTAAAATTGAACTAGCAAATATTGGTGGTATT 810120 810240

NC_000963_1_Rickettsia_prowazekii_str__Madrid_E_chromosome_complete_genome probe_cstm_Rprow_021218_1x_106006 ACACCAGAAGTATTTAATTTAAGAGGCATATGAGTCGCTTCTCCCCCATAAATTTTATTGCCTACTTGTCTTTTGGGATACTGAACCAATAATTTTCTTTGTGCTTTTTCaaaaaaaaTA 810240 810360

NC_000963_1_Rickettsia_prowazekii_str__Madrid_E_chromosome_complete_genome probe_cstm_Rprow_021218_1x_106007 ATTATAGCGATTAATAAAACTACTCCGATACAAACAGTTATTGCTATTAAAGGCGACAATGCGCCTTTTCTTGATAATTCAAACATACTAATAATAGCACTAGGTACTCCAGAAATTATA 810360 810480

NC_000963_1_Rickettsia_prowazekii_str__Madrid_E_chromosome_complete_genome probe_cstm_Rprow_021218_1x_106008 CCTATAAATATAATTAAAGACGTACCATTGCCTATTCCACGTTGCGTAATTTGTTCTCCTAACCACATCAATAGAATTGTGCCTACAACCAAAGTAATCACAGTTGTCACTCTAAAGAAA 810480 810600

NC_000963_1_Rickettsia_prowazekii_str__Madrid_E_chromosome_complete_genome probe_cstm_Rprow_021218_1x_106009 AACCCCGCTAAAATTACTACAGGACCGGTATTTGTAACCATTGATTCTAAACTTAGAGCAACACCATAAGCTTGGAAAGAAGCAAGCAGAACTGTTAAATACCTTGATAACTGATTTATT 810600 810720

NC_000963_1_Rickettsia_prowazekii_str__Madrid_E_chromosome_complete_genome probe_cstm_Rprow_021218_1x_106010 TTTCGTTTACCAGTTTCTCCTTCtttttttAAATTTTCTAAAGGTTTATATGCAACCGACATTAATTGGATAATAATTGATGCGGTAATATATGGCATAATTGCTAAAGCAAAAATAGAC 810720 810840

NC_000963_1_Rickettsia_prowazekii_str__Madrid_E_chromosome_complete_genome probe_cstm_Rprow_021218_1x_106011 ATTCTACCTAACGACCCGCCAGATAGCATATTGAACATCCCGAGTATTCCAAATTGATTCTTTTCAGCTACACTATTTAAAGCAATTGAATCTATACCTGGTATAGGTATAAAAGACCCA 810840 810960

NC_000963_1_Rickettsia_prowazekii_str__Madrid_E_chromosome_complete_genome probe_cstm_Rprow_021218_1x_106012 AATCTACATATGATTAGCATGAAAAGAGTAAAAATAATGCGATTAACTAAATCATTACTGGATTTTTTAGAAAAATTTTGCCCCATATATTATAATAATTGTCCACCTACTTTCTCAATT 810960 811080

NC_000963_1_Rickettsia_prowazekii_str__Madrid_E_chromosome_complete_genome probe_cstm_Rprow_021218_1x_106013 AAATACTTAGCTTTAGAAGAATAAGCATCTAATTTTAATGATAAAGGAGAAGCAAAATCATCACTACAAATAGATAATAACTTTACTAAATTTTTATTATTAATTAAACCAACTTCGAGT 811080 811200

NC_000963_1_Rickettsia_prowazekii_str__Madrid_E_chromosome_complete_genome probe_cstm_Rprow_021218_1x_106014 AACTTTTCTTTGGTAATAATATCATTAGTACTTAAACGACCATCGGTTAATGCTTCTTCAATATTGTAAATATTTATTATATTATATTTTTTAGTTGAAATACACTTAAAACCTCTTTTA 811200 811320

NC_000963_1_Rickettsia_prowazekii_str__Madrid_E_chromosome_complete_genome probe_cstm_Rprow_021218_1x_106015 GGTAATCTTTTAATCATAGGTGTTTGACCACCTTCAAAACCTTTTATTGCAACACCTGATCTAGATTTTTGACCTTTAATTCCTCTACCAGCAGTTTTTCCTTTACCACTACCAATACCT 811320 811440

NC_000963_1_Rickettsia_prowazekii_str__Madrid_E_chromosome_complete_genome probe_cstm_Rprow_021218_1x_106017 CCCTCTGATTGAATTAGTATTTTTAAGAATAACGCTCTTATTAATTTTATTTAAACCAAGCCCAATTAATGTAAATCTTTGATCATACTTACATCCTATAGCACTTTTAATTTGAGTAAT 811560 811680

NC_000963_1_Rickettsia_prowazekii_str__Madrid_E_chromosome_complete_genome probe_cstm_Rprow_021218_1x_106018 TTTTATATTATTGATTTTATTATTCATAACTTCACCTTACCTATGAAACATTTATTCATTAACTTGAATATAAGATTTGATAGATATTTCATGTACTTTTTTATCTCTTCTTATAGCAAT 811680 811800

NC_000963_1_Rickettsia_prowazekii_str__Madrid_E_chromosome_complete_genome probe_cstm_Rprow_021218_1x_106019 AGATTTTGGTGAGGCAAGCTTATTTAATGCATCAAATGTTGCAGAAATCATTGCATAAACATTAGTTGAACCTATTGATTTAGCAACAATATCATGTACACCTAAAGAATCAAAAATCGC 811800 811920

NC_000963_1_Rickettsia_prowazekii_str__Madrid_E_chromosome_complete_genome probe_cstm_Rprow_021218_1x_106020 TCTCATAGATCCACCCGCTATAACACCTGTACCCGCTTTAGCTCTTCTTAAAATTACTTTAGCAGCCCCACTTTTACCAACAACGTCATGATGAATAGTCCTATTTTGATATAATGGAAC 811920 812040

NC_000963_1_Rickettsia_prowazekii_str__Madrid_E_chromosome_complete_genome probe_cstm_Rprow_021218_1x_106021 CTTCATCATTCGCTTTTTAGCGGCTTGTTTCGCCTTTCCCCGAGCTTCGTTTACTTCTTTAGCTTTCCCATGTCCAGCCCCTACTCTTCCAGCTTTATCACCAACAACTACATAAGCAGA 812040 812160

NC_000963_1_Rickettsia_prowazekii_str__Madrid_E_chromosome_complete_genome probe_cstm_Rprow_021218_1x_106022 AAAAGCAAATCTTCTACCACCTTTTACTACCTTGGTAACTCTATTGACATCAACTAAAACCTCGCTTAAAGCCTCTTCAttttttttAACTTTAGACATTATTACAACCTATAACCTAAA 812160 812280

NC_000963_1_Rickettsia_prowazekii_str__Madrid_E_chromosome_complete_genome probe_cstm_Rprow_021218_1x_106023 ATTTTATTTTCTCTCTAGCTGCATCAGCTAAAGCTTTTATAATACCGTGATACTTATATCCACCTCTATCAAATACTACTTCTTTTATACCTGCAGAATCAGCtttttttGCTATTTCTT 812280 812400

NC_000963_1_Rickettsia_prowazekii_str__Madrid_E_chromosome_complete_genome probe_cstm_Rprow_021218_1x_106024 TACCAACTTTGATAGCATTTTCAATATTACAATGAGAtttttttattttttttatttttttATCTAAAGTTGAAGCAGCAGCAATTGTTATAGACTTAGAATCATCAATAATTTGTGCAT 812400 812520

NC_000963_1_Rickettsia_prowazekii_str__Madrid_E_chromosome_complete_genome probe_cstm_Rprow_021218_1x_106025 ATATATGTCGACACGATTTAAATATCGATAATCTCATTCTATTAGATGTTTTAGATATTTTATATCTTATCCTACTTTTCCTTTTTTCAAATTTTAGCTTAGCACTACGCATATTTAACT 812520 812640

NC_000963_1_Rickettsia_prowazekii_str__Madrid_E_chromosome_complete_genome probe_cstm_Rprow_021218_1x_106026 CTTAAATTTAATTTTTCTTACCTTCTTTACGCTGTATAAATTTATTTTCAAATTTAATGCCTTTTCCTTTATAAGGCTCAGGTGGTCTCTGTTTTATAATAATTGAAGCGAATTGTCCTA 812640 812760

NC_000963_1_Rickettsia_prowazekii_str__Madrid_E_chromosome_complete_genome probe_cstm_Rprow_021218_1x_106027 ATTTTTCTTTATCTATCCCTTCAAGAAGAATAATATTCTGCTTAGGCAGATCTATTTTAATATTTGATGGTATTTCAATTTTTGTATTATGGCTTTTAGCAAGCATTAAATTTAAATATT 812760 812880

NC_000963_1_Rickettsia_prowazekii_str__Madrid_E_chromosome_complete_genome probe_cstm_Rprow_021218_1x_106028 TACCTTTTACCATTGCCCTATAACCGACACCGTTAATTTCAAGCTTTAATTTAAAACCTTCTTTAACTCCAGTGATCATATTACATATTATACTCCTTGCAGTACCCCACATAGCACGTG 812880 813000

NC_000963_1_Rickettsia_prowazekii_str__Madrid_E_chromosome_complete_genome probe_cstm_Rprow_021218_1x_106029 CATTTTTATTTACAGCTAAAGGTTTTACTACAAGCTTATTTTCTTCCATTATAATAGCTATATTACCTTTAAAAGTTTTCGATAATTCACCTTTAGGACCGGATATTTTTACTTCTAAAT 813000 813120

NC_009488_1_Orientia_tsutsugamushi_Boryong_complete_genome probe_cstm_Rprow_021218_1x_10603 ATTAATACATCTACAGTTAAATTTATATATCAATTTAACGAGAATAAACTACATAGCAGATTTTATAAGGCGTATAAAGCTCACAATAACTCTTCCTTTGACTTATTTTTTCTAGTTAGT 1272240 1272360

NC_000963_1_Rickettsia_prowazekii_str__Madrid_E_chromosome_complete_genome probe_cstm_Rprow_021218_1x_106030 CATTTAAACCAACTTTTACACCTTCAGGTATAGTAATCGGCAATTTTCCAACACGTGACATTTTTTACCTTAAAATACTTTACAAATTACTTCACCACCAACATTTTTAATATGAGCTTC 813120 813240

NC_000963_1_Rickettsia_prowazekii_str__Madrid_E_chromosome_complete_genome probe_cstm_Rprow_021218_1x_106031 TCTATCAGACATAACACCGTAAGGAGTAGAAAGAATATATATGCCCATATTATTATAATATCCTTTCAAATCTTTAATAGCAGAATATACCCTTTTTCCAGGCTTTGATACTCTATGAAT 813240 813360

NC_000963_1_Rickettsia_prowazekii_str__Madrid_E_chromosome_complete_genome probe_cstm_Rprow_021218_1x_106032 TTCGCATATAGAAGCCTCACCGTTTACAGAGTATTTTAAAGCTACTTCAGTATAACTGATATTAtttttttGAGTAGTTACATAATCTTTTATATAACCTTCTTTTTGCAATACATCTAA 813360 813480

NC_000963_1_Rickettsia_prowazekii_str__Madrid_E_chromosome_complete_genome probe_cstm_Rprow_021218_1x_106033 AATTGAAGTTTTAATTTTAGAACTTGGAAAAGAAACATTTATTAATTTACTTTTATAAGCATTTCTAATTCTAGTTAACATATCTGCTACATTATCAGTCATTGACATATTATTTACCTA 813480 813600

NC_000963_1_Rickettsia_prowazekii_str__Madrid_E_chromosome_complete_genome probe_cstm_Rprow_021218_1x_106034 TCCACTTTACCAACTTGCCTTAACTACACCAGGAACTAAACCTCTACCAATTAATTCCCTAAGCTTATTCCTTGATATACCAAATTTTCTTATAACACCCCTTGGTCTACCTGTAAGCTC 813600 813720

NC_000963_1_Rickettsia_prowazekii_str__Madrid_E_chromosome_complete_genome probe_cstm_Rprow_021218_1x_106035 ACATCTATTTCTTATTCTAGTAGATGATGAATTTCTAGGTAATTGTGCCAGTGCAATTATCAAAGGAAAACGCTGTTCTAATGAAAGACTTTTATCATAAATTTTACTTTTTAGTTCTGA 813720 813840

NC_000963_1_Rickettsia_prowazekii_str__Madrid_E_chromosome_complete_genome probe_cstm_Rprow_021218_1x_106036 CCGTTTATTATGTAAACTTTGTGATTTTTTCTGCCTACTTTTGTTCTTTTGTATAGAACTTACTTTTGCCATTATATATACTCtaaaaattaattataaaaaggtaaattaaatcctgat 813840 813960

NC_000963_1_Rickettsia_prowazekii_str__Madrid_E_chromosome_complete_genome probe_cstm_Rprow_021218_1x_106037 aataaaaaCTTACTTTCTTGATCTGTTTTAGCAGATGTAACGATTGTAATATCCATACCCCTTATTGTATCGATTTTATCGTAATTAATTTCAGGAAAAACTATCTGCTCTTTTAATCCA 813960 814080

NC_000963_1_Rickettsia_prowazekii_str__Madrid_E_chromosome_complete_genome probe_cstm_Rprow_021218_1x_106038 AAAGTAAAATTCCCTTTACCATCAAAACTCTTATAAGAAAAGCCACGAAATTCTTTAACACGAGGTAATGCAACAATTACTAACCTTTCTAAAAAATCATACATTCTATCTTTACGTAAT 814080 814200

NC_000963_1_Rickettsia_prowazekii_str__Madrid_E_chromosome_complete_genome probe_cstm_Rprow_021218_1x_106039 GTAACCTTGCATCCTATTTTCATATTTTCACGTAACTTAAAGGTTGCGATAGATTTTCTTGCTAAAGTTACAACTGGCTTTTGACCTGAAATTAAAGTAAGGTCATTTAGTGCATTATTA 814200 814320

NC_009488_1_Orientia_tsutsugamushi_Boryong_complete_genome probe_cstm_Rprow_021218_1x_10604 TAAATCATCTTAAACATGAAAGGTACTCGTATATTGTGATACATCATAATATGAAAGAGCTGAACGGCTAAGAGTAAGTAAGTTAAGTATACAAAAAGCCATTCAAGAGATTAAATATTA 1272360 1272480

NC_000963_1_Rickettsia_prowazekii_str__Madrid_E_chromosome_complete_genome probe_cstm_Rprow_021218_1x_106041 tGCTTATATAATTCTTTAAACCTTAACATTATTACCTACCTTCCCAATAATTTCCCCTGAtttttttGCAACTCTAACCTTAGAACCATCTTCTAAGAATTTAAAAGCTACTTTAGTAGG 814440 814560

NC_000963_1_Rickettsia_prowazekii_str__Madrid_E_chromosome_complete_genome probe_cstm_Rprow_021218_1x_106042 ATTACCAGTTTTTGGATCGATGTGTGCAATATTTGAGATATGTATAGGTGATTCCTGAGTTATTATCCCGCCTTCACTCATTTTATTAGGTTTAGTATGCTTTTTTACTAAATTTACTCC 814560 814680

NC_000963_1_Rickettsia_prowazekii_str__Madrid_E_chromosome_complete_genome probe_cstm_Rprow_021218_1x_106043 AGAAACAACTACTGTATTTTCTTCCGGAAAAACTTTTAATACCTTACCTTTTTTGCCTTTATACTTTCCAGTAATAATAACAACTTCATCACCtttttttACTTTTAATTTAATCATAAC 814680 814800

NC_000963_1_Rickettsia_prowazekii_str__Madrid_E_chromosome_complete_genome probe_cstm_Rprow_021218_1x_106044 ACTTCCTCTGCAAGCGACATTATTCTAACATATTTCTTTGCTCTAAGTTCTCTTGTAACAGGACCAAACACTCTGGTTCCTATAGGCTCATCTTGTTTATTCAAAAGTACTAATGCATTT 814800 814920

NC_000963_1_Rickettsia_prowazekii_str__Madrid_E_chromosome_complete_genome probe_cstm_Rprow_021218_1x_106045 TTATCAAATTTTATTGTACTACCGTCAGATCTGACTACTCCCGTCTTTGTACGCACGATCACACCTTTATAAACATCACCTTTTTTAACCTTGCCACCTGGTATCGCTTCTTTAATAGAT 814920 815040

NC_000963_1_Rickettsia_prowazekii_str__Madrid_E_chromosome_complete_genome probe_cstm_Rprow_021218_1x_106046 ACCACTATAACATCACCAAGCTTTGCCATCATATGATGAGAGCCACCTAAAACTTTAATACACATAACTTTTTTAGCACCAGAATTATCTGCCACTTCCAAAATGCTCTGCATTTGAATC 815040 815160

NC_000963_1_Rickettsia_prowazekii_str__Madrid_E_chromosome_complete_genome probe_cstm_Rprow_021218_1x_106047 ATAAACTACTTCCTTTTATACATGAGAGTTAAAATAAACAATTTACATTATTAAGTCAAAAGTTATTCCAAATTTAGCACTACCCATGTTTTTGTTTTTGAGATAGGACGACTTTCAACT 815160 815280

NC_000963_1_Rickettsia_prowazekii_str__Madrid_E_chromosome_complete_genome probe_cstm_Rprow_021218_1x_106049 ACTGTCTTATCAGTTTTTGAACTTATAACCACGCCTTGTAACACTCTTTTCGGCATTTTAATATTCCTCACTATTAGATCTTTTTGTTAATTCAGTTTTAATACGTGCTATAGACTTCTT 815400 815520

NC_000963_1_Rickettsia_prowazekii_str__Madrid_E_chromosome_complete_genome probe_cstm_Rprow_021218_1x_106050 GACTAACGAAAACCTACTAGTATTTTTTAACTCTCCTAAAGCTTGTTGAAATCTTAAATTAAATAACTCtttttttAAAAGATTAAGGTTTTTATAAAGCTCTTCTATTGTTTCAGTAGA 815520 815640

NC_000963_1_Rickettsia_prowazekii_str__Madrid_E_chromosome_complete_genome probe_cstm_Rprow_021218_1x_106051 CAACTTACTTCTTAATAATTTTAAATCATTCATAACGTCTCACTATCCTTGTTCTAACAGGTAATTTTGCACTTGCAAGTTCTAAAGCTCTAAGTGCAATATTTTCTTCTACTCCTTCAA 815640 815760

NC_000963_1_Rickettsia_prowazekii_str__Madrid_E_chromosome_complete_genome probe_cstm_Rprow_021218_1x_106052 TTTCAAACATAATTCTTCCAGGCGAAACTCTAACTGCAAAAAATTCAGGTGTACCTTTACCTTTACCCATTCTTACTTCAGCAGGTTTCTTAGAAACTGGCACATCCGGAAAAATTCTAA 815760 815880

NC_000963_1_Rickettsia_prowazekii_str__Madrid_E_chromosome_complete_genome probe_cstm_Rprow_021218_1x_106053 TCCATAACCTACCTTGCCTTTTCATACATCTAGTAGCAGCTTTTCTTCCTGCTTCTATTTGTCTTGCAGTAACACGCCAACCATCTATAGATTTAAGACCAAATGATCCAAAAGCAAGTG 815880 816000

NC_000963_1_Rickettsia_prowazekii_str__Madrid_E_chromosome_complete_genome probe_cstm_Rprow_021218_1x_106054 TCATACCTGCTTTTGCTTTTGACATAACTCTACCTTTATGAGCTTTTCTAAAtttttgtttttttGGAGCTAACATTTGAATACTTAAAATTAATTATATCTTTTATTTTCTTTATATTC 816000 816120

NC_000963_1_Rickettsia_prowazekii_str__Madrid_E_chromosome_complete_genome probe_cstm_Rprow_021218_1x_106055 ACCTTTATAAATCCACACTTTAACACCTATAACTCCATAAGTTGTTATAGCCTCAGCTGTTGAATAATCAATATCAGCTCTTAAAGTATGTAATGGCATTCTACCTTCTATATACCACTC 816120 816240

NC_000963_1_Rickettsia_prowazekii_str__Madrid_E_chromosome_complete_genome probe_cstm_Rprow_021218_1x_106056 GGTTCTAGCAATCTCAGCACCACCAAGGCGCCCTGAACAACTTACTCTTATACCTTGTCCACCTTGTTTAAATGAAGCCTGAATTGCTGTTTTCATAGCTTTTCTAAAAGAAACTCTTTT 816240 816360

NC_000963_1_Rickettsia_prowazekii_str__Madrid_E_chromosome_complete_genome probe_cstm_Rprow_021218_1x_106057 TTCAAGCTGTGCTGCTATAGTTTGAGCTACTATAGCAGCATCTATATTAAATTTTCTAACTTCATGAATATTGATATAAACTTCTTTTAAAAAAGTCATGTTTTCTATAGCtttttttAG 816360 816480

NC_000963_1_Rickettsia_prowazekii_str__Madrid_E_chromosome_complete_genome probe_cstm_Rprow_021218_1x_106058 CTTATCAATTTCAGTACCATTTTTACCTATAATAATATTTGGtttttttGCATTAATATTAATTATAATACTTTTATTAGAAGGACGTTCAATTAAAACTCGACTAATTTGAGCTTGATT 816480 816600

NC_000963_1_Rickettsia_prowazekii_str__Madrid_E_chromosome_complete_genome probe_cstm_Rprow_021218_1x_106059 AAACCACTTATTTATTAAATCTCTAATTTTAAGATCTTGTATAAAAAGAGTTTTATAATGTTTTTCTGCATATAAGATTGAATCCCAATCTTTAATTAAAGTAGGTCCAACTCTAAAACC 816600 816720

NC_009488_1_Orientia_tsutsugamushi_Boryong_complete_genome probe_cstm_Rprow_021218_1x_10606 TTCATAGTACCTAGAACTCACGAATATGCAGCAAAAGGCATGAGATGTTATGGTGTTTATAATTGACATCTATCAAAGAGAGAACTAATATTATAGGAATATTAGTAGATAAATCTCTGT 1272600 1272720

NC_000963_1_Rickettsia_prowazekii_str__Madrid_E_chromosome_complete_genome probe_cstm_Rprow_021218_1x_106060 ATGTGCACAAACTTTCTGCCCCATTTTAATTATCCTCTTTTTCTGTAACAGTTATATAAAGATTACTAAAAAACTTATTTATTCTAGTTGCTCTGCCTTTTGCTCTTGGCATAATCCTTT 816720 816840

NC_000963_1_Rickettsia_prowazekii_str__Madrid_E_chromosome_complete_genome probe_cstm_Rprow_021218_1x_106061 TCATTACTACTGACTTACCTACAGTAGCTTTAGTAATAATTAATCTATCTATGTCTAAACCTAAATTATTTTCAGCATTTGCAATAGCAGATTGTAGACAATCTTTTACAATTTTTGCAA 816840 816960

NC_000963_1_Rickettsia_prowazekii_str__Madrid_E_chromosome_complete_genome probe_cstm_Rprow_021218_1x_106062 TTCTTTTAGGAGAAAAAGTTAATTGTACTAATGCTTCAGATACTTTCATATTTCTAATAAAGGACGCAACTAAATTAAGTTTCCTTGGGCTTACTCTAATAGATTTAGCttttgccgtag 816960 817080

NC_000963_1_Rickettsia_prowazekii_str__Madrid_E_chromosome_complete_genome probe_cstm_Rprow_021218_1x_106064 CTTCATTTATATAAACCGGGATAAATTTATTGCCATTATGAACAGAAAATGTAAATCCTACAAAAATAGGTAAAATTGTTGATCTTCTAGACCAAGTTTTAATCATTTCAGATTTACCTG 817200 817320

NC_000963_1_Rickettsia_prowazekii_str__Madrid_E_chromosome_complete_genome probe_cstm_Rprow_021218_1x_106065 ATTTCATTAATTTTTGAACTTTCTTTATTAAATAACCATCCACAAAAGGCCCTTTCCATATTGAACGTGCCATACCAATACCTAATTAATTAAAAATCTATTTTCttttttttACAATAA 817320 817440

NC_000963_1_Rickettsia_prowazekii_str__Madrid_E_chromosome_complete_genome probe_cstm_Rprow_021218_1x_106066 ATTTTGAAGTGCGTTTATTTTTACGTGTCTTCTTACCTTTAGTTGAGAATCCCCAAGGAGTCACAGGATGACGACCTCCTGAAGTTTTTCCTTCACCACCTCCATGAGGGTGATCCACAG 817440 817560

NC_000963_1_Rickettsia_prowazekii_str__Madrid_E_chromosome_complete_genome probe_cstm_Rprow_021218_1x_106067 GATTCATTGCTACACCTCTAACATGTGGCCTCCAACCAAGCCATCTATTTCTACCTGCTTTACCTAAATTAATAtttttttGATCCGGATTAGATATACTACCTATAGTAGCTTTACAAT 817560 817680

NC_000963_1_Rickettsia_prowazekii_str__Madrid_E_chromosome_complete_genome probe_cstm_Rprow_021218_1x_106068 CTAAAGGTACTAACCTAAATTCACCTGATCTTAATTTAATCTGAGCGTACCCTGAATCTTTACCAACTAATTCTACTGAAGTACCTGCAGAACGTGCAATTTGTCCACCTTTACCAATTT 817680 817800

NC_000963_1_Rickettsia_prowazekii_str__Madrid_E_chromosome_complete_genome probe_cstm_Rprow_021218_1x_106069 TCATTTCAACATTATGTAAAGTAGTGCCGATAGGAATGGATTTTAAAGGCAAACAATTTCCTATTTTGATATCAGCAGCTTGACTTGAAATGACTCTATCACCTATAGATAATTTTTGCG 817800 817920

NC_009488_1_Orientia_tsutsugamushi_Boryong_complete_genome probe_cstm_Rprow_021218_1x_10607 AACAGTATCAATTTTTGACTGCAATGTTAATACTGCTATTTTTATTGGGTAGAACAGGATTTAATTTCAAGATTACCTAGTAATTCTGTAGTTATGATCTATAATGCAAGCTTTCATAAA 1272720 1272840

NC_000963_1_Rickettsia_prowazekii_str__Madrid_E_chromosome_complete_genome probe_cstm_Rprow_021218_1x_106070 GTGCTAAAATATAAGAATATTCTCCATCATCATATTTTATTAAAGCAATAAAAGCAGTCCTATTAGGATCATATTCTATTCTTTCAACAATAGCAGAAATATCTATTTTCTTTCTTTTAA 817920 818040

NC_000963_1_Rickettsia_prowazekii_str__Madrid_E_chromosome_complete_genome probe_cstm_Rprow_021218_1x_106071 AATCAATAACACGATATAATTTTTTGTGCCCACCACCTCTGTGCCAAGAAGTAATTCTTCCTTGCTGATTGCGTCCACCAGTTTTTGACATGCCTTTAGTCAAAGTTTTTAAAGGTCTAC 818040 818160

NC_000963_1_Rickettsia_prowazekii_str__Madrid_E_chromosome_complete_genome probe_cstm_Rprow_021218_1x_106072 CTTTCCATAAATTCGTTTTATCAACTTGCACTAGCTCTCTAAGAGAAGGAGTAATTGGATTAAAATTTTTTAAAGCCATTTATTTAATTCCTCCGGCATAATCTATATTATGATCTTTTT 818160 818280

NC_000963_1_Rickettsia_prowazekii_str__Madrid_E_chromosome_complete_genome probe_cstm_Rprow_021218_1x_106074 CTATAGCCCTTTTAAGGCTCAATTTTTTAGCAAACTTATTTACATAAAAAGTATATTTATTTTGTTCAGAAATACTTGTAGTTTTTTCCGTAATAATAGGTTTTCTAATTAAATCGTAGT 818400 818520

NC_000963_1_Rickettsia_prowazekii_str__Madrid_E_chromosome_complete_genome probe_cstm_Rprow_021218_1x_106075 ATTTATATACACTCATCTTAACCTCTCTTCTAAAAAGCTAACCGCTTCTTGTGATAAGAGTACATACTCATGTCGTATGATATCATAAACATTAGCTCCTATTTGTGGGACAATTAACGT 818520 818640

NC_000963_1_Rickettsia_prowazekii_str__Madrid_E_chromosome_complete_genome probe_cstm_Rprow_021218_1x_106076 ATTATAAATATTTTTTGTAGCTAAAGAAAAATTAATATCTACTTTATTTCCATCAATTACaaaaaaaCTCTGACCTTGAAATTTACTTAATAAATTTACAAGAATAGACGTTTTAGGCTT 818640 818760

NC_000963_1_Rickettsia_prowazekii_str__Madrid_E_chromosome_complete_genome probe_cstm_Rprow_021218_1x_106077 ATCTAACTTTAAAGAATCTATTATTAATAATTTCTCTTCAGCATATTTCTCAGATAAAGCATGGATTAAACCAAGTTTTCGTACTTTTTTAGGTAATTTTATTGCATGACTCCGTACCTT 818760 818880

NC_000963_1_Rickettsia_prowazekii_str__Madrid_E_chromosome_complete_genome probe_cstm_Rprow_021218_1x_106078 AGGTCCATGTGATATACCACCACCACGCATCTGTACAGACCTAAGAGAACCTTGTCGTGCATTACCTGTACCTTTTTGCTTAAAAGGtttttttGTTGTCCCTGATACTTCAGATACTGT 818880 819000

NC_000963_1_Rickettsia_prowazekii_str__Madrid_E_chromosome_complete_genome probe_cstm_Rprow_021218_1x_106079 TTTAGTTTTATGGTTACCAAACATTGCTTTAGCTCTTTGCCAATCGATAACCTGCTTTATTATATCATCTCTGATAAATTCAACAGCAAATATATCTTTATTTAAAGTAATCTCACCAAC 819000 819120

NC_009488_1_Orientia_tsutsugamushi_Boryong_complete_genome probe_cstm_Rprow_021218_1x_10608 CTTCCTCATTTAATAAAAACTATGATAGAAAAAGATGGATACATATTAGACTATTTACCTCCTTATTCTCATAATTTAAATTCTATTTCACAAAGTATATAACATAACTGTTTTATATAG 1272840 1272960

NC_000963_1_Rickettsia_prowazekii_str__Madrid_E_chromosome_complete_genome probe_cstm_Rprow_021218_1x_106080 TTCTTCATTAGCAAGACTTAATATTTTAGTTTTCATCTTTATTTAACCTTATGCAGTCTTATCTAAAACTTCTATATAAATCATTTAATATTGCATGACGCTTTGTTAATTTTAAATCCT 819120 819240

NC_000963_1_Rickettsia_prowazekii_str__Madrid_E_chromosome_complete_genome probe_cstm_Rprow_021218_1x_106081 AAATCTGAATAACATCTAAGAGATTATTATTAAATAAACTTACGATAATTTAATTATTTTGCACTATAAATCACTAAATATTTATAGTATTTATGTAATGTAAACACCTTATTTCGTaaa 819240 819360

NC_000963_1_Rickettsia_prowazekii_str__Madrid_E_chromosome_complete_genome probe_cstm_Rprow_021218_1x_106082 aaaaaGCTCTTGGAATCAAAACAATCTAATCAAATATTCATGCGATAGTTATTGCAGCCTTTTTTATTGCATCTTTTACTAAAAGATACGAATTTTTGTGACCAGGTATACTACCCTGAA 819360 819480

NC_000963_1_Rickettsia_prowazekii_str__Madrid_E_chromosome_complete_genome probe_cstm_Rprow_021218_1x_106083 TCATAATCAGCTTACGATTTGTATCAACAGCAAATATTTTTAAATTTTGAATAGTAACTTTATTACATCCCATATGACCAGCCAttttttttCCTTTAAAAACCTTTCCTGGATCTTGTC 819480 819600

NC_000963_1_Rickettsia_prowazekii_str__Madrid_E_chromosome_complete_genome probe_cstm_Rprow_021218_1x_106084 TTTGGCCTGTAGAACCATGTGAACGATGTGATATAGAAACACCATGTGAAGCCTCAAGACCTCTAAAATTATGTCTTTTCATACTACCGGCAAACCCTTTACCTATAGTAGTCGCAGTAA 819600 819720

NC_000963_1_Rickettsia_prowazekii_str__Madrid_E_chromosome_complete_genome probe_cstm_Rprow_021218_1x_106085 TATCAACAAATTGCCCCACCCTAAAATGGTCTACTTCTAGAATTGATGCGATATCAATAAAATTATCTTCAGAAATTCTAAATTCTTTTAATTTAGTTTTAGGAGCTATTTTAGCATTAG 819720 819840

NC_000963_1_Rickettsia_prowazekii_str__Madrid_E_chromosome_complete_genome probe_cstm_Rprow_021218_1x_106086 CAAAAACTTGTTTCATAGGTTTAGTAACTTTAGATATTTTTTGATCTTTTACACCTATAACCAAAGCATTATATCCATGTTTTGCTAGAGTTTTATGCCCAACTACTTGACAATCATCAA 819840 819960

NC_000963_1_Rickettsia_prowazekii_str__Madrid_E_chromosome_complete_genome probe_cstm_Rprow_021218_1x_106087 CTTTTACTAAAGTTAAAGAAATTCTTTCGCCTTTATCATTAAAAACACTAGTCATACCAACTTTTTGAGCAATTATACCTGTTCTCATTTATTCCCCACTTTCTAATTCAATCACTACAT 819960 820080

NC_000963_1_Rickettsia_prowazekii_str__Madrid_E_chromosome_complete_genome probe_cstm_Rprow_021218_1x_106088 CAACACCTGCTGCTAAATCAACTTTACTTAATGCATCAACAACTGCTGGATTTGGATCATCTATAACTAATAATCTTTTATGCTTTCTAATTTCAAATTGTTCTCTTGACTTTTTATGTA 820080 820200

NC_000963_1_Rickettsia_prowazekii_str__Madrid_E_chromosome_complete_genome probe_cstm_Rprow_021218_1x_106089 CATGAGGGGATCTATTGACAGTAAATCTTTCAATTTTTCTAGGTAAAGGAATAGGACCATTAATAGTAGCAAATGTTCTTTTAACAGCACTAACTATCTCTTTTGTAGCTTGGTCAAGAC 820200 820320

NC_009488_1_Orientia_tsutsugamushi_Boryong_complete_genome probe_cstm_Rprow_021218_1x_10609 ATTTAGCTATATAGCTCAAGATATGTATCTTATTTTATAAGATACATAATATACTATTGACAATTTTAGTAAAAAACTATCAATGTAAGGTTGAAAAAATGAAAGAAAGAAATTTTGTTT 1272960 1273080

NC_000963_1_Rickettsia_prowazekii_str__Madrid_E_chromosome_complete_genome probe_cstm_Rprow_021218_1x_106090 TACGGTGATCAAACGACTTTAAACGAATTTTGATTTTATTTTTCATTCATTAAACCTAATATTTAAACAGCAAGATGTTTTTCAGatattaaaatacaaatacctattattttttaataa 820320 820440

NC_000963_1_Rickettsia_prowazekii_str__Madrid_E_chromosome_complete_genome probe_cstm_Rprow_021218_1x_106091 taggtatttgtaaaataaattaattatttattttaGTTACTATACCGGCTCCTACTGTTCTACCACCTTCACGTATAGAGAATTTTAACCCTTCTTGCATAGCAATCGGCTTAATTAATT 820440 820560

NC_000963_1_Rickettsia_prowazekii_str__Madrid_E_chromosome_complete_genome probe_cstm_Rprow_021218_1x_106092 CTACTGAAAAAGTAGCATTATCTCCAGGCATAACCATCTGCTTATCAGAAGGCAATTTTATTGTGCCGGTAACATCTGTTGTTCTAAAATAGAACTGTGGGCGATAATCATTAGTAAATG 820560 820680

NC_000963_1_Rickettsia_prowazekii_str__Madrid_E_chromosome_complete_genome probe_cstm_Rprow_021218_1x_106093 GGGTATGACGTCCACCTTCCTCTTTACTAAGCACATACACTTCAGCTTCAAATTTATCATGCGGTTTTATGCTCCCAGGTTTTGCAAGTACTTGTCCTCTTTCTACTTCTTCTCTTTTTG 820680 820800

NC_000963_1_Rickettsia_prowazekii_str__Madrid_E_chromosome_complete_genome probe_cstm_Rprow_021218_1x_106094 TACCACGTAGTAATATACCGACATTATCTCCAGATTGTCCTTCATCAAGTAATTTTCTGAACATTTCTACACCTGTACAAGTCGTTTTTTGCGTATTTTTTAGACCTACTATTTCAATTT 820800 820920

NC_000963_1_Rickettsia_prowazekii_str__Madrid_E_chromosome_complete_genome probe_cstm_Rprow_021218_1x_106095 CTTCACCCACCTTAATTATGCCTGACTCCACTCTACCAGTTACAACGGTACCTCTGCCTGAAATAGAAAATACATCCTCTATTGGCATTAAAAAAGGTTTATCTGTAGCTCTTATAGGCT 820920 821040

NC_000963_1_Rickettsia_prowazekii_str__Madrid_E_chromosome_complete_genome probe_cstm_Rprow_021218_1x_106096 GAGGTATATAGCTATCTACTGCATTCATTAACTCATTAATAGCTTTTTCACCTTCAGGTTTTCCTTCTAAAGCTTGAAGTGCAGAACCTTTAATAATAGGTATTTCATTACCAGGGAAAC 821040 821160

NC_000963_1_Rickettsia_prowazekii_str__Madrid_E_chromosome_complete_genome probe_cstm_Rprow_021218_1x_106097 CATATTTTGATAATAATTCTCTTACTTCCATCTCAACTAATTCTAATAGGTCAGGATCATCTACCATATCTACTTTATTCAAAAATACTACCATAGCAGGTACACCTACCTGTTTTGCCA 821160 821280

NC_000963_1_Rickettsia_prowazekii_str__Madrid_E_chromosome_complete_genome probe_cstm_Rprow_021218_1x_106098 GTAATATATGTTCTCTAGTTTGAGGCATAGGACCATCAGCAGCAGAAACTACTAATATAGCACCATCCATCTGAGCGGCACCAGTTATCATATTCTTGACATAATCAGCATGTCCAGGAC 821280 821400

NC_000963_1_Rickettsia_prowazekii_str__Madrid_E_chromosome_complete_genome probe_cstm_Rprow_021218_1x_106099 AATCTACGTGTGCATAGTGCCTATTTTGAGTCTCATATTCTACGTGCGCAGTAGAAATAGTAATACCTCTTTCTTTTTCTTCAGGAGCAGCATCAATTTGATCATACGCTGTAGCTTTTG 821400 821520

NC_009488_1_Orientia_tsutsugamushi_Boryong_complete_genome probe_cstm_Rprow_021218_1x_10610 ATGAATGGTTGGAACAACATGACTGATAGTACTACTAGTAACATAACGTGTAATTGTAGTCAATCTTGTCAAGAAAATATATACGGTTCGTATTGTCAAGGAATTGATATCTGGCTTGCT 1273080 1273200

NC_000963_1_Rickettsia_prowazekii_str__Madrid_E_chromosome_complete_genome probe_cstm_Rprow_021218_1x_106100 CTCCACCTGTTTTAGCAAGTATTATAGTTATTGCTGCGGTTAAGGAAGTTTTACCGTGGTCTACGTGACCAATCGTACCTATATTAACATGCGGTTTAGTACGTTCAAATTTTGCTTTTG 821520 821640

NC_000963_1_Rickettsia_prowazekii_str__Madrid_E_chromosome_complete_genome probe_cstm_Rprow_021218_1x_106101 CCATATTATTACTCTCTACAATTTTTAGGTTTAATACAAGTTATTAGTGCAttttttttGCTAAATTTATTTGTTACCACTATCTCTGGAGCGGGTGATGGGAATCGAACCCACACAGCC 821640 821760

NC_000963_1_Rickettsia_prowazekii_str__Madrid_E_chromosome_complete_genome probe_cstm_Rprow_021218_1x_106102 AGCTTGGAAGGCTGGAACTCTACCATTGAGCTACACCCGCATTGGACACTATTAAATATATAACAGTGCTATATCATTTTTCAGATTATAAAAAATAACATATAAATCTATCTTTTAAAT 821760 821880

NC_000963_1_Rickettsia_prowazekii_str__Madrid_E_chromosome_complete_genome probe_cstm_Rprow_021218_1x_106103 AGATATATTTCCCTGAACCATTAAGTGGTGGAGGGAGAAGGATTCGAACCTTCGAACGCTTACGCGGGCAGATTTACAGTCTGCTGCCATTGACCACTCGGCCACCCCTCCTAAAGAACT 821880 822000

NC_000963_1_Rickettsia_prowazekii_str__Madrid_E_chromosome_complete_genome probe_cstm_Rprow_021218_1x_106104 TTACTGTATTAAATAGAGTAATTCACTTTAAAATACAAATATAGTATTAGGACTTAATAAACTTTGCCGAATCATCAAATAAACAAATAGCAGCTTGCACATTTATTTAATTTAGTTGTA 822000 822120

NC_000963_1_Rickettsia_prowazekii_str__Madrid_E_chromosome_complete_genome probe_cstm_Rprow_021218_1x_106105 ACTTTCTATAATAAATAGTAATTTCTGTCAAGAACAAATTAATGACGGTTTTCAATAAGATGCAAAATAAACAGCTTGATTATAAAAATAGTTATTATATGTATGGGAAACATCCTGTGC 822120 822240

NC_000963_1_Rickettsia_prowazekii_str__Madrid_E_chromosome_complete_genome probe_cstm_Rprow_021218_1x_106106 TTTCTGCTATCAATAATCCAAAACGTCAAATTGAAAATATTTTATGCACTAAGGAATTTTTTGATATAAATAAAAAATTAATAGGTACTAGATCTTATGAAATTGTTAATAACTATATTT 822240 822360

NC_000963_1_Rickettsia_prowazekii_str__Madrid_E_chromosome_complete_genome probe_cstm_Rprow_021218_1x_106107 TATCTAAATTACTGAAACACCAAACACATCAAGGCATAGCTGCAAAAGTAAGACCAATTTTTTCTTATAATCTAGACGACATAGATATTAATAAACCACAATGTAAAATTGTGATTTTAG 822360 822480

NC_000963_1_Rickettsia_prowazekii_str__Madrid_E_chromosome_complete_genome probe_cstm_Rprow_021218_1x_106108 ATCAAATCACAGATACACAAAATATTGGAGCAATTATTCGAAGTGCAGCTGCTTTTTATATAAATGCTATAATATTACCTCAAGATAATTCTCCAAATGAAAGCGGTAGCATTGCGAAAG 822480 822600

NC_000963_1_Rickettsia_prowazekii_str__Madrid_E_chromosome_complete_genome probe_cstm_Rprow_021218_1x_106109 CAGCATGCGGCACTTTAGAATTAATACCTATCATTAAAGTGACTAACTTATGCTCATATATGAATTATCTTAAAAAACGTGGCTTTTGGATTATCGGAGTAACAGGTTATGCAAAGGAAT 822600 822720

NC_009488_1_Orientia_tsutsugamushi_Boryong_complete_genome probe_cstm_Rprow_021218_1x_10611 TTAGGATATACAGTGGCATTATTGATTTTATGTTTTATAGCATACTACTATAAGAATAAGTGTCAGGACCTAGAAAGAAAAATAAAACATATACCTCAATCTTTAAATGTTATGCCTGGT 1273200 1273320

NC_000963_1_Rickettsia_prowazekii_str__Madrid_E_chromosome_complete_genome probe_cstm_Rprow_021218_1x_106110 ACTTTACTGATAAATTAATTTCTGACAAAATAGCATTAGTATTTGGGGCGGAAGATAAAGGTATGCGAAGATTAGTTAAAGAAACATGTGATTATTTAGCTAAAATACCTATTTCGAAGA 822720 822840

NC_000963_1_Rickettsia_prowazekii_str__Madrid_E_chromosome_complete_genome probe_cstm_Rprow_021218_1x_106111 GTGTAGAAAGTCTTAATGTTGCAAATGCTGCTTCTATAATTTTTCATTCATTACATAAATATTTTTAATAATTCAATAACTATTATTGAGAATTGTGCAAAGTAATTTAGACAAATCTCT 822840 822960

NC_000963_1_Rickettsia_prowazekii_str__Madrid_E_chromosome_complete_genome probe_cstm_Rprow_021218_1x_106112 AAATGTACTTAGAGAAGAAGCAATAACAACACCTAACTTGAAACAATTTCTTTGATATATCAAGGTAAGTATTAATTACTAAACCTATATATAGAATAATCTAACGTACATTAGCAAAAA 822960 823080

NC_000963_1_Rickettsia_prowazekii_str__Madrid_E_chromosome_complete_genome probe_cstm_Rprow_021218_1x_106113 TTTTATAGTTTTAATCTTAATCTTATGATAAATAAGTATATCTAATCTTTATTGCTAAGAATCTAAAAAGCTTCTTTAACAGAGAATATTACAGCTTTGCtaaattaagcaaaaaattag 823080 823200

NC_000963_1_Rickettsia_prowazekii_str__Madrid_E_chromosome_complete_genome probe_cstm_Rprow_021218_1x_106116 TGGTGATTTAGAATATAAAATAGCTACAAGTATAGATAAAGCAATTGATAGAATCCTAGCAGGCGAATTTGAAGATAATTTTCCTTTAGTAGTTTGGCAAACAGGGTCAGGTACACAAAC 823440 823560

NC_000963_1_Rickettsia_prowazekii_str__Madrid_E_chromosome_complete_genome probe_cstm_Rprow_021218_1x_106117 AAACATGAATATGAACGAGGTTATTGCATCTATTGCTAATGAAGAATTAACTGGTAAGAAAGGTGGTAAATTCCCAGTACATCCTAACGATCATGTTAATAAAGGGCAATCATCCAACGA 823560 823680

NC_000963_1_Rickettsia_prowazekii_str__Madrid_E_chromosome_complete_genome probe_cstm_Rprow_021218_1x_106118 CTCTTTTCCAACAGCTATGCATATAGCAACTGTACTTGCAACTAAACAGCAGCTTATACCAGCTTTGAATAATTTACTTACATATTTGCAAGACAAGTCAAAAGATTGGGATAAAATCAT 823680 823800

NC_000963_1_Rickettsia_prowazekii_str__Madrid_E_chromosome_complete_genome probe_cstm_Rprow_021218_1x_106119 AAAAATAGGACGCACTCACTTGCAGGATGCAACTCCTTTAACACTTAAACAGGAATTCTCAGGATATATTACACAAATAGAGTATGCTTTAGAACGCATAGAAGATGCACTaaaaaaaGT 823800 823920

NC_009488_1_Orientia_tsutsugamushi_Boryong_complete_genome probe_cstm_Rprow_021218_1x_10612 ACTCAAGGTGATTATTATTTTAGTGCTGCAGAAGAAGTAATATCTGAATGTCGTGTTTCTAGTTTAGAATTAAATAATGTACTTAGCCCACGTAATTACAACTGCGAAGAATCTCTTCAG 1273320 1273440

NC_000963_1_Rickettsia_prowazekii_str__Madrid_E_chromosome_complete_genome probe_cstm_Rprow_021218_1x_106120 TTATCTACTTGCTCAAGGCGGAACAGCAGTCGGTACAGGTATTAATTCAAAAATAGGCTTTGATATAAAATTTGCTCAAAAAGTTGCAGAGTTTACTCAACAACCATTTAAAACTGCTCC 823920 824040

NC_000963_1_Rickettsia_prowazekii_str__Madrid_E_chromosome_complete_genome probe_cstm_Rprow_021218_1x_106121 TAATAAATTTGAAAGTTTAGCAGCACATGATGCACTAGTAGAATTTTCTGGAACTCTTAATACTATAGCAGTTAGCTTAATGAAAATAGCAAATGATATAAGATTGCTCGGCTCCGGTCC 824040 824160

NC_000963_1_Rickettsia_prowazekii_str__Madrid_E_chromosome_complete_genome probe_cstm_Rprow_021218_1x_106122 AAGATGCGGTCTTGGTGAACTGCATTTACCAGAAAACGAACCTGGTTCTTCTATTATGCCAGGTAAAGTAAACCCTACACAAGTTGAAGCATTAACAATGGTGTGCACTCAGGTGATGGG 824160 824280

NC_000963_1_Rickettsia_prowazekii_str__Madrid_E_chromosome_complete_genome probe_cstm_Rprow_021218_1x_106123 TAATCATGTGACTGTGACTATTGCTGGCTCAAATGGGCATCTTGAACTTAACGTATTTAAACCAGTGATAATATACAATATCCTACAATCTATAGAGCTATTATCTGATAGCGTAAATAG 824280 824400

NC_000963_1_Rickettsia_prowazekii_str__Madrid_E_chromosome_complete_genome probe_cstm_Rprow_021218_1x_106124 TTTTGTTACACACTGTGTTAAAGGATTAGAGCCCAATATAGCACGCATTAATACTTTACGTGATAAATCTCTAATGCTTGTTACAGTTCTTAATCCACATATAGGCTATGATAATGCTGC 824400 824520

NC_000963_1_Rickettsia_prowazekii_str__Madrid_E_chromosome_complete_genome probe_cstm_Rprow_021218_1x_106125 AAAAATTGCTAAAGAAGCACATAAGTATGGGATCACTTTAAAAGAAGCAGCTAAAAAACTCAATTTTTTATCAGAAGAAGAATTTGATAAAATAGTAGTACCTGAAAAAATGATTTCCTA 824520 824640

NC_000963_1_Rickettsia_prowazekii_str__Madrid_E_chromosome_complete_genome probe_cstm_Rprow_021218_1x_106126 ATCTTAATATTAGATTAGAGACTAAAAATATCTTTTAATGTTAATATAGGATAAAAACGCATTTTATGTATATACGACTTGTAACAAATTGATAATTTGTGAGTATAATATGCTACAGCG 824640 824760

NC_000963_1_Rickettsia_prowazekii_str__Madrid_E_chromosome_complete_genome probe_cstm_Rprow_021218_1x_106127 CTTTCACCAAATAATAAAAAACCCATGTTACTACAGCAATCCAGCAATACATAAGTATATACAATAAATTTGTAAAATTAATAACTCGATTTATTAAGCTTTGTTATGAACTCTTTTAGC 824760 824880

NC_000963_1_Rickettsia_prowazekii_str__Madrid_E_chromosome_complete_genome probe_cstm_Rprow_021218_1x_106128 TAGGAAAGATATAGAAATTTTTTCAACTTTTTCTAGGCAATAATTGATATATTAATTAAATTTTTCTTCACATAGCCTTTATATTTGATTATACTTAAAACATTAATGATATAAAAATTA 824880 825000

NC_000963_1_Rickettsia_prowazekii_str__Madrid_E_chromosome_complete_genome probe_cstm_Rprow_021218_1x_106129 TAATGGTTTTAAATATAAAAGCTCCTGAAAATATAGTATTAAAGCCGACCATTACTGTTTTTGGTGTCGGAGGTGCAGGTAGTAATGCAGTAAATAATATGATTCATGCTAATCTACAAG 825000 825120

NC_009488_1_Orientia_tsutsugamushi_Boryong_complete_genome probe_cstm_Rprow_021218_1x_10613 CAAAATCTTAGCTCTAATAATCAAAATAACAATGAAGAGCATATTATTGTTCAGCAAAATCCTACTTAtttttttAGAGGCATCAATGCAAATTCTTGCACCTCTATTGATAATAATTTG 1273440 1273560

NC_000963_1_Rickettsia_prowazekii_str__Madrid_E_chromosome_complete_genome probe_cstm_Rprow_021218_1x_106130 GTGCTAATTTTGTAGTAGCGAATACTGATGCACAATCCCTTGAGCATTCTTTATGCATTAACAAAATACAATTAGGCGTTTCTACTACTAGAGGTCTTGGTGCAGGTGCATCACCTGAGG 825120 825240

NC_000963_1_Rickettsia_prowazekii_str__Madrid_E_chromosome_complete_genome probe_cstm_Rprow_021218_1x_106131 TTGGCGCGCTTGCTGCTCAAGAATCAGAAAATGAAATTCGTAGTTCACTAGAAAATAGCAATATGGTATTTATTACAGCAGGTATGGGAGGTGGTACAGGTACTGGGTCTGCACCTATTA 825240 825360

NC_000963_1_Rickettsia_prowazekii_str__Madrid_E_chromosome_complete_genome probe_cstm_Rprow_021218_1x_106132 TTGCACGCATTGCGAAAGAACTAGGCATCCTTACGGTTGGGGTAGTGACTAAACCTTTCCACTTTGAAGGAGGTCATCGTATGAAAACAGCTGATAAAGGACTTATTGAACTACAGCAAT 825360 825480

NC_000963_1_Rickettsia_prowazekii_str__Madrid_E_chromosome_complete_genome probe_cstm_Rprow_021218_1x_106133 TCGTTGATACTTTAATTGTAATACCGAACCAAAATCTATTCCGTATCGCTAACGAACAAACAACATTTGCTGATGCTTTCAAAATGGCAGATGACGTATTACATGCAGGTGTTAGAGGAG 825480 825600

NC_000963_1_Rickettsia_prowazekii_str__Madrid_E_chromosome_complete_genome probe_cstm_Rprow_021218_1x_106134 TAACAGATTTAATGATTATGCCTGGACTGATTAATCTTGATTTTGCTGACATTAAAGCAGTAATGAGCGAAATGGGTAAAGCAATGATGGGTACAGGTGAAGATAGTGGGGAAGATAGAG 825600 825720

NC_000963_1_Rickettsia_prowazekii_str__Madrid_E_chromosome_complete_genome probe_cstm_Rprow_021218_1x_106135 CTATTAAAGCAGCAGAATCTGCAATTTCAAATCCATTGCTAGATCATAGCTCAATGTGCGGTGCGAGAGGTGTATTAATCAATATTACTGGCGGTCCTGACATGACATTATTTGAAGTGG 825720 825840

NC_000963_1_Rickettsia_prowazekii_str__Madrid_E_chromosome_complete_genome probe_cstm_Rprow_021218_1x_106136 ATAACGCTGCTAATAGAATTAGAGAAGAAGTAGATAACATTGATGCTAATATTATTTTTGGTTCAACATTTAATCCAGAACTCAAAGGGATTATCAGAGTATCAGTTGTTGCGACAGGTA 825840 825960

NC_000963_1_Rickettsia_prowazekii_str__Madrid_E_chromosome_complete_genome probe_cstm_Rprow_021218_1x_106137 TTGATGCCGATAAAGTACCAAAATATAAACTCGCAATAGATAAAAATACCAATACACTTCCTGAAGAAACTTACAATGAGTCTATAATACAACATACACAAATAGAAACAATTCCTTCGT 825960 826080

NC_000963_1_Rickettsia_prowazekii_str__Madrid_E_chromosome_complete_genome probe_cstm_Rprow_021218_1x_106138 TTAATAGCTACAGTACTGAAAATATTGAAATTAATGAATCTTCAATAAAGCAAGATTATACTGGAAATGAACAAGAACTACGATTACATGTAAATGCTGTCAACAAACCTGAAAATAACT 826080 826200

NC_000963_1_Rickettsia_prowazekii_str__Madrid_E_chromosome_complete_genome probe_cstm_Rprow_021218_1x_106139 CACAGAAATCATCATTTTTAGGGAAGATATGGGAGTCGCTACGTACTTCAAATAATCAAACTTTAGAACGCAAAAATGTTATTGTGAATACTGTAGATCAAGATAATAAAGAATCTGATA 826200 826320

NC_009488_1_Orientia_tsutsugamushi_Boryong_complete_genome probe_cstm_Rprow_021218_1x_10614 TTTTCTATAGATGATAGTTCTATGGTTGGAGCAACAGGTAGTATCATGATGGGGACACTTACTGGTAATGTAATGGATGACTTAGAATAATAAATCTGTTTTAGTTTGCCTAGAATAAGA 1273560 1273680

NC_000963_1_Rickettsia_prowazekii_str__Madrid_E_chromosome_complete_genome probe_cstm_Rprow_021218_1x_106140 TTCATGACATACCAGCGTTTTTAAGAAAGAAACGTGATTAGATTTACCAATGAAGAATACAACTTCCAAATAATATAAAAAGCATTATTCTATAGCATCACTCATCGATTATGTAGATAC 826320 826440

NC_000963_1_Rickettsia_prowazekii_str__Madrid_E_chromosome_complete_genome probe_cstm_Rprow_021218_1x_106141 AAAAATTTATGTTTTATCTTGATTATATCAAAGATATTTAAAGTTCTTGAGCTATACAAATCAAATTGATACTTTTAAATCTTAAGAAGATACATAAAAAATTTTTAGGTATCAGTAGAT 826440 826560

NC_000963_1_Rickettsia_prowazekii_str__Madrid_E_chromosome_complete_genome probe_cstm_Rprow_021218_1x_106142 ATTAGAATATAGGCTTTAGATATTAATAGAATAATACATTATGCCAAATTAGATTTTTAAAGGAATCCAATCAAAATAATGTTATAAAATAAGCTTTAGCACAAACTCTCTTATTTAATA 826560 826680

NC_000963_1_Rickettsia_prowazekii_str__Madrid_E_chromosome_complete_genome probe_cstm_Rprow_021218_1x_106143 GAAACGCTATCTAAtttttttAGTGATAAAAATATAAGTATAAAATTTGTGATGAACACGACAAATAATATATTGTCACACTATGTAAAAATAAGCCTAATTTCAGCTTTAAATTAGAGT 826680 826800

NC_000963_1_Rickettsia_prowazekii_str__Madrid_E_chromosome_complete_genome probe_cstm_Rprow_021218_1x_106144 ATTTAAGCTAAATTATTTTTTAAACAtttttttATAAAGATATTACTAAATAATAAACAGATAACAATTACTTAAAATCCTCTTCTACAGCTTTAACTTCTTGCACTTCTGGTATAAAAT 826800 826920

NC_000963_1_Rickettsia_prowazekii_str__Madrid_E_chromosome_complete_genome probe_cstm_Rprow_021218_1x_106145 GTTTCAGCATTGATTCAATGCCGTTTTTTAAGGTAATAGTAGAACTAGGACATCCAAGACATGCACCACGAAGTGCTAATTTCACTACACCGCTTTCAAAACCTTTATATATTATATCAC 826920 827040

NC_000963_1_Rickettsia_prowazekii_str__Madrid_E_chromosome_complete_genome probe_cstm_Rprow_021218_1x_106146 CACCATCTTGAGTAACAAATGGACGCACTCTAGTTTCAATAGTCTCGATGATTTGCTTTTCTATTTCTGAAAGCATATCAAGATTATGTTTTTCATCATCTATTTTAGTATTTTCGTTAA 827040 827160

NC_000963_1_Rickettsia_prowazekii_str__Madrid_E_chromosome_complete_genome probe_cstm_Rprow_021218_1x_106147 ATACAGGAAATCCTGAAATAAAATGATCCATAATAACCATTAAAATTTCAGGCTTTATAACTTGCCAATTACCTCGAGCTTGTTTAGTTACTGTGATAAAATCACTACCCAGAAAAACTG 827160 827280

NC_000963_1_Rickettsia_prowazekii_str__Madrid_E_chromosome_complete_genome probe_cstm_Rprow_021218_1x_106148 ATTTCACATTATTAATATGGAATAGCGATTCTGCAAGTGCGCTTCTTCCTTTAACTTCAGCAAGCTCACTAAAAAAGACCGGCTGATCAACACTTATTTCTTGTCCAGGAAAAAATTTTA 827280 827400

NC_000963_1_Rickettsia_prowazekii_str__Madrid_E_chromosome_complete_genome probe_cstm_Rprow_021218_1x_106149 TTGCATCAGGATTTGGAGTTTCTTCAGTTTGAATAAACATAATTACAGTAATAAATTTGTTGATAAATGATAATTTAGGTTATACACTATTTATAGTTTTAGGTAAATATAGAATTAATA 827400 827520

NC_000963_1_Rickettsia_prowazekii_str__Madrid_E_chromosome_complete_genome probe_cstm_Rprow_021218_1x_106150 TATAAGCAGGAATAAAACATAATCCTGCTACGATaaaaaaaTAACTCCATCCAAGATAAGTAGCTGCATATCCTGAGATACTACCTATCAATATAGTACCAACATTGGTAATAGATGTAA 827520 827640

NC_000963_1_Rickettsia_prowazekii_str__Madrid_E_chromosome_complete_genome probe_cstm_Rprow_021218_1x_106151 TTAAAGCAATTTGAGTTATGCAATATCTAGAACTACAACATTTTAGTTGATAAGaaaaaaaTGGTGACATAGTAAGCCCTTTAGTAAATTCTTGACAAAAGACAGCTATATAAAGACTTG 827640 827760

NC_000963_1_Rickettsia_prowazekii_str__Madrid_E_chromosome_complete_genome probe_cstm_Rprow_021218_1x_106152 TAATATCCTGATTCAAAAAATAAAGATATATGAAAGATAAACTAGATAAAGCATGATATATTAATACTCTCTTAAGTAAGTAAGAATATTCATATTTTCTACATAAAAATCCACCTATAA 827760 827880

NC_000963_1_Rickettsia_prowazekii_str__Madrid_E_chromosome_complete_genome probe_cstm_Rprow_021218_1x_106153 CGCCACCAAAGATAGTAGCACACATACCAAAGGCTTTATAACCAACAGCTAAATCTTGTTTAGTATAACCAATGTCGAGATAAAACATATTAGGCATAATTGAGAGAAAACTATCTTGAA 827880 828000

NC_000963_1_Rickettsia_prowazekii_str__Madrid_E_chromosome_complete_genome probe_cstm_Rprow_021218_1x_106154 GACGATATAAAAGCATGAAACTAATAATTACTATACATTTTGGTTTCTTTATAAAGTCATAAAAAGCATTTAAAAATCTATCAAAGTCATTATCAATAATCTTATCTTTAAATTTAAGCG 828000 828120

NC_000963_1_Rickettsia_prowazekii_str__Madrid_E_chromosome_complete_genome probe_cstm_Rprow_021218_1x_106155 GATAGAATATAATTAACAGTAACGACGGTATACATAAAATTGCCATAGTGCGATAAACATCCTGCCAAGATATGATAGTAGACAAATATAAAGCTCCAGAACCAGAAATAAGTATACCAA 828120 828240

NC_000963_1_Rickettsia_prowazekii_str__Madrid_E_chromosome_complete_genome probe_cstm_Rprow_021218_1x_106156 TTCTAAAACCAGTAGTACAAGCCGCTTCACTTATGCCCCAATTTTTATCGGTAATCAATAACATTTGTGAAGACTGAATTAACATATCATAAATAGATGaaaaaaacgctaaaactacta 828240 828360

NC_000963_1_Rickettsia_prowazekii_str__Madrid_E_chromosome_complete_genome probe_cstm_Rprow_021218_1x_106158 TAGAAAAACTTATTTTTTCAAGTAAAGGTCCCCATAAAAACTTAAATATGTGAATAAAATTAACTAAACCGAAAAGTCCAATAGTAATTTTATCAAAATCGGATTCTCGCAGCCAAAAAG 828480 828600

NC_009488_1_Orientia_tsutsugamushi_Boryong_complete_genome probe_cstm_Rprow_021218_1x_10616 GGGGTTCAACTCCCTTTATCGGCACCGCAATATCTTTCTGTTTGTAATCAGATTTCTGTAATATTAATTTCCTACTTAAATGACCAATAATTTACACAGTTACTACTTTGTTTCATGATT 1273800 1273920

NC_000963_1_Rickettsia_prowazekii_str__Madrid_E_chromosome_complete_genome probe_cstm_Rprow_021218_1x_106160 TATATAAAATGACTTAATTATAACAAAAAATTAGCAATGAAATGGTGAATAATATAGAGTTATGAATTAGATCAAATCGTACTACGATACCTGTTACAGTCAAAAGTTCTTCTACCACTA 828720 828840

NC_000963_1_Rickettsia_prowazekii_str__Madrid_E_chromosome_complete_genome probe_cstm_Rprow_021218_1x_106161 CTATCAAAAGAATTAAACTTCTTACTGTCACAAATGCTAGTTCTTTTACCAAATGATCTTTTACGtttttttttATCACTTCTGAACGCACTACGTGGTGTAGATTCACCTTTATTTACT 828840 828960

NC_000963_1_Rickettsia_prowazekii_str__Madrid_E_chromosome_complete_genome probe_cstm_Rprow_021218_1x_106162 AAACGATCAATTGCACGCCATCTAACAACATCATCAGGAGAAATAAAAGACAGTGCATGTCCGATAGCACCTGCTCTACCAGTTCTACCTATCCTATGTAAATAATCTTCAGGACACATA 828960 829080

NC_000963_1_Rickettsia_prowazekii_str__Madrid_E_chromosome_complete_genome probe_cstm_Rprow_021218_1x_106163 GGTAAATCATAATTAATAACATGCTGTGTATGAGGAATATCAAGTCCACGTGCTGCTACATCAGTTGCTACCATTATTCTATGATTTAACTTACGAAATGATAAAATTACTCTTTCACGT 829080 829200

NC_000963_1_Rickettsia_prowazekii_str__Madrid_E_chromosome_complete_genome probe_cstm_Rprow_021218_1x_106164 TGACTCTGACTTAAATCACCATGTATAGCTTCTGCTTTATGATTTTCATATTTTAACATTTTAGCCAATTGATCTGCAGAGCGTTTAGTCTTCACAAAAATAATTACCGATCCTACTCTA 829200 829320

NC_000963_1_Rickettsia_prowazekii_str__Madrid_E_chromosome_complete_genome probe_cstm_Rprow_021218_1x_106165 TTACCAAGTTGCTTAGTTAATTCACTAAATTTTTCTTTATTAGAAACATGCATTAATTCTTGTTTTATTTCTGCTGCTGCTTTATTAGTTGCGCCTACCATAATACGTACTGGATTGTTT 829320 829440

NC_000963_1_Rickettsia_prowazekii_str__Madrid_E_chromosome_complete_genome probe_cstm_Rprow_021218_1x_106166 AAATATTTTTGAGAAACAGCAATAATATGTTTTGGCATAGTCGCAGAAAACATTAAAACTTGTCTTTTTTCTGGCAAAAATTTATTAATTTCTTCTAGCTGTTCTTTCATGCCCATATCA 829440 829560

NC_000963_1_Rickettsia_prowazekii_str__Madrid_E_chromosome_complete_genome probe_cstm_Rprow_021218_1x_106167 AGCATTCTATCCATTTCATCCAGCACTATTATACCTATATGCTCAATTTTTAAACTTCTTCTATTTAAGTGCTCGATAATACGCCCTGGTGTACCGATAATAACTTTCGGAttttttttt 829560 829680

NC_000963_1_Rickettsia_prowazekii_str__Madrid_E_chromosome_complete_genome probe_cstm_Rprow_021218_1x_106168 AATTGCATAAATTGTTTAAACATAGGTTCACCACCTATTAAAACTGCACTATTCATTTTATAGGCTGTAGTTACTTTATTTAAAGTACTGTGTATTTGCGTTGCTAATTCTCTAGTCGGC 829680 829800

NC_000963_1_Rickettsia_prowazekii_str__Madrid_E_chromosome_complete_genome probe_cstm_Rprow_021218_1x_106169 ACGAGAATTAAAGCAGTAGTTTTGTTTTTAATAAATGCATCAATTAAAGGTAATAAATAAGCAAGGGTTTTTCCTGACCCTGTTTGGCTAGAAGCAAGTATATCTGCCCCAGACATTGCA 829800 829920

NC_009488_1_Orientia_tsutsugamushi_Boryong_complete_genome probe_cstm_Rprow_021218_1x_10617 AATTTTCTTTAACATGCATTCTATAGCATAGCTTATTTTGCTTCCATTATGTAATATCGAATAAGCGCAGTCAATAATATGACAATTAAGGTTTTGTTTAACTGTTAGAGGATATAAGAT 1273920 1274040

NC_000963_1_Rickettsia_prowazekii_str__Madrid_E_chromosome_complete_genome probe_cstm_Rprow_021218_1x_106170 ACTGGAATAGATTGCTTCTGTATTTCAGTTGGCTCAGTTATATTCATTGTCTCAAGAGCAATAATTAATTCTTTAGATAAATTAAAATTTTTCATTATATAATCCAATATAGTTTTAAAT 829920 830040

NC_000963_1_Rickettsia_prowazekii_str__Madrid_E_chromosome_complete_genome probe_cstm_Rprow_021218_1x_106171 CAAAATATGCATATTATTGATGAGAACTGCTTACATACTACATTGATTAATAAGAACAGGACATGTGCAATGCTCTCTCATAGAAATAAAGTAGAAATGATGATCCCATAAGATTTATTT 830040 830160

NC_000963_1_Rickettsia_prowazekii_str__Madrid_E_chromosome_complete_genome probe_cstm_Rprow_021218_1x_106172 ATATAAAAAATAAGTTTATAGTAAAATAACTACATAATAATTATCTTGAATCAAAAAATATGCTTAAACTAGATTACAAGATACCAACTAAATATATAAAACGATAATGAATAATGAAAC 830160 830280

NC_000963_1_Rickettsia_prowazekii_str__Madrid_E_chromosome_complete_genome probe_cstm_Rprow_021218_1x_106173 TATTTTAGAATCTTATTCAAACCAGCATGATATATGAAATTCAGGACATATACATGCCATTTACTATACTTATAGGTTATTATAAGACAAAAATTATTTACTATTTTAAGATAATTATAA 830280 830400

NC_000963_1_Rickettsia_prowazekii_str__Madrid_E_chromosome_complete_genome probe_cstm_Rprow_021218_1x_106174 GATACCACAAATTATAAACAATTTAGGATAACTGATTTTAGTATTATTTAACTCTGAGATTAACAGCATAGGCTTTGCCTTGTTTTTCTTCAATGTCAAAAATTACTTCTTGTCCTTCTT 830400 830520

NC_000963_1_Rickettsia_prowazekii_str__Madrid_E_chromosome_complete_genome probe_cstm_Rprow_021218_1x_106175 CAAGGCTATGTAAACCTGCTGCGTCTATAGCTGATTTATGTACAAATACATCTTTCCCGCCATTATCCTGTTCAATAAATCCAAAATTCTTTGTAGAATTATACCATTTAACTTTACCTA 830520 830640

NC_000963_1_Rickettsia_prowazekii_str__Madrid_E_chromosome_complete_genome probe_cstm_Rprow_021218_1x_106176 CTATATTTGTAGCCATAAAAACCTTTATTCGATAGAAATTACTTACTTGAACTTAAATACACACTTATAATAACTGAAACTCAAATTTTAAGCCTCACCTAATATAATTAGGTGATAAAT 830640 830760

NC_000963_1_Rickettsia_prowazekii_str__Madrid_E_chromosome_complete_genome probe_cstm_Rprow_021218_1x_106177 TCATTATGCAAAACTACCTTACCTTAAGATATTTGTCAATAAACATGTCAATAACATGCAATAGAAAGTATTACTTTCTCAATTTAATCTATCATATTATTACTAATTTGTCACTATTTA 830760 830880

NC_000963_1_Rickettsia_prowazekii_str__Madrid_E_chromosome_complete_genome probe_cstm_Rprow_021218_1x_106178 AGTTAATTATATAATAAATTAATGAGTAAATTCACTATTTTACTTAGCTTTACATATCAGACTAATAATTAACGATAAAAGTACTTGCttttttatattttttttATTAATCTCTGCACT 830880 831000

NC_000963_1_Rickettsia_prowazekii_str__Madrid_E_chromosome_complete_genome probe_cstm_Rprow_021218_1x_106179 GAGCAAATGAAGTCTTAATATCAATAATTGCCCGCGTGATGGAATGGTAGACATAACGGACTTAAAATCCGTGGAGCGTAAGCTCTTGCCGGTTCAAGTCCGGCCGCGGGTACCATTAAA 831000 831120

NC_009488_1_Orientia_tsutsugamushi_Boryong_complete_genome probe_cstm_Rprow_021218_1x_10618 TTTAGCTGCTTTAATTCCTTCTATTAAATTTGGATTAGTATTATTGATTAGATTAGTATAGTATTCATGTTGCCCTAAATCATAACCAAATTTTGTATTACGAGAAGTAATGGCATAACT 1274040 1274160

NC_000963_1_Rickettsia_prowazekii_str__Madrid_E_chromosome_complete_genome probe_cstm_Rprow_021218_1x_106180 AGCATATGTTAGACAAATTTACTAATGCATAAGATAGTAAACGCACTTCATCAAGAGTTTTATTCTCAGAATCCAACACAATAATTAAATGTAAATTCACAACTAAGTAGTAGCTTTAGC 831120 831240

NC_000963_1_Rickettsia_prowazekii_str__Madrid_E_chromosome_complete_genome probe_cstm_Rprow_021218_1x_106181 ATTGCTTCGATAAAACTTAATTATTATATGCTGCCATATCTAATATTATTGCTAAGTATTATCTTCTTCATACTCTTAATCTCTTCTCTAACAATTTAGTATTCACTCCTAATTAAAAAG 831240 831360

NC_000963_1_Rickettsia_prowazekii_str__Madrid_E_chromosome_complete_genome probe_cstm_Rprow_021218_1x_106182 TTTTAGATTCTTGAGTGAGGCATGGGACAAGATATTTTGCTCAAATACTATTTTTCATTTTTGCCACTACCTTTTTCTATAAACATCTTTAAAAGTACCTTATATTCCCAATTTCTATAT 831360 831480

NC_000963_1_Rickettsia_prowazekii_str__Madrid_E_chromosome_complete_genome probe_cstm_Rprow_021218_1x_106183 ACTCCCTTTTGAGTTTCAATCATTATATTCTACAGCTTTTAAATTAGTGAGCATCAATTTTTCCAATTTTAAAAATACTTTATTATTACTGATGTGCAGTATACAAATAATTCTTACGCA 831480 831600

NC_000963_1_Rickettsia_prowazekii_str__Madrid_E_chromosome_complete_genome probe_cstm_Rprow_021218_1x_106184 ATAATATGCTCGTAAAAATCGGGATAATATTTATTGTCTTATAATACAGTCCAAATTAACCCTTTTGTCTCTTATTAAGTACTTGTACTTTTTCTGTTAATACGTGTATGTGTATACTAA 831600 831720

NC_000963_1_Rickettsia_prowazekii_str__Madrid_E_chromosome_complete_genome probe_cstm_Rprow_021218_1x_106185 ACATGTTGAGTATTTAAGGAGGACTTTGCTATGGTCAAATACTATAAGATAACTGATTGGTGCAGTAGTTACATATTAACCACTTAAATAATTTCAGTGCTATACCAAAATGCACTGTTA 831720 831840

NC_000963_1_Rickettsia_prowazekii_str__Madrid_E_chromosome_complete_genome probe_cstm_Rprow_021218_1x_106186 TCTGTTATATTATACATAAAATTTTGATCTAAAATTCTAAATCTTTCTTAGAAGTTCTAATATTCCGGTTTCCCTTATAAAAAGTTATTTCTATAATGcttttttctatctttgttgagt 831840 831960

NC_000963_1_Rickettsia_prowazekii_str__Madrid_E_chromosome_complete_genome probe_cstm_Rprow_021218_1x_106188 ttttattttgataaaaatcatttaaTCGTATTGTTTGTAAATCTATTGAATAACATTCGTTATTATGATAAATAGCTTGCAACTGACAATAAAGCGCCTGAACTTAAACCTGATTATTTT 832080 832200

NC_000963_1_Rickettsia_prowazekii_str__Madrid_E_chromosome_complete_genome probe_cstm_Rprow_021218_1x_106189 AGAGAATTTTAGAGAATATGTAAGGTGTATATAGTTTTTAATTATTATATCGCAATTGAATCTTATGATATCTCTATTAAATAAATAGAAAACACTTTAAAACACATGTATAGCCTTTAT 832200 832320

NC_009488_1_Orientia_tsutsugamushi_Boryong_complete_genome probe_cstm_Rprow_021218_1x_10619 AGTTAATATTAGGTCTCCTATTACTCCAAATTGACTAATATCTGGGTTTGTACTGCCAGCTGCTCTAGCAAATGCCACAATTTCCTTTATTCCTTGAGTGATAATAGATGCTTTACAGTT 1274160 1274280

NC_000963_1_Rickettsia_prowazekii_str__Madrid_E_chromosome_complete_genome probe_cstm_Rprow_021218_1x_106190 TTAACAAAGATAAACAAATCTTTAGTCTTATAAAATTCAGAACCTGATAATACTCATACTATCAAATATGCACTATAAAAACTGTAATAACACTTGATCTATAAAATAACAAGTATACTG 832320 832440

NC_000963_1_Rickettsia_prowazekii_str__Madrid_E_chromosome_complete_genome probe_cstm_Rprow_021218_1x_106191 AAAAGGGAAAAGCAAGGAATATTAGATATGCAAAAATCTAAATATTATTTACTGGTGTTGTGCTAGTGCTTATATTTTATTATTTATGAATACATAAATATTGATTTACAATAAATTTTC 832440 832560

NC_000963_1_Rickettsia_prowazekii_str__Madrid_E_chromosome_complete_genome probe_cstm_Rprow_021218_1x_106192 ACAGCATATACCAAAACTAACTCTCTTCTAGATAACTAAGTATAATATAACTACATCCTCTACCATATCAATATTTATAATATTATCAAACCAATATGGTTTATAACCCATAAACAATCA 832560 832680

NC_000963_1_Rickettsia_prowazekii_str__Madrid_E_chromosome_complete_genome probe_cstm_Rprow_021218_1x_106193 GCACATTACCTCGATATTCTCACAATAATTAATTATGAAATGTGAAACCAAATTCATCGATTTCACCGGTGATTGTACCATACATATAACTCAGAACTACATCATTGATCTTTGCtttaa 832680 832800

NC_000963_1_Rickettsia_prowazekii_str__Madrid_E_chromosome_complete_genome probe_cstm_Rprow_021218_1x_106195 TTAATTATTTATGAAAACAATACATCAACTTTTTCATAGCAATATAGTAATGCAGTATAGCTTAAAAAGCAAAATACTAATGGTATATAATACTCTATCTTAATTTCATGTCTTATATGA 832920 833040

NC_000963_1_Rickettsia_prowazekii_str__Madrid_E_chromosome_complete_genome probe_cstm_Rprow_021218_1x_106198 TTTAATATCTAAAATGGGTATTATATATTCCACCCTATCTTTAACCTATAAAAACGCAACTTATCTGTTAGAAAATCATCAATCATTGATATTATATTAAATCACAACTAATGATTAAAA 833280 833400

NC_000963_1_Rickettsia_prowazekii_str__Madrid_E_chromosome_complete_genome probe_cstm_Rprow_021218_1x_106199 ATTGTTGACATATTTTAAGTATCTTAATATCGTAATTTACCTAAATGATATTTAAACTCTATCATTAATTTTTAATGAACTGAAGATAAGGTAACACATGAAAAATAAAGAAGAATCCAA 833400 833520

NC_009488_1_Orientia_tsutsugamushi_Boryong_complete_genome probe_cstm_Rprow_021218_1x_10620 TTGACCATATTGTTTAGCAATAATAATACCAGCAATGATAGCAATGATATTTTTAAATGCGCTTGCAACTTGAATAGTAATAATATCTTCAGTAGTACTAGTTATAACATTAGTACTATG 1274280 1274400

NC_000963_1_Rickettsia_prowazekii_str__Madrid_E_chromosome_complete_genome probe_cstm_Rprow_021218_1x_106200 TATATTACCTGAGTTTATAATTGATGAAGAGGAAAGAAAGCAATTAAAAGCTAAAAACTCTAAACAAGAACTTAAATTAATATTAATAACTTTTACAATCATTTTAACTAGCTTTTTAAC 833520 833640

NC_000963_1_Rickettsia_prowazekii_str__Madrid_E_chromosome_complete_genome probe_cstm_Rprow_021218_1x_106201 ATTTTGTTATTTCTTCTTTAACTATATAGAAGAGAAAGCAGCAGAGTATAAATTACAGCGAGAGGTAGAACCaaaaaaaaTAGATCAACTGAATGATTAAAGCTTATTATATCTGAGGTT 833640 833760

NC_000963_1_Rickettsia_prowazekii_str__Madrid_E_chromosome_complete_genome probe_cstm_Rprow_021218_1x_106202 ATGAGTAAACGAATTTAGACCGATAATTTAATTCtttttttCTGCAAATTCTAAGAATATTTTACAAAATGAATAACTATTTCTaaaaaaaTCTTATTAATGCTCACTGAAAAGCTTTTA 833760 833880

NC_000963_1_Rickettsia_prowazekii_str__Madrid_E_chromosome_complete_genome probe_cstm_Rprow_021218_1x_106203 ATTACTAATTAAATTTTATTTACTTATAACTTCATAGCAATTCTTAAATAATCCTTAGGGGTAAGATTTTCAGCGCGATCATTACAGTTGATTTTCAACTGAGTTAATACTTCATGTATA 833880 834000

NC_000963_1_Rickettsia_prowazekii_str__Madrid_E_chromosome_complete_genome probe_cstm_Rprow_021218_1x_106204 TTAGGTATGAGATTTCTTAGTGAAGATTTGATCATTTTACGTCGTCCTGCAAAAACAAGTTTTGTTATTTGCTCAACTTTATTTATCAAAGCAATAGACGGTGGATTTTCTAAAGGTATT 834000 834120

NC_000963_1_Rickettsia_prowazekii_str__Madrid_E_chromosome_complete_genome probe_cstm_Rprow_021218_1x_106205 ATTTTAACTATTGCAGAATATACTTTAGGATGTGGATAGAAAGCAGTAGGTGCGACATTAAAGCATTTTTCGACTTTAGCTAATATCTGACATATTACCGATAATCTGCCATATGCTTTA 834120 834240

NC_000963_1_Rickettsia_prowazekii_str__Madrid_E_chromosome_complete_genome probe_cstm_Rprow_021218_1x_106206 GTTGAAGGTATAGCACAAATACGTTCTACTACTTCCTTTTGCAACATTAATGTCATATTAGTAATTAGTTTCACTTCTTTCAGCAATCTAATCACTAACTCAGTACCTATATGATATGGT 834240 834360

NC_000963_1_Rickettsia_prowazekii_str__Madrid_E_chromosome_complete_genome probe_cstm_Rprow_021218_1x_106207 AAATTAGAAATTACAGTTACTTTATCATAGATTAAATCAGTTAAATTTATTTTAAGGACATCTTGTTTAATAATATTGAGATTAGGATAATATTCTTGAATCTCATTAAGTAGTGGTATG 834360 834480

NC_000963_1_Rickettsia_prowazekii_str__Madrid_E_chromosome_complete_genome probe_cstm_Rprow_021218_1x_106208 CAGCGCTCATCAATTTCTATCACGGTTAAGGATTTAGGATTTTTCTGCAAAATTGACCTAGTTAAACCTCCTATACCCGGCCCTATTTCTATCACATTACTATTTTCTAAAACATTACTA 834480 834600

NC_000963_1_Rickettsia_prowazekii_str__Madrid_E_chromosome_complete_genome probe_cstm_Rprow_021218_1x_106209 GCCCGTATAATTTTATCACATAGGCTACTATCAAAAATAAAATTTTGTCCATGTTTTTTCAGAGGATTAATTTTATGAGATGTAGCATGTTTTACGATCGAAGGAAGCATGGATTTATTT 834600 834720

NC_009488_1_Orientia_tsutsugamushi_Boryong_complete_genome probe_cstm_Rprow_021218_1x_10621 AAATAATGTAGAAATATTGAATTGCACTTCTTTTTGAATAGCAGCAATTGTCAGAGCACATGGCAAACCTTGGGCTACCTCATTTGCTAAATTTGGTCCAGCTATGATGAATAAAGGATT 1274400 1274520

NC_000963_1_Rickettsia_prowazekii_str__Madrid_E_chromosome_complete_genome probe_cstm_Rprow_021218_1x_106210 TAAACGTTTACATAAATAACAAAACTCATTATTAAATTGCAGAACCAAAATCATGCAATCCAGTATAAAAATAACTATTAATAATAAAACATTATCAAAAACCTAATATTGACACCTAAG 834720 834840

NC_000963_1_Rickettsia_prowazekii_str__Madrid_E_chromosome_complete_genome probe_cstm_Rprow_021218_1x_106212 CATTTAAGAtttttttACTGCACACTAATATTATTTCGAACTTGTTATTTACTTCAAAAACATTACTAGCTTTATCAGGTATTAAATCCTTTACAATAGTTTGTTTTACTCCTTCTATGT 834960 835080

NC_000963_1_Rickettsia_prowazekii_str__Madrid_E_chromosome_complete_genome probe_cstm_Rprow_021218_1x_106213 TACTAAGCTTACTGGTAATAATTTGCATAGTAGCAAATTTATCGTAAAGCGTTCTTTTGACATCAGAACAtttttttAACCGATTTTTTAAATGATTCATCTTCGTAAATGCTTTATTAC 835080 835200

NC_000963_1_Rickettsia_prowazekii_str__Madrid_E_chromosome_complete_genome probe_cstm_Rprow_021218_1x_106214 TACCATCTTTAGATCTGAATACTTGCATTAAAATTTCTACGTCTTTTTGATCGCTAGATAAAATTGCAACATCTATTTCTTTATTACTTACTTGTACTGATCGTGATAAACTTGATAAAA 835200 835320

NC_000963_1_Rickettsia_prowazekii_str__Madrid_E_chromosome_complete_genome probe_cstm_Rprow_021218_1x_106215 TATTCATTTTAATCAACTCAGATTTAATTTGCAAAATAAAACTTTCAGGATTCACAGATTTATTTTTTAGATACTGTAGCAGAGAACCATGAGCCATTTTATTACGATCTTCAATAGATT 835320 835440

NC_000963_1_Rickettsia_prowazekii_str__Madrid_E_chromosome_complete_genome probe_cstm_Rprow_021218_1x_106216 TAATAGCATTATCTATCTCGTCTTGCGGGATTTCTCTATCACCTGCATATTGAAAAAGTAAAGATTCATCAATTAAGCTGTTGATTGCTAAGTCATTCAGTTGCTTATCTTGATCAGCAG 835440 835560

NC_000963_1_Rickettsia_prowazekii_str__Madrid_E_chromosome_complete_genome probe_cstm_Rprow_021218_1x_106217 TTAAATTTTCAATATTATTTAATGCCATAATCAttttttttCTAGCATGGAACTCATTAAGAGTAATAGGTTTATCATTTACTGAGGCTACTATACTAGTCAATGATGCTTGTGCTACAT 835560 835680

NC_000963_1_Rickettsia_prowazekii_str__Madrid_E_chromosome_complete_genome probe_cstm_Rprow_021218_1x_106218 TAAAAGTGaaaaaaaCAGTAATGATTAATAATAATTTATGCATATTTCTCGACTCACATAACGTAATAATTTAAATAGATTTATATGGATACTAACTTATTGGAAATAACGCAATAATTA 835680 835800

NC_000963_1_Rickettsia_prowazekii_str__Madrid_E_chromosome_complete_genome probe_cstm_Rprow_021218_1x_106219 ACTAAGCAATCCAGTTCAAAATTTCTCAATAGCACTTTATATTACTTTTTCTGGATTTCTAAATTGCTTAACAATAATAACTATCCACACAAGCACAATGCTAAACTCACATATTCAAAA 835800 835920

NC_009488_1_Orientia_tsutsugamushi_Boryong_complete_genome probe_cstm_Rprow_021218_1x_10622 ATTTGCTAAATAATCTTTTACTACGTCACTTAATAGTTTAGATTGATTATGATCTAATCCTTTTGATGCAATTCCAATAATGGCATTATTAGATATGCTATGTTGCTTTAAATTTTCAAT 1274520 1274640

NC_000963_1_Rickettsia_prowazekii_str__Madrid_E_chromosome_complete_genome probe_cstm_Rprow_021218_1x_106220 CCTTTAAACTAACAGCAACAGTAGGGGAAGATGTAGTCTTTTTGATTCCTCTACTCCCATCAGCTAGATAATCGGAATAAATCTTTGTAGTAATTCTAACACAATCTTTAGCATATGTCA 835920 836040

NC_000963_1_Rickettsia_prowazekii_str__Madrid_E_chromosome_complete_genome probe_cstm_Rprow_021218_1x_106221 CTCTAATAGTTCGAGTAAATAGATTTGGAGAACGTCTAGAGAGATCAATGCGTGTCGCAAAGCCTATAGTCCAATTTTCTGCTAACTGATAATTTATGACTCCATAAAATTGTCGCACTC 836040 836160

NC_000963_1_Rickettsia_prowazekii_str__Madrid_E_chromosome_complete_genome probe_cstm_Rprow_021218_1x_106222 TATTATTTGATACTTTTATACTTTCTACAGAATAATATTTTTTAAGATTAGTAAGCTGAATAAAACTACCTAAAAAGTTAATCTTATTATAGTTAAAATTACCACCAACTTCATCTCTAA 836160 836280

NC_000963_1_Rickettsia_prowazekii_str__Madrid_E_chromosome_complete_genome probe_cstm_Rprow_021218_1x_106223 TAGGCCTAAAATATCTATCTTGCCGGAAGTTATAAAACACTTCTAAATTATTAGAAAGATTACCTAATATCTGCCCTACATTTTCTGTATCATCAGTAGGCAAATCTATACTATATCGTG 836280 836400

NC_000963_1_Rickettsia_prowazekii_str__Madrid_E_chromosome_complete_genome probe_cstm_Rprow_021218_1x_106224 TATTACGAGACTGTCCTAAAAATAATTTTAAATAATTATCTTCTTGAAGAATACTAGCATTTAAGCCATAACTTAATCTATTACCAAAATCATAACAATCAATACCGCTATAACGATTTG 836400 836520

NC_000963_1_Rickettsia_prowazekii_str__Madrid_E_chromosome_complete_genome probe_cstm_Rprow_021218_1x_106225 ATAAAAAGATATTTTTCTCTGATAATTCATACTTAACAGGATCAATTATAACAAATTGTTTATCTTGCTTAGATAATTTACGTCCAATACTAAAAGAAACAATCGGCTCAATAAGTAAAT 836520 836640

NC_000963_1_Rickettsia_prowazekii_str__Madrid_E_chromosome_complete_genome probe_cstm_Rprow_021218_1x_106226 TTATCGTTTTAATATTACCTACTAACGGATAACGCCATAAAGTTTGCAATTCCGGTATATTCCTTTGCAATGCTTGATTTTTTCTTTGCTTATCTAGATAAGAACTTCTAGCAAGATAAA 836640 836760

NC_000963_1_Rickettsia_prowazekii_str__Madrid_E_chromosome_complete_genome probe_cstm_Rprow_021218_1x_106227 AATCACCTCTATCTTTTGCTATAAAATTTAAAATTTGCCCTGAAGAAGTTAGTACATTATGCATAAATGATAATTGTAAAGATGTACGAGCCACTTGTTCTCCTATCCTTGCTTTATACA 836760 836880

NC_000963_1_Rickettsia_prowazekii_str__Madrid_E_chromosome_complete_genome probe_cstm_Rprow_021218_1x_106228 TTAACGTATTATTTTCAACAACGATATAGCTGTCACCATTATCATTAAGATGTATAACATTTTTTGTATTGATTTTTGGGAATACTAACGGATCAGTATATTTACTATCATTACTACCCA 836880 837000

NC_000963_1_Rickettsia_prowazekii_str__Madrid_E_chromosome_complete_genome probe_cstm_Rprow_021218_1x_106229 ATCCTTGAAAACTTAATACTTCTATCGCAAAATAATCAGCACCAAGAATTTTATATAAAAACATTTTAGAAGTTAAATATGAAGAGTAGTTATTATAATAATTTTTTAAATAAGCTTTAT 837000 837120

NC_009488_1_Orientia_tsutsugamushi_Boryong_complete_genome probe_cstm_Rprow_021218_1x_10623 AGTAGCTCTTACTTGATCTGATGGTGTAACAATAATAATTATTTCATGATCAACAATATCAGAAAAGTTGCTAGTAGCCTTAATGATATTTGGTAATATGATATTAGGTAGGTATTTTTT 1274640 1274760

NC_000963_1_Rickettsia_prowazekii_str__Madrid_E_chromosome_complete_genome probe_cstm_Rprow_021218_1x_106230 CTGATGTACGTTCTATTCTAAAACCGTAATCATAAGTTTGATCTCTACTAATAAAATTACCGCTTGCTATATAATGATAAGAAGAGACTTTTCTATTTTTTACTACACTTCCGTCTTTCT 837120 837240

NC_000963_1_Rickettsia_prowazekii_str__Madrid_E_chromosome_complete_genome probe_cstm_Rprow_021218_1x_106231 TAAGCAAATAAGGTAACTGCCCGTAACTACCTTGAAAGTTCATATTATCGGTATCATTCAGACGATAACGTGCTTCTAACTCATAAGTTTGATATTTACTAGCAATTCTTGGAGTAAGGG 837240 837360

NC_000963_1_Rickettsia_prowazekii_str__Madrid_E_chromosome_complete_genome probe_cstm_Rprow_021218_1x_106232 TAAAATCAATATTAGGTTTTGCACGCAGATATATTGGAATACCAAATCCTTTATTTTTAACATCAGGTACTAATAATCCCGAAGTTGCAGGTGCAGAAGGAGTCGGATGaaaaaaaTATG 837360 837480

NC_000963_1_Rickettsia_prowazekii_str__Madrid_E_chromosome_complete_genome probe_cstm_Rprow_021218_1x_106233 GCAAGTAAAAAATCGGTACACCATATACTTCAAAAAATACGTTTTTATAAACTATTCTATGCTCTGCAGACTTAATATAAGTATCATTAGCCGCAATTTGCCAGATAGGTTTGCTATTAC 837480 837600

NC_000963_1_Rickettsia_prowazekii_str__Madrid_E_chromosome_complete_genome probe_cstm_Rprow_021218_1x_106234 AAGTAACATCGCAAGGGGTAAATTCAGCACCATACAACTTAAGGTTATTTTCATCTATTCTTTCCGCAAGTTTAGCAATTAAGAGATTATGATCGCCGACAAGCAAAATGAATTCAGATA 837600 837720

NC_000963_1_Rickettsia_prowazekii_str__Madrid_E_chromosome_complete_genome probe_cstm_Rprow_021218_1x_106235 TAATACCTTTCTTAAATTCATTTTTTAAAACCGCTTTATCACCCTCTATTATTCTGTTTTGTTTATCCTTAATACGAATATTACCTTCTGCCCATAAAATATCATTTTTTACATCATAAA 837720 837840

NC_000963_1_Rickettsia_prowazekii_str__Madrid_E_chromosome_complete_genome probe_cstm_Rprow_021218_1x_106236 GTAAATTATTTGTTGTCAATAAATACTCATCAGTAATAATTTTTATATTACCTTTAGCATATATAAAATTCTTATTTTCATTATACTCTACGAACACGGAATCGGCAATTAGATTATATT 837840 837960

NC_000963_1_Rickettsia_prowazekii_str__Madrid_E_chromosome_complete_genome probe_cstm_Rprow_021218_1x_106237 TAACGCTTTGATTTTGCTTAAACTGTTGAGTAAAACTAGCTAGAGGTAATAGTACGATAATAATTAGGTATATTAGTCGCATTTATTATGTTATCTAAATATTCAATAGTTAAAAACTAT 837960 838080

NC_000963_1_Rickettsia_prowazekii_str__Madrid_E_chromosome_complete_genome probe_cstm_Rprow_021218_1x_106238 AGCAAGCTATTATTAATATAGCAAATATTTAAAATTTTACTTGCTCTTTTACAGTGGAAGGTTATTTTATGTATATCCACAACACAATCAATAGTATCTTAGACAAAACGTGTAATACAG 838080 838200

NC_000963_1_Rickettsia_prowazekii_str__Madrid_E_chromosome_complete_genome probe_cstm_Rprow_021218_1x_106239 ATTCCATGAATAAAGTCACGTGATGAAATAATATCAACATGATAGACAATTGCATTGCAAATCAAGCATCTAAAGAGTTCTCAGTTAGTGAGATTTCTAATAAAATTAAAGAATTATTAG 838200 838320

NC_009488_1_Orientia_tsutsugamushi_Boryong_complete_genome probe_cstm_Rprow_021218_1x_10624 ATTAGTATGCAGCTGATTGATTTCTTGAGTATGGGCTGAGTGTCTAGTATATAATGTTACTCTGTAATTATTACGAGCTAGAAGCATAGCTATCGCTGTTCCCCAAGCTCCAGCTCCTAT 1274760 1274880

NC_000963_1_Rickettsia_prowazekii_str__Madrid_E_chromosome_complete_genome probe_cstm_Rprow_021218_1x_106240 AAAATAATTTTGGCTATATTAAGGTAAAAGGAGAAATTTCTGGTTTAAAAAGAGCTAGTTCAGGTCATGCTTATTTTAACTTAAAAGAAAATACTGCCATTTTAGCTTGTACTTGTTGGC 838320 838440

NC_000963_1_Rickettsia_prowazekii_str__Madrid_E_chromosome_complete_genome probe_cstm_Rprow_021218_1x_106241 GCCCTATTCTTGCTAAAATAAAATTTCCTTTAAATGACGGCATGGAAGTAGTAATTGGTGGTAAACTCTCAAGTTACTCAGGTAATTCACGCTATCAATTATCGGTAGATAATTTGCAAC 838440 838560

NC_000963_1_Rickettsia_prowazekii_str__Madrid_E_chromosome_complete_genome probe_cstm_Rprow_021218_1x_106242 CTGCAGGACTTGGAGCTATGCTACAAATTCTTAATGAGCGCAAAACCCGTTTAGAAAAAGAAGGACTCTTTAATAAGAAACGTATTCCTATACCTTTTTTACCTGATAAAATAGGCGTTA 838560 838680

NC_000963_1_Rickettsia_prowazekii_str__Madrid_E_chromosome_complete_genome probe_cstm_Rprow_021218_1x_106243 TCACTTCAATAACCGGAGCTGTTATTAAAGATATTATTCATCGTATTCGTGAACGTTTTCCGACGCGGATAATAATATGGCAGGTTAGCGTGCAAGGGGAAAATTCCGGCCATGAAATGG 838680 838800

NC_000963_1_Rickettsia_prowazekii_str__Madrid_E_chromosome_complete_genome probe_cstm_Rprow_021218_1x_106244 CGGAAGCAATTGAAGGATTTAACAATTTAGAAGAGATACATAAACCAAGTGTGATAATTGTTGCTAGAGGTGGGGGTTCTATAGAAGATCTTTGGTCATTTAACGATGAGATATTAGTAC 838800 838920

NC_000963_1_Rickettsia_prowazekii_str__Madrid_E_chromosome_complete_genome probe_cstm_Rprow_021218_1x_106245 GTGCTGCTTATAACTCAAAAATTCCTATTATTTCTGCAGTAGGTCATGAAGCAGATTATACTTTAATAGATCTAGCAGCAGACAAAAGAGCCCCAACACCTACTGCTGCAGCAGAATTTG 838920 839040

NC_000963_1_Rickettsia_prowazekii_str__Madrid_E_chromosome_complete_genome probe_cstm_Rprow_021218_1x_106246 CTGTACCTGTACGATCTATTTTGAATAATACAATCCAATCTTATGAAAAAATATTATTTAATAATACTAACCGATTAATTAAATATCACGAACAAAGTATAGTAAATTACGATAAAATAC 839040 839160

NC_000963_1_Rickettsia_prowazekii_str__Madrid_E_chromosome_complete_genome probe_cstm_Rprow_021218_1x_106247 ACAGCTATTTCTCCTATTATATAAATAATAGGCAGCAATTACTAGACGAAATCGGTTTTAATTTGCTAGATGTTTTAATACGATTTATTGCTCTCAAAGAAACAAAAATTAAATCTTTTT 839160 839280

NC_000963_1_Rickettsia_prowazekii_str__Madrid_E_chromosome_complete_genome probe_cstm_Rprow_021218_1x_106248 CTAAAGAAAGGATCAATTATGCTAAAATCATCAATTACAAAATATTAGAATTAACACATCAAACAGCGTATCTATTCAAATCGGTAAATAATACCTTGAAAAACTTTGAATATAAACTAG 839280 839400

NC_000963_1_Rickettsia_prowazekii_str__Madrid_E_chromosome_complete_genome probe_cstm_Rprow_021218_1x_106249 AATTAAACAGTACATTACTTGCAAGCCTTGATTATCATAACGTATTAAAACGAGGTTTTGCTATAGTTAAAGGAGACGCAGGAAATTTCTTATCTTCTAAATCTGCCGCCACAAATGAGC 839400 839520

NC_009488_1_Orientia_tsutsugamushi_Boryong_complete_genome probe_cstm_Rprow_021218_1x_10625 TATTGCGATTTTCATATTTAAAATAATTTTAGTTATTGAAATAGTTTATTTGATTTTAAAAAGTATAAATTGTTGATTTAATTAACCACTGTTCCAGTCATATGATCATTATCAATACCT 1274880 1275000

NC_000963_1_Rickettsia_prowazekii_str__Madrid_E_chromosome_complete_genome probe_cstm_Rprow_021218_1x_106250 AGAGTCTAAATATTAAATTTTTTGATGGTGAAATTAATGTAGTATTATCATGCCATGACTTGAACACGAGATCCAATTAAGTTAAAATTATTTAGTAAAATATTGATTCTAAAAAATACA 839520 839640

NC_000963_1_Rickettsia_prowazekii_str__Madrid_E_chromosome_complete_genome probe_cstm_Rprow_021218_1x_106251 TACAATGTAGATTGTAAAGCTTTTTATTTTTATACTTCTAATTACTAAAAATTTTTATGCTTTAAGCATAGGCATAAATGGACAGCACAAAACTTTAAGATGCAGAAAAATATATTATAA 839640 839760

NC_000963_1_Rickettsia_prowazekii_str__Madrid_E_chromosome_complete_genome probe_cstm_Rprow_021218_1x_106252 CTAGTATCAATGTCAATTCGATATTCAAAGTACTGACATAACAGCAGGGCATTATTACTTCTGATAGAAGTAATAATGCTTATTTCGTCTTGAAGAGTTATCATTATTTATTATTGAATC 839760 839880

NC_000963_1_Rickettsia_prowazekii_str__Madrid_E_chromosome_complete_genome probe_cstm_Rprow_021218_1x_106253 ACTTTGCACAAAGTTAAATTTCCAAAATGTTAATGAACTAACGCACAAATACGTCTATTTAATCTTAGATTAGAAATAGCAAGGTACTGTATACGGAGTGCATAAATTTAGGAAGTTTTG 839880 840000

NC_000963_1_Rickettsia_prowazekii_str__Madrid_E_chromosome_complete_genome probe_cstm_Rprow_021218_1x_106254 ATATAATGAAAGAAATCAATATATTATTATAACATAATATTGAATTGATAAACGTAATAATTAAGCAAGAAATTGATAGTTTCATTAGTGATCGAATTTCCAGTATAAAATGAAGACTTC 840000 840120

NC_000963_1_Rickettsia_prowazekii_str__Madrid_E_chromosome_complete_genome probe_cstm_Rprow_021218_1x_106255 AGGCAGTACAATAATACAATAAACAGTGATATATTAGGAATACCATGAAATATTAAAACTCTACTAAATTTAATGTTCAGCAAAAACGTACCTCAAATATCGTTAAACAATTTAATTTTG 840120 840240

NC_000963_1_Rickettsia_prowazekii_str__Madrid_E_chromosome_complete_genome probe_cstm_Rprow_021218_1x_106256 ATATCGATGATTTTCTCGCAAGTAACGAATATaaaaaaaTTATTCAATAATGTAGTTGCAATTTGCTATGGATTTGGGCAAGCAACTTTTATAATTATATGAGTGGCAATGATATCTAGA 840240 840360

NC_000963_1_Rickettsia_prowazekii_str__Madrid_E_chromosome_complete_genome probe_cstm_Rprow_021218_1x_106257 AAACTATAACTTTTTGGGTACCATTCTTTGCTTTCATTACTGCTCATTTCGGTACAATATTACGAGATTTTATCATTAAGGCTCTTCAATTAAGAGAATACTAATAAGGTTAATATCGGA 840360 840480

NC_000963_1_Rickettsia_prowazekii_str__Madrid_E_chromosome_complete_genome probe_cstm_Rprow_021218_1x_106258 ATCACTGTATTATGGAGTATAGCTTTTAGCGTATTATTAGATAGCTACGGTAGTAGTTCCAATTATCATACAATAAAATACTCAATAATTGTAGTAATATCTGGGAGCATTTATTACTAG 840480 840600

NC_009488_1_Orientia_tsutsugamushi_Boryong_complete_genome probe_cstm_Rprow_021218_1x_10626 GTTAATTTTACATTTACTATCTCGCCTATTGTTTGCCTAGTAGCAATATAAGTAGGAATAAAATTTTCACTGTGTCCAAATTGTTCTTTTTCTACTAACACTTTAACTATCTTGCCTATT 1275000 1275120

NC_000963_1_Rickettsia_prowazekii_str__Madrid_E_chromosome_complete_genome probe_cstm_Rprow_021218_1x_106260 TTAATTTATTGCGTAACTTTTTATCTAAAGAGAATCCGGACATTTTATTATTACAAGAGATAAAATGTGAAACAGAAAAATTTCCTTTTAATGAATTATCTGATTTACCTTATCATTTTT 840720 840840

NC_000963_1_Rickettsia_prowazekii_str__Madrid_E_chromosome_complete_genome probe_cstm_Rprow_021218_1x_106261 ATGTTCACGGACAAAAATCATATAATGGCGTTGCTATAATTTCAAAATTTCCAGCTGATCAAATCATTAAGGACTTTCAGAATAATTACTGCAACGATCAAGCAAGATTCCTAGAAATAA 840840 840960

NC_000963_1_Rickettsia_prowazekii_str__Madrid_E_chromosome_complete_genome probe_cstm_Rprow_021218_1x_106262 AATTATCATCACCTATAGGATATAGTAATATCATCTCACTCTATGCTCCTAACGGTTCATTTGTCGGTAGCAATAAATTTGTAGAAAAGCTTAAATTTTATGATAATTTTATCAATTATC 840960 841080

NC_000963_1_Rickettsia_prowazekii_str__Madrid_E_chromosome_complete_genome probe_cstm_Rprow_021218_1x_106263 TATCTACTAAAAAATCTTGTTACGAAAAAACTATCATAGGTGGTGACTTCAATATTGCACCATTCGATATAGACGTATATTCTAAGGTACTCACTGAAACTACTTGTTGTACTGAGGTTG 841080 841200

NC_000963_1_Rickettsia_prowazekii_str__Madrid_E_chromosome_complete_genome probe_cstm_Rprow_021218_1x_106264 AACaaaaaaaaTTGCGTACTATTCTTAATTCTGGATTTGAGGATTTGTATAGATTAATGCATCCGGATAAACAAGAATTTTCATGGTGGGATTATAGAGCAGGATGTTTTGAACAAAATA 841200 841320

NC_000963_1_Rickettsia_prowazekii_str__Madrid_E_chromosome_complete_genome probe_cstm_Rprow_021218_1x_106265 AAGGTATGAGAATTGATATGATTCTTGGTTGTAATAATACAATTGACTATTTAGAAAGATGCTATATGGATTATAATTTAAGGACTCaaaaaaaaCCTTCAGATCATATACCGATAATTG 841320 841440

NC_000963_1_Rickettsia_prowazekii_str__Madrid_E_chromosome_complete_genome probe_cstm_Rprow_021218_1x_106266 CAAGTTTTTGCCATAGCTTGTCTACTAAATCTGGCATCAAATACTAAAATTATTCGGTATTTATTTTTATTCTGTTGATAAACTCTATTATTATAATAGAACTTGTAACACTAAACAATT 841440 841560

NC_000963_1_Rickettsia_prowazekii_str__Madrid_E_chromosome_complete_genome probe_cstm_Rprow_021218_1x_106267 TGTTTTTGCTCTATGCACCACACCAATGAGCATTATATCAAACACTTTTTGTAAAAAATATATGTCAGGGAAACTAATAAAATTTTATGAGAAATATATGACTATTGTCGGTACAATTGG 841560 841680

NC_000963_1_Rickettsia_prowazekii_str__Madrid_E_chromosome_complete_genome probe_cstm_Rprow_021218_1x_106268 CAATTTTATGTTTTATATACAGGCTAATAAGATTTGGGCCTACACATCTTCATATTCCGTTTCGATACCAACCTTCAATATAAGTGTTATTGCTTTTTGTAATTGGCTTATATACAACAT 841680 841800

NC_000963_1_Rickettsia_prowazekii_str__Madrid_E_chromosome_complete_genome probe_cstm_Rprow_021218_1x_106269 ATTAATAATAAATACACCTATTATAATAGATGTAGTTATCAGAGAATTAATTCTTCTCTTAACCATAATAATATATTAATAATGACTaaaaaaaTCATTACTTTAGTTGGTCGTCCTAAT 841800 841920

NC_009488_1_Orientia_tsutsugamushi_Boryong_complete_genome probe_cstm_Rprow_021218_1x_10627 TGTTGTTGAAAAAATAGTTGTAGTTGtttttttCCTTCATTACGCAATAATTGAGCTCttttttttCTGATATGTTTTTGCACTTGTGGCATTTTACTAGCTGGAGTATTTTCTCTTTCT 1275120 1275240

NC_000963_1_Rickettsia_prowazekii_str__Madrid_E_chromosome_complete_genome probe_cstm_Rprow_021218_1x_106270 GTAGGCAAATCAACACTTTTTAATAGATTAAGTATACGTAAAAAGGCTATCGTTCATGATTTACCAGGGGTAACTAGAGATAGAAAATATACCGATGGTAAAATTGGCTCTTTTGAATTT 841920 842040

NC_000963_1_Rickettsia_prowazekii_str__Madrid_E_chromosome_complete_genome probe_cstm_Rprow_021218_1x_106271 TTGTTAATTGATACCCCAGGACTTGAAGAGAATCCTGATAACATGGGAGAGAGGTTAATGGGACAAACTACTCAAGCAATTTTAGAGGCAGATCTAATTTGCTTTATGGTTGACGGTAAA 842040 842160

NC_000963_1_Rickettsia_prowazekii_str__Madrid_E_chromosome_complete_genome probe_cstm_Rprow_021218_1x_106272 AGTGGAGTATTACCTGATGATAAGCTACTGAGTAACTTTGTTCGCAAATACAATAAGCATTGTATATTGGTAGTTAATAAGTGTGAAAAAGCTTTTGATTTTGATAAAGAGTACTACAAA 842160 842280

NC_000963_1_Rickettsia_prowazekii_str__Madrid_E_chromosome_complete_genome probe_cstm_Rprow_021218_1x_106273 TTAGGCTTTGATAGTATTGTGATTATCTCTGCAGAGCATGGCATAGGATTAATTGATTTATATGACGCAATTATTTCTAAATTATCTGTAGAAGAATCAATTGAGCGAAATATAGCAGAT 842280 842400

NC_000963_1_Rickettsia_prowazekii_str__Madrid_E_chromosome_complete_genome probe_cstm_Rprow_021218_1x_106274 CCGTTCAGAGGAGATTGTTTGCAGATAGTAGTTAGTGGCAGACCTAACGCTGGAAAATCTACTTTTATAAATGCTATTATTAATGATGAAAGATTATTAACAGGTCCTGAAGCAGGAATT 842400 842520

NC_000963_1_Rickettsia_prowazekii_str__Madrid_E_chromosome_complete_genome probe_cstm_Rprow_021218_1x_106275 ACGCGTGAATCAATTGAAGTTGATTGGCAATATAAAAATACTCATATTAAATTGATTGATACGGCAGGACTCCGTAAAAAATCTACTATCACAGCATCTTTAGAAAAATTATCGACATCA 842520 842640

NC_000963_1_Rickettsia_prowazekii_str__Madrid_E_chromosome_complete_genome probe_cstm_Rprow_021218_1x_106276 GATACTATTAACTCTATTAAATTTGCTAATACCGTAATCTTAATGATTGATGCTTTAGCTCATGTAAAACAGCAAGATTTTAATATTGCAAGCCATATTGTAAATGAGGGAAGAAGTATA 842640 842760

NC_000963_1_Rickettsia_prowazekii_str__Madrid_E_chromosome_complete_genome probe_cstm_Rprow_021218_1x_106277 ATTATAGTAGTTAATAAATGGGATTTAGTTAAAGAATCTGAAAAAGAAGCGTTTCAGAAAGAATTTTATTATCAGATAAATACTCATCTACCTCAAATTAAAGGTGTCCCAGTTCTATTT 842760 842880

NC_000963_1_Rickettsia_prowazekii_str__Madrid_E_chromosome_complete_genome probe_cstm_Rprow_021218_1x_106278 ATCTCAGCTATAAATAAACAAAATATTGAGCAAGTTTTAGATGCTTGTCTTAAAATTTATAAAATTTGGAATAAGAAAATAACAACTAACAAATTAAATAAATGGCTTGATTTTACTACA 842880 843000

NC_000963_1_Rickettsia_prowazekii_str__Madrid_E_chromosome_complete_genome probe_cstm_Rprow_021218_1x_106279 AAAATACATCCATTGCCTCTACAAAAATGTGGTAGAAGAGTACGTATAAAATATATGACACAAATAAAAACACGCCCACCTACTTTCAAGTTATTCTCCAACAATCCAGGAAAAATTACT 843000 843120

NC_009488_1_Orientia_tsutsugamushi_Boryong_complete_genome probe_cstm_Rprow_021218_1x_10628 GAATAAGGAAAAACATGTAAATATTGAATTCCAGTTTCTGAAATCAAGTTTAAGCTATTGTTAAACATAATTTCAGTTTCAGTAGGAAAGCCAGCTATCATATCAGCTCCAAATGATGCA 1275240 1275360

NC_000963_1_Rickettsia_prowazekii_str__Madrid_E_chromosome_complete_genome probe_cstm_Rprow_021218_1x_106280 GATAGTTATACTAGGTATTTAGTAAACAATATGCGTGATGCTTTTGATATGCATGGTATTCCTATCAGATTTACTTACGTaaaaaacaaaaaTCCTTATGTATAAAATTAAATATTTTTA 843120 843240

NC_000963_1_Rickettsia_prowazekii_str__Madrid_E_chromosome_complete_genome probe_cstm_Rprow_021218_1x_106281 GTAAATGAGATATATTGAAGCTGTTTAAATACGAGTGAGCTAAAATAATGTTATTGCAAGAGATTTCATTTACAAGTACAAGTCAAGATTACCTTACAATGGTTTTGTGATTATAATTAC 843240 843360

NC_000963_1_Rickettsia_prowazekii_str__Madrid_E_chromosome_complete_genome probe_cstm_Rprow_021218_1x_106282 TTAAACCAATTTTAAGATCTCCAAATAATAAAATGATTAGCTAGACTCTATATATTGATATTCATAAATCTAGTTATAATCATAAAAAGATTTTTACTAGTACAGACACTAACGTTTCAA 843360 843480

NC_000963_1_Rickettsia_prowazekii_str__Madrid_E_chromosome_complete_genome probe_cstm_Rprow_021218_1x_106283 CCTACATGCACCATACATCACCAATATAATATAGTTATTGAGTGTAATGCAAAATTGATTTAATGCGTGATTAGTTATATATAGATTATAATCATAATGAAGCAATCATATTACTCGCGT 843480 843600

NC_000963_1_Rickettsia_prowazekii_str__Madrid_E_chromosome_complete_genome probe_cstm_Rprow_021218_1x_106284 TGATTAATCAATAAAGATATTTTTAAGCCATATCTTCTTTTATAAGTTTTAGTAGTATTACAGAAGATCTGCGGCAATTAATTTTTCAAACTTGACCTATTCTATGTTACCTTTTAGCAT 843600 843720

NC_000963_1_Rickettsia_prowazekii_str__Madrid_E_chromosome_complete_genome probe_cstm_Rprow_021218_1x_106285 ATTCGTATTATGATCCTTAATCTAGGAGGCAATACGAATTTATCATTTTTATAATCAACAAAAAAGTTCGTAACAAGATATATGCTTAATAAATAAACCTATTAGTTTTATAAAAATTGC 843720 843840

NC_000963_1_Rickettsia_prowazekii_str__Madrid_E_chromosome_complete_genome probe_cstm_Rprow_021218_1x_106286 TTAAGAAATATAAAATATGATATTAACTCATATTTTAAGTAATATCATTTACACTATTTAAGTAGTGCTTGATCAAACGTAATAACTTCGCTTCAGGACTTAGATTTTTTACTGCCCATT 843840 843960

NC_000963_1_Rickettsia_prowazekii_str__Madrid_E_chromosome_complete_genome probe_cstm_Rprow_021218_1x_106287 TTTTAATCCAAGGTTCAGCTAACTGCCACATATTAACATTACTGTCTAACTGTCTTCCTATCCCTTCCACCATTATTAAAGTCTTTTGTAAGATCAATAAATCTGGCTGTACTTCCATGC 843960 844080

NC_000963_1_Rickettsia_prowazekii_str__Madrid_E_chromosome_complete_genome probe_cstm_Rprow_021218_1x_106288 CGAAATCTTCCGTGATTTTAAATAAATGCGTCAACAACTTGCCAATTGAAATATTTTTTGTTGGTGTTCCTACTATAGGTTCAGTCACTGCTCTACAACTTCCAGCGAACAAATCTAAAT 844080 844200

NC_000963_1_Rickettsia_prowazekii_str__Madrid_E_chromosome_complete_genome probe_cstm_Rprow_021218_1x_106289 CAGTATTTGCAGGAATATAACCTGCTCTTAAATGTACTTTAGCGACTAATTTATAATCACGTTTTAAAAAGGCAAATAGAATTTCTGCAACTGATAAACGATCTTTTTCTTTAAGTCTAC 844200 844320

NC_009488_1_Orientia_tsutsugamushi_Boryong_complete_genome probe_cstm_Rprow_021218_1x_10629 TCTGGAAGAATTTTGCGCATTTTGTTGCAAAATTCTATTATTTGTTCTCTAGTGTGACGTCTTTTCATACGTTTTAATATCATATTATCACCTGCTTGCAAACTAATATGAAAGTGTGGC 1275360 1275480

NC_000963_1_Rickettsia_prowazekii_str__Madrid_E_chromosome_complete_genome probe_cstm_Rprow_021218_1x_106290 CCATAATACCAAAATCAAGCAGAATAATCTTACCTTGGTTATTGACTAAAATATTACCTGCGTGTAAATCTGCATGAAAAAAGCCGTCTCTGTAAGCTTGATTGAAAAACATTACAGCAA 844320 844440

NC_000963_1_Rickettsia_prowazekii_str__Madrid_E_chromosome_complete_genome probe_cstm_Rprow_021218_1x_106291 AATCCTGAGCTAttttttttGGTTCTAAGTTCATTTCTTTTAGAAGCGAAATATCATATATAGAAGTTCCATCCAACCACTCAGTAGTTAGTATATTTTCTGACGTTAAATCCCAATATA 844440 844560

NC_000963_1_Rickettsia_prowazekii_str__Madrid_E_chromosome_complete_genome probe_cstm_Rprow_021218_1x_106292 TTTTAGGGATTATCACGTTAATATCATGTCGCATATTATCCTTAAGCTCAGAAGCTGCTGCTGCTTCTAGCCTCAAATCAAGCTCAAATCTCATAGTTTCATGGAATTTGTCGATTACTG 844560 844680

NC_000963_1_Rickettsia_prowazekii_str__Madrid_E_chromosome_complete_genome probe_cstm_Rprow_021218_1x_106293 TAATAGGTTTTAGCCTTTTTGCTTTAGAAAATTTTGAGATAATTTTTGCaaaaaaaTATAGTAACTTAATATCTCTATTATATTTTTTCCGAATATCAGGACGCAAAATTTTTAATGCTA 844680 844800

NC_000963_1_Rickettsia_prowazekii_str__Madrid_E_chromosome_complete_genome probe_cstm_Rprow_021218_1x_106294 CATATCCACCAGTTATAAGTTGTGCTTTATGTACTTGTGAGATTGAGGCAGCAGCGATTGGATTGTTGTCGAAGTGTAGGAAAGGAAGAGATACATTACAAGTATTTGTAGAATGCTTTT 844800 844920

NC_000963_1_Rickettsia_prowazekii_str__Madrid_E_chromosome_complete_genome probe_cstm_Rprow_021218_1x_106295 CAACATTTTGTTTGTGCTCCGAAATTATCGCATCGGCACTTTTCTCACCCTTACAATGATCAATAAATGATTTATATATCAATTTTCGAGCCACCGCTCCATCAAAAGGAGGTAGTTTAT 844920 845040

NC_000963_1_Rickettsia_prowazekii_str__Madrid_E_chromosome_complete_genome probe_cstm_Rprow_021218_1x_106296 CTTGGCAGTAATCTTAAATAACATGCTATCTCTGCTCCTACTAAATCTGCTCTTGTTGAAAGAGTTTGTCCAAATTTTATATAAATAGGTCCAAGATCAGTTAAACAATCTGTTAAACGT 845040 845160

NC_000963_1_Rickettsia_prowazekii_str__Madrid_E_chromosome_complete_genome probe_cstm_Rprow_021218_1x_106297 TTACCGTAATCTTCACGTGAtttttttATTAATGAACTTGGAGCAAAAAACAAAGCTAATATATAACCTATAAATCTAAAATATTTAGGACTTCTTGAATCAATGAGAATTTGTTTTTTA 845160 845280

NC_000963_1_Rickettsia_prowazekii_str__Madrid_E_chromosome_complete_genome probe_cstm_Rprow_021218_1x_106298 CTGACTATACGGAAAATACGTATTAAATTTaaaaaaaTACTTATCATATTTTATATGCACTGTGAATAGCAACTATTCCACCACTCAAATTTTTATAATGTACCTCTTCAAAACCAGATT 845280 845400

NC_000963_1_Rickettsia_prowazekii_str__Madrid_E_chromosome_complete_genome probe_cstm_Rprow_021218_1x_106299 CTTTAATCATTATTCTAAAATCATCTTGTGATGGGAATAAAGCTATACTTTCGACCAAATATTCATATGCTTCTTTATTGCCTGCGATTATTTGACCTATACTAGGTATAATATTAAATG 845400 845520

NC_009488_1_Orientia_tsutsugamushi_Boryong_complete_genome probe_cstm_Rprow_021218_1x_1063 CAAAGATTAATGAATGAGGTGGAACAAACAGAAAAACGAAGAAACAGTAAGTTGTTGAAGGATATCGTAATAGCACTGCCAGACGATAAGGAATTGAATTTAGAAGATAGAATAAAGATT 127440 127560

NC_009488_1_Orientia_tsutsugamushi_Boryong_complete_genome probe_cstm_Rprow_021218_1x_10630 ATTAAACGTGGTTCATCAGTCATCAATTTAAATAATTCTTGATCTATTTCAGCTATATCTATTGAAGATAATCTCAATCTTGGTAAAGCTGGTACTAGCATTAAAACACGTTTAATCATT 1275480 1275600

NC_000963_1_Rickettsia_prowazekii_str__Madrid_E_chromosome_complete_genome probe_cstm_Rprow_021218_1x_106300 AGTAGAATTTATAAAAATCTTTTAAGATGCCCTCTTTTACTTTTGAAAACTCAAGACATACAAACTTCCCCATAGGCTTTAATACCCTATAAGCTTCCTTCAAAGCTTTATTAATGTCAG 845520 845640

NC_000963_1_Rickettsia_prowazekii_str__Madrid_E_chromosome_complete_genome probe_cstm_Rprow_021218_1x_106301 GTACGTTTCTAATACCAAATGCTATAGTATAATAATCGAAACTATTATCTGAGAATGGCAACTCTTCAGCATTTGCTACAATAAATTTAATGTTCTGAAATAGATTTAGATCAATTGATT 845640 845760

NC_000963_1_Rickettsia_prowazekii_str__Madrid_E_chromosome_complete_genome probe_cstm_Rprow_021218_1x_106302 TCTTTTTAGCATTATTTAACATCTCTTCATTTATGTCACTTAAAATTAGAGAAATATTATTACCTCGATCTTTTGCTTTTTTAGCGAGTTTTAAAGCAATATCGCCACTACCGCTAGCGA 845760 845880

NC_000963_1_Rickettsia_prowazekii_str__Madrid_E_chromosome_complete_genome probe_cstm_Rprow_021218_1x_106303 CATCTAATATATTAGAGTTAAGATTTGGAATTTGCATAATAAACTCATCTTTCCACAGACGATGTAAGCCAATACTCATTAAATCATTCATTAAATCATACTTATCAGCTACTCTAGAAA 845880 846000

NC_000963_1_Rickettsia_prowazekii_str__Madrid_E_chromosome_complete_genome probe_cstm_Rprow_021218_1x_106304 AAATGTTATTTATTAACCATTGTTTTTTAGTGTAATCTACTTTCTTAAAGCCAAAGTTTGTGTTGTACATGTATATATGATTAAAAATATAGTAACATAATGATTCTTTATTTACTGTCA 846000 846120

NC_000963_1_Rickettsia_prowazekii_str__Madrid_E_chromosome_complete_genome probe_cstm_Rprow_021218_1x_106305 TTCTTAGTATAAACAGTGTGAATCAAGTACTTTAACGTTGCAAGCATTATCATAAAAAAGCTTGCACATTAAGCTTTTTTAGCATAGATCAATAGTTTTACCTACATAACATTGAGGTAT 846120 846240

NC_000963_1_Rickettsia_prowazekii_str__Madrid_E_chromosome_complete_genome probe_cstm_Rprow_021218_1x_106306 TTAGGTCAAGACACGTATAGTAACATATTTATGATTGATCCACGCATGTAATATTTGCTTACAATATTACTTAAGTTTCAATTTTTGGACATTAGTTTTATCAAAATCATAGATAAACTC 846240 846360

NC_000963_1_Rickettsia_prowazekii_str__Madrid_E_chromosome_complete_genome probe_cstm_Rprow_021218_1x_106307 TTTAATAAGCGGTACTATAAACTGTTTAAATTTACTTCCACTAAATACTCCATAATGCCCTACACCGTTTTGTAAATGATATCTTTTCATTGATTCAGATATATTTACACAAAGTTTTAA 846360 846480

NC_000963_1_Rickettsia_prowazekii_str__Madrid_E_chromosome_complete_genome probe_cstm_Rprow_021218_1x_106308 AGCAGCTTTTGTTTGACCTACTGCCGCAATATCATCTAATTCTCCTTCAATACCAAGTAAAGCACATTTAGTAATATGTTTTAAATCAATCGGACGCTTCTCAGAAATAAATTTACCTCT 846480 846600

NC_000963_1_Rickettsia_prowazekii_str__Madrid_E_chromosome_complete_genome probe_cstm_Rprow_021218_1x_106309 TGCTAATGAAAATTGTTGAAATACTTCATCTATAGTTTGTAAGTAAAATTCTGCTGGCATATCCATACCTGATAAATACTCATCATAAAATTTAATAATATAATCAGCTTTTTGATAATC 846600 846720

NC_009488_1_Orientia_tsutsugamushi_Boryong_complete_genome probe_cstm_Rprow_021218_1x_10631 TGAGCTAAAGTAGGAGATCCTGGTAAGTCAGCTCCATAAGAAGTTAAATCTACTCCTGTAAAAACTATTTCCTTGTACCCTTGATTGATAAGAAGCTGAATCTGCTGAGTTATTACACCA 1275600 1275720

NC_000963_1_Rickettsia_prowazekii_str__Madrid_E_chromosome_complete_genome probe_cstm_Rprow_021218_1x_106310 AGAATTTAATAAACTTTGCCATAATTCTAAATGTGAATCTATATGACGTAATAAGTTTAAACTTATAAATCCGGTAAGTTGTAGGAACCCTGGATATACTTTTCTACCATATCCGGGATA 846720 846840

NC_000963_1_Rickettsia_prowazekii_str__Madrid_E_chromosome_complete_genome probe_cstm_Rprow_021218_1x_106311 GTTTGGTGGCACCTGCATTGTAAGCATCTCACAGAACCATTCCAAACTTTTACTGAGTGCGAATTCATTAACTACAGTTGGATTTTTTCTAGCATCGATAGGACCACCAATCAAAATCAT 846840 846960

NC_000963_1_Rickettsia_prowazekii_str__Madrid_E_chromosome_complete_genome probe_cstm_Rprow_021218_1x_106312 CGAACTCGGCACATTTGGACTATTATTTTCTGACATTAAACTAATGGCAGCAAGCAATGGAACAGTAGGTTGGCAAACTGCCATAGTGTGAATATTCTGTCCCATAAAATTTATAAACTC 846960 847080

NC_000963_1_Rickettsia_prowazekii_str__Madrid_E_chromosome_complete_genome probe_cstm_Rprow_021218_1x_106313 AATTAGGTAATCAATATAATCATCCATATCAAAATGCCCTGCTTCAAGCGGTACATAATTGGCTTCTGTCCAATCTGTAATATAAATATCAGTATACGGTAATAATGCATGCACTGTTGA 847080 847200

NC_000963_1_Rickettsia_prowazekii_str__Madrid_E_chromosome_complete_genome probe_cstm_Rprow_021218_1x_106314 TCTAAGTAAAGTAGCATGATGTCCTGCCATAGGTGCTACTATTAATAATTTAGGTAGTTCTTTTTTAAAACCTATTTTTTGAAAATGCCTTAGTTCACAAAAAGGTTTCTTAAAAATAAC 847200 847320

NC_000963_1_Rickettsia_prowazekii_str__Madrid_E_chromosome_complete_genome probe_cstm_Rprow_021218_1x_106315 TTGTTCATTAATATTATAAGTTTTATCATTAATAATTGTTTCAAGAATATTAAATTCAGGTTTTGTATATTTTCTAGTCATGCGTTCGCAAAGCGTTAAATACGCATGTATAATTCTTGC 847320 847440

NC_000963_1_Rickettsia_prowazekii_str__Madrid_E_chromosome_complete_genome probe_cstm_Rprow_021218_1x_106316 AAAACCGTTATCTTCTAGAGGCTCATGTTCATATAATTCTTTTAGAATTTTTATACCTAAATGCATTGGAGCAATTTGTGCTCTAAGCCACTCTAAAATATAATATGTATAATTAGTACT 847440 847560

NC_000963_1_Rickettsia_prowazekii_str__Madrid_E_chromosome_complete_genome probe_cstm_Rprow_021218_1x_106317 TAAATCACAATGCATCATAATCTCCAAATTTaaaaaaaTTAATAATATTTAGTTTAACATCAAAAACTCATTCAGTAATATTAATTTTAAAATTCACCACTAAGTTTTTATAGATACGTG 847560 847680

NC_000963_1_Rickettsia_prowazekii_str__Madrid_E_chromosome_complete_genome probe_cstm_Rprow_021218_1x_106318 ATAATAATGGTCTATATAGACGTTTATTTTTGTTTTACTGCAAAATTTATTTACGTGTTTTAGTATGCAAGCAAGTACTAGTTTTTATAAAATGTGTCAATATTTTAAttttttttATTA 847680 847800

NC_000963_1_Rickettsia_prowazekii_str__Madrid_E_chromosome_complete_genome probe_cstm_Rprow_021218_1x_106319 TATTTTGCTAATAATCTATAAGATAGATATCTGATCTTTTCTAATAAGCATCAATATTTTCAACTATTAAAATAAAACTATATCATGTCCATTCCACAATATTTAAAGTTAATAACTAAT 847800 847920

NC_009488_1_Orientia_tsutsugamushi_Boryong_complete_genome probe_cstm_Rprow_021218_1x_10632 ATTGGTACAGATCTACTATTACCTCGAACATATGGTATTATGCAAAACGTACAGCGATGGTTACATCCATTTTGGACCTGTATAAATGCTCTTGTTTTACCATCAAAATTGCTAATCATA 1275720 1275840

NC_000963_1_Rickettsia_prowazekii_str__Madrid_E_chromosome_complete_genome probe_cstm_Rprow_021218_1x_106320 ATCATATTTCTATGTTTTTGTGACTTTAAATTATCAATCTTGATGATTTTAAGTATATAAGTATAATTTATACTATTAGTAGTAAATCAGCAGATAAGATAAGAAGAATTGAATTTAAAA 847920 848040

NC_000963_1_Rickettsia_prowazekii_str__Madrid_E_chromosome_complete_genome probe_cstm_Rprow_021218_1x_106321 TAACAATGTATCTTCAAATTTATGTCAACAGTATACACGCTAGTAATGAGGATTAACAGTAAGAGACAACGTAGCAAATTTTTTAAATTAAACTAACCTGCTTTTCAAATTTTGAAAATA 848040 848160

NC_000963_1_Rickettsia_prowazekii_str__Madrid_E_chromosome_complete_genome probe_cstm_Rprow_021218_1x_106322 ATATTTTGAAGTTATGAGTAGTAACATTTTCTACTTCTTGTGAAGTAATATTTTTGAGTTCGGCAACTTTCTCGGCAACATATCTAACAAATGCTGGTTCATTTTGTTTGCCGCGCATAG 848160 848280

NC_000963_1_Rickettsia_prowazekii_str__Madrid_E_chromosome_complete_genome probe_cstm_Rprow_021218_1x_106323 GAGTAGGAGCTAAGTAAGGAGAATCTGTTTCTATTAATAACCTATCAAGTGGTATATATTTAACTATCTCTTGCAAATTTGTTGCATTCTTAAAAGTTATAATCCCTGACACTGAAATAT 848280 848400

NC_000963_1_Rickettsia_prowazekii_str__Madrid_E_chromosome_complete_genome probe_cstm_Rprow_021218_1x_106324 ATAAACCTATATCTAACATTTTTGCCGCTAGATTCTTTGAAGAGGCGAAGCAATGTATTAAACCTGAAAATTTGCTATTATGCATTGCTGATGTTAAAATATCAATAGTATCTTCATCTG 848400 848520

NC_000963_1_Rickettsia_prowazekii_str__Madrid_E_chromosome_complete_genome probe_cstm_Rprow_021218_1x_106325 CTTCTCTTGTATGAACAATAATAGGTAGATTAGTAGCTGATGCTGCACTTATATGTGCTATAAATGAATCTCGTTGTAATTTTTTATTATAAGGCTGGTGATAATAATCAAGTCCTGTTT 848520 848640

NC_000963_1_Rickettsia_prowazekii_str__Madrid_E_chromosome_complete_genome probe_cstm_Rprow_021218_1x_106326 CCCCTATACCGATAATTTTCTGATGGTTGGTAAGCTCTATTATTTCTAAATCCGTAATTAGTCTTTTCTTGTCATTAACCTCACATGGATGCACACCAACACTTGCAAAAACATTTTTAT 848640 848760

NC_000963_1_Rickettsia_prowazekii_str__Madrid_E_chromosome_complete_genome probe_cstm_Rprow_021218_1x_106327 ATTGTTCAGCTATTTTTAAAACAATAGGTAAATCTTCAAGCTTAGTGCAGATAGTTTGTATATATTGTACATTATTTTCTAATGCTCTTTGAATGAAAGAATCTAAAAACACCGTATCAT 848760 848880

NC_000963_1_Rickettsia_prowazekii_str__Madrid_E_chromosome_complete_genome probe_cstm_Rprow_021218_1x_106328 ACATTTTAGTATCaaaaaaaCAACTAGAATTAGTTGTTTTAATATTTTTACTAGATATTGTGATCGAACCACAGGATGAAAAATTAGTAAGTAAATTAAGATGACAATGTGAATCTATTA 848880 849000

NC_000963_1_Rickettsia_prowazekii_str__Madrid_E_chromosome_complete_genome probe_cstm_Rprow_021218_1x_106329 ACATATTATACAATTAATAAGGGTAATAGTATGACTTTACTATGGTATCTAACTCAAAACGATAAATTAGAGCTTTTAATTTTTAAAATAGTCTTTTTTACTCGACACCATGTACAAATC 849000 849120

NC_009488_1_Orientia_tsutsugamushi_Boryong_complete_genome probe_cstm_Rprow_021218_1x_10633 TGAGTAGCAGTTTCTTGAATTGACATAATATCATTTACTTGAATTTTATTTTCATCAAATTGATAAAATTCAGGATACAGTTTTTCCTCATTGCCAAGTATCTTATTCACTTGTGGCATT 1275840 1275960

NC_000963_1_Rickettsia_prowazekii_str__Madrid_E_chromosome_complete_genome probe_cstm_Rprow_021218_1x_106330 ATAATATTACTAGTTAAACTTTGGAAAAATTATACTAGGCTCTAAAATATTACTACCTGCTTTTAAAGCATGATCACGTACTAGATGTTTAAATAATCTCTCTTCTTTAGATACTCCTAA 849120 849240

NC_000963_1_Rickettsia_prowazekii_str__Madrid_E_chromosome_complete_genome probe_cstm_Rprow_021218_1x_106331 CTGATCTAACATTTTATTTGCTGAATTTGGTACAAAAGGCTGAAGCATTATTGCAATATAACGTAGTACTTCTAACAGAGAATATAACACCTCTAACATCTTATCAGGATCAGTTGTTTT 849240 849360

NC_000963_1_Rickettsia_prowazekii_str__Madrid_E_chromosome_complete_genome probe_cstm_Rprow_021218_1x_106332 TAAATTCCAAGGCGCTTCATTAGTAATATAGATATTTGCATCTTCAGCCAAATTAATAATATTTTCAAGAATTTTATTAATCTCTGTTTTATCCATCAATAAAATATTCTCTTGAGCAAA 849360 849480

NC_000963_1_Rickettsia_prowazekii_str__Madrid_E_chromosome_complete_genome probe_cstm_Rprow_021218_1x_106333 TTTAATTGCGGTTTTTAAAATCGGGAGCTCATATATTTTATCTATAACACCTGACTTAATCAAAGGAACTTTAGCATCATTATTCTTATAAACAAAAGATACAATACGATGTAATAAATT 849480 849600

NC_000963_1_Rickettsia_prowazekii_str__Madrid_E_chromosome_complete_genome probe_cstm_Rprow_021218_1x_106334 ACCTATTTTATTTGAAAGTTCACTATTAATACGTGTAATTAAATTATTACGAGCAAAATTAGCATCTGCCCCAAAAATGACTTCACGCATAAGAAAATACCTAACCTGATCAACACCAAA 849600 849720

NC_000963_1_Rickettsia_prowazekii_str__Madrid_E_chromosome_complete_genome probe_cstm_Rprow_021218_1x_106335 TTCATCGATTAATGTAATCGGATCAATGGTATTACCCAGAGATTTAGAAATCTTCTGTCCCTCATTAGTCCACCAACCATGTACCATAATTGCCTTTGGCAGTGGAATTTCCGCAGCCAT 849720 849840

NC_000963_1_Rickettsia_prowazekii_str__Madrid_E_chromosome_complete_genome probe_cstm_Rprow_021218_1x_106336 TAAGAAAGCAGGCCAATATACAGCATGAAAACGCAATATATCCTTACCTACTATTTGTAAATTAGCAGGCCAAAACTTGGCATAATTACTTTGTTTATCAGGATAACCAAGTGCTGAAAT 849840 849960

NC_000963_1_Rickettsia_prowazekii_str__Madrid_E_chromosome_complete_genome probe_cstm_Rprow_021218_1x_106337 ATAATTAACAAGTGCATCTAGCCAAACATAAATTACATGTTTTCTATTATTTGGTACTTTGATTCCCCAATGAAAAGTAGTGCGTGATATGGATAAATCTTTTAGACCTGATTTAATAAA 849960 850080

NC_000963_1_Rickettsia_prowazekii_str__Madrid_E_chromosome_complete_genome probe_cstm_Rprow_021218_1x_106338 GCTAATTACTTCATTACGCCTTGATATTGGTCTGACAAAATCAGGGTTTAGCTCATAAAACTCAAGTAATTTATCTTGCCATTTTGCAAGATTAAAGAAATAGCTTGGTTCTTTAACCCA 850080 850200

NC_000963_1_Rickettsia_prowazekii_str__Madrid_E_chromosome_complete_genome probe_cstm_Rprow_021218_1x_106339 TTCAACAGGCGCTCCAGTTGGTGCTAATTTATCTTCATTTATCTCTGATTCATCATAAAAAGCCTCATCACGCACTGAATACCAACCCTCATAAAATCCTTCATAGATTGCACCATTATC 850200 850320

NC_009488_1_Orientia_tsutsugamushi_Boryong_complete_genome probe_cstm_Rprow_021218_1x_10634 TGATTAAAGAGATCAGGGTTGTTCTGAGCAGCGCAGCCAGTAACAATAATCTTAATGTTAGGATTTTCTCTTTTTGCTTTTCTAATAGCTTGCTTAGCCTGCCTTTCAGCTTCTTGTGTT 1275960 1276080

NC_000963_1_Rickettsia_prowazekii_str__Madrid_E_chromosome_complete_genome probe_cstm_Rprow_021218_1x_106340 TAGTAATTTCTTCCAAAAAACGGCTACAGCTTTTTTGTGTCTATTTTCAGTTGTTCTAATAAAATCATCATTAGAAATATTCATTGCAACCATAAGATCACGAAAATTTTGTGACGTCTT 850320 850440

NC_000963_1_Rickettsia_prowazekii_str__Madrid_E_chromosome_complete_genome probe_cstm_Rprow_021218_1x_106341 ATCAGTAAATGTTTGAGGATCAATATTTTGATTAATAGCTGCTTTTTCTACTTTTTGTCCATGTTCATCAGTACCTGTTAGAAACATTACATCCTTACCGCAAAAACGCATAAAACGGGC 850440 850560

NC_000963_1_Rickettsia_prowazekii_str__Madrid_E_chromosome_complete_genome probe_cstm_Rprow_021218_1x_106342 AATAACATCACTTGCAACACTAGTATAAGCGTGACCGATATGGGCAACATCATTAACATAATATATAGGGGTAGTAATATAGTAAGTATTTTTCATAAATGCTTTTGTATTAACTCATGT 850560 850680

NC_000963_1_Rickettsia_prowazekii_str__Madrid_E_chromosome_complete_genome probe_cstm_Rprow_021218_1x_106343 ACTTCAAGTGGGCTTAAATGTGAAGCTTTAATTGTTTTAATTCTCTCAGGAAATCTATTACTTAATTCTTTAAAGCAAGTATAGAtttttttATAAAAATCTATACTTCTTATATCAAAT 850680 850800

NC_000963_1_Rickettsia_prowazekii_str__Madrid_E_chromosome_complete_genome probe_cstm_Rprow_021218_1x_106344 TTATTACTCATATTCCGTGCATTCACTCTCTTAATAGCAGTATGCGGTTCTACATCAATAAAAAACGTAATATCTGGCATAAGAGAAGGCATCAAAGTTTTGTGCAAACTATACACTAAA 850800 850920

NC_000963_1_Rickettsia_prowazekii_str__Madrid_E_chromosome_complete_genome probe_cstm_Rprow_021218_1x_106345 TCTATACCATTTTCTAGCTCTAATCCTTGATAACATGCAGTTGAATCAATAAACCTATCACATATTACTATATATCCATCTTTTAGTGCAGGAATAATCTTACGTGCCATATGATCATAA 850920 851040

NC_000963_1_Rickettsia_prowazekii_str__Madrid_E_chromosome_complete_genome probe_cstm_Rprow_021218_1x_106346 CGTGCTGCCATAGCTTGTAATAACTCAGACATCGGTAATAATTCCTCATTCACTAAAATTTCACGCATTTTTTCAGCAACAGTAGTACCACCAACTTCACGAGTTAAAATAACAGGAATT 851040 851160

NC_000963_1_Rickettsia_prowazekii_str__Madrid_E_chromosome_complete_genome probe_cstm_Rprow_021218_1x_106347 TTTTGAGATTTTAAATATTCATAGAGCATTTTAGATTGGGTAGATTTGCCTATCCCATCCACTCCCTCAAAGGTAATAAACTTGCCTTGTGTTAATTTGTTCATTAAAAATTGCAATTTT 851160 851280

NC_000963_1_Rickettsia_prowazekii_str__Madrid_E_chromosome_complete_genome probe_cstm_Rprow_021218_1x_106348 AAAAACTAAAGCATAATCATATCTTTaaaaaaaaTATTTACTATTAtttttttATTGCATGATATACTTACTTGAATAACCACTTAATTATTAGCAAATACTTTACTTTAAAATCTACAA 851280 851400

NC_009488_1_Orientia_tsutsugamushi_Boryong_complete_genome probe_cstm_Rprow_021218_1x_10635 ACAGTACAAGTATTAAATACTATAACGTTATCAAGATTAGCAATTTTAAGATTCTGTTTAATAATCTCACTTTCATATGCATTTAAGCGGCAACCAAATGTTATTACCTTATTAGTGTAA 1276080 1276200

NC_000963_1_Rickettsia_prowazekii_str__Madrid_E_chromosome_complete_genome probe_cstm_Rprow_021218_1x_106350 GGCTTAGATATATCTTCTCCTGATCAAGAATTACATTTAAAACTTAATTTTTGATTTTTAAAACTTTTGCTAAATCATTTTGATAAAGAACGCTTAATTATGTATAAAATAAACGAGGTA 851520 851640

NC_000963_1_Rickettsia_prowazekii_str__Madrid_E_chromosome_complete_genome probe_cstm_Rprow_021218_1x_106351 AAGTATAATAAAGGCACAAACGTATTAATTATAAGACTACGGTATTGCATATTATTTATTACTTTTCTTTAAAAAATTACTTGAATAAAATTAATATTTAGCACTGCAATTATTTATTAT 851640 851760

NC_000963_1_Rickettsia_prowazekii_str__Madrid_E_chromosome_complete_genome probe_cstm_Rprow_021218_1x_106352 TACGAAGTTAAGTTTAGAACATACTAAAACACATGGTGATCTATTTAAATGAGCAATTCGTTAAGCAAAAGGGATTTTTCCATCATAATCGGTAATGCTATGGATCATTTCGATACTGCA 851760 851880

NC_000963_1_Rickettsia_prowazekii_str__Madrid_E_chromosome_complete_genome probe_cstm_Rprow_021218_1x_106353 CTTTATGGATTTCTAGCACCACTACTTGCAAGtttttttttCCCGAATCATGATAAAGTTGTGGCCTTAATTCTTACCTATAGTGTACTTGCTACGTCTTTATTTACACGGCCTATAGGT 851880 852000

NC_000963_1_Rickettsia_prowazekii_str__Madrid_E_chromosome_complete_genome probe_cstm_Rprow_021218_1x_106354 TCTTATTTCTTTGGCGTTATTGCTAAAAAATATGGAAGTATTTTTGCTTTATCTCATTCATTAATCGGAATTGCGTACACTACTGTTTTGATAGGCTTAATACCATCTCATGCACAAATA 852000 852120

NC_000963_1_Rickettsia_prowazekii_str__Madrid_E_chromosome_complete_genome probe_cstm_Rprow_021218_1x_106355 GGATGGTTTGCTCCGCTATTATTAGTAGTGCTTAGAATATTACAAGGAATATATTCTGAAGGCGAATGCGCAATTGCTCAATTAGTTATTTTAGAGAATAAAGAAGaaaaaaaaGCGTTT 852120 852240

NC_000963_1_Rickettsia_prowazekii_str__Madrid_E_chromosome_complete_genome probe_cstm_Rprow_021218_1x_106356 AAAGCTTCATACCTTTATCAAACCTCAACTATGTTAGGTATTATTCTTGCTTCATTTATCAGTAGCATAGTTTTAAATCTGGAATATAATGAATATTGGAGATTATGTTTTATATTCGGC 852240 852360

NC_000963_1_Rickettsia_prowazekii_str__Madrid_E_chromosome_complete_genome probe_cstm_Rprow_021218_1x_106357 GGTCTTACAGGATTTATAGGTGCTTTTCTAAGAAAGAGTGAGACAATGGTATTCAACGACTTGGTTACAGAACCTAGCAAATCACTCAATAATCTAGATCTCGTGATCAAACAACGTGAT 852360 852480

NC_000963_1_Rickettsia_prowazekii_str__Madrid_E_chromosome_complete_genome probe_cstm_Rprow_021218_1x_106358 AATATTATGGTAATGTGTACTTTTGCAGAAATGAAATTTTTAAATGATCTAACTACAATTTGGAACAACAAACTAAGTATCTTACGTATTAGTGTTGCTGTAGGGTTCTCATATATGAGC 852480 852600

NC_000963_1_Rickettsia_prowazekii_str__Madrid_E_chromosome_complete_genome probe_cstm_Rprow_021218_1x_106359 TACAGCGTACCTTTCGTTTTTATGAATAGTTTTATTCCACTTATTACAAATATATCTATTGCAAAAATGATGGAATTTAATACTGAATTTCTGATTTTTGATATGGTTATGATTCCAATA 852600 852720

NC_009488_1_Orientia_tsutsugamushi_Boryong_complete_genome probe_cstm_Rprow_021218_1x_10636 GTACTAGTACTTATTTTATCTTCATTCATTTGAATTGTTATAATTATAGGAGTATTAAGTAGCTTTATTTAATACTACTTTACTAAGATTAGCAAACTATATTACTATATAATATAGTTG 1276200 1276320

NC_000963_1_Rickettsia_prowazekii_str__Madrid_E_chromosome_complete_genome probe_cstm_Rprow_021218_1x_106360 ATAGGTCATCTCACaaaaaaaTTAAATTATCATAAAATACTAAATGGTACTCTTATTATAATGAGTTTAAGCATAATTCCTTTATGGTTATTCTTGAATAATGCATCTATATGGTACGTT 852720 852840

NC_000963_1_Rickettsia_prowazekii_str__Madrid_E_chromosome_complete_genome probe_cstm_Rprow_021218_1x_106361 CATTTTGTACGTATTTGGATTATAATACTTGGTGTCAGCTTCTTAGCGCCTTTAAACTGCTGGCTCAATAATCTATTTAAAACTAACGACAAATACATGTTAGTAGGTATCGGAAGCAGT 852840 852960

NC_000963_1_Rickettsia_prowazekii_str__Madrid_E_chromosome_complete_genome probe_cstm_Rprow_021218_1x_106362 ATCGGTGCTTCACTAATAGGACGCCTTACATCATCAATTTGCCTAATGCTATGGCATATAACCAACAATTCCTTATCAATTGGAATTTATATAGCGATAGTCTCAATGATAACCTTATGT 852960 853080

NC_000963_1_Rickettsia_prowazekii_str__Madrid_E_chromosome_complete_genome probe_cstm_Rprow_021218_1x_106363 GCAGTTAAAAGAGTAGTTACAACATCCTGCATGACAAAGCAATAATCCAATAAAACATATAAAATTCACAATCAGAATTTAACAAAACGCCATAATTAAAAGGACACCTACATAATTATT 853080 853200

NC_000963_1_Rickettsia_prowazekii_str__Madrid_E_chromosome_complete_genome probe_cstm_Rprow_021218_1x_106364 AGCGCTAAATCTTTTTATACAATTCACAGGATTTTTGATATCAAGAGTTGCTATTTGCAATATTAGTAAAATTAAAGCAGCTAAAACAGGTAAATAATCGATATTAAAATTAGCAATTTT 853200 853320

NC_000963_1_Rickettsia_prowazekii_str__Madrid_E_chromosome_complete_genome probe_cstm_Rprow_021218_1x_106365 TGTTGCTAGAATAAATAACAATATAAAACCTATATAACATATATAAAGCCAAAACTTAGGATTCTTATTCTCTAAATATATGCTTAAAGATTTTACTCCTATTTTCTTATCATCTTTTAT 853320 853440

NC_000963_1_Rickettsia_prowazekii_str__Madrid_E_chromosome_complete_genome probe_cstm_Rprow_021218_1x_106366 ATCCATATATCCGTAAATCGTATCATATCCAATCGCCCAAAAGCAACATGCTAAATACATAATAATTGCTTCTGTATCAAGTTTATCCTGCACTGCTCCATAAGCTACTAATACTCCTAG 853440 853560

NC_000963_1_Rickettsia_prowazekii_str__Madrid_E_chromosome_complete_genome probe_cstm_Rprow_021218_1x_106367 TTTAAATGTAAAACCTAGAAAGATTTGCGGGAAGTAAGTAATACGTTTCATTAATGGGTATAGAATAATCATTATTACTGCAAAGAATCCAGTATATATGGCTGTTTTGTTTAAAAGTAG 853560 853680

NC_000963_1_Rickettsia_prowazekii_str__Madrid_E_chromosome_complete_genome probe_cstm_Rprow_021218_1x_106368 CAAAATACTAAGCGAGATAATACTAAGAATCAATAGTATAAAAATAGCATAAAATACAGATACAGCACCACTGGCCAAAGGTCGTTTTTTAGTCCTCTCAACATACTTATCAAATTTTCG 853680 853800

NC_000963_1_Rickettsia_prowazekii_str__Madrid_E_chromosome_complete_genome probe_cstm_Rprow_021218_1x_106369 ATCAAATATATCGTTAATAATACATCCGCTACTTCTAGCTGTTATACTACCGAAAATAAATAACGGTAATAAATAAGCTAATTCCATGTTAGAAGGATTTGCCAGCAATAATCCAAATAA 853800 853920

NC_009488_1_Orientia_tsutsugamushi_Boryong_complete_genome probe_cstm_Rprow_021218_1x_10637 ATAGTTTTTAACGCCAGATTAGGATAAGTGGAAAGATGAAATGTAGAGAATAAGAGGTATACAAAGAGAGAATGTTAAGTTATaaaaaaaaTTTAAATAAACAGTGAATAACCCAACATa 1276320 1276440

NC_000963_1_Rickettsia_prowazekii_str__Madrid_E_chromosome_complete_genome probe_cstm_Rprow_021218_1x_106370 AGCAGGaaaaaaaaCTAGCAAATAAGCTACCGGCTTATCTGCACGCATTAATTTAAAGATAATTAAAAGTTTATTTAGCATTAACATATTAAGTTtaaataaatacagataatttaaata 853920 854040

NC_000963_1_Rickettsia_prowazekii_str__Madrid_E_chromosome_complete_genome probe_cstm_Rprow_021218_1x_106371 aaatacgcaacaaaaataaatCTACTAGATTTTTATAGCTAAAAACTCAAGCATGATTAATTAGTAAAAATAATAATGACAGTCAAATAGCAGCTATAAGGAGTTTTTATTATTTAGCTA 854040 854160

NC_000963_1_Rickettsia_prowazekii_str__Madrid_E_chromosome_complete_genome probe_cstm_Rprow_021218_1x_106372 AGCATTTTAAATGCTAGTACATCTATCTAACTGTAGAGTACAACAATAGTGTAATAGGATATAAATTACAATTATTGATTATTGGATGATTTTGAGTTTATGCTAATATATCCGCATTTA 854160 854280

NC_000963_1_Rickettsia_prowazekii_str__Madrid_E_chromosome_complete_genome probe_cstm_Rprow_021218_1x_106373 GTTATTTACAACTGTAAAAAAGTGTATAATGCTaaaaaaaTTTGAGCGATTTCTTCATAAGACAATAGAAAAGCAATAAATTACTTGAGCTGAGGGTTTATTTACTAATTTCAGATCATG 854280 854400

NC_000963_1_Rickettsia_prowazekii_str__Madrid_E_chromosome_complete_genome probe_cstm_Rprow_021218_1x_106375 tcatagtattGAATCAACATCTATTACCACAACATAAGAATATTATTATGAAAACTATAGCATATACATGTAAATATGCTGGAAGGAGTCATGGTATAAAATAAGAACACTTATTGCGCA 854520 854640

NC_000963_1_Rickettsia_prowazekii_str__Madrid_E_chromosome_complete_genome probe_cstm_Rprow_021218_1x_106376 CATAACAAAGTGGTAATATAGCAAAACAAATTTGTTCTCAAGTAAAGAATCAAGGACTAAAAATTTCTATGATTGATTAGGTTAAAGATATTAAAGAAACTTTTATGCATTCAATTATAA 854640 854760

NC_000963_1_Rickettsia_prowazekii_str__Madrid_E_chromosome_complete_genome probe_cstm_Rprow_021218_1x_106377 AAAAGTAATTTAACGACGATAAGTTACATGCTGTACAAATGCTAAATCTCTAAACCAAAGCGAAACAACACATCTTGTCATAATCATACATAATCTTTATAATAATATTCTTAACACGAA 854760 854880

NC_000963_1_Rickettsia_prowazekii_str__Madrid_E_chromosome_complete_genome probe_cstm_Rprow_021218_1x_106378 TAAAGAATATGAAAGAATTTCCAAAGCATTACAATTTTACCGAAAATGAGAAAAAGTGGCAGAACATTTGGCAAGAGCTACAAATTTATGCGTATGATCCAAATATTTCAAAAGCAGAAA 854880 855000

NC_000963_1_Rickettsia_prowazekii_str__Madrid_E_chromosome_complete_genome probe_cstm_Rprow_021218_1x_106379 TCTACATAGTGGATACGCCACCACCTACTGTGTCAGGGCAATTACATATTGGGCATATTTATAGCTATACGCAAACAGATTTTATTGTACGTTTTCAACGTATGATAGGTAAAAATATCT 855000 855120

NC_000963_1_Rickettsia_prowazekii_str__Madrid_E_chromosome_complete_genome probe_cstm_Rprow_021218_1x_106380 TTTATCCTATTGGTTTTGACGATAATGGACTACCAACCGAGAGACTTGTTGAAAAGCAGAAGCAGATTAAAGCTTATAATATGGAGAGAGATGAGTTTATAAAAATTTGCCTGGAAGTTG 855120 855240

NC_000963_1_Rickettsia_prowazekii_str__Madrid_E_chromosome_complete_genome probe_cstm_Rprow_021218_1x_106381 TAAAGAATGAAGAAGCAAAATTTAGAAGTTTATTCAAACAAATAGCTTTATCTGTTGATTGGAGTTTAGAGTACCAAACTATCAGTCCATTATCACGGAAAATATCACAAATGTCTTTTC 855240 855360

NC_000963_1_Rickettsia_prowazekii_str__Madrid_E_chromosome_complete_genome probe_cstm_Rprow_021218_1x_106382 TTGATTTACTACATAAAGGAGAAGTTTATCGTGCTAATCAACCAATTTTATGGGATACTGTAGATGGTACTGCTCTTGCTCAAGCAGATATTGAAGATAAGCAGAAAATCTCTTCTATGA 855360 855480

NC_000963_1_Rickettsia_prowazekii_str__Madrid_E_chromosome_complete_genome probe_cstm_Rprow_021218_1x_106383 ATTATATTACATTTAAAACCGAGCAAGGAGACCAGCTTACTATAGCCACTACAAGACCTGAGTTATTACCAGCGTGCGTAGCGGTATTTTATCACCCTGACGATGTGCGTTATAAGCATC 855480 855600

NC_000963_1_Rickettsia_prowazekii_str__Madrid_E_chromosome_complete_genome probe_cstm_Rprow_021218_1x_106384 TAGCAGATAAATCAGCTATAACGCCACTCTTTAATGAGAAAGTTCCAATCCTAGCTGATCCTTTAGTACAACAAGATAAAGGTACAGGACTGGTAATGTGTTGTACATTTGGTGATCAAA 855600 855720

NC_000963_1_Rickettsia_prowazekii_str__Madrid_E_chromosome_complete_genome probe_cstm_Rprow_021218_1x_106385 CAGATATTACATGGTGGAAGTCACATAATCTACCATTAAAAACCATTATCACTAAAAAAGGGACTATAAATTTCCCTCATAAACTTGATATAGATGGCTTAACAATTAAAGAAGCACGTA 855720 855840

NC_000963_1_Rickettsia_prowazekii_str__Madrid_E_chromosome_complete_genome probe_cstm_Rprow_021218_1x_106386 CAAAAATAATAGATATTTTAAAAGAACAGAGTTTACTTACTAAACAAGAAGAAATTATACAAACCGTAAAATGTGCCGAGAGATCAGGTGCTCCTCTTGAGATATTAACCGTCACACAAT 855840 855960

NC_000963_1_Rickettsia_prowazekii_str__Madrid_E_chromosome_complete_genome probe_cstm_Rprow_021218_1x_106387 GGTTTATTAAAACGATAACTCATAAAGAAGCATTACTTAAAAGAACAAACGAACTAAACTGGTATCCTAAAAATATGAAAATGCGCTTAGAGAATTGGATTAATTCTCTAAGCTGGGATT 855960 856080

NC_000963_1_Rickettsia_prowazekii_str__Madrid_E_chromosome_complete_genome probe_cstm_Rprow_021218_1x_106388 GGTGCATCAGTCGACAACGTTATTTTGGTGTACCGTTTCCTATTTGGTACTCTAAAAGGATTGGCGAAGAAGGTAAAATTTTATATGCTGATATCTCACAACTACCTGTTGACCCACTAA 856080 856200

NC_000963_1_Rickettsia_prowazekii_str__Madrid_E_chromosome_complete_genome probe_cstm_Rprow_021218_1x_106389 AAGATTTACCTATAGGTTATAGTAAGGAGGAAGTCGATCCTGACTTAGATGTTATGGATACTTGGGCGACAAGTTCTGTTTCACCTCAACTTTCTACCTATGGCATCTCTGAAGATTTAG 856200 856320

NC_009488_1_Orientia_tsutsugamushi_Boryong_complete_genome probe_cstm_Rprow_021218_1x_10639 TTAGTGAGTTATTAACAATAGCGATATATTTTTATGTATCTCCATGCAAGGATTGTAAAAATTACTATCTATATTACTTGAGTCATAAGTATAAAAGATACTTTTGTTTACCAAGTTATA 1276560 1276680

NC_000963_1_Rickettsia_prowazekii_str__Madrid_E_chromosome_complete_genome probe_cstm_Rprow_021218_1x_106390 CAATTAATAAAGTAAGACACGATAAATTATTTCCGATGGATTTAAGACCACAAGCACATGAAATTATTAGGACTTGGGCATTTTATACAATCCTGAAATCGCATTTGCATCAAAATATTT 856320 856440

NC_000963_1_Rickettsia_prowazekii_str__Madrid_E_chromosome_complete_genome probe_cstm_Rprow_021218_1x_106391 TACCATGGAAAAATATCATGGTAAGCGGTTGGTGTTTAGCTGAAGATCGCAGTAAAATGTCAAAATCGAAGGGCAATGTTTTAGTCCCTGAAAAATTGCTAGAACGATATGGTGCTGACG 856440 856560

NC_000963_1_Rickettsia_prowazekii_str__Madrid_E_chromosome_complete_genome probe_cstm_Rprow_021218_1x_106392 TAATACGTTATTGGTCAGCAAATTCAAAACTTGGAGCTGACACTGCTTACTCAGAAGACGTAATGAAAAATGGGAAAAGACTTGTAAACAAGCTATGGAATGCTGCTAAATTTGTTTCTA 856560 856680

NC_000963_1_Rickettsia_prowazekii_str__Madrid_E_chromosome_complete_genome probe_cstm_Rprow_021218_1x_106393 TACATTTTGATAAACTTACAAGTGAAGATAAAAAAGTGAGTTTGTTTGATATAAAAGAGAAAATTACTAACGAATTTGATCAATGGATGATTAATAAGCTAGTAGCACTAGTTAAACTAG 856680 856800

NC_000963_1_Rickettsia_prowazekii_str__Madrid_E_chromosome_complete_genome probe_cstm_Rprow_021218_1x_106394 CTACAAATGCACTACAAAATTACGAATATGCAAACGCTATTTATCTAACAGAAAAATTCTTTTGGTCTATATTTTGTGATAATTATTTAGAAATAAGCAAAACAAGAAGCTATGACGAGG 856800 856920

NC_000963_1_Rickettsia_prowazekii_str__Madrid_E_chromosome_complete_genome probe_cstm_Rprow_021218_1x_106395 CGAACAAAAACCCACAAGGACAATATAGTAGCATATTAACTTTATATCATATTATGCAAACCTTACTGAAACTATTTGCTCCTTTCATGCCTCATATTACCGAAGAACTATATCAAATAT 856920 857040

NC_000963_1_Rickettsia_prowazekii_str__Madrid_E_chromosome_complete_genome probe_cstm_Rprow_021218_1x_106396 TATATAACAAAAATTCTATTCACATGCAAGGTAACTGGATTAATTATGGTGACTTAAACTATGAAATCGATGTACAAGGACCAGAAGGACTGCTTGAAATACTAGATATCGTCAGAAAAT 857040 857160

NC_000963_1_Rickettsia_prowazekii_str__Madrid_E_chromosome_complete_genome probe_cstm_Rprow_021218_1x_106397 TTAAAGCTGAGTATAATCTATCTATAAAAGCACCAATAAAATTGCTTGAAGTTAGTGGCATAGTATTATCTACAGAATTAGTAGAAGATTTAAAAAACGTCACATCAGCAGAAGAAATAC 857160 857280

NC_000963_1_Rickettsia_prowazekii_str__Madrid_E_chromosome_complete_genome probe_cstm_Rprow_021218_1x_106398 AATTTAAGGCAAAAGATGATCAAATTAAAGTCAATATTAAACTTTTTGTATAACATAACTAATAGAGATGGATTTAAAGGATACAAGGAATGCAACGCTGCTGCATACAAAAACGTATGT 857280 857400

NC_000963_1_Rickettsia_prowazekii_str__Madrid_E_chromosome_complete_genome probe_cstm_Rprow_021218_1x_106399 AATGATTCAAGTACCAAATCAACGTCTAAATTAGCTTTAATAATAAGTTATACACAAAATCTATTATCATTTAAAAACATTATAGGCTTAATGTTAATTATATTTGCAGGTATATTATTT 857400 857520

NC_009488_1_Orientia_tsutsugamushi_Boryong_complete_genome probe_cstm_Rprow_021218_1x_1064 ACACATGAAATAGTTGATGCAATGGAATGGGTGCAAAATGGTCTTGGAGTACAGATAGACATTCATAAGCTTCATAGAGGAGATAAAAACTGGCATGCGCATATATTGGTTACTACAAGA 127560 127680

NC_009488_1_Orientia_tsutsugamushi_Boryong_complete_genome probe_cstm_Rprow_021218_1x_10640 GTAGAATAATACAACTGTGGCCTAGAATGTTACTACCGCTAGATTAATGCATTATCTGAGAGGAGAAGAGACTGGTATATATTACATCGATTCTACAAAGTTAGCAATTTGTCATAATAA 1276680 1276800

NC_000963_1_Rickettsia_prowazekii_str__Madrid_E_chromosome_complete_genome probe_cstm_Rprow_021218_1x_106402 GACGAAATACATTTACAACAAATATTACCACAATTTGAGCAATTAGGATATCGTGTCAAACATAATAAAATCTATGTAATTTGTGGAGAAAGATGCTTAGATAtttttttATTTCATAAG 857760 857880

NC_000963_1_Rickettsia_prowazekii_str__Madrid_E_chromosome_complete_genome probe_cstm_Rprow_021218_1x_106403 GAAAAGGATAAATTTATTCATGTTATATATGATAAATATCCAAATGACtttttttATGATCATGAATTATATCCTTTaaaaaaaTATAAATTTGGTAGTATAGAAGTCTATGGTCCATCT 857880 858000

NC_000963_1_Rickettsia_prowazekii_str__Madrid_E_chromosome_complete_genome probe_cstm_Rprow_021218_1x_106404 GATCCTATAGGCAATTTAAATAGACAATATCCAGAATGGGATAAATATGCAATAATATACAGTCCTCATAGTTTACATCTACCTTTTTTATCAAATATTGaaaaaaaGAGTAAATTTATA 858000 858120

NC_000963_1_Rickettsia_prowazekii_str__Madrid_E_chromosome_complete_genome probe_cstm_Rprow_021218_1x_106405 CTTACTCCAGAATTGTTAAAGCCAGCTCAACCGCTTGGACCTTTAGAAGACAGAGTAAATCTTTTTTGAAAATATTAAATCAGAATTGAAAATGTAATTTCAGGTTCAAAGGTGATGAAA 858120 858240

NC_000963_1_Rickettsia_prowazekii_str__Madrid_E_chromosome_complete_genome probe_cstm_Rprow_021218_1x_106406 TTAAATACGGTATTATCATTTAAAAACATTATAGGCTTAATGTTAATTATATTTGCAGGTATATTATTTTATGCTTATATATTACAACATGAATGGCAATATGTAACCTTAAGTGATGAA 858240 858360

NC_000963_1_Rickettsia_prowazekii_str__Madrid_E_chromosome_complete_genome probe_cstm_Rprow_021218_1x_106408 CTTGGAGCTGTTAGACACCAAGGCATCATTCCTTTTGATGATGATTTAGATATCGGGATTATGCATGAAGACGAAATACATTTACAACAAATATTACCACAATTTGAGCAACTCGGCTAT 858480 858600

NC_000963_1_Rickettsia_prowazekii_str__Madrid_E_chromosome_complete_genome probe_cstm_Rprow_021218_1x_106409 ACTGTTTCCTATGAGAGAGCTTATAATATATGCaaaaaaaCTTGTTTAGATATTTTTATTGTCCATAAAGaaaaaaaCAAATTTATATATACTAATATAATGCTTCGAGATAAGTATCCG 858600 858720

NC_009488_1_Orientia_tsutsugamushi_Boryong_complete_genome probe_cstm_Rprow_021218_1x_10641 ACGCACCTCCAGCAATAGAGTTTTTAACAGAATTTCTAAAATTGCTAAGAGTAGTTATGGCTGGTTCTTATGCTTTAAGCTACATATTATAATCAATAATAAAGGCGAAATAATGTCAGT 1276800 1276920

NC_000963_1_Rickettsia_prowazekii_str__Madrid_E_chromosome_complete_genome probe_cstm_Rprow_021218_1x_106410 GAACATTTCTTTTATGATCATGAGTTATATCCTTTaaaaaaaTATAAATTTGGTAGTATAGAAGTCTATGGTCCATCTGATCCTATAGGCAATTTAAATAGACAATATCCAGAATGGGAT 858720 858840

NC_000963_1_Rickettsia_prowazekii_str__Madrid_E_chromosome_complete_genome probe_cstm_Rprow_021218_1x_106412 TTAGAAGATAATGTGAATATAGTGAATTCAGCTAAATTTATTCGTATTTACAAGGATATAGATGATCATTAAATGTTGAATTTATTATAACCATTCCAATATTTATAACAAAATGATCAC 858960 859080

NC_000963_1_Rickettsia_prowazekii_str__Madrid_E_chromosome_complete_genome probe_cstm_Rprow_021218_1x_106413 ATAAATTATAAAACACATGCCTAGTTTATTACTACTTACTACAACTATACTACTTATTTTATGCGTTTTAATAATTTGGTTTTATATAAAAACTCATACCTTAAAAAGGCAGTTACAATT 859080 859200

NC_000963_1_Rickettsia_prowazekii_str__Madrid_E_chromosome_complete_genome probe_cstm_Rprow_021218_1x_106414 CTTGTCGGAACAGAATCTAGAAATTAATAATAATAATCGATTACTGAATCAAGAAAAAATAGCTTATTTACAAAAAATTGAGCAATTGAAGTGTAAAGTAGAGTATCAAGAGCAGATGAT 859200 859320

NC_000963_1_Rickettsia_prowazekii_str__Madrid_E_chromosome_complete_genome probe_cstm_Rprow_021218_1x_106415 TAAGGACTCAGAAAAAATAAGAGAAGAGTCATTTACATCAGCTAAAGCTGCGTTATTTGATTTAGGTAAGGATTTATCTAAACAATTAATCGAAATTCATAAAATAGAAAATAATACAGC 859320 859440

NC_000963_1_Rickettsia_prowazekii_str__Madrid_E_chromosome_complete_genome probe_cstm_Rprow_021218_1x_106416 AAGAGAGCTAGCCGAGCAAAATATCACGACCGCTTCTCGCAAATTTAATAGTGAGCTTGAACGATTAATTACGATGGTTGGGGCATTAAATAAAGATATTGAGCAATCAAAAAGTACAGT 859440 859560

NC_000963_1_Rickettsia_prowazekii_str__Madrid_E_chromosome_complete_genome probe_cstm_Rprow_021218_1x_106417 TGATTTGATAAAACAGTCACTACTTTCGCCTATCGGGGCAGGGTTGCTTTCTGAAATTACACTTGAGAATATCCTCAAATCTTCAGGATTACGTCCTAATTTAGATTTTATAATGCAATA 859560 859680

NC_000963_1_Rickettsia_prowazekii_str__Madrid_E_chromosome_complete_genome probe_cstm_Rprow_021218_1x_106418 TGGTCTCACTACATCAGATAGTGTCAAGCTAAGACCTGATGCAATCATTTTTCTTCCTTCAGGCAATTTAATGGTTATTGATTCCAAAGCTTCAAAGTTTCTAGTAGACTCTCAGGATAA 859680 859800

NC_000963_1_Rickettsia_prowazekii_str__Madrid_E_chromosome_complete_genome probe_cstm_Rprow_021218_1x_106419 TAGCGTGAATCTTAGTAAAACTATGAACTATCATTTGAAATCTCTCGCTAATAAAGACTATGCTGAAAATATTTTAACTACTCTAAACAAAAAGGCTCACAACTTTAACAATGTTATTAC 859800 859920

NC_009488_1_Orientia_tsutsugamushi_Boryong_complete_genome probe_cstm_Rprow_021218_1x_10642 TAAAATTACTAAAGGCAATAAAAACGACCTTATCTGTAGCTTTAGTTTTTTCTAAAGGCTTATCTGGTAAATTGTTTGGTGATAAAGCTACATATCTAAAGAGTTATTTCATCAACTGTT 1276920 1277040

NC_000963_1_Rickettsia_prowazekii_str__Madrid_E_chromosome_complete_genome probe_cstm_Rprow_021218_1x_106420 TTTAATGTTTTTGCCAACTGAACAAGCGGTTGAAAAGGTTATTGCAGCCAATCCTGAATTTTTACAAAAAGCTTGGGGATGTAATATTTTTCCGGTAGGGCCTGCAGGACTGATGAATAT 859920 860040

NC_000963_1_Rickettsia_prowazekii_str__Madrid_E_chromosome_complete_genome probe_cstm_Rprow_021218_1x_106421 GTTATCATTTGCAAAATTTCAAATTACCGATAATCGTCGGTCAGAAAATTATAAGGTAATAATTTCAGAAGTTAGAAAATTGTTAAGTTCTATAGGTACTATAGCTGATTATTCCaaaaa 860040 860160

NC_000963_1_Rickettsia_prowazekii_str__Madrid_E_chromosome_complete_genome probe_cstm_Rprow_021218_1x_106422 aaTTGGTTATAACCTACAAAATATGGTTACTAATTATGATAAATTTGCTGCCTCGTTTAACCGTAATTTAATGTCAAGAGTGAAAACTATTCAAAAACTTGGTATTGATTCAGGAGATAA 860160 860280

NC_000963_1_Rickettsia_prowazekii_str__Madrid_E_chromosome_complete_genome probe_cstm_Rprow_021218_1x_106423 AGCGATGCCTGCTACCTTAGAGCGTTATCAGATTGTTTCTTCTAAATCAGAAATTATTGAAGTAGATGCAGAAAATCCAACACAAATAGAAGAATAATATATAATTAAATGTTAATGTTC 860280 860400

NC_000963_1_Rickettsia_prowazekii_str__Madrid_E_chromosome_complete_genome probe_cstm_Rprow_021218_1x_106424 CATAGTGGTGCGGTGTCAAAATAtttttttATGTAAGCCATATCTATTATTAAAGCTCCTAAAAAAGAAAATACACATGTTATAAGTGTAAGTTTAATTAAGGCATGGATTGATTTTACA 860400 860520

NC_000963_1_Rickettsia_prowazekii_str__Madrid_E_chromosome_complete_genome probe_cstm_Rprow_021218_1x_106425 ATAGACATAAAAACGTACAAATTGTTATAGTGATCATAATAATTATAACATTATTATTGTGTTATGGTAGAAGAACTGTTAGTAATTAACAAATAAGAATACCTTGAATATATCAAAAAA 860520 860640
[truncated: 13,035,172 more chars]
