# Supplementary material for: A proof of concept for a targeted enrichment approach to the simultaneous detection and characterization of rickettsial pathogens from clinical specimens
Source: Front Microbiol. 2024 Apr 10;15:1387208. doi: 10.3389/fmicb.2024.1387208 (PMC11039911; doi:10.3389/fmicb.2024.1387208)
Supplement: Supplementary file 2 [file Table_2.docx]

Supplementary Material

**Table S2: A) Ct values (with replicates) for 15 sequencing libraries made from *R. prowazekii* Breinl spiked blood samples and analyzed by qPCR after enrichment. B) Copy number of sequencing libraries made from *R. prowazekii* Breinl spiked blood samples analyzed by qPCR after enrichment.**

| A. | Tube 1 | | Tube2 | | Tube3 | |
| --- | --- | --- | --- | --- | --- | --- |
|  | 1-a | 1-b | 2-a | 2-b | 3-a | 3-b |
| Spiked Copy#/µl |  |  |  |  |  |  |
| 0 | - | - | - | - | - | - |
| 1 | 27.55 | 27.33 | 26.04 | 26.03 | 29.17 | 29.17 |
| 3 | 24.48 | 24.48 | 23.2 | 23.17 | 24.61 | 24.6 |
| 10 | 22.66 | 22.61 | 22.74 | 22.76 | 22.09 | 22.13 |
| B. 30 | 21.07 | 21.11 | 20.89 | 20.88 | 21.03 | 21.02 |
| Spiked Copy#/µl |  | |  | |  | |
|  | Tube 1 | Tube 2 | Tube 3 | Average | Std Dev |  |
| 0 | 0 | 0 | 0 | 0 | 0 |  |
| 1 | 56060 | 70110 | 38760 | 54977 | 31406 |  |
| 3 | 85660 | 98610 | 84410 | 89560 | 15725 |  |
| 10 | 104110 | 102960 | 109360 | 105477 | 6824(6.5%) |  |
| 30 | 119560 | 121610 | 120210 | 120460 | 2095 (1.7%) |  |
