# Supplementary material for: A proof of concept for a targeted enrichment approach to the simultaneous detection and characterization of rickettsial pathogens from clinical specimens
Source: Front Microbiol. 2024 Apr 10;15:1387208. doi: 10.3389/fmicb.2024.1387208 (PMC11039911; doi:10.3389/fmicb.2024.1387208)
Supplement: Supplementary file 3 [file Table_3.docx]

Supplementary Material

**Table S3: Summary of clinical parameters associated with pathogen groups.** Values are medians with the range and number of patients given.

|  | *O. tsutsugamushi* Negative  (overall n= 54) | | | *O. tsutsugamushi* Positive  (overall n= 8) | | |  |
| --- | --- | --- | --- | --- | --- | --- | --- |
| Clinical Parameter | Median | Range | n= | Median | Range | n= | p-value |
| WBC | 1.21E+10 | 3.1E+09-4.2E+10 | 51 | 1.14E+10 | 7.4E+09-1.4E+10 | 8 | 0.3121 |
| Granulocyte Count | 1E+10 | 1E+09-4E+10 | 51 | 8.3E+09 | 4.8E+09-1.05E+10 | 8 | 0.2343 |
| Granulocyte % | 82 | 29-96.4 | 51 | 76.25 | 53-89 | 8 | 0.1607 |
| Lymphocyte/Monocyte Count | 2.2E+09 | 1.5E+8-6.4E+9 | 53 | 2.2E+09 | 4E+08-4.8E+10 | 8 | 0.9373 |
| Lymphocyte/Monocyte % | 18 | 6-71 | 51 | 23.75 | 11-47 | 8 | 0.1893 |
| Hgb (g/dL) | 11.9 | 7.2-18.2 | 54 | 12.4 | 9.7-14.6 | 8 | 0.8975 |
| Hct (%) | 36.6 | 13.3-55.9 | 54 | 38 | 27.6-45.5 | 8 | 0.9057 |
| Platelet | 2.07E+11 | 1.6E+10-8.9E+11 | 53 | 1.26E+11 | 4.2E+10-2.6E+11 | 8 | 0.0206 |
| Na+ (mmol/L) | 132 | 104-142 | 54 | 128.5 | 123-135 | 8 | 0.1414 |
| K+ (mmol/L) | 3.7 | 2.2-5.8 | 51 | 4.15 | 3.7-4.8 | 8 | 0.0219 |
| Ca++ (mmol/L) | 1.125 | 0.87-1.35 | 54 | 1.1 | 1.02-1.2 | 8 | 0.3587 |
| BUN (mg/dL) | 13 | 7-144 | 52 | 19 | 11-67 | 8 | 0.0243 |
| CRE (mg/dL) | 0.8 | 0.4-13.3 | 49 | 1 | 0.5-4.6 | 8 | 0.208 |
| Glu (mg/dL) | 125.5 | 71-507 | 54 | 99 | 82-250 | 8 | 0.0428 |
| ALB (g/dL) | 2.55 | 1.4-4.2 | 52 | 2.05 | 1.7-2.7 | 8 | 0.0021 |
| ALT (u/µL) | 61 | 17-313 | 51 | 185.5 | 106-322 | 8 | 0.0276 |
| AST (u/µL) | 50 | 9-480 | 51 | 85.5 | 63-156 | 8 | <0.0001 |
| Lac (mg/dL) | 23.25 | 9.4-84.5 | 52 | 21.05 | 10.1-54.6 | 8 | 0.7208 |
| pH | 7.46 | 7.21-7.58 | 52 | 7.45 | 7.40-7.51 | 8 | 0.5675 |
| pC02 (mmHg) | 30.6 | 20.5-54.8 | 52 | 25 | 22.3-37.3 | 8 | 0.022 |
| p02 (mmHg) | 78.05 | 21-220.4 | 52 | 49.55 | 28.3-230.2 | 8 | 0.4082 |
| Heart Rate | 100 | 68-162 | 54 | 101.5 | 88-124 | 8 | 0.6311 |
| Respiration Rate | 24 | 16-38 | 54 | 26 | 22-46 | 8 | 0.1947 |
| Temperature | 38 | 36.3-40 | 54 | 38.25 | 37-40 | 8 | 0.6292 |
| Age | 46 | 18-77 | 54 | 42 | 26-62 | 8 | 0.8007 |
| Hospital Duration (days) | 8 | 1-73 | 53 | 5 | 4-13 | 8 | 0.2158 |

*Notes: Bolded value indicates the value is significantly different in patients with O. tsutsugamushi infection as compared to those with other infections. WBC, White Blood Count; Hgb, Hemoglobin; Hct, Hematocrit; Na, Sodium; K, Potassium; Ca, Total Calcium; BUN, Blood Urea Nitrogen; CRE, Creatinine; Glu, Glucose; ALB, Albumin; ALT, Alanine Aminotransferase; AST, Aspartate aminotransferase; Lac, Lactate*
