# Supplementary material for: A proof of concept for a targeted enrichment approach to the simultaneous detection and characterization of rickettsial pathogens from clinical specimens
Source: Front Microbiol. 2024 Apr 10;15:1387208. doi: 10.3389/fmicb.2024.1387208 (PMC11039911; doi:10.3389/fmicb.2024.1387208)
Supplement: Supplementary file 4 [file Table_4.docx]

Supplementary Material

**Table S4. Clinical symptoms associated with sepsis patients infected with and without *O****.* ***tsutsugamushi***

|  | *O. tsutsugamushi* Negative | | *O. tsutsugamushi* Positive | |  |
| --- | --- | --- | --- | --- | --- |
| Clinical Symptom | Percent | n= | Percent | n= | p-value |
| Fever | 92.59% | 50 | 100.00% | 8 | >0.9999 |
| Rigors | 75.93% | 41 | 87.50% | 7 | 0.6697 |
| Sweat | 66.67% | 36 | 87.50% | 7 | 0.4158 |
| Dizzy | 51.85% | 28 | 62.50% | 5 | 0.7126 |
| Headache | 66.67% | 36 | 87.50% | 7 | 0.6027 |
| Retro-orbital pain | 31.48% | 17 | 50.00% | 4 | 0.4259 |
| Blurred vision | 44.44% | 24 | 75.00% | 6 | 0.1411 |
| Hearing | 31.48% | 17 | 37.50% | 3 | 0.7052 |
| Confusion | 3.70% | 2 | 37.50% | 3 | 0.013 |
| Stiff neck | 3.70% | 2 | 12.50% | 1 | 0.3442 |
| Sore throat | 29.63% | 16 | 75.00% | 6 | 0.0193 |
| Swollen glands | 1.85% | 1 | 0.00% | 0 | >0.9999 |
| Shortness of breath | 74.07% | 40 | 87.50% | 7 | 0.6665 |
| Palpitations | 50.00% | 27 | 37.50% | 3 | 0.7085 |
| Cough | 74.07% | 40 | 75.00% | 6 | >0.9999 |
| Joint pain | 31.48% | 17 | 50.00% | 4 | 0.4259 |
| Muscle soreness | 48.15% | 26 | 75.00% | 6 | 0.2577 |
| Fatigue | 90.74% | 49 | 100.00% | 8 | >0.9999 |
| Anorexia | 81.48% | 44 | 75.00% | 6 | 0.6455 |
| Abdominal pain | 51.85% | 28 | 100.00% | 8 | 0.0164 |
| Nausea | 31.48% | 17 | 50.00% | 4 | 0.4259 |
| Diarrhea | 14.81% | 8 | 50.00% | 4 | 0.0387 |
| Swelling | 3.70% | 2 | 25.00% | 2 | 0.0774 |
| Itching | 7.41% | 4 | 12.50% | 1 | 0.5113 |
| Rash | 7.41% | 4 | 12.50% | 1 | 0.5113 |
| Skin lesions | 5.56% | 3 | 0.00% | 0 | >0.9999 |

*** *Complaints of symptoms prior to hospitalization collected during enrollment. Bolded value indicates the value is significantly different in patients with O. tsutsugamushi infection as compared to those with other infections.*
