# Supplementary material for: A proof of concept for a targeted enrichment approach to the simultaneous detection and characterization of rickettsial pathogens from clinical specimens
Source: Front Microbiol. 2024 Apr 10;15:1387208. doi: 10.3389/fmicb.2024.1387208 (PMC11039911; doi:10.3389/fmicb.2024.1387208)
Supplement: Supplementary file 5 [file Table_5.docx]

Supplementary Material

**Table S5.** **Pairwise comparisons of *R. typhi* and *R. prowazekii***

| Strain | GenBank Entry | Source | Genome length (Mbp) | % identity to *R. prowazekii* Breinl | % identity to *R. prowazekii* Madrid |
| --- | --- | --- | --- | --- | --- |
| Wilmington | NC_006142.1 | ATCC VR-144 | 1.11150 | 94.07 | 94.09 |
| TH1527 | NC_017066.1 | Human (Thailand) | 1.11237 | 94.07 | 94.09 |
| B9991CWPP | NC_017062.1 | Bandicoot (Burma) | 1.11296 | 94.07 | 94.09 |
| TM2540 | NZ_L992663.1 | Human (Laos) | 1.11194 | 94.02 | 94.04 |
