# Supplementary material for: A proof of concept for a targeted enrichment approach to the simultaneous detection and characterization of rickettsial pathogens from clinical specimens
Source: Front Microbiol. 2024 Apr 10;15:1387208. doi: 10.3389/fmicb.2024.1387208 (PMC11039911; doi:10.3389/fmicb.2024.1387208)

Supplementary Material

Figure S1. Breadth of coverage from *R. prowazekii* spiked singly or as a contrived coinfection and prepared for sequencing using targeted enrichment. A) Coverage resulting from spiked-in concentrations of 25 and 250 gc/mL; **B)** Coverage resulting from spiked-in concentration of 12 gc/mL in combination with 12 gc/mL *O. tsutsugamushi.* Reads are mapped to *R. prowazekii* reference NC_020993.

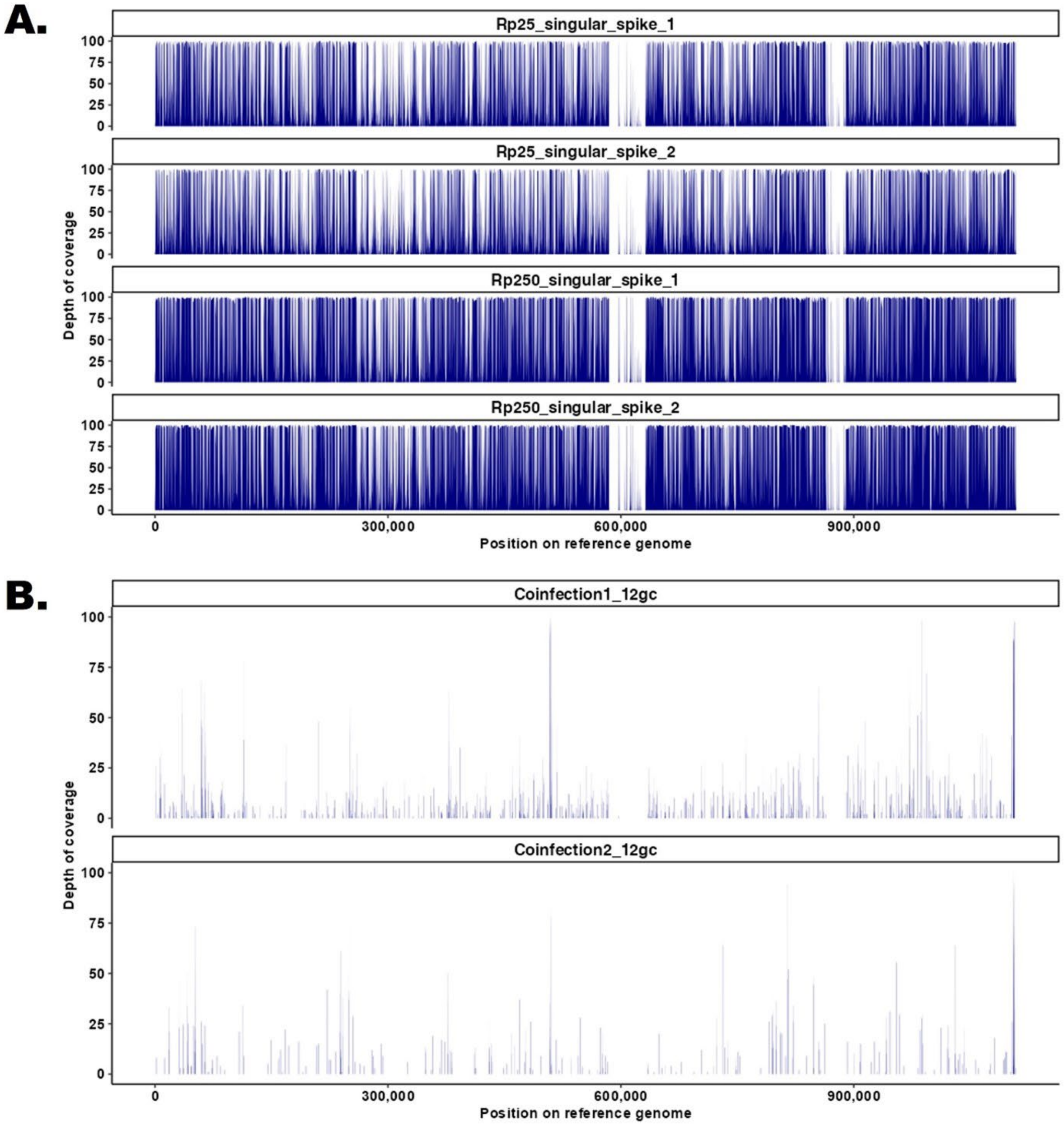

Supplement: Supplementary file 6 [file Image_1.pdf]
