# Supplementary material for: A proof of concept for a targeted enrichment approach to the simultaneous detection and characterization of rickettsial pathogens from clinical specimens
Source: Front Microbiol. 2024 Apr 10;15:1387208. doi: 10.3389/fmicb.2024.1387208 (PMC11039911; doi:10.3389/fmicb.2024.1387208)

Supplementary Material

Figure S2. Phylogeny of partial 338 bp fragment of *tsa56* sequences demonstrates relatedness to known *O. tsutsugamushi.* strains. Alignments of 338 bp fragments of *tsa56* sequences were used to generate a Maximum Likelihood tree. Four samples (CAMB_01, CAMB_02, CAMB_04, and CAMB_05) were more closely related to the Kato strain and two samples (CAMB_03 and CAMB_06) were more closely related to Karp, UT76, and UT176 strains. Samples CAMB_07 and CAMB_08 are not included in the tree because *tsa56* was not present in the assemblies for these samples.

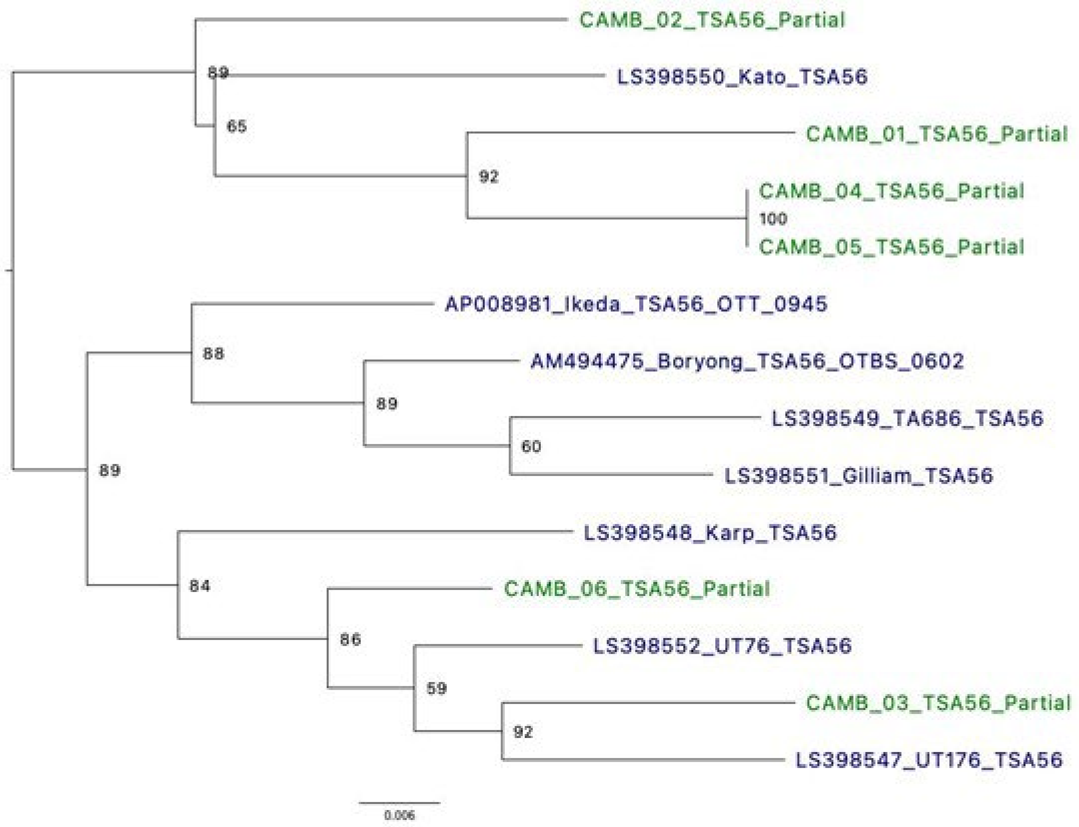

Supplement: Supplementary file 7 [file Image_2.pdf]
