# Supplementary material for: A proof of concept for a targeted enrichment approach to the simultaneous detection and characterization of rickettsial pathogens from clinical specimens
Source: Front Microbiol. 2024 Apr 10;15:1387208. doi: 10.3389/fmicb.2024.1387208 (PMC11039911; doi:10.3389/fmicb.2024.1387208)

Supplementary Material

Figure S3. Multiple sequence alignment of *R. typhi* vs. *R. prowazekii* illustrates overall genome synteny and identity, indicating our assay will be sufficient to enrich for *R. typhi*.

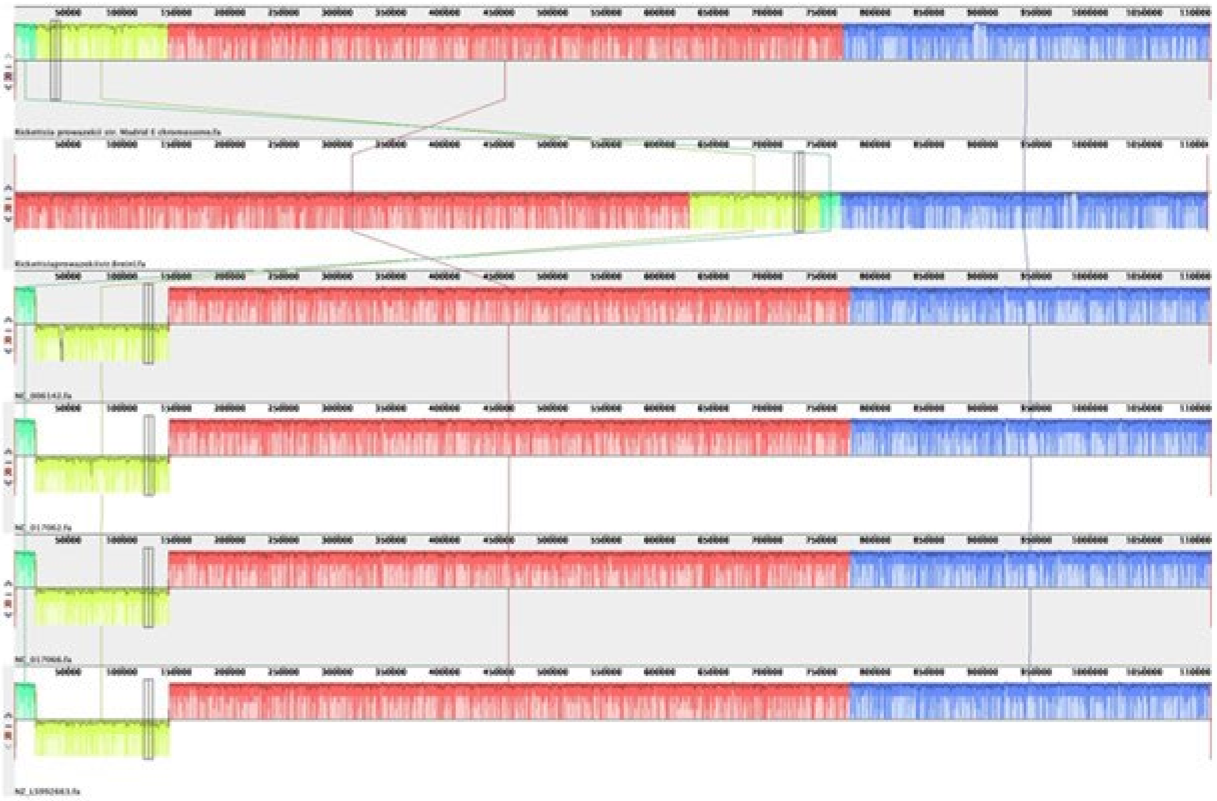

Supplement: Supplementary file 8 [file Image_3.pdf]
